# Native trees of Mexico: diversity, distribution, uses and conservation

**DOI:** 10.7717/peerj.9898

**Published:** 2020-09-18

**Authors:** Oswaldo Tellez, Efisio Mattana, Mauricio Diazgranados, Nicola Kühn, Elena Castillo-Lorenzo, Rafael Lira, Leobardo Montes-Leyva, Isela Rodriguez, Cesar Mateo Flores Ortiz, Michael Way, Patricia Dávila, Tiziana Ulian

**Affiliations:** 1 Facultad de Estudios Superiores Iztacala, Av. De los Barrios 1, Los Reyes Iztacala Tlalnepantla, Universidad Nacional Autónoma de México, Estado de México, Mexico; 2 Wellcome Trust Millennium Building, RH17 6TN, Royal Botanic Gardens, Kew, Ardingly, West Sussex, United Kingdom

**Keywords:** Árboles, Biodiversity conservation, Distribution maps, Natural capital, Priority areas, Seed bank, Species richness, Threatened species

## Abstract

**Background:**

Mexico is one of the most floristically rich countries in the world. Despite significant contributions made on the understanding of its unique flora, the knowledge on its diversity, geographic distribution and human uses, is still largely fragmented. Unfortunately, deforestation is heavily impacting this country and native tree species are under threat. The loss of trees has a direct impact on vital ecosystem services, affecting the natural capital of Mexico and people’s livelihoods. Given the importance of trees in Mexico for many aspects of human well-being, it is critical to have a more complete understanding of their diversity, distribution, traditional uses and conservation status. We aimed to produce the most comprehensive database and catalogue on native trees of Mexico by filling those gaps, to support their in situ and ex situ conservation, promote their sustainable use, and inform reforestation and livelihoods programmes.

**Methods:**

A database with all the tree species reported for Mexico was prepared by compiling information from herbaria and reviewing the available floras. Species names were reconciled and various specialised sources were used to extract additional species information, i.e. endemic status, threat status, availability in seed collections, reports on plant uses and conservation actions currently in place. With this information, a comprehensive catalogue of native trees from Mexico was redacted. Available georeferenced records were used to map each species distribution and perform spatial analyses to identify gaps of information and priority areas for their conservation and exploration.

**Results:**

Mexico has at least 2,885 native tree species, belonging to 612 genera and 128 families. Fabaceae is the most represented family and *Quercus* the most represented genus. Approximately 44% of tree species are endemic to the country. The southern part of the country showed the highest values of species richness. Six hundred and seventy-four species have at least one documented human use. In terms of conservation assessment, ca. 33% of species have been assessed by either the IUCN Red List (919) or the National protection catalogue “NORMA Oficial Mexicana NOM-059” (29) or both (45). Additionally, 98 species have been included in the CITES listing for protection. In terms of existing conservation efforts, 19% of species have ex situ protection in seed banks, while protected areas overlap with all the identified peaks of species richness, except for those in the states of Veracruz and Chiapas. This work constitutes a key milestone for the knowledge, management, and conservation of the Mexican native trees. The two areas with high density of tree species identified in Veracruz and Chiapas represent two priority areas for tree conservation in Mexico, where integrated in situ and ex situ conservation efforts should be focused.

## Introduction

The biological, ecological and economic importance of trees is unquestionable. They are the main components of the forest ecosystem biomass, hosting ca. 50% of terrestrial biodiversity (Millennium Ecosystem Assessment, 2005). Tree–based ecosystems play an important role in the earth biogeochemical processes, containing ca. 50% of the available terrestrial carbon (FAO, 2010; Millennium Ecosystem Assessment, 2005), and therefore, they are crucial to mitigate the effects of human driven climate change. Trees and forests also provide vital ecosystem services such as clean water and prevention of soil erosion, as well as many direct benefits for human wellbeing (e.g., food, medicine, timber; Millennium Ecosystem Assessment, 2005). Despite their widely documented importance, only recently a comprehensive assessment of the number of tree species known to science in the world was published. In this assessment, it is suggested that globally there are approximately 60,000 tree species (Beech et al., 2017), representing ca. 15% of all vascular plant species (almost 393,000 species; Willis, 2017). Nearly half of these tree species are found in just 10 families, with the richest families being Fabaceae, Rubiaceae and Myrtaceae, and the countries with the most tree species being Brazil, Colombia and Indonesia (Beech et al., 2017).

Mexico has ca. 23,000 vascular plants and is the fourth most floristically rich country in the world, after Brazil, China and Colombia (Ulloa Ulloa et al., 2017; Villaseñor, 2016). Over 50% of the Mexican plant species are endemic to the country and this high level of endemism is surpassed only by South Africa among mainland countries in the world (Villaseñor, 2016). Despite the richness of Mexico’s flora, its inventory and mapping are incomplete (CONABIO, CONANP, TNC, 2012; Villaseñor, 2016).

Many important studies have been dealing with Mexican tree species, dating as far back as early in the previous century (Barwick, 2004; Pennington & Sarukhán, 2005; Ricker et al., 2013; Ricker & Hernández, 2010; Standley, 1920-1926; Villaseñor & Ibarra-Marníquez, 1998; Villaseñor & Ortiz, 2014), including studies on specific taxonomic groups (Farjon, 1990; Farjon, 2001; Farjon, 2005; Farjon, Pérez de la Rosa & Styles, 1997; Sousa, Ricker & Hernández, 2001), and regional floras, such as the Vascular Plants of the Americas, Flora of North America, Flora Neotrópica, Flora Mesoamericana, Flora of Veracruz, Flora of Bajío, Flora Novo-Galiciana, Flora of Guerrero, Flora of Jalisco, and Flora of the Tehuacán-Cuicatlán Valley. Other significant efforts contributing to knowledge of the Mexican trees include regional studies, such as in the state of Veracruz (Ibarra-Manríquez & Sinaca, 1995), in the Yucatán Peninsula (Ibarra-Manríquez, Villaseñor & Durán-García, 1995), in the state of Sonora (Felger, Johnson & Wilson, 2001), and in the central and northern regions of Mexico (Villanueva-Díaz et al., 2006).

A recent study suggested that Mexico has 3,364 tree species, and it is therefore among the top ten countries for tree species richness (Beech et al., 2017), with figures rising to ca. 3,500 species according to the last updates (GlobalTreeSearch, 2019). However, the focus of this study, being at a global level, lacked comprehensive species information at country level. It also lacked information on distribution, conservation status and uses of Mexico’s trees. Given the importance of trees for many aspects of human well-being, including material and non-material goods (e.g., food and medicinal resources, and recreational experiences, respectively) and regulation services (e.g., carbon sequestration), the need to have a more complete understanding on the diversity, distribution and status of Mexico’s trees has been recently recognised as a critical knowledge gap (Sarukhán et al., 2010). This is particularly the case, given that the world’s human population is projected to reach 9.6 billion by 2050. Along with population growth, the demand for energy and wood products for both industrial and domestic uses is expected to increase by 40% in the next 20 years (FAO, 2010). The demand for other forest-related goods (food, medicine, fodder and other commodities) is also predicted to increase (FAO, 2014), putting at risk the global natural resources of forests and their associated traditional knowledge. Loss of forest through land use change is also a serious problem in Mexico. Coverage of tropical and temperate forests in the country represented only 38% of their original extent by 2002, with the largest losses taking place in the tropics (Sarukhán et al., 2010).

To counter the forest loss, some large scale reforestation programmes have been activated in the past few decades in this country (Sarukhán et al., 2010). However the lack of information on native woody species has often led to the use of a few exotic species such as *Eucalyptus* and *Casuarina* spp, in the Millennium Seed Bank (MSB) of despite many woody native species of Mexico being potentially suitable for reforestation purposes (Vázquez-Yanes & Batis, 1996).

In situ conservation measures, such as the protection and restoration of natural habitats, are the best methods of preserving plant diversity (CBD, 2002). In Mexico, the National Commission of Protected Natural Areas (CONANP) currently administers 182 federal natural areas, 176 of which are terrestrial and cover ca. 45 million hectares (CONANP, 2017). Ex situ conservation provides a complementary way to prevent immediate extinctions. Botanic gardens conserve plant diversity and can prevent extinction through integrated conservation actions (Mounce, Smith & Brockington, 2017; Oldfield, 2009), while seed banks allow the preservation of large amounts of genetic material in a small space and with minimum risk of genetic damage (Iriondo & Pérez, 1999), at least for species with orthodox (i.e., desiccation tolerant) seeds. Since February 2002, seeds of Mexican native species were collected and stored at the Facultad de Estudios Superiores, Iztacala (Fes-I) Seed Bank at the National Autonomous University of Mexico (UNAM) and duplicated in the Millennium Seed Bank (MSB) of the Royal Botanic Gardens, Kew (RBG Kew) in the UK, as a result of several collaborative projects between the two institutions (León-Lobos et al., 2012; Rodríguez-Arévalo et al., 2017). In situ and ex situ conservation approaches should be viewed as complementary rather than alternative. However, while there are economic drivers working against in situ conservation, ex situ conservation must still address some technical challenges, particularly for trees (Li & Pritchard, 2009; Pritchard et al., 2014). Around 33% of the tree species worldwide are reported to be likely to produce desiccation sensitive seeds (Wyse & Dickie, 2018) and therefore not suitable for ex situ conservation under standard seedbanking techniques.

Thus, this paper aims to provide and map taxonomic, geographical, ethnobotanical and conservation information on native tree species of Mexico, in order to provide a first, comprehensive account of this incredible natural capital and support their conservation and sustainable use in reforestation and livelihoods programmes.

## Materials & Methods

### Study area

**Figure 1:**
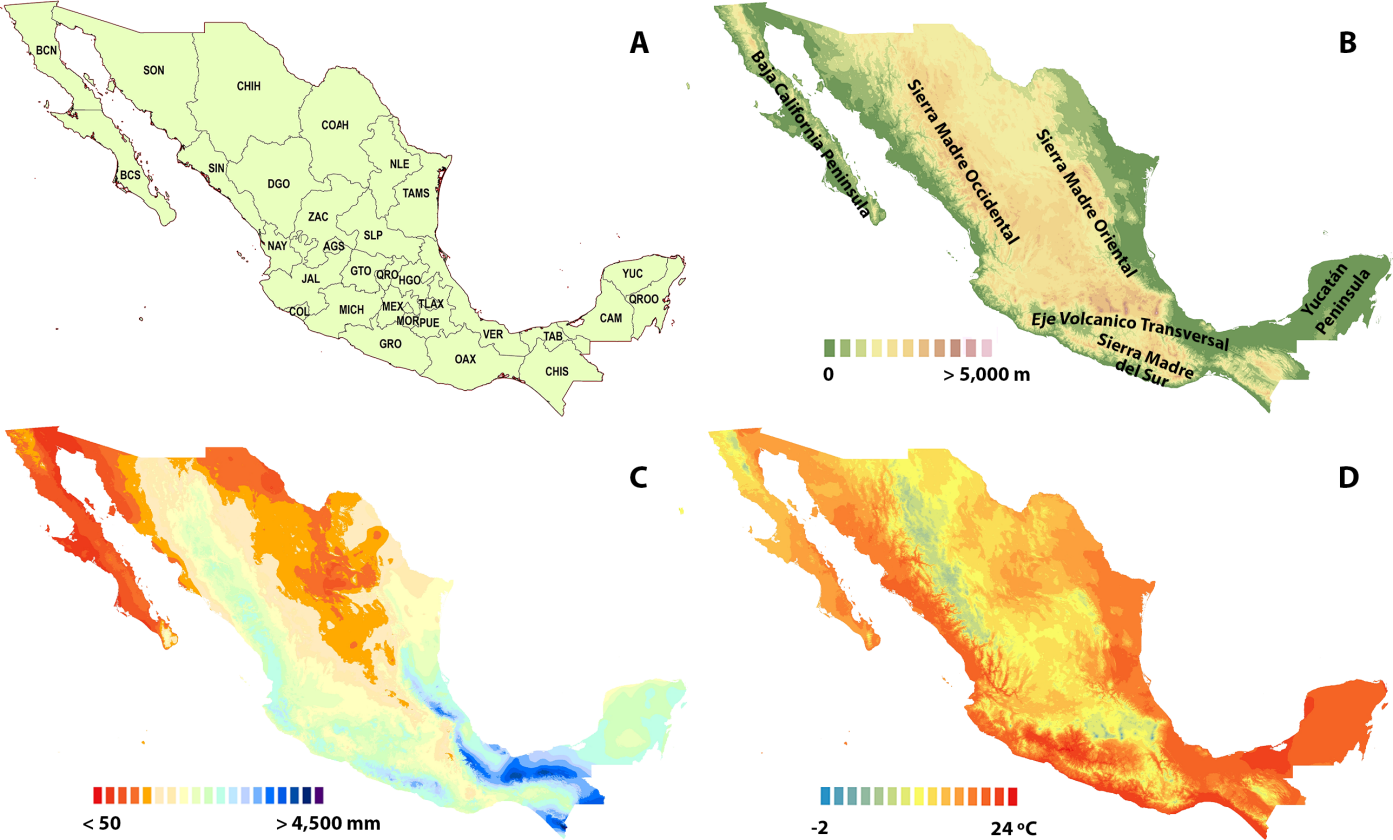
Study area. (A) States of Mexico, labelled with the official abbreviations. (B) Topographic map of Mexico, showing the elevation and the main five geographic features mentioned in the text. (C) Annual precipitation of Mexico (1910–2009). (D) Mean annual temperature of Mexico (1910–2009).

Mexico covers a surface area of 1,964,375 km^2^, extending either side of the Tropic of Cancer, between 32°42′N and 14°30′N of longitude. With an approximate triangular shape, the country reaches ca. 2,000 km of width in the North and only 200 km in the Isthmus of Tehuantepec in the South (Morrone, 2019). Mexico’s territory includes two large peninsulas: Baja California in the North West and Yucatán in the South East. The “Sierra Madre Occidental” is the main mountain range of the country, stretching along the west coast. The “Sierra Madre Occidental” mountain range runs in the East and it is linked to the “Sierra Madre Oriental” in the South through the “Eje Volcánico Transversal”. Finally, the “Sierra Madre del Sur” is found in the South, with a Northwest-Southeast direction, close to the Pacific coast (Fig. 1).

Occupying14th place in land area in the world, Mexico has the fourth richest biota overall, specifically ranking first for reptiles, third for mammals, fifth for vascular flora and amphibians, and eighth for birds, contributing, on average, to the 10% of the global richness of each taxon (Espinosa et al., 2008). Its wealth of ecosystems and its genetic diversity place this country in a privileged position in the world. This high biodiversity is explained by its great physiographic complexity and by its intricate geological and climatic history. The richness of species and endemism of each group are not uniform throughout the Mexican territory, but show geographic trends and discontinuities (Espinosa et al., 2008).

### Tree definition and study limitations

**Figure 2:**
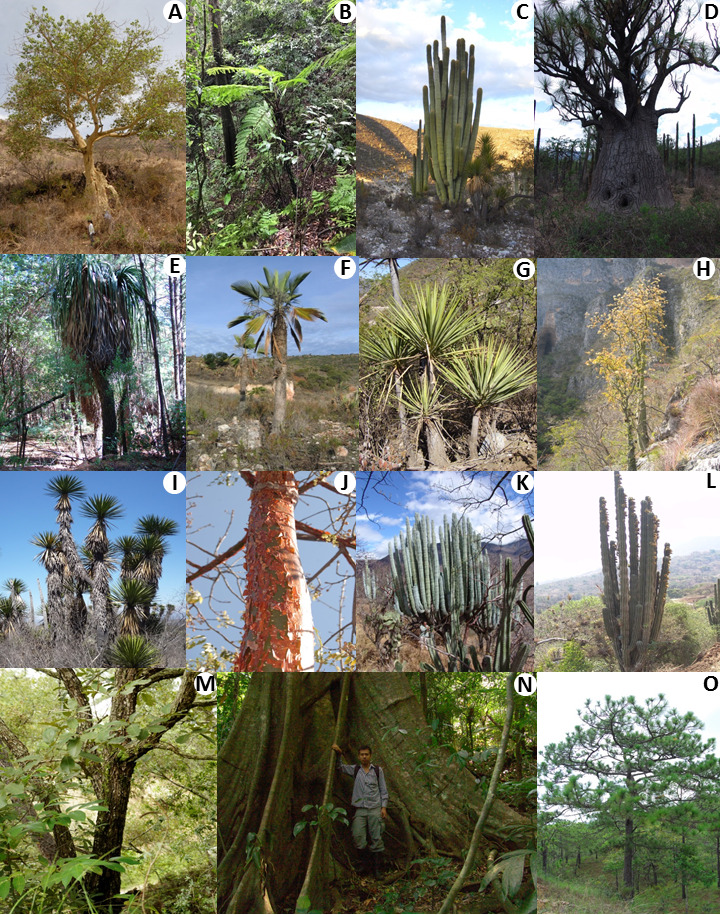
Overal habits of the species included in the study. (A) *Ficus petiolaris* (Moraceae). (B) *Alsophila firma* (Cyatheaceae). (C) *Cephalocereus fulviceps* (Cactaceae). (D) *Beaucarnea gracilis* (Asparagaceae). (E) *Nolina parviflora* (Asparagaceae). (F) *Brahea dulcis* (Arecaceae). (G) *Yucca mixtecana* (Asparagaceae). (H) *Fouquieria purpusii* (Fouquieriaceae). (I) *Yucca periculosa* (Asparagaceae). (J) *Bursera simaruba* (Burseraceae). (K) *Pachycereus weberi* (Cactaceae) (L) *Pachycereus grandis* (Cactaceae). (M) *Quercus magnoliifolia* (Fagaceae). (N) *Vatairea lundellii* (Fabaceae). (O) *Pinus pseudostrobus* (Pinaceae). All pictures by Oswaldo Tellez, except for (M) and (O) by Cesar Miguel Talonia.

Despite the existence of various tree definitions (Fernald, 1993; Font Quer, 1979; Moreno, 1984; Sousa & Zárate, 1983; Rzedowski & Rzedowski, 2001), in this work we used a broader definition, similar to that utilized in Beech et al. (2017), including all woody species showing a single conspicuous stem, which in the upper part produces branches that form a canopy. Our definition differs from that of Beech et al. (2017) by requiring height of at least 4 m, and DBH of at least 10 cm. Therefore, species of Arecaceae, Asparagaceae, and Cactaceae which complied with this definition were included as they are commonly referred to by local people as “árboles” (i.e., trees in Spanish), see Fig. 2 for some examples. Considering that the focus of this study was on native Mexican trees, we excluded exotic, cultivated and naturalized species. We only included species for which the status of tree was confirmed in Mexico by scientific literature records, herbarium specimens and lifetime field expertise of the Mexican taxonomists among the co-authors of this study. We excluded those species that are reported as trees outside Mexico, but that can be found only as shrubs in the country, as for example *Rauvolfia tetraphylla* L. (Apocynaceae) and *Piper amalago* L. (Piperaceae), which are reported as trees in Honduras and Puerto Rico, respectively, but only as shrubs in Mexico.

### Species list

The list, similar to that in Beech et al. (2017), was restricted to species level and therefore the infraspecific *taxa*, such as subspecies and varieties, were not included. Several bibliographic sources were consulted, including monographs and taxonomic revisions (Borhidi, 2012; Fryxell, 1988; Pennington & Sarukhán, 2005), following the taxonomic treatments proposed in the main floristic projects (e.g., Flora Novo-Galiciana, Flora Mesoamericana, Flora of Bajío, Flora of Guerrero, Flora of Jalisco, Flora of Valle de Tehuacán-Cuicatlán). Also, an online consultation was carried out of four databases: Tropicos (Missouri Botanical Garden, 2020), The World Information Network on Biodiversity (REMIB), The National Information System on Biodiversity (SNIB; CONABIO, 2017) and The Plant List (2013). Herbarium specimens of the National Autonomous University of Mexico (MEXU) were consulted, as well as, electronically, those of the Arizona State University (http://www.swbiodiversity.org), Missouri Botanical Garden (MO; https://tropicos.org), New York Botanical Garden (NY; http://sweetgum.nybg.org/science/vh/), and the Smithsonian Institution (US; https://collections.nmnh.si.edu). To our knowledge, we did not leave out any important source that could contribute to the species list at this time.

Species names were reconciled against Kew’s Plants of the World Online (POWO; http://www.plantsoftheworldonline.org/) which was also used as reference for families and genera classification. The POWO reconciliation was run on 19 March 2019 for the vast majority of the species (99%). For the few species that were not found in POWO we followed Tropicos (Missouri Botanical Garden, 2020) and Villaseñor (2016) except for two species (*Magnolia yajlachhi* and *Populus primaveralepensis*) described in 2019 (Dominguez-Yescas & Vázquez-García, 2019; Vázquez-García et al., 2019).

### Species information

The endemic status of a species was extracted from the checklist of plant species of Mexico (Villaseñor, 2016) with few exceptions after verifying it with POWO (http://www.plantsoftheworldonline.org/) and Tropicos (Missouri Botanical Garden, 2020).

All available data on the threat status was extracted from The International Union for the Conservation of Nature (IUCN) Red List of Threatened Species™ website (IUCN, 2019). For the analytical purposes of this study, according to the version 3.1 of the IUCN Red List Categories and Criteria (IUCN, 2001) species were considered “threatened” if listed as Critically Endangered (CR), Endangered (EN) or Vulnerable (VU). Threat status at national level was extracted from the “NORMA Oficial Mexicana NOM-059” (Semarnat, 2010). CITES (i.e., Convention on International Trade in Endangered Species) listing information for all plant species of Mexico was extracted from the Checklist of CITES species website (UNEP-WCMC, 2019).

Data of seed collections of Mexican native trees stored at the Fes-I Seed Bank and those duplicated at the RBG Kew’s Millennium Seed Bank (MSB) was collated from the Fes-I seedbank (data updated at 07 September 2019) and Kew’s MSB (data updated at 28 August 2019) databases.

Data of useful tree species is based on bibliography, databases, herbarium specimens, and websites, especially from “The World Agroforestry Centre (ICRAF)”, which collate information from 23 other sites on agroforestry systems, and is available at http://apps.worldagroforestry.org/products/switchboard/index.php/species_search/Acacia/. Furthermore, data was extracted from the “Millennium Seedbank collections” database (latest data download: 28 August 2019) for plant species in our species list. A species was considered “useful” when at least one material (e.g., human food) or non-material (e.g., cultural) use was recorded in any of the sources.

All information was linked using the full name of the taxa to the species list, in order to generate an attribute table representing all data for each species (see Appendix S1). Information linked to subspecific taxa was therefore not included

### Spatial analysis

The majority of the tree species data and their occurrence records were obtained from the database of the National Biodiversity Information System (SNIB) of the National Commission for the Knowledge and Use of Biodiversity (CONABIO, 2017). This data, consisting of voucher information from herbarium specimens (called “georeferenced records” hereafter) was linked to the species list based on the species name, using the package “plyr” (Wickham, 2011) in R 3.3.3 (R Core Team, 2017). Initial data cleaning was performed in ArcMap 10.7 (Esri, Redlands, CA, USA) and R 3.3.3, deleting or fixing wrong coordinates or dubious records: (1) most records reported for Mexico but with coordinates located in other countries or falling into the ocean were deleted, unless it was possible to fix coordinates from descriptions of collection localities (e.g., cases of inverted coordinates, where the Lat. had been indicated as Lon., or cases of Lon. lacking the negative sign); (2) all records with coordinates pointing to the Mexico’s geographic centre (i.e., Lat. 23°N, Lon. 102°W) were deleted; (3) coordinates with a precision larger than 10 km (e.g., with only one decimal degree or without decimals) were manually inspected and deleted if dubious; and (4) all records falling outside the country’s continental boundary (e.g., in the sea) but within 1 km of distance, were moved to the closest area inside the country’s continental boundary. To reduce data redundancy, database normalisation was performed in R 3.3.3, by deleting duplicated records, standardising names and abbreviations of Mexican states, and species attributes. Because of the computational burden, a separate dataset was prepared for each variable.

A spatial grid layer was generated in R 3.3.3 with the package “raster” (Hijmans, 2016), using the Coordinate Reference System as the initial grid. Various grid cell resolutions were explored (i.e., with grid cell sizes of 10 × 10 km, 20 × 20 km, 25 × 25 km, 30 × 30 km and 50 × 50 km), considering computational time and response signal: grids with very fine resolution (e.g., 10 × 10 km) had too many empty cells, poor signal (e.g., very small hot spots difficult to see) and would take several days to compute, while very coarse resolution grids (e.g., 50 × 50 km) would not allow us to identify more precise geographic areas of interest. The overall patterns did not change among grids with different resolutions, therefore we decided to use a standard grid cell size of 25 × 25 km for all the maps. Richness measurements by state (choropleths) and by grid cell were computed with the R packages “rgdal” (Bivand, Keitt & Rowlingson, 2017), “raster”, and “sp” (Bivand, Pebesma & Gomez-Rubio, 2013). Additional shapefiles for the general description of the study area and interpretation of the observed patterns were downloaded from the CONABIO “Geoportal del Sistema Nacional de Información sobre Biodiversidad”: mean annual precipitation (Cuervo-Robayo et al., 2015a), mean annual temperature (Cuervo-Robayo et al., 2015b), municipal, state and private nature reserves (CONANP, 2017), and federal nature reserves (CONABIO, 2015). The natural protected areas were then plotted on a map of species richness of trees, allowing us to identify unprotected areas with high richness.

## Results

### Tree species diversity and endemism

According to our work, Mexico is represented by 2,885 tree species, belonging to 612 genera and 128 families (see Appendix S1). About 51% of the tree species belong to just 10 families, with Fabaceae being the most represented family with 513 species, while the most represented genus is *Quercus* with 133 species (Table 1). Among the most represented taxa, there are “unusual trees” which complied with the tree definition used in this study, as for example columnar cacti (Cactaceae; tenth most represented family) and *Yucca* species (11th most represented genus). When analysing the endemic component (Table 1) almost half of these species (1,264, ca. 44%) are endemic to Mexico. About 60% of the endemic species are found in 10 families, which differ from the top 10 of the overall tree flora by the higher rank of Asparagaceae and the lower rank of Myrtaceae (Table 1), while *Quercus* is the richest genus even among endemic trees (84 species; Table 1).

**Table 1:** Most represented families and genera of tree species of Mexico. Values represent number of species and, in brackets, their percentage with respect to the total.

	**Family (overall)**	**Number of species (%)**	**Family (endemic to Mexico)**	**Number of endemic species (%)**	**Genus (overall)**	**Number of species (%)**	**Genus (endemic to Mexico)**	**Number of species endemic to Mexico (%)**
1	Fabaceae	513 (17.8)	Fabaceae	229 (18.1)	*Quercus*	133 (4.6)	*Quercus*	84 (6.6)
2	Rubiaceae	210 (7.3)	Rubiaceae	91 (7.2)	*Bursera*	85 (2.9)	*Bursera*	75 (5.9)
3	Fagaceae	134 (4.6)	Fagaceae	84 (6.6)	*Lonchocarpus*	79 (2.7)	*Lonchocarpus*	51 (4.03)
4	Lauraceae	131 (4.5)	Burseraceae	76 (6.0)	*Eugenia*	56 (1.9)	*Ocotea*	22 (1.7)
5	Malvaceae	112 (3.9)	Malvaceae	59 (4.7)	*Pinus*	45 (1.6)	*Randia*	21 (1.6)
6	Myrtaceae	92 (3.2)	Lauraceae	57 (4.5)	*Ocotea*	44 (1.5)	*Eugenia*	20 (1.6)
7	Burseraceae	90 (3.1)	Cactaceae	53 (4.2)	*Inga*	35 (1.2)	*Magnolia*	20 (1.6)
8	Euphorbiaceae	74 (2.6)	Euphorbiaceae	39 (3.1)	*Arachnothryx*	32 (1.1)	*Arachnothryx*	19 (1.5)
9	Rosaceae	60 (2.1)	Asparagaceae	37 (2.9)	*Randia*	30 (1.0)	*Jatropha*	16 (1.3)
10	Cactaceae	59 (2.0)	Rosaceae	31 (2.4)	*Coccoloba*	27 (0.9)	*Pinus*	16 (1.3)
11	Salicaceae	57 (2.0)	Rutaceae	26 (2.1)	*Yucca*	27 (0.9)	*Yucca*	16 (1.2)
12	Pinaceae	55 (1.9)	Anacardiaceae	25 (1.9)	*Cordia*	25 (0.9)	*Aiouea*	15 (1.0)
13	Asparagaceae	53 (1.8)	Myrtaceae	24 (1.9)	*Ficus*	25 (0.9)	*Clethra*	13 (1.0)
14	Rutaceae	53 (1.8)	Annonaceae	22 (1.7)	*Magnolia*	25 (0.9)	*Stenocereus*	13 (1.0)
15	Annonaceae	51 (1.8)	Malpighiaceae	22 (1.7)	*Palicourea*	23 (0.8)	*Cephalocereus*	12 (0.9)
16	Moraceae	43 (1.5)	Magnoliaceae	20 (1.6)	*Prunus*	23 (0.8)	*Deppea*	11 (0.9)
17	Sapotaceae	42 (1.5)	Pinaceae	20 (1.6)	*Sideroxylon*	23 (0.8)	*Erythrina*	11 (0.9)
18	Anacardiaceae	41 (1.4)	Salicaceae	16 (1.3)	*Morisonia*	22 (0.7)	*Erythrostemon*	11 (0.9)
19	Arecaceae	41 (1.4)	Arecaceae	14 (1.1)	*Psychotria*	22 (0.7)	*Mimosa*	11 (0.9)
20	Asteraceae Boraginaceae	41 (1.4)	Asteraceae, Boraginaceae, Primulaceae, Rhamnaceae	14 (1.1)	*Aiouea*	21 (0.7)	*Bauhinia Brongniartia, Diospyros, Inga, Esenbeckia, Malpighia, Parathesis, Prunus*	10 (0.8)

### Spatial distribution of richness and endemism

The spatial dataset contained 1,026,559 clean georeferenced records, corresponding to 128 families, 591 genera and 2,723 species (including 74 species with one record and 109 with two records), representing 94% of the total number of reported species. Details on the number of georeferenced records for species endemic to Mexico, threatened according to the IUCN, useful, banked and CITES listed species are reported in Table S1. We could not map spatial distributions for 162 species, either because georeferenced specimens had inconsistencies with the geographic coordinates (e.g., 70 of those species had either coordinates pointing to the geographic centre of the country or falling far outside Mexico’s boundary and were deleted during the cleaning process), or because voucher specimens did not have sufficient information to georeference them accurately.

There is relative consistency between the diversity within the taxa and the number of georeferenced records, so that the richest families (Table 1) rank in the top most collected ones, and at the genus level, *Quercus* is the genus with more georeferenced records, with 12 species of this genus ranking in the top 20 with the highest number of mapped records (Table 2).

**Table 2:** Most represented families, genera and species of trees of Mexico in the spatial dataset. *Taxa* are sorted by number of georeferenced records.

**Family**	**No. genera**	**No. Species**	**No. Records**	**Genera**	**No. Species**	**No. Records**	**Species**	**No. Records**
Fagaceae	2	126	225,881	*Quercus*	125	225,842	*Bursera simaruba*	17,125
Fabaceae	86	483	138,080	*Bursera*	85	61,301	*Quercus laeta*	15,259
Burseraceae	3	90	63,382	*Pinus*	45	59,436	*Quercus magnoliifolia*	14,583
Pinaceae	4	54	63,309	*Lysiloma*	7	18,137	*Quercus arizonica*	13,291
Rubiaceae	46	201	34,040	*Coccoloba*	26	12,423	*Quercus rugosa*	12,200
Malvaceae	32	108	32,149	*Lonchocarpus*	77	10,638	*Quercus sideroxyla*	10,927
Arecaceae	13	33	22,619	*Juniperus*	15	10,134	*Quercus crassifolia*	10,001
Euphorbiaceae	19	70	22,481	*Ficus*	25	9,768	*Quercus resinosa*	9,877
Moraceae	10	42	19,946	*Piscidia*	4	9,216	*Quercus obtusata*	9,489
Polygonaceae	5	36	19,865	*Cordia*	24	9,198	*Quercus castanea*	9,400
Lauraceae	8	123	17,553	*Arbutus*	5	9,143	*Lysiloma latisiliquum*	9,317
Salicaceae	15	55	17,411	*Thrinax*	1	9,109	*Thrinax radiata*	9,109
Anacardiaceae	14	41	16,482	*Guazuma*	1	8,907	*Guazuma ulmifolia*	8,907
Sapindaceae	13	34	16,402	*Croton*	8	7,481	*Piscidia piscipula*	8,834
Cactaceae	13	57	16,254	*Bauhinia*	20	7,373	*Arbutus xalapensis*	7,669
Sapotaceae	5	38	14,959	*Heliocarpus*	9	6,914	*Quercus grisea*	7,609
Boraginaceae	5	39	13,519	*Senna*	14	6,822	*Pinus durangensis*	7,547
Myrtaceae	11	84	13,191	*Eugenia*	51	6,502	*Quercus laurina*	7,109
Ericaceae	5	13	13,104	*Sabal*	6	6,321	*Juniperus deppeana*	6,373
Meliaceae	4	21	12,955	*Mimosa*	16	6,139	*Quercus eduardi*	6,196

**Figure 3:**
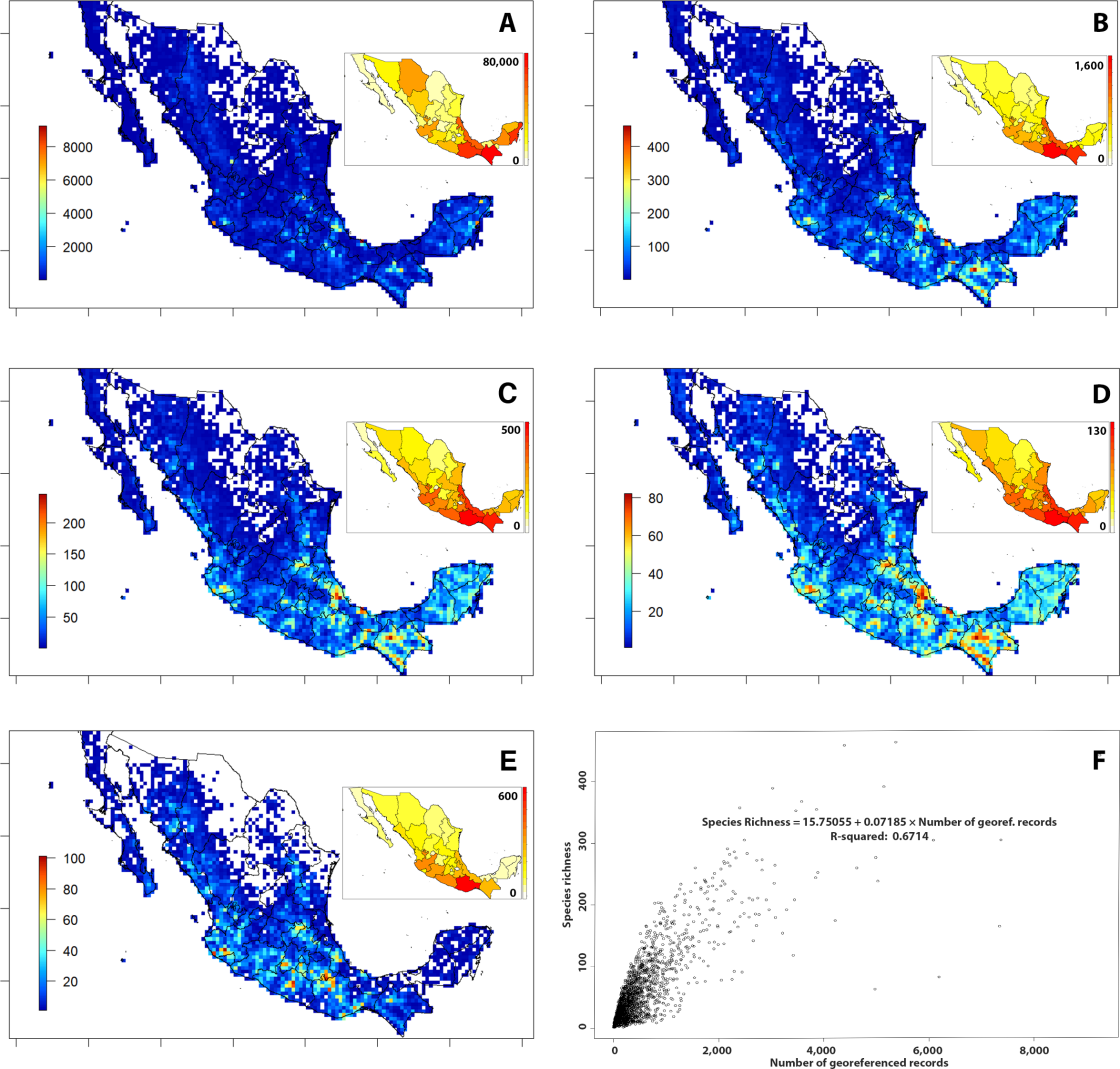
Spatial density and richness of trees from Mexico. (A) Density of records. (B) Species richness. (C) Genus richness. (D) Family richness. (E) Richness of species endemic to Mexico. (F) Correlation plot of georeferenced records versus spatial species richness, including equation of linear regression and R-squared. Bars on the left of maps A–E indicate the colour code for the records/taxa number of each cell. Grid cell size: 25 × 25 km; empty cells show 0-values of mapped records. Smaller maps in A–E show the same statistics calculated by Mexican state.

Georeferenced records of tree species cover most of the country but there are data gaps in the Sonoran and Chihuahuan desert regions in the North-East and North-West, respectively and in the areas corresponding to the southern part of the “Mesa del Norte” and the “Sierra de Órganos” in Durango and Zacatecas states and on the North-East border of San Luis Potosí (Fig. 3A). A higher density of georeferenced records was found in the three main mountain ranges (i.e., “Sierra Madre Occidental”, “Sierra Madre Oriental” and “Sierra Madre del Sur”), the “Eje Volcánico Transversal” and the Yucatán Península, as also highlighted by the analysis by states (Fig. 3A and in-plot map).

When analysing species richness, the southern part of the country showed the highest values, with at least one peak of >400 species per 625 km^2^ cell in the states of Veracruz and Chiapas and values >300 species per cell in Jalisco, Oaxaca, Querétaro and México (Fig. 3B). The same overall pattern was also detected when analysing genus and family richness, although in these two measures values increased towards the northern regions through the two main mountain chains (“Sierra Madre Occidental” and “Sierra Madre Oriental”) and towards the east in the Yucatán Península (Figs. 3C–3D).

The distribution patterns above described for overall species were also detected for the species endemic to Mexico, although they were less evident in the dry areas of the Chihuahua Desert, and in the Yucatán Península (Fig. 3E). Peaks of >80 endemic species per 625 km^2^ cell were identified in Jalisco, Guerrero, Oaxaca, Puebla, Veracruz and Querétaro, (Fig. 3E).

However, results show no clear spatial correlation (R-squared 0.6714) between the species richness and number of georeferenced records (Fig. 3F), implying that the most collected areas are not necessarily the richest ones.

### Useful species

**Figure 4:**
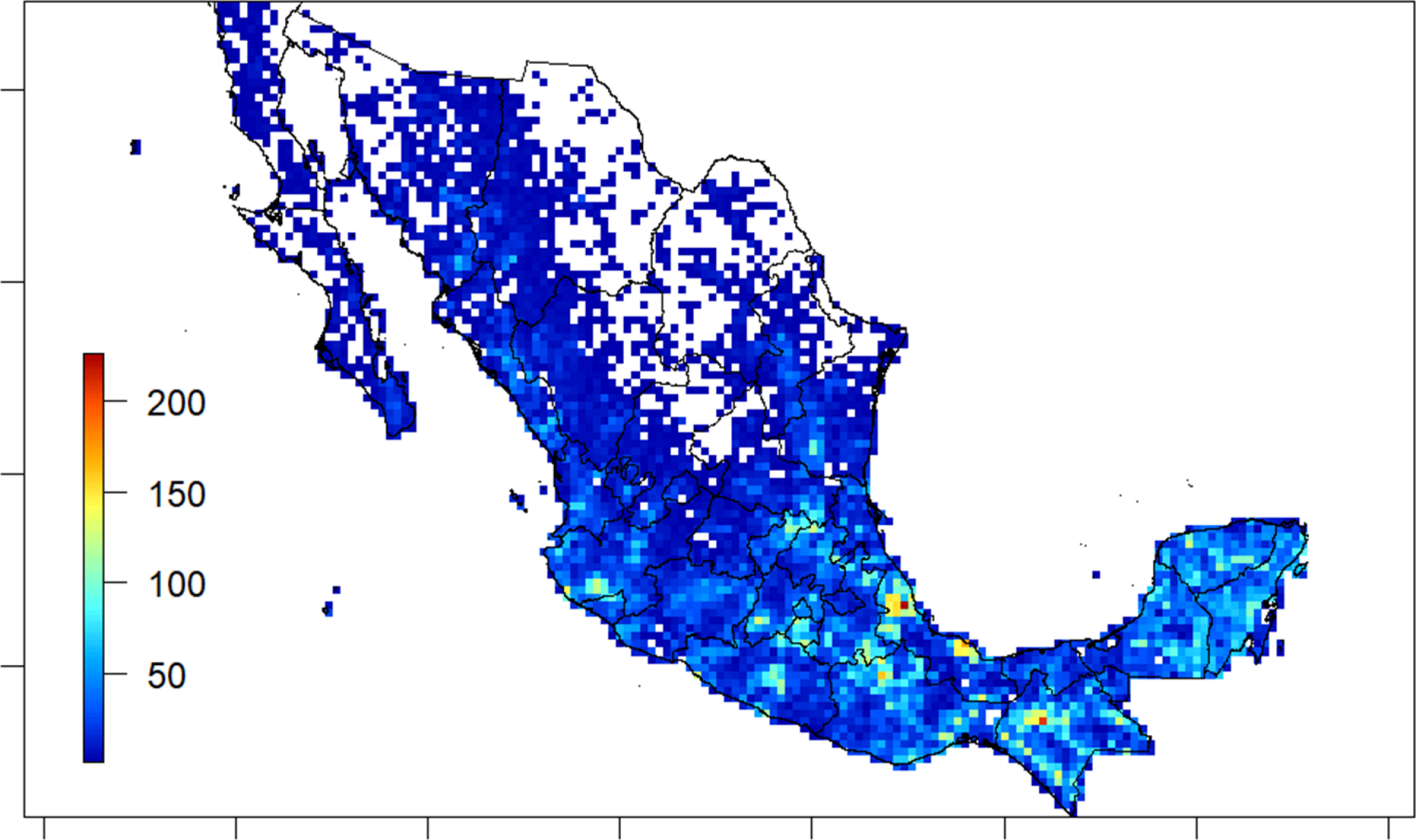
Spatial species richness of useful trees from Mexico. Bar on the left indicates the colour code for the number of species of each cell. Grid cell size: 25 × 25 km; empty cells show 0-values of mapped records.

Almost seven hundred (674) species (ca. 23% of the total) were reported to have at least one material or non-material use and could therefore be identified as “useful plants” for the analysis of this study. Richness of useful trees (Fig. 4) followed the distribution pattern of overall tree species (Fig. 3B), with higher values in the southern region of the country than in the arid regions of the north, and towards north through the two main chains and east to the Yucatán Península, with peaks of species richness (>200 useful species per cell) in Veracruz and Chiapas (Fig. 4).

### Conservation status

Around 33% of the tree species have been assessed so far for conservation status, either at global or at national level. In particular, 920 species (corresponding to 32%) have been assessed only in the IUCN Red List at global level, 29 species (1%) only by the NOM-059 at national level and another 44 species (1.5%) at both levels, leading to a total of 964 IUCN listed species, with 249 species reported as threatened (Table 3), and 73 nationally listed species (Table 3). In addition, 98 species (ca. 3.4%) are listed in the CITES annexes (Table 3).

**Table 3:** Conservation status assessments and listing in international protection catalogues for the tree species of Mexico. Values represent number of species and, in brackets, their percentage respect to the total.

**The IUCN Red List of Threatened Species™(IUCN, 2019)**	**NORMA Oficial Mexicana NOM-059 (Semarnat, 2010)**	**Checklist of CITES species (UNEP-WCMC, 2019)**	
**CR = 12 (0.42)**	*P* = 13 (0.48)	Annex I = 3 (0.10)	
**EN = 113 (3.92)**	*A* = 31 (1.07)	Annex II = 93 (3.20)	
**VU = 124 (4.31)**	Pr = 29 (1.01)	Annex II/NC = 1 (0.03)	
NT = 27 (0.93)		Annex III = 1 (0.03)	
LC = 643 (22.29)			
DD = 21 (0.73)			
LR/cd = 3 (0.10)^*^			
LR/nt = 4 (0.14)^*^			
LR/lc = 17 (0.59)^*^			

**Notes.**

CR Critically Endangered EN Endangered VU Vulnerable NT Not Threatened LC Least Concern DD Data Deficient LR/cd Lower Risk:Conservation Dependent LR/nt Lower Risk LR/lc Lower Risk:Least Concern

In bold the IUCN categories used to define a species as “threatened”.

* IUCN categories according to the criteria version 2.3 (IUCN, 1994).

P, “En peligro de extinción”, i.e., at the brink of extinction; A, “Amenazadas”, i.e., threatened; and Pr, “Sujetas a proteccóin especial”, i.e., to be subjected to special protection.

**Figure 5:**
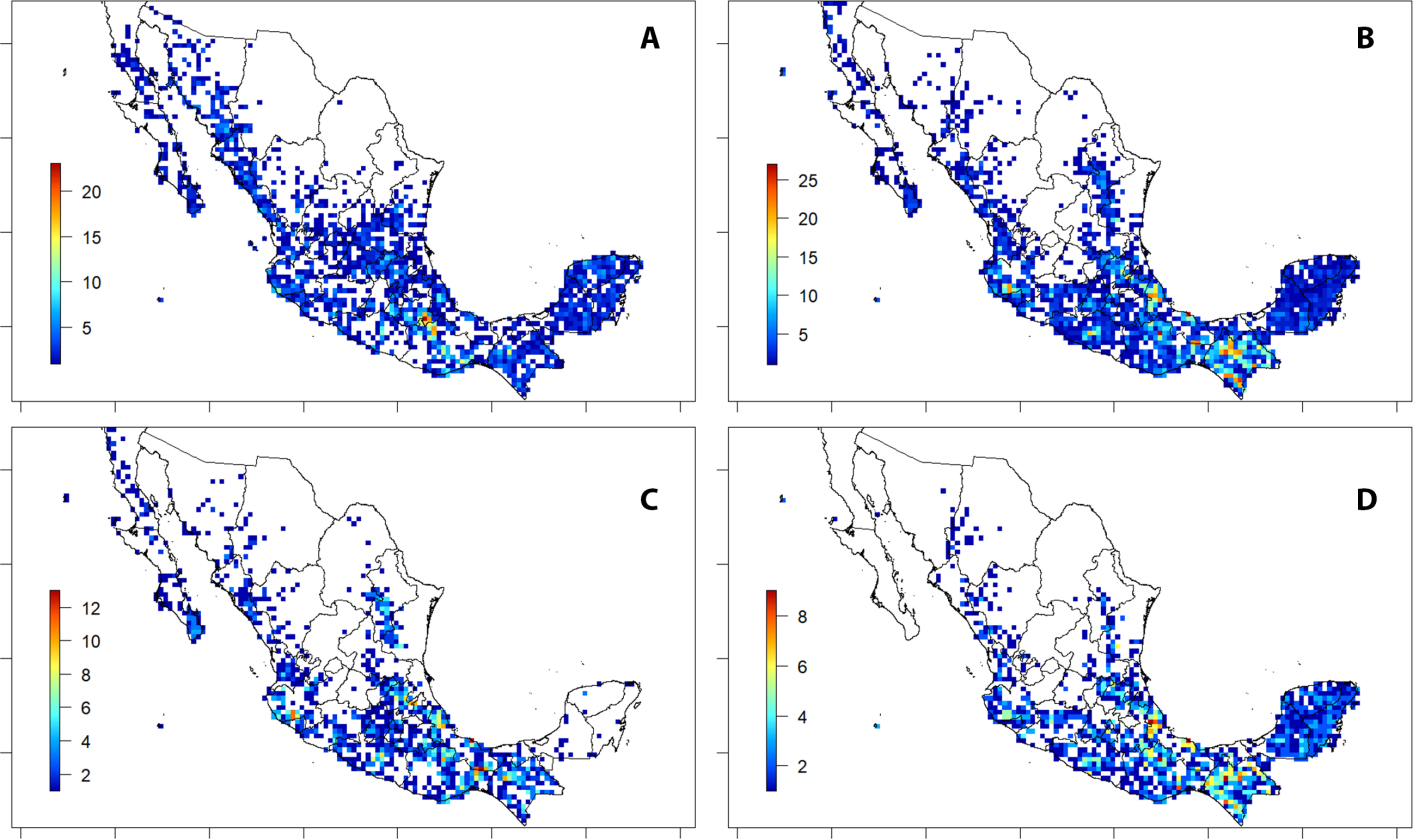
Spatial species richness and conservation risk. (A) Trees listed in CITES I, II or III. (B) Threatened trees (IUCN: VU, EN, or CR). (C) IUCN threatened and endemic to Mexico trees. (D) IUCN threatened and useful trees. Bars indicate the colour code for the species number of each cell. Grid cell size: 25 × 25 km; empty cells show 0-values of mapped records.

The highest richness of CITES listed tree species was detected in the Tehuacán-Cuicatlán Valley (Fig. 5A). Threatened species followed the same distribution pattern as overall species richness (Fig. 3B), with higher values in the three main mountain chains and in the “Eje Volcánico Transversal”. Five main peaks of threatened tree richness (>20 species per cell) were identified among the states of Veracruz, Chiapas, Oaxaca and Jalisco (Fig. 5B). Although no high values of threatened species were identified in the Yucatán Península, the whole area showed a quite homogenous presence of trees of threatened status (Fig. 5B), which were predominantly species non endemic to Mexico (Fig. 5C). Threatened useful trees did not differ in their distribution and richness pattern with respect to the overall threatened species patterns, with peaks in Veracruz and Chiapas (Fig. 5D).

### Conservation actions currently in place

Regarding ex situ conservation through seedbanking, a total of 560 species (19% of the total) have been stored in the Fes-I seed bank of Mexico or Kew’s Millennium Seed Bank in the UK (Table 4).

**Figure 6:**
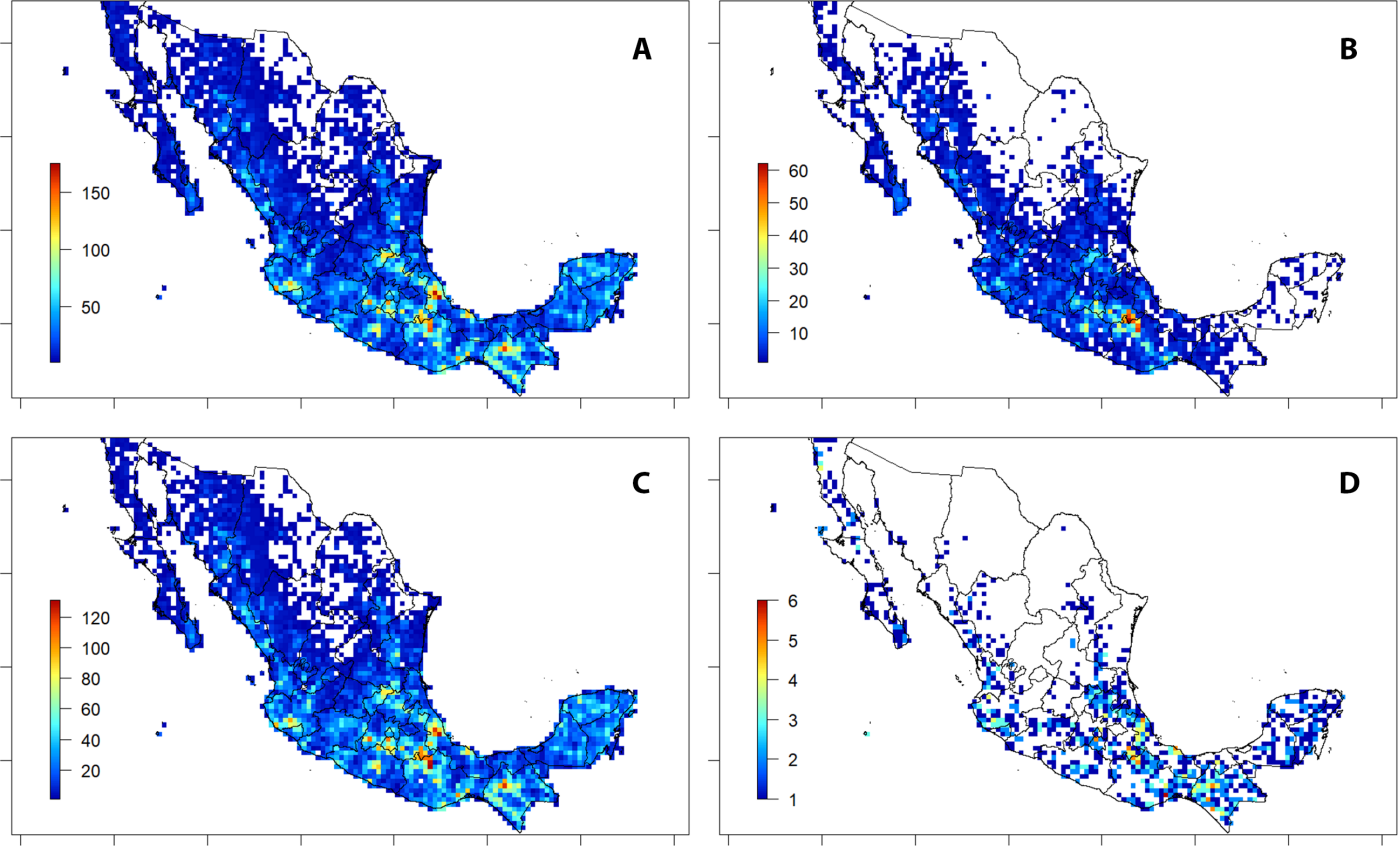
Spatial species richness and seed-banking preservation. (A) Banked species of trees either at the MSB or FES-I. (B) Banked treespecies endemic to Mexico. (C) Banked useful species of trees. (D) Banked threatened species of trees. Bars indicate the colour code for the species number of each cell. Grid cell size: 25 × 25 km; empty cells show 0-values of mapped records.

Seed-banked species followed the distribution pattern previously detected for overall species (Fig. 3B), with three peaks of high density of banked trees (>150 species per cell) in Puebla and Oaxaca (Tehuacán-Cuicatlán Valley), Veracruz and Chiapas (Fig. 6A). The Tehuacán-Cuicatlán Valley was an early priority for ex situ conservation by Fes-I and therefore has high density of banked trees, even when considering trees endemic to Mexico (peak of >60 species per cell) (Fig. 6B). The distribution patterns of banked useful trees (Fig. 6C) followed that of the overall banked species (see Fig. 6A). The spatial analysis of banked threatened trees highlighted how in the Yucatán Península, there is a homogeneous cover of threatened, although non endemic to Mexico, species (see Fig. 5B). However very few species have been banked in this region (Fig. 6D), highlighting a collection gap.

**Table 4:** Tree species banked in country and at Kew’s MSB. Values represent the number of species and, in brackets, their percentage with respect to the total. Threatened species are those listed as CR (Critically Endangered), EN (Endangered) or VU (Vulnerable), according to IUCN (2019).

**Category (Total)**	**Banked in country (Fes-I Seed bank)**	**Banked in country and duplicated in the UK (Kew’s MSB)**	**Total of tree species banked**
Overall (2885)	493 (17.1)	425 (14.7)	560 (19.4)
Endemic to Mexico (1264)	168 (14.1)	144 (11.4)	197 (15.6)
Useful plants (674)	310 (45.7)	2909 (43.0)	341 (50.6)
Threatened species (249)	25 (10.0)	17 (6.8)	25 (10.0)

**Figure 7:**
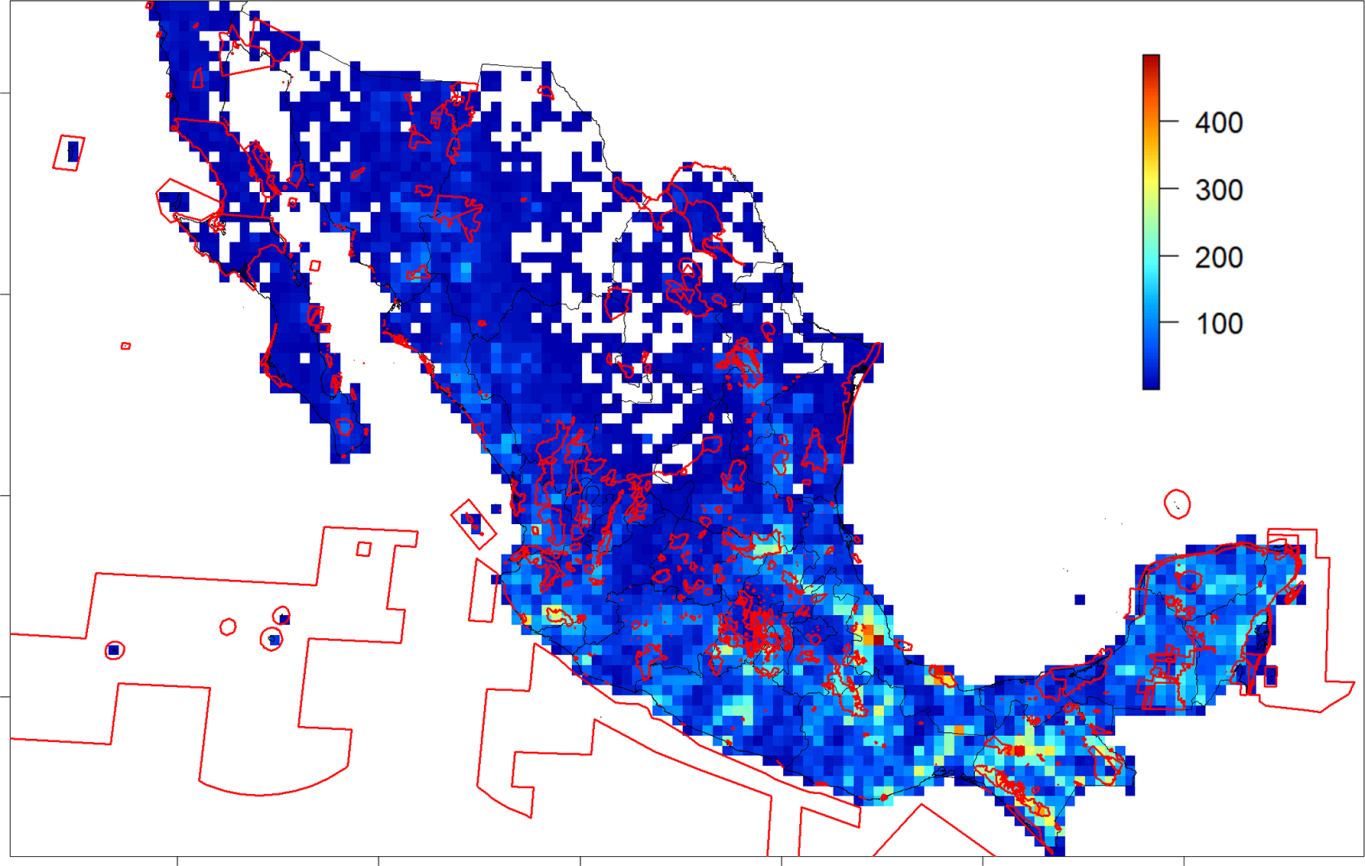
Spatial species richness and in-situ conservation. Purple polygons identify the protected areas; bars indicate the colour code for the species number of each cell. Grid cell size: 25 × 25 km; empty cells show 0-values of mapped records.

When overlapping the species richness layer with all the protected areas, it is possible to see two large and species-rich areas with few or no protected areas: one in Veracruz and the other in Chiapas (Fig. 7).

## Discussion

Generating a comprehensive catalogue for the Mexican native trees has been a national objective for many years, but never fully accomplished, mainly due to an unsatisfactory knowledge of the national flora and the difficulty of synthesizing scattered information (Villaseñor, 2016). A big step forward was made by the publication of the checklist of the native vascular plants of Mexico (Villaseñor, 2016). However, this catalogue did not provide information on the growth form of the listed species, leaving unanswered the question of how many of those were trees. An answer to this question was recently provided by the “GlobalTreeSearch” initiative of the Botanic Gardens Conservation International (BGCI), which documented of the world’s known trees and their country level distributions, including Mexico (Beech et al., 2017). In this paper, we present a comprehensive catalogue of native tree species of Mexico, integrated with analysis of their distribution, conservation status and material and non-material uses at country level. The number of recognized trees presented in this work (2885 species) represents ca. 13% of the whole native vascular flora of Mexico (ca. 23,000 species; Villaseñor, 2016; Ulloa Ulloa et al., 2017), in line with their proportion at global level (ca. 15%; Beech et al., 2017; Willis, 2017).

Fabaceae is the family with the highest number of tree species in Mexico (see Table 1). This is not surprising, considering that, after Asteraceae, it is the second most represented plant family in the country (1,903 species; Villaseñor, 2016) and, globally, the one with the highest number of tree species (5,405 species; Beech et al., 2017). The mountain regions of Mexico represent one of the two global centres of diversity of the genus *Quercus* (Valencia Avalos, 2004), as also confirmed in this work, with *Quercus* being the genus with the highest number of Mexican tree species overall and among tree species (see Table 1). At the same time, *Bursera* is a species-rich genus of woody plants whose distribution is limited to the Mesoamerican and Caribbean regions (Espinosa, Llorente & Morrone, 2006), as also confirmed in this study, where it occupies second place in the number of trees overall and of trees endemic to the country (see Table 1). Considering “unusual trees”, Mexico hosts ca. 60% of all *Yucca* species (Asparagaceae; García-Mendoza & Galván, 1995), as well as the highest species richness and endemism rate of Cactaceae (Hernández & Godínez, 1994).

When considering the whole vascular flora of Mexico, ca. half of the species are endemic to the country (Ulloa Ulloa et al., 2017; Villaseñor, 2016). This high value is reflected by the proportion of endemic species among trees, highlighting their contribution to the overall endemic flora of Mexico.

The spatial analysis carried out in this study for tree species, confirmed the same distribution patterns of the overall vascular flora at species, genus, and family level, with peaks of high tree diversity in the states of Veracruz, Oaxaca, and Chiapas in the South of Mexico and (at species level only) Jalisco state in the western part of the country (Villaseñor, 2016). The highest plant species richness in Mexico is associated with regions of high rainfall and temperatures (Villaseñor & Ortiz, 2014), as previously reported for specific taxonomic groups such as gymnosperms (Contreras-Medina & Luna-Vega, 2007) and grasses (Dávila-Aranda, Lira & Valdés-Reyna, 2004). The distribution pattern detected in this study with a tree diversity gradient from the South to the North of the country at species, genus, and family level, might reflect the contact between the Nearctic and Neotropical biogeographic realms, known as the Mexican Transition Zone (Halffter, 1987; Villaseñor, 2016). However, there are some states of central Mexico breaking from this gradual shift of richness, particularly from the western region formerly known as Nueva Galicia (i.e., Aguascalientes, Colima, Jalisco, Nayarit, and Zacatecas states; McVaugh, 1974; McVaugh, 1983; McVaugh, 1984; McVaugh, 1987; McVaugh, 1992), as confirmed by the peak of species richness identified in this study for trees in Jalisco.

Low levels of tree richness have also been detected for the two main peninsulas (Baja California and Yucatán), as previously reported for the whole vascular flora (Villaseñor, 2016), likely due to a “peninsular effect” (Gaston & Williams, 1996). However, although the taxonomic and floristic knowledge of vascular plants in northern Mexico present relevant advances (González-Elizondo et al., 2017), it still needs to be confirmed if the low diversity detected in the northern regions (Villaseñor, 2016) results from an incomplete taxonomic and floristic knowledge in these states.

Species endemic to the country are widespread throughout Mexico, probably in a differential response to environmental, historical and genetic factors (Rzedowski, 1991). The overall tree species richness correlated with the distribution of trees endemic to the country, with four out of the six peaks of endemic species richness (in Jalisco, Puebla, Veracruz, and Querétaro; Figs. 3B–3E) overlapping with those of overall species richness. These six peaks are all included in the areas of Mexican endemism of red oaks (Torres Miranda, Luna-Vega & Oyama, 2013); excluding the state of Veracruz, in that of the genus *Bursera* (Gámez et al., 2014); and of gymnosperms in the states of Oaxaca and Veracruz (Contreras-Medina & Luna-Vega, 2007).

Nearly 5,000 to 7,000 plant species are reported to have at least one material or non-material use in Mexico (Casas, Viveros & Caballero, 1994). Therefore, trees, with ca. 700 useful species, represent 10–14% of the useful flora of the country, almost in line with their proportion in the whole vascular flora (see section 3.1). Their distribution follows that of the overall tree species. This is not surprising, considering the complex forms of interactions developed between plants and human groups inhabiting the Mexican territory through a rich cultural history >of over 10,000 years (MacNeish, 1992). In addition, the two peaks of useful species richness identified in Veracruz and Oaxaca states (see Fig. 4), correspond to the two areas with the highest diversity of current territories of indigenous people in the country (Sarukhán et al., 2010).

At the global level, ca. 37,000 conservation assessments of vascular plant species have been carried out and published in the IUCN Red List (IUCN, 2019), representing only around 9.5% of all the world’s plants (Willis, 2017). The percentage of the Mexican vascular flora assessed in the IUCN Red List for Mexico (ca. 2,600 species of the ca. 23,000, i.e., ca. 11%) is in line with the figures at global level (IUCN, 2019; Villaseñor, 2016). However, this percentage increases to ca. 33% when considering only native trees. The highest proportion of conservation assessments identified for trees is likely to be due to the strong bias in the Red List towards threatened tree species (Oldfield, Lusty & MacKinven, 1998) and to the recent redlisting efforts of BGCI’s Global Trees Campaign (https://globaltrees.org/threatened-trees/red-list/).

The peak of CITES listed tree species richness found in the Tehuacán-Cuicatlán Valley can be explained considering that this area has the highest concentration of columnar cacti in the world, which has led to its declaration as Biosphere Reserve in 2012 (http://www.unesco.org/new/en/natural-sciences/environment/ecological-sciences/biosphere-reserves/latin-america-and-the-caribbean/mexico/tehuacan-cuicatlan/). In addition, the findings of this study showed that ca. 60% of the Mexican trees listed in CITES are cacti (Appendix S1). Richness of threatened species was similar to that of the trees endemic to Mexico, with peaks coinciding with those of the overall and endemic tree species richness. Geographic range and population size constitute the main criteria to assess a species in IUCN Red List Categories (IUCN, 2001), so it is not surprising that 65% of the threatened tree species are endemic to the country (Appendix S1), i.e., with a relatively narrower distribution compared to the widespread ones.

In order to be effective, in situ and ex situ conservation measures need to complement each other. To date, seeds of ca. 17% of the Mexican trees are stored in *ex situ* facilities for long term conservation under internationally recognized standards (Liu et al., 2018). Although this figure is limited to the seedlots stored in Mexico at the Fes-I UNAM seed bank and duplicated at the RBG Kew’s MSB in the UK, we believe that it represents a good estimate, due to the continuous joint conservation efforts carried out in Mexico by these two institutions in the last 15 years (León-Lobos et al., 2012; Rodríguez-Arévalo et al., 2017). However, there does appear to be a biased geographical focus (see Fig. 6), with efforts previously concentrated in the Tehuacán-Cuicatlán Valley (Rodríguez-Arévalo et al., 2017).

The success of future ex situ conservation efforts might be jeopardized by the seed physiological responses of tree species to desiccation and therefore their ability to be stored under traditional seed banking techniques. Information on the seed biology (storage and germination) of tree species is sparse beyond the main species of interest to commercial forestry and detailed studies of the storage biology of desiccation tolerant tree seeds are uncommon (Pritchard et al., 2014). Therefore, a screening programme of native trees of Mexico, by using the “100-seed test” (Pritchard et al., 2004), could help to assess their seed desiccation responses as was recently conducted for the trees of the Caribbean region (Mattana et al., 2020).

The national system of protected areas covers most of the peaks of tree species richness identified in this study, except for the ones in the states of Veracruz and Chiapas (see Fig. 7), suggesting a potentially satisfactory level of in situ conservation for tree species in Mexico. However, even if the distribution of a species is included within the limits of a protected area, this does not guarantee that the management of that species is in place, due to resource and lack of knowledge. In particular, in many protected areas, people still depend on biodiversity, as well as agricultural and cattle products for their livelihoods, and these activities are not always managed (Pérez-Solano & Mandujano, 2018; Vallejo et al., 2018). However, some protected areas offer ecotourist programmes, with examples of government support for habitat restoration and sustainable management initiatives, including in the project PROCODES 2020 (http://www.gob.mx/conanp/acciones-y-programas/programa-para-el-desarrollo-sostenible-procodes-2020) and as reported in Segura-Warnholtz (2014).

In addition, the role that the system of protected areas can play in the future, due to climate change and its effect on plant distributions, needs to be assessed (Lira, Téllez & Dávila, 2009). The two areas with high richness of tree species identified in the states of Veracruz and Chiapas, which are not currently included in the system of national protected areas, represent two priority areas for tree conservation in Mexico, where integrated in situ and ex situ conservation efforts should be focused and information used to support reforestation and livelihoods programs.

## Conclusions

The catalogue presented in this study constitutes a key milestone towards the better understanding, conservation and management of the Mexican native tree flora. This is relevant not only from a scientific point of view, but also environmentally, economically and socially, considering the significant potential uses that trees have in restoration/reforestation programmes. In addition, it provides pivotal information for planning in situ and ex situ conservation actions focused on trees, their ecosystems and ecosystem services and for the benefits that people derive from trees in terms of livelihoods, food security and human health.

##  Supplemental Information

10.7717/peerj.9898/supp-1 Supplemental Information 1 R Code used for the geographic analyses

10.7717/peerj.9898/supp-2 Table S1 Number of species with georeferenced records and georeferenced records for each category

10.7717/peerj.9898/supp-3 Appendix S1 Catalogue of trees of Mexico with information on endemicity, uses and conservation status Species are listed in alphabetical order of family, genus and species, respectively. Separated by semicolons is reported for each species: information on distribution by states (when available from Villasenor, 2016) and endemicity according to Villasenor (2016) with few exceptions after verifying it with POWO (http://www.plantsoftheworldonline.org/) and Tropicos (Missouri Botanical Garden, 2020); the risk category of the species if listed in “The IUCN Red List of Threatened Species™” (IUCN, 2019); the risk category of the species if listed in the “NORMA Oficial Mexicana (NOM-059)” (Semarnat, 2010); the Cites category from the “Checklist of CITES species” (UNEP, 2015); if the species has been banked (either at FES and/or MSB); and if the species is known as ‘useful’, according to the definitions in the manuscript. P = “En peligro de extinción”, i.e. at the brink of extinction; A = “Amenazadas”, i.e. threatened; and Pr = “Sujetas a protección especial”, i.e. to be subjected to special protection.

## Additional Information and Declarations

### Data Availability

The following information was supplied regarding data availability: The catalogue of trees of Mexico with information on endemicity, uses and conservation status and the R code that was used to analyse the data are available as Supplementary Files. The spatial data were collected from CONABIO and are available at Figshare: Tellez, Oswaldo; Mattana, Efisio; Diazgranados, Mauricio; Kühn, Nicola; Castillo-Lorenzo, Elena; Lira, Rafael; et al. (2020): Native trees of Mexico: diversity, distribution, uses and conservation (datasets used for spatial analyses). figshare. Dataset. https://doi.org/10.6084/m9.figshare.12745931.v1.
