# Supplementary material for: Native trees of Mexico: diversity, distribution, uses and conservation
Source: PeerJ. 2020 Sep 18;8:e9898. doi: 10.7717/peerj.9898 (PMC7505059; doi:10.7717/peerj.9898)
Supplement: Table S1 [file peerj-08-9898-s002.docx]

**Table S1. Number of species with georeferenced records and georeferenced records for each category.**

| **Category** | **Species** | **Georeferenced records** |
| --- | --- | --- |
| Overall | 2,723 | 1,026,559 |
| Endemic trees | 1,200 | 250,011 |
| Useful trees | 651 | 630,369 |
| Threatened trees | 238 | 43,951 |
| Threatened-endemic trees | 156 | 16,356 |
| Threatened-useful trees | 29 | 22,402 |
| Trees banked either at the MSB or FES-I | 541 | 458,446 |
| Trees banked either at the MSB or FES-I endemic | 187 | 74,371 |
| Trees banked either at the MSB or FES-I threatened | 25 | 11,276 |
| Trees banked either at the MSB or FES-I useful | 330 | 376,399 |
| Trees listed in CITES I, II or III | 92 | 25,257 |
