# Supplementary material for: Native trees of Mexico: diversity, distribution, uses and conservation
Source: PeerJ. 2020 Sep 18;8:e9898. doi: 10.7717/peerj.9898 (PMC7505059; doi:10.7717/peerj.9898)
Supplement: Appendix S1 — Species are listed in alphabetical order of family, genus and species, respectively. Separated by semicolons is reported for each species: information on distribution by states (when available from Villasenor, 2016) and endemicity according to Villasenor (2016) with few exceptions after verifying it with POWO (http://www.plantsoftheworldonline.org/) and Tropicos (Missouri Botanical Garden, 2020); the risk category of the species if listed in “The IUCN Red List of Threatened Species™” (IUCN, 2019); the risk category of the species if listed in the “NORMA Oficial Mexicana (NOM-059)” (Semarnat, 2010); the Cites category from the “Checklist of CITES species” (UNEP, 2015); if the species has been banked (either at FES and/or MSB); and if the species is known as ‘useful’, according to the definitions in the manuscript. P = “En peligro de extinción”, i.e. at the brink of extinction; A = “Amenazadas”, i.e. threatened; and Pr = “Sujetas a protección especial”, i.e. to be subjected to special protection. [file peerj-08-9898-s003.docx]

**Native trees of Mexico: diversity, distribution, uses and conservation**

**Supplementary Information**

**Appendix S1**. Catalogue of native trees of Mexico with information on endemicity, uses and conservation status.

**Appendix S1**. Catalogue of trees of Mexico with information on endemicity, uses and conservation status.

Species are listed in alphabetical order of family, genus and species, respectively. Separated by semicolons is reported for each species: information on distribution by states (when available from Villaseñor, 2016) and endemicity according to Villaseñor (2016) with few exceptions after verifying it with POWO (<http://www.plantsoftheworldonline.org/>) and Tropicos (Missouri Botanical Garden. 2020); the risk category of the species if listed in “The IUCN Red List of Threatened Species^TM^” (IUCN, 2019); the risk category of the species if listed in the “NORMA Oficial Mexicana (NOM-059)” (Semarnat, 2010); the Cites category from the “Checklist of CITES species” (UNEP, 2015); if the species has been banked (either at FES and/or MSB); and if the species is known as ‘useful’, according to the definitions in the manuscript. P = “En peligro de extinción”, i.e. at the brink of extinction; A = “Amenazadas”, i.e. threatened; and Pr = “Sujetas a protección especial”, i.e. to be subjected to special protection.

**Cited references**

IUCN. The IUCN Red List of Threatened Species. www.iucnredlist.org (2019).

Semarnat. Norma Oficial Mexicana NOM-059-SEMARNAT-2010. Protección ambiental-especies nativas de México de flora y fauna silvestres - Categorías de riesgo y especificaciones para su inclusión, exclusión o cambio - Lista de especies en riesgo. (Diario Oficial, 2010).

Missouri Botanical Garden. 2020. Tropicos.org. Missouri Botanical Garden http://www.tropicos.org (Accessed 16 January 2020).

UNEP-WCMC. The Checklist of CITES Species Website. http://checklist.cites.org (2015).

Villaseñor, J.L. Checklist of the native vascular plants of Mexico. Rev. Mex. Biodivers. 87, 559-902 (2016).

| **Acanthaceae** |
| --- |
| ***Avicennia*** |
| *Avicennia* *germinans* (L.) L.; BCN, BCS, CAM, CHIS, COL, GRO, JAL, MICH, NAY, OAX, QROO, SLP, SIN, SON, TAB, TAMS, VER, YUC; Red List: LC; NOM-59: A; banked. |
| ***Bravaisia*** |
| *Bravaisia* *integerrima* (Spreng.) Standl.; CAM, CHIS, COL, GRO, JAL, MEX, MICH, OAX, QROO, TAB, TAMS, VER, YUC; NOM-59: A; useful (MEDICINES, ENVIRONMENTAL USES). |
|  |
| **Achariaceae** |
| ***Chiangiodendron*** |
| *Chiangiodendron* *mexicanum* Wendt; CHIS, TAB, VER (endemic); Red List: EN. |
| ***Lindackeria*** |
| *Lindackeria laurina* C.Presl; CHIS; Red List: LC. |
| ***Mayna*** |
| *Mayna odorata* Aubl.; VER; Red List: LC. |
|  |
| **Achatocarpaceae** |
| ***Achatocarpus*** |
| *Achatocarpus* *gracilis* H.Walter; COL, GRO, JAL, MICH, NAY, OAX, SIN, SON. |
| *Achatocarpus* *nigricans* Triana; CAM, CHIS, GRO, JAL, MICH, OAX, QRO, QROO, SLP, TAB, TAMS, VER, YUC; Red List: LC; useful (FOOD, ENVIRONMENTAL USES). |
| *Achatocarpus* *oaxacanus* Standl.; CHIS, GRO, MICH, OAX (endemic). |
|  |
| **Actinidiaceae** |
| ***Saurauia*** |
| *Saurauia angustifolia* Turcz.; CHIS, OAX. |
| *Saurauia aspera* Turcz.; CHIS, HGO, OAX, QRO, SLP, TAB, TAMS, VER. |
| *Saurauia comitis-rossei* R.E.Schult.; (endemic). |
| *Saurauia conzattii* Buscal.; OAX. |
| *Saurauia kegeliana* Schltdl.; CHIS. |
| *Saurauia* *leucocarpa* Schltdl.; CHIS, GRO, HGO, MICH, OAX, PUE, SLP, TAB, VER; Red List: VU. |
| *Saurauia* *madrensis* B.T.Keller & Breedlove; CHIS (endemic). |
| *Saurauia* *montana* Seem. |
| *Saurauia* *oreophila* Hemsl.; CHIS, OAX, VER; Red List: VU. |
| *Saurauia* *pringlei* Rose; CHIS, GRO, MICH, OAX, SLP, VER. |
| *Saurauia* *pustulata* G.E.Hunter; CHIS, TAB (endemic); Red List: LR/lc. |
| *Saurauia* *radlkoferi* Buscal.; PUE, VER (endemic). |
| *Saurauia* *rubiformis* Vatke; CHIS, TAB. |
| *Saurauia scabrida* Hemsl.; CHIS, GRO, HGO, MEX, OAX, PUE, QRO, SLP, TAB, VER; Red List: LR/lc; banked; useful. |
| *Saurauia serrata* DC.; AGS, CHIS, COL, DGO, GRO, JAL, MEX, MICH, MOR, NAY, OAX, PUE, SLP, SIN, TAB, VER (endemic); Red List: EN; NOM-59: Pr. |
| *Saurauia villosa* DC.; CHIS, OAX, PUE, TAB, VER; Red List: VU. |
| *Saurauia yasicae* Loes.; CHIS, HGO, MEX, OAX, PUE, QRO, SLP, TAB, VER; Red List: LC. |
|  |
| **Altingiaceae** |
| ***Liquidambar*** |
| *Liquidambar styraciflua* L.; CHIS, CDMX, GRO, HGO, MEX, MICH, MOR, NLE, OAX, PUE, QRO, SLP, TAB, TAMS, VER; Red List: LR/lc; banked. |
|  |
| **Anacardiaceae** |
| ***Actinocheita*** |
| *Actinocheita filicina* (DC.) F.A.Barkley; GRO, MEX, MOR, OAX, PUE (endemic); banked. |
| ***Amphipterygium*** |
| *Amphipterygium adstringens* (Schltdl.) Schiede ex Standl.; AGS, COL, DGO, GRO, JAL, MEX, MICH, MOR, NAY, OAX, PUE, SLP, VER, ZAC (endemic); banked; useful (MEDICINES). |
| *Amphipterygium glaucum* (Hemsl. & Rose) Hemsl. & Rose ex Standl.; COL, GRO, JAL, MICH, NAY (endemic). |
| *Amphipterygium molle* (Hemsl.) Hemsl. & Rose ex Standl.; AGS, DGO, JAL, NAY, ZAC (endemic). |
| *Amphipterygium simplicifolium* (Standl.) Cuevas-Figueroa; OAX. |
| ***Astronium*** |
| *Astronium graveolens* Jacq.; CAM, CHIS, COL, GRO, HGO, JAL, MICH, NAY, OAX, QRO, QROO, TAB, VER, YUC; NOM-59: A; banked; useful (MATERIALS). |
| ***Attilaea*** |
| *Attilaea abalak* E.Martínez & Ramos; CAM, QROO, YUC. |
| ***Comocladia*** |
| *Comocladia guatemalensis* Donn.Sm.; CHIS. |
| *Comocladia macrophylla* (Hook. & Arn.) L.Riley; GRO (endemic). |
| *Comocladia mollissima* Kunth; CHIS, COL, GRO, JAL, MEX, MICH, MOR, NAY, OAX, PUE, SLP, SIN, TAB, TAMS, VER (endemic); useful (MATERIALS). |
| *Comocladia palmeri* Rose; COL, GRO, JAL, OAX (endemic). |
| *Comocladia repanda* S.F.Blake; GRO, OAX (endemic). |
| ***Cyrtocarpa*** |
| *Cyrtocarpa edulis* (Brandegee) Standl.; BCS, SON (endemic); banked; useful (MEDICINES). |
| *Cyrtocarpa kruseana* R.M.Fonseca; CHIS, COL, GRO, JAL, NAY (endemic). |
| *Cyrtocarpa procera* Kunth; BCS, CHIS, COL, DGO, GRO, JAL, MEX, MICH, MOR, NAY, OAX, PUE, TAMS, ZAC (endemic); banked; useful (FOOD, ANIMAL FOOD, MEDICINES, MATERIALS, FUELS, SOCIAL USES). |
| ***Metopium*** |
| *Metopium brownei* (Jacq.) Urb.; CAM, CHIS, QROO, TAB, VER, YUC; Red List: LC; useful (ANIMAL FOOD, MEDICINES, POISONS, MATERIALS). |
| ***Mosquitoxylum*** |
| *Mosquitoxylum jamaicense* Krug & Urb.; CAM, CHIS, OAX, QROO, TAB, VER; Red List: LC; useful (FOOD). |
| ***Pachycormus*** |
| *Pachycormus discolor* (Benth.) Coville ex Standl.; BCN, BCS (endemic). |
| ***Pistacia*** |
| *Pistacia mexicana* Kunth; AGS, CHIS, CHIH, COAH, COL, GTO, GRO, HGO, JAL, MEX, MICH, NLE, OAX, PUE, QRO, SLP, TAMS, VER, ZAC; Red List: NT; banked; useful (FOOD, MEDICINES). |
| ***Pseudosmodingium*** |
| *Pseudosmodingium* *andrieuxii* (Baill.) Engl.; GRO, HGO, MEX, MOR, OAX, PUE, QRO, SLP, VER (endemic); banked. |
| *Pseudosmodingium* *barkleyi* Miranda; GRO (endemic). |
| *Pseudosmodingium* *perniciosum* (Kunth) Engl.; COL, CDMX, GRO, JAL, MEX, MICH, MOR, NAY, OAX, PUE, QRO, SIN, TAMS (endemic); banked; useful (MEDICINES, POISONS). |
| *Pseudosmodingium* *rhoifolium* (DC.) F.A.Barkley; ND (endemic). |
| *Pseudosmodingium* *virletii* Engl.; GTO, HGO, MEX, QRO, SLP (endemic); banked; useful. |
| ***Rhus*** |
| *Rhus* *andrieuxii* Engl.; COAH, HGO, NLE, OAX, PUE, QRO, SLP, VER, ZAC (endemic). |
| *Rhus* *duckerae* F.A.Barkley; OAX (endemic). |
| *Rhus* *glabra* L.; CHIH, SON; Red List: LC; useful (ENVIRONMENTAL USES). |
| *Rhus* *hartmanii* F.A.Barkley; CHIH, SON (endemic). |
| *Rhus* *integrifolia* (Nutt.) Benth. & Hook.f. ex W.H.Brewer & S.Watson; BCN, BCS, SON; banked. |
| *Rhus* *jaliscana* Standl.; JAL, ZAC (endemic). |
| *Rhus* *kearneyi* F.A.Barkley; BCN, BCS, SON. |
| *Rhus* *lanceolata* (A.Gray) Britton; CHIH, COAH, NLE, SLP, TAMS. |
| *Rhus* *muelleri* Standl. & F.A.Barkley; COAH, NLE (endemic). |
| *Rhus* *oaxacana* Loes.; GRO, OAX (endemic); banked. |
| *Rhus* *ovata* S.Watson; BCN, BCS, CHIH, SON; banked. |
| *Rhus* *schiedeana* Schltdl.; BCN, BCS, CHIS, COAH, DGO, GTO, GRO, HGO, JAL, MEX, NAY, OAX, PUE, QRO, SLP, VER, ZAC; useful (MEDICINES, MATERIALS). |
| *Rhus* *standleyi* F.A.Barkley; CHIS, CDMX, GRO, HGO, MEX, OAX, PUE, QRO, TLAX, VER (endemic); banked; useful (FUELS). |
| ***Spondias*** |
| *Spondias* *mombin* L.; BCS, CAM, CHIS, COL, GTO, GRO, HGO, JAL, MEX, MICH, MOR, NAY, OAX, PUE, QRO, QROO, SLP, SIN, TAB, TAMS, VER, YUC; Red List: LC; useful (FOOD, MEDICINES, MATERIALS). |
| *Spondias* *purpurea* L.; CAM, CHIS, CHIH, COL, DGO, GRO, HGO, JAL, MEX, MICH, MOR, NAY, OAX, PUE, QRO, QROO, SLP, SIN, SON, TAB, TAMS, VER, YUC, ZAC; banked; useful (FOOD, MEDICINES, MATERIALS, ENVIRONMENTAL USES). |
| ***Tapirira*** |
| *Tapirira* *chimalapana* T.Wendt & J.D.Mitch.; OAX, VER (endemic); Red List: VU. |
| *Tapirira mexicana* Marchand; CHIS, OAX, PUE, VER; banked; useful (FOOD, MATERIALS, FUELS). |
|  |
| **Annonaceae** |
| ***Anaxagorea*** |
| *Anaxagorea* *guatemalensis* Standl.; OAX, VER; Red List: LC. |
| ***Annona*** |
| *Annona* *cherimola* Mill.; Red List: LC; banked; useful (FOOD, ANIMAL FOOD, MEDICINES, FUELS, ENVIRONMENTAL USES). |
| *Annona* *contrerasii* J.Jiménez Ram. & J.C.Soto; GRO, MICH (endemic). |
| *Annona* *glabra* L.; CAM, CHIS, COL, GRO, JAL, NAY, OAX, QROO, TAB, TAMS, VER, YUC; useful (FOOD, MEDICINES, MATERIALS, ENVIRONMENTAL USES). |
| *Annona* *globiflora* Schltdl.; CHIS, GTO, HGO, MEX, QRO, QROO, SLP, TAMS, VER (endemic). |
| *Annona* *liebmanniana* Baill.; CHIS, OAX, VER. |
| *Annona* *longiflora* S.Watson; COL, GRO, JAL, MEX, MICH, MOR, NAY, OAX, PUE, VER, ZAC (endemic). |
| *Annona* *longipes* Saff.; VER (endemic). |
| *Annona* *macroprophyllata* Donn.Sm.; CHIS, VER; Red List: LC; banked. |
| *Annona* *mucosa* Jacq.; CHIS, OAX, PUE, TAB, VER; banked; useful (FOOD, ANIMAL FOOD, MEDICINES). |
| *Annona* *muricata* L.; Red List: LC; useful (FOOD, ANIMAL FOOD, MEDICINES, FUELS, ENVIRONMENTAL USES). |
| *Annona* *palmeri* Saff.; COL, GRO, JAL, MICH, OAX, VER. |
| *Annona* *purpurea* Moc. & Sessé ex Dunal; CHIS, COL, JAL, MICH, OAX, PUE, QROO, TAB, VER, YUC; Red List: LC; useful (FOOD, ANIMAL FOOD). |
| *Annona* *rensoniana* (Standl.) H.Rainer; CHIS, OAX, PUE, TAB, VER; Red List: LC. |
| *Annona* *reticulata* L.; CAM, CHIS, COL, DGO, GRO, HGO, JAL, MEX, MICH, MOR, NAY, OAX, PUE, QRO, QROO, SLP, SIN, TAB, TAMS, VER, YUC; banked; useful (FOOD, ANIMAL FOOD, MEDICINES, FUELS, ENVIRONMENTAL USES). |
| *Annona* *scleroderma* Saff.; CHIS; useful (ENVIRONMENTAL USES). |
| *Annona* *spraguei* Saff.; CHIS; Red List: VU; useful (ENVIRONMENTAL USES). |
| *Annona* *squamosa* L.; CAM, CHIS, COL, GRO, JAL, MEX, MICH, MOR, OAX, PUE, QRO, QROO, SLP, SIN, SON, TAB, VER, YUC, ZAC; Red List: LC; useful (FOOD, ANIMAL FOOD, MEDICINES, MATERIALS, ENVIRONMENTAL USES). |
| ***Cymbopetalum*** |
| *Cymbopetalum baillonii* R.E.Fr.; CHIS, MEX, OAX, PUE, TAB, VER (endemic); Red List: VU; banked. |
| *Cymbopetalum gracile* R.E.Fr.; GRO, OAX, VER (endemic). |
| *Cymbopetalum hintonii* Lundell; JAL, MICH, NAY (endemic). |
| *Cymbopetalum mayanum* Lundell; CAM, CHIS, QROO (endemic); Red List: EN. |
| *Cymbopetalum mirabile* R.E.Fr.; CHIS, VER. |
| *Cymbopetalum penduliflorum* (Dunal) Baill.; CAM, CHIS, GRO, JAL, MICH, NAY, OAX, PUE, TAB, VER. |
| *Cymbopetalum stenophyllum* Donn.Sm.; CHIS, GRO, OAX. |
| ***Desmopsis*** |
| *Desmopsis* *erythrocarpa* Lundell; CHIS (endemic). |
| *Desmopsis* *guerrerensis* Gonz.-Martínez & J.Jiménez Ram.; GRO (endemic). |
| *Desmopsis* *lanceolata* Lundell; CHIS, OAX (endemic). |
| *Desmopsis mexicana* R.E.Fr.; MICH (endemic). |
| *Desmopsis* *schippii* Standl.; CHIS; Red List: LC. |
| *Desmopsis* *trunciflora* (Schltdl. & Cham.) G.E.Schatz ex Maas, E.A.Mennega & Westra; CHIS, COL, JAL, NAY, OAX, TAB, VER (endemic). |
| *Desmopsis* *uxpanapensis* G.E.Schatz; OAX, VER (endemic). |
| ***Guatteria*** |
| *Guatteria* *amplifolia* Triana & Planch.; CHIS, GRO, OAX, PUE, TAB, VER; Red List: LC. |
| *Guatteria* *dolichopoda* Donn.Sm.; CHIS; Red List: LC. |
| *Guatteria* *galeottiana* Baill.; CAM, OAX, VER (endemic). |
| *Guatteria* *grandiflora* Donn.Sm.; CHIS, GRO, OAX, TAB, VER; NOM-59: A. |
| ***Mosannona*** |
| *Mosannona* *depressa* (Baill.) Chatrou; CAM, CHIS, GRO, JAL, MICH, OAX, PUE, QROO, TAB, VER, YUC; Red List: LC. |
| ***Oxandra*** |
| *Oxandra* *maya* Miranda; CHIS, TAB. |
| ***Sapranthus*** |
| *Sapranthus campechianus* (Kunth) Standl.; CAM, CHIS, GRO, QROO, TAB, VER, YUC; Red List: LC. |
| *Sapranthus microcarpus* (Donn.Sm.) R.E.Fr.; CHIS, COL, GRO, JAL, MOR, OAX, SIN, VER; Red List: LC. |
| *Sapranthus chiapensis* Standl. ex G.E. Schatz ; CHIS, OAX (endemic). |
| *Sapranthus violaceus* (Dunal) Saff.; COL, GRO, JAL, NAY, OAX, SIN, VER; Red List: LC. |
| ***Stenanona*** |
| *Stenanona flagelliflora* T.Wendt & G.E.Schatz; OAX, VER (endemic). |
| *Stenanona humilis* (Miranda) G.E.Schatz ex Maas, E.A.Mennega & Westra; CHIS, OAX, VER (endemic). |
| *Stenanona migueliana* Ortíz-Rodr. & G.E.Schatz; CHIS (endemic). |
| *Stenanona stenopetala* (Donn.Sm.) G.E.Schatz ex Maas, E.A.Mennega & Westra; CHIS, TAB. |
| ***Tridimeris*** |
| *Tridimeris* *hahniana* Baill.; OAX, QRO, SLP, VER (endemic). |
| ***Unonopsis*** |
| *Unonopsis mexicana* Maas & Westra; OAX (endemic). |
| *Unonopsis* *pittieri* Saff.; CHIS; Red List: LC; useful (ENVIRONMENTAL USES). |
| ***Uvaria*** |
| *Uvaria* *grandiflora* Roxb. ex Hornem.; ND (endemic). |
| ***Xylopia*** |
| *Xylopia* *frutescens* Aubl.; CAM, CHIS, COL, OAX, QROO, TAB, VER. |
|  |
| **Apocynaceae** |
| ***Alstonia*** |
| *Alstonia* *longifolia* (A.DC.) Pichon; CHIS, COL, GRO, HGO, JAL, MEX, MICH, MOR, NAY, OAX, PUE, VER. |
| ***Aspidosperma*** |
| *Aspidosperma* *desmanthum* Benth. ex Müll.Arg.; CAM, CHIS, QROO, TAB, VER; Red List: LC; useful (ENVIRONMENTAL USES, MEDICINES, MATERIALS). |
| *Aspidosperma* *megalocarpon* Müll.Arg.; CAM, CHIS, GRO, OAX, QROO, TAB, VER, YUC; Red List: LR/nt; useful (MATERIALS). |
| ***Cascabela*** |
| *Cascabela* *balsaensis* L.O.Alvarado & J.C.Soto; GRO, MICH (endemic). |
| *Cascabela* *ovata* (Cav.) Lippold; CAM, CHIS, COL, DGO, GRO, JAL, MEX, MICH, MOR, NAY, OAX, PUE, QROO, SIN, TAB, VER, ZAC; useful (POISONS, FUELS). |
| *Cascabela* *pinifolia* (Standl. & Steyerm.) L.O.Alvarado & Ochot.-Booth; CAM, GRO, MEX, MICH, PUE, QROO, YUC. |
| *Cascabela* *thevetia* (L.) Lippold; BCS, CAM, CHIS, COL, GTO, GRO, HGO, JAL, MEX, MICH, MOR, NAY, NLE, OAX, PUE, QRO, QROO, SLP, SIN, SON, TAB, TAMS, VER, YUC; Red List: LC; banked; useful (MEDICINES, MATERIALS, ENVIRONMENTAL USES). |
| *Cascabela* *thevetioides* (Kunth) Lippold; AGS, CAM, CHIS, CHIH, CDMX, GTO, GRO, HGO, JAL, MEX, MICH, MOR, OAX, PUE, QRO, QROO, SLP, SIN, TAB, TAMS, VER, ZAC (endemic); banked; useful (POISONS, MATERIALS). |
| ***Plumeria*** |
| *Plumeria* *obtusa* L.; AGS, CAM, CHIS, CHIH, COL, HGO, JAL, MICH, NAY, QRO, QROO, SLP, SON, VER, YUC, ZAC; useful (MEDICINES, ENVIRONMENTAL USES). |
| *Plumeria* *rubra* L.; AGS, BCN, BCS, CAM, CHIS, CHIH, COL, CDMX, DGO, GTO, GRO, HGO, JAL, MEX, MICH, MOR, NAY, NLE, OAX, PUE, QRO, QROO, SLP, SIN, SON, TAB, TAMS, VER, YUC, ZAC; Red List: LC; banked; useful (FOOD, MEDICINES, POISONS, MATERIALS, ENVIRONMENTAL USES). |
| ***Tabernaemontana*** |
| *Tabernaemontana alba* Mill.; CAM, CHIS, COL, HGO, JAL, MEX, MICH, NAY, OAX, PUE, QRO, QROO, SLP, SIN, TAB, TAMS, VER, YUC; Red List: LC; banked; useful (MEDICINES, POISONS). |
| *Tabernaemontana amygdalifolia* Jacq.; CAM, CHIS, COL, DGO, GRO, JAL, MICH, NAY, OAX, PUE, QRO, QROO, SLP, SIN, VER, YUC, ZAC; Red List: LC; useful (MEDICINES). |
| *Tabernaemontana arborea* Rose; CHIS, OAX, TAB, VER; Red List: LC. |
| *Tabernaemontana donnell-smithii* Rose; CAM, CHIS, COL, DGO, GRO, HGO, JAL, MEX, MICH, NAY, OAX, PUE, QRO, QROO, SLP, SIN, TAB, TAMS, VER, YUC; Red List: LC; banked. |
| *Tabernaemontana eubracteata* (Woodson) A.O.Simões & M.E.Endress; CHIS, OAX, VER. |
| *Tabernaemontana glabra* (Benth.) A.O.Simões & M.E.Endress; CHIS, CHIH, COL, DGO, GRO, JAL, MEX, MICH, MOR, NAY, OAX, PUE, SLP, VER. |
| *Tabernaemontana hannae* (M.Méndez & J.F.Morales) A.O.Simões & M.E.Endress; CHIS, OAX. |
| *Tabernaemontana litoralis* Kunth; BCS, CAM, CHIS, GRO, HGO, JAL, MEX, MICH, MOR, NAY, OAX, PUE, QROO, TAB, VER, YUC; banked; useful. |
| *Tabernaemontana oaxacana* (L.O.Alvarado) A.O.Simões & M.E.Endress; OAX (endemic). |
| *Tabernaemontana stenoptera* (Leeuwenb.) A.O.Simões & M.E.Endress; COL, JAL (endemic). |
| *Tabernaemontana tomentosa* (Greenm.) A.O.Simões & M.E.Endress; BCS, CHIS, CHIH, COL, DGO, GRO, HGO, JAL, MEX, MICH, MOR, NAY, OAX, PUE, SIN, SON, VER, ZAC; banked. |
| *Tabernaemontana venusta* (J.F.Morales) A.O.Simões & M.E.Endress; OAX (endemic). |
| ***Thevetia*** |
| *Thevetia ahouai* (L.) A.DC.; BCS, CAM, CHIS, CDMX, GTO, GRO, MOR, NAY, OAX, PUE, QROO, SIN, SON, TAB, TAMS, VER, YUC; banked; useful (FOOD, MEDICINES). |
| ***Vallesia*** |
| *Vallesia conzattii* Standl.; OAX (endemic). |
| *Vallesia glabra* (Cav.) Link; BCN, BCS, CAM, CHIS, CHIH, CDMX, GTO, GRO, HGO, JAL, MICH, MOR, NAY, OAX, PUE, QRO, QROO, SLP, SIN, SON, TAB, TAMS, VER, YUC; Red List: LC; banked; useful (FOOD, ANIMAL FOOD, MEDICINES, FUELS, ENVIRONMENTAL USES). |
|  |
| **Aquifoliaceae** |
| ***Ilex*** |
| *Ilex anodonta* Standl. & Steyerm.; CHIS. |
| *Ilex belizensis* Lundell; CHIS, HGO, VER; useful (ENVIRONMENTAL USES). |
| *Ilex brandegeeana* Loes.; BCS, CHIS, COAH, COL, DGO, GTO, GRO, JAL, MEX, MICH, MOR, NAY, NLE, OAX, QRO, SLP, SIN, TAMS; Red List: LC; banked. |
| *Ilex condensata* Turcz.; CHIS, OAX, PUE, QRO, SLP, TAB, VER (endemic). |
| *Ilex costaricensis* Donn.Sm.; CHIS, OAX, VER; Red List: VU. |
| *Ilex discolor* Hemsl.; BCS, CHIS, CHIH, COL, CDMX, DGO, GTO, GRO, HGO, JAL, MEX, MICH, MOR, NAY, NLE, OAX, PUE, QRO, SLP, SIN, SON, TAB, TAMS, VER; Red List: LC. |
| *Ilex dugesii* Fernald; GTO, DGO, JAL, MICH, NAY, SLP, SIN, ZAC (endemic). |
| *Ilex guianensis* (Aubl.) Kuntze; CHIS, TAB, VER; Red List: LC. |
| *Ilex pringlei* Standl.; CHIS, HGO, OAX, PUE, VER (endemic). |
| *Ilex quercetorum* I.M.Johnst.; CHIS, GRO, OAX, VER; Red List: VU. |
| *Ilex rubra* S.Watson; CHIH, COAH, DGO, GTO, JAL, NAY, NLE, QRO, SLP, SIN, SON, TAMS, ZAC (endemic). |
| *Ilex servinii* E.Carranza; QRO, SLP (endemic). |
| *Ilex socorroensis* Brandegee; COL (endemic). |
| *Ilex tonii* Lundell; CHIS (endemic). |
| *Ilex vomitoria* Aiton; CHIS; Red List: LC; useful (ENVIRONMENTAL USES). |
| *Ilex mexicana* (Turcz.) Black ex Hemsl.; HGO, JAL, MEX, MICH, MOR, NAY, NLE, SIN, VER (endemic). |
|  |
| **Araliaceae** |
| ***Aralia*** |
| *Aralia excelsa* (Griseb.) J.Wen. |
| *Aralia humilis* Cav.; AGS, CHIS, CHIH, COAH, COL, DGO, GTO, GRO, HGO, JAL, MEX, MICH, MOR, NAY, OAX, PUE, QRO, SIN, SON, VER; banked; useful (ENVIRONMENTAL USES). |
| *Aralia regeliana* Marchal; COAH, DGO, GTO, HGO, NLE, QRO, SLP, TAMS, VER, ZAC (endemic). |
| *Aralia scopulorum* Brandegee; BCN, BCS (endemic). |
| ***Dendropanax*** |
| *Dendropanax arboreus* (L.) Decne. & Planch.; CAM, CHIS, CHIH, COAH, COL, DGO, GRO, HGO, JAL, MEX, MICH, NAY, OAX, PUE, QRO, QROO, SLP, SIN, TAB, TAMS, VER, YUC; banked; useful (ANIMAL FOOD, MEDICINES, MATERIALS, FUELS). |
| *Dendropanax gonatopodus* (Donn.Sm.) A.C.Sm.; CHIS. |
| *Dendropanax hondurensis* M.J.Cannon & Cannon; CHIS; Red List: CR. |
| *Dendropanax leptopodus* (Donn.Sm.) A.C.Sm.; CHIS, GRO, OAX, SLP, VER. |
| *Dendropanax oliganthus* (A.C.Sm.) A.C.Sm.; CHIS, OAX; Red List: CR. |
| *Dendropanax pallidus* M.J.Cannon & Cannon; CHIS. |
| *Dendropanax populifolius* (Marchal) A.C.Sm.; CHIS, OAX (endemic). |
| ***Oreopanax*** |
| *Oreopanax arcanus* A.C.Sm.; CHIS; Red List: VU. |
| *Oreopanax capitatus* (Jacq.) Decne. & Planch.; CHIS, HGO, OAX, PUE, TAB, VER; Red List: LC. |
| *Oreopanax echinops* (Schltdl. & Cham.) Decne. & Planch.; CHIS, COL, DGO, GRO, HGO, JAL, MEX, MICH, OAX, PUE, SIN, VER; Red List: VU; useful (MATERIALS). |
| *Oreopanax flaccidus* Marchal; HGO, OAX, PUE, VER (endemic). |
| *Oreopanax geminatus* Marchal; CHIS (endemic). |
| *Oreopanax peltatus* Linden ex Regel; CHIS, CHIH, COL, DGO, GRO, JAL, MEX, MICH, MOR, NAY, OAX, PUE, SLP, SIN, SON, VER, ZAC; Red List: VU. |
| *Oreopanax platyphyllus* Marchal; CHIS, OAX, VER. |
| *Oreopanax sanderianus* Hemsl.; CHIS, COL, GRO, JAL, OAX; Red List: VU. |
| *Oreopanax xalapensis* (Kunth) Decne. & Planch.; CHIS, COL, DGO, GRO, HGO, JAL, MEX, MICH, MOR, NAY, OAX, PUE, QRO, SLP, SIN, TAB, VER. |
| ***Schefflera*** |
| *Schefflera* *morototoni* (Aubl.) Maguire, Steyerm. & Frodin; CHIS, OAX, TAB, VER; Red List: LC. |
|  |
| **Arecaceae** |
| ***Acoelorraphe*** |
| *Acoelorraphe* *wrightii* (Griseb. & H.Wendl.) H.Wendl. ex Becc.; CAM, CHIS, QROO, TAB, TAMS, VER, YUC; useful (ENVIRONMENTAL USES). |
| ***Acrocomia*** |
| *Acrocomia* *aculeata* (Jacq.) Lodd. ex R.Keith; CAM, CHIS, COL, GRO, HGO, JAL, MEX, MICH, NAY, OAX, PUE, QRO, QROO, SLP, SIN, TAB, TAMS, VER, YUC, ZAC; banked; useful (FOOD, MEDICINES, MATERIALS). |
| ***Astrocaryum*** |
| *Astrocaryum* *mexicanum* Liebm. ex Mart.; CHIS, JAL, NAY, OAX, TAB, VER. |
| ***Attalea*** |
| *Attalea butyracea* (Mutis ex L.f.) Wess.Boer; CAM, CHIS, HGO, OAX, PUE, QROO, TAB, TAMS, VER, YUC; useful (MATERIALS). |
| *Attalea cohune* Mart.; CAM, CHIS, COL, GRO, JAL, MICH, NAY, OAX, QROO, SIN, TAB, YUC. |
| *Attalea guacuyule* (Liebm. ex Mart.) Zona; COL, GRO, JAL, MICH, NAY, OAX (endemic). |
| *Attalea rostrata* Oerst.; CHIS. |
| ***Bactris*** |
| *Bactris* *major* Jacq.; CAM, CHIS, GRO, OAX, QROO, TAB, VER; useful (FOOD). |
| *Bactris mexicana* Mart.; CAM, CHIS, GRO, OAX, PUE, QROO, TAB, VER; useful (MATERIALS). |
| ***Brahea*** |
| *Brahea aculeata* (Brandegee) H.E.Moore; CHIH, DGO, NAY, SIN, SON (endemic); Red List: VU. |
| *Brahea armata* S.Watson; BCN, BCS, SON (endemic); Red List: LC; banked. |
| *Brahea brandegeei* (Purpus) H.E.Moore; BCN, BCS, JAL, MICH (endemic); Red List: LC; banked. |
| *Brahea dulcis* (Kunth) Mart.; CHIS, CHIH, COAH, COL, DGO, GTO, GRO, HGO, JAL, MEX, MICH, MOR, NAY, NLE, OAX, PUE, QRO, SLP, SIN, SON, TAMS, VER; Red List: LC; banked; useful (MEDICINES, MATERIALS). |
| *Brahea edulis* H.Wendl. ex S.Watson; BCN (endemic); Red List: EN. |
| *Brahea moorei* L.H.Bailey ex H.E.Moore; HGO, NLE, QRO, SLP, TAMS (endemic); Red List: LC; NOM-59: Pr; banked. |
| *Brahea nitida* André; CHIS, CHIH, GRO, OAX, PUE, SIN, SON; banked. |
| *Brahea pimo* Becc.; COL, GRO, JAL, MEX, MICH, NAY, OAX, SIN, ZAC (endemic); Red List: VU. |
| *Brahea sarukhanii* H.J.Quero; JAL, NAY (endemic). |
| ***Chamaedorea*** |
| *Chamaedorea tepejilote* Liebm.; CHIS, GRO, HGO, OAX, PUE, TAB, VER; banked; useful (FOOD, ENVIRONMENTAL USES). |
| *Chamaedorea woodsoniana* L.H.Bailey; CHIS, OAX, TAB, VER. |
| ***Coccothrinax*** |
| *Coccothrinax argentea* (Lodd. ex Schult. & Schult.f.) Sarg. ex Becc. |
| *Coccothrinax readii* H.J.Quero; CAM, QROO, YUC (endemic); NOM-59: A. |
| ***Cryosophila*** |
| *Cryosophila nana* (Kunth) Blume; CHIS, COL, GRO, JAL, MICH, NAY, OAX, SIN (endemic); Red List: NT. |
| *Cryosophila stauracantha* (Heynh.) R.J.Evans; CAM, CHIS, GRO, MICH, OAX, QROO, SLP, TAB, VER. |
| ***Euterpe*** |
| *Euterpe precatoria* Mart. |
| ***Gaussia*** |
| *Gaussia gomez-pompae* (H.J.Quero) H.J.Quero; CHIS, OAX, TAB, VER (endemic); Red List: VU. |
| *Gaussia maya* (O.F.Cook) H.J.Quero & Read; CAM, CHIS, OAX, QROO, TAB, VER; Red List: VU; NOM-59: A. |
| ***Geonoma*** |
| *Geonoma interrupta* (Ruiz & Pav.) Mart.; CHIS, OAX, TAB, VER. |
| ***Pseudophoenix*** |
| *Pseudophoenix sargentii* H.Wendl. ex Sarg.; CAM, QROO, YUC. |
| ***Roystonea*** |
| *Roystonea dunlapiana* P.H.Allen; CAM, CHIS, QROO, TAB, VER; Red List: EN. |
| *Roystonea regia* (Kunth) O.F.Cook; CHIS, CAM, QROO, TAB, VER, YUC. |
| ***Sabal*** |
| *Sabal gretherae* H.J.Quero; CAM, QROO, YUC (endemic); Red List: VU. |
| *Sabal mauritiiformis* (H.Karst.) Griseb. & H.Wendl.; CAM, CHIS, OAX, QROO, TAB, VER, YUC. |
| *Sabal mexicana* Mart.; CAM, CHIS, COL, GRO, HGO, JAL, MEX, MICH, NAY, NLE, OAX, PUE, QRO, QROO, SLP, SIN, TAB, TAMS, VER, YUC, ZAC; useful (FOOD, ANIMAL FOOD, MATERIALS). |
| *Sabal pumos* (Kunth) Burret; BCS, DGO, GTO, GRO, JAL, MEX, MICH, MOR, NAY, SIN, SON, ZAC (endemic); Red List: VU. |
| *Sabal uresana* Trel.; BCS, CHIH, SIN, SON (endemic); Red List: VU; NOM-59: Pr. |
| *Sabal yapa* C.Wright ex Becc.; CAM, QROO, YUC. |
| ***Schippia*** |
| *Schippia concolor* Burret; CHIS; Red List: VU. |
| ***Thrinax*** |
| *Thrinax radiata* Lodd. ex Schult. & Schult.f.; CAM, QROO, YUC; NOM-59: A; useful (FOOD, MEDICINES). |
| ***Washingtonia*** |
| *Washingtonia filifera* (Rafarin) H.Wendl. ex de Bary; BCN, BCS, CHIH, COAH, DGO, SIN, SON; Red List: NT; banked; useful. |
| *Washingtonia robusta* H.Wendl.; BCN, BCS, CHIH, SON; banked; useful. |
|  |
| **Asparagaceae** |
| ***Beaucarnea*** |
| *Beaucarnea goldmanii* Rose; CHIS (endemic); NOM-59: A; Cites: II; useful (ENVIRONMENTAL USES). |
| *Beaucarnea gracilis* Lem.; OAX, PUE (endemic); Cites: II; banked; useful (MATERIALS, ENVIRONMENTAL USES, SOCIAL USES). |
| *Beaucarnea hiriartiae* L.Hern.; GRO, OAX (endemic); Cites: II; useful (ENVIRONMENTAL USES). |
| *Beaucarnea pliabilis* (Baker) Rose; CAM, QROO, YUC; NOM-59: A; Cites: II; useful (ENVIRONMENTAL USES). |
| *Beaucarnea purpusii* Rose; PUE (endemic); Cites: II. |
| *Beaucarnea recurvata* (K.Koch & Fintelm.) Lem.; OAX, PUE, TAMS, VER (endemic); Cites: II; useful (ENVIRONMENTAL USES). |
| *Beaucarnea sanctomariana* L.Hern.; OAX (endemic); Cites: II. |
| *Beaucarnea stricta* (K.Koch & Fintelm.) Lem.; OAX, PUE (endemic); Cites: II; banked; useful (MATERIALS). |
| ***Dracaena*** |
| *Dracaena americana* Donn.Sm.; CAM, CHIS, OAX, QROO, TAB, VER, YUC; Red List: LC. |
| ***Furcraea*** |
| *Furcraea flavoviridis* Hook.; HGO (endemic). |
| *Furcraea longaeva* Karw. & Zucc.; OAX, PUE. |
| *Furcraea macdougallii* Matuda; OAX, PUE (endemic). |
| *Furcraea martinezii* García-Mend. & L.de la Rosa; GRO (endemic). |
| *Furcraea parmentieri* (Roezl) García-Mend.; COL, CDMX, GTO, GRO, HGO, JAL, MEX, MICH, MOR, OAX, QRO, VER (endemic). |
| *Furcraea stricta* Jacobi; ND (endemic). |
| *Furcraea undulata* Jacobi; CHIS, TAB (endemic). |
| ***Nolina*** |
| *Nolina azureogladiata* D.Donati; OAX (endemic). |
| *Nolina beldingii* Brandegee; BCS (endemic); banked. |
| *Nolina bigelovii* (Torr.) S.Watson; BCN, SON; Red List: LC. |
| *Nolina durangensis* Trel.; CHIH, DGO (endemic). |
| *Nolina erumpens* (Torr.) S.Watson; CHIH, COAH, DGO. |
| *Nolina excelsa* García-Mend. & E.Solano; OAX (endemic). |
| *Nolina hibernica* Hochstätter & D.Donati; QRO, TAMS (endemic). |
| *Nolina matapensis* Wiggins; CHIH, SON (endemic); useful (MATERIALS). |
| *Nolina parviflora* (Kunth) Hemsl.; AGS, CHIH, COAH, COL, CDMX, DGO, GTO, HGO, JAL, MEX, MICH, MOR, NAY, NLE, OAX, PUE, QRO, SLP, SIN, TAMS, TLAX, VER, ZAC (endemic); banked; useful (MATERIALS). |
| *Nolina rigida* Trel.; ND (endemic). |
| ***Yucca*** |
| *Yucca* × *schottii* Engelm.; CHIH, SON. |
| *Yucca aloifolia* L.; CHIS, GRO, MOR, OAX, PUE, VER, YUC. |
| *Yucca baccata* var. *brevifolia* L.D.Benson & Darrow; CHIH, SON. |
| *Yucca brevifolia* Engelm.; BCN, SON; useful (ENVIRONMENTAL USES). |
| *Yucca capensis* L.W.Lenz; BCS (endemic). |
| *Yucca carnerosana* (Trel.) McKelvey; CHIH, COAH, DGO, GTO, NLE, SLP, SON, TAMS, ZAC; banked; useful (FOOD, MATERIALS, ENVIRONMENTAL USES). |
| *Yucca coahuilensis* Matuda & I.L.Pina; COAH (endemic). |
| *Yucca decipiens* Trel.; AGS, DGO, GTO, JAL, NLE, SLP, ZAC (endemic); banked; useful (FOOD, ANIMAL FOOD, MEDICINES, MATERIALS). |
| *Yucca declinata* Laferr.; SON (endemic); banked. |
| *Yucca desmetiana* Baker; CHIH (endemic). |
| *Yucca elata* (Engelm.) Engelm.; CHIH, COAH, DGO, SON; banked; useful (MATERIALS). |
| *Yucca faxoniana* Sarg.; CHIH, COAH. |
| *Yucca filifera* Chabaud; AGS, CHIH, COAH, CDMX, DGO, GTO, HGO, JAL, MEX, MICH, NLE, OAX, PUE, QRO, SLP, TAMS, TLAX, VER, ZAC (endemic); banked; useful (FOOD, ANIMAL FOOD, MEDICINES, MATERIALS, FUELS, ENVIRONMENTAL USES). |
| *Yucca gigantea* Lem. |
| *Yucca grandiflora* Gentry; CHIH, SON (endemic). |
| *Yucca jaliscensis* (Trel.) Trel.; COL, DGO, GTO, JAL, MEX, MICH, NAY, SIN, ZAC (endemic); useful (ENVIRONMENTAL USES). |
| *Yucca lacandonica* Gómez Pompa & J.Valdés; CAM, CHIS, OAX, TAB, VER (endemic); NOM-59: A. |
| *Yucca linearifolia* Clary; COAH, NLE (endemic). |
| *Yucca madrensis* Gentry; CHIH, SON (endemic); useful (FOOD). |
| *Yucca mixtecana* García-Mend.; OAX, PUE (endemic); banked; useful (ENVIRONMENTAL USES). |
| *Yucca periculosa* Baker; OAX, PUE, TLAX, VER (endemic); banked; useful (FOOD). |
| *Yucca potosina* Rzed.; SLP, TAMS (endemic). |
| *Yucca queretaroensis* Piña Luján; GTO, HGO, QRO, SLP (endemic); NOM-59: Pr; Cites: II; useful (ENVIRONMENTAL USES). |
| *Yucca reverchonii* Trel.; COAH. |
| *Yucca schidigera* Roezl ex Ortgies; BCN, SON; banked; useful (ENVIRONMENTAL USES). |
| *Yucca treculeana* Carrière; AGS, COAH, DGO, NLE, QRO, SLP, TAMS, VER. |
| *Yucca valida* Brandegee; BCN, BCS (endemic); banked; useful. |
|  |
| **Asteraceae** |
| ***Acourtia*** |
| *Acourtia* *glomeriflora* (A.Gray) Reveal & R.M.King; BCS (endemic); banked; useful. |
| ***Ageratina*** |
| *Ageratina* *mairetiana* (DC.) R.M.King & H.Rob.; AGS, CHIS, COL, CDMX, DGO, GTO, GRO, HGO, JAL, MEX, MICH, MOR, NAY, OAX, PUE, QRO, SLP, SIN, TAMS, TLAX, VER, ZAC; Red List: LC; banked; useful (ANIMAL FOOD). |
| ***Amolinia*** |
| *Amolinia heydeana* (B.L.Rob.) R.M.King & H.Rob.; CHIS. |
| ***Barkleyanthus*** |
| *Barkleyanthus salicifolius* (Kunth) H.Rob. & Brettell; AGS, CHIS, CHIH, COAH, COL, CDMX, DGO, GTO, GRO, HGO, JAL, MEX, MICH, MOR, NAY, NLE, OAX, PUE, QRO, SLP, SIN, SON, TAMS, TLAX, VER, ZAC; banked; useful (MEDICINES, FUELS). |
| ***Bartlettina*** |
| *Bartlettina platyphylla* (B.L.Rob.) R.M.King & H.Rob.; CHIS, HGO, OAX, PUE, VER. |
| ***Clibadium*** |
| *Clibadium arboreum* Donn.Sm.; CHIS, COL, GRO, HGO, JAL, MEX, NAY, OAX, PUE, QRO, SLP, TAB, VER; Red List: LC; banked. |
| ***Critonia*** |
| *Critonia daleoides* DC.; CAM, CHIS, HGO, NLE, OAX, PUE, QRO, QROO, SLP, TAB, TAMS, VER, YUC; Red List: LC; banked. |
| *Critonia hebebotrya* DC.; CHIS, CHIH, COL, DGO, GRO, JAL, MEX, MICH, MOR, NAY, OAX, PUE, QRO, SLP, SIN, SON, ZAC; banked. |
| *Critonia morifolia* (Mill.) R.M.King & H.Rob.; CAM, CHIS, COL, GRO, HGO, JAL, NLE, OAX, PUE, QRO, QROO, SLP, SIN, TAB, TAMS, VER; Red List: LC; banked; useful. |
| *Critonia paneroi* B.L.Turner; GRO (endemic). |
| ***Dendroviguiera*** |
| *Dendroviguiera puruana* (Paray) E.E.Schill. & Panero; CHIS, GRO, MEX, MICH (endemic). |
| *Dendroviguiera quinqueradiata* (Cav.) E.E.Schill. & Panero; AGS, COL, DGO, GTO, GRO, JAL, MICH, NAY, QRO, ZAC (endemic). |
| *Dendroviguiera sphaerocephala* (DC.) E.E.Schill. & Panero; GTO, GRO, HGO, JAL, MEX, MICH, MOR, OAX, PUE (endemic). |
| ***Eremosis*** |
| *Eremosis leiocarpa* Gleason. |
| *Eremosis salicifolia* (DC.) Gleason; AGS, CHIS, COL, CDMX, DGO, GRO, HGO, JAL, MEX, MICH, MOR, NAY, OAX, PUE, QRO, SIN, TAMS, VER, ZAC (endemic); banked. |
| *Eremosis shannonii* Gleason; CHIS. |
| *Eremosis triflosculosa* Gleason; AGS, CHIS, CHIH, COL, DGO, GRO, JAL, MEX, MICH, MOR, NAY, NLE, OAX, PUE, SIN, SON, TAMS, VER, ZAC. |
| ***Gochnatia*** |
| *Gochnatia smithii* B.L.Rob. & Greenm.; OAX, PUE (endemic); banked; useful. |
| ***Koanophyllon*** |
| *Koanophyllon albicaule* (Sch.Bip. ex Klatt) R.M.King & H.Rob.; CAM, CHIS, COL, GRO, HGO, JAL, MEX, MICH, MOR, NAY, OAX, PUE, QRO, QROO, SLP, SIN, TAB, TAMS, VER, YUC, ZAC. |
| *Koanophyllon pittieri* (Klatt) R.M.King & H.Rob.; CHIS, OAX, QROO, TAB, VER. |
| ***Lepidaploa*** |
| *Lepidaploa polypleura* (S.F.Blake) H.Rob.; CHIS, OAX. |
| ***Montanoa*** |
| *Montanoa bipinnatifida* (Kunth) K.Koch; COL, DGO, GTO, GRO, JAL, MEX, MICH, MOR, NAY, OAX, PUE, SIN (endemic); banked. |
| *Montanoa grandiflora* Hemsl.; CAM, CHIS, COL, CDMX, GTO, GRO, HGO, JAL, MEX, MICH, MOR, OAX, PUE, QRO, QROO, SLP, SIN, TAB, TAMS, VER, YUC, ZAC (endemic); banked. |
| *Montanoa guatemalensis* B.L.Rob. & Greenm.; Red List: LC. |
| *Montanoa leucantha* (Lag.) S.F.Blake; AGS, CHIS, CHIH, COL, CDMX, DGO, GTO, GRO, HGO, JAL, MEX, MICH, MOR, NAY, NLE, OAX, PUE, QRO, SLP, SIN, SON, TAMS, TLAX, ZAC; banked; useful. |
| *Montanoa revealii* H.Rob.; GRO, OAX (endemic). |
| *Montanoa* tomentosa Cerv.; CHIS, CHIH, COL, CDMX, DGO, GTO, GRO, HGO, JAL, MEX, MICH, MOR, NAY, NLE, OAX, PUE, QRO, SLP, SIN, SON, TAMS, TLAX, VER, ZAC; banked; useful (ANIMAL FOOD, MEDICINES, FUELS). |
| ***Perymenium*** |
| *Perymenium grande* Hemsl.; CHIS, OAX, TAB; Red List: LC. |
| ***Podachaenium*** |
| *Podachaenium eminens* (Lag.) Sch.Bip.; CHIS, COL, DGO, GRO, HGO, JAL, MICH, NAY, OAX, PUE, QRO, SLP, SIN, TAB, VER; Red List: LC; banked; useful (MATERIALS). |
| ***Rojasianthe*** |
| *Rojasianthe superba* Standl. & Steyerm.; CHIS. |
| ***Roldana*** |
| *Roldana candicans* (Née) Villaseñor, S.Valencia & Coombes; CHIS, CHIH, COL, CDMX, DGO, GTO, GRO, HGO, JAL, MEX, MICH, MOR, NAY, OAX, PUE, QRO, SLP, SIN, SON, TAMS, TLAX, VER, ZAC; useful (FUELS, ENVIRONMENTAL USES). |
| ***Squamopappus*** |
| *Squamopappus skutchii* (S.F.Blake) R.K.Jansen, N.A.Harriman & Urbatsch; CHIS. |
| ***Telanthophora*** |
| *Telanthophora cobanensis* (J.M.Coult.) H.Rob. & Brettell; CHIS, HGO, OAX, VER. |
| *Telanthophora* *grandifolia* (Less.) H.Rob. & Brettell; CHIS, COL, GRO, HGO, JAL, OAX, PUE, QRO, SLP, VER. |
| *Telanthophora uspantanensis* (J.M.Coult.) H.Rob. & Brettell; CHIS, OAX, PUE, VER (endemic). |
| ***Tithonia*** |
| *Tithonia koelzii* McVaugh; COL, JAS (endemic). |
| ***Verbesina*** |
| *Verbesina apleura* S.F.Blake; CHIS. |
| *Verbesina breedlovei* B.L.Turner; CHIS, COL, GRO, JAL, MEX, MICH, OAX (endemic). |
| *Verbesina furfuracea* McVaugh; COL, JAL, MICH (endemic). |
| *Verbesina oerstediana* Benth.; CHIS, OAX. |
| ***Vernonanthura*** |
| *Vernonanthura patens* (Kunth) H.Rob.; CAM, CHIS, COL, GRO, HGO, JAL, MEX, MICH, MOR, NAY, OAX, PUE, QRO, QROO, SLP, SIN, TAB, TAMS, VER, YUC, ZAC; Red List: LC; banked; useful (ANIMAL FOOD, MEDICINES, MATERIALS). |
|  |
| **Berberidaceae** |
| ***Berberis*** |
| *Berberis volcania* (Standl. & Steyerm.) Marroq. & Laferr.; CHIS. |
|  |
| **Betulaceae** |
| ***Alnus*** |
| *Alnus acuminata* Kunth; AGS, CHIS, CHIH, COL, CDMX, DGO, GTO, GRO, HGO, JAL, MEX, MICH, MOR, NAY, OAX, PUE, QRO, SLP, SIN, SON, TAMS, TLAX, VER, ZAC; Red List: LC; banked; useful (ANIMAL FOOD, MEDICINES, MATERIALS, FUELS, ENVIRONMENTAL USES). |
| *Alnus jorullensis* Kunth; AGS, CHIS, CHIH, COL, CDMX, DGO, GTO, GRO, HGO, JAL, MEX, MICH, MOR, NAY, OAX, PUE, QRO, SLP, SIN, SON, TLAX, VER, ZAC; Red List: LC; banked; useful (ANIMAL FOOD, MEDICINES, MATERIALS, FUELS, ENVIRONMENTAL USES). |
| *Alnus oblongifolia* Torr.; CHIH, DGO, NAY, SIN, SON; Red List: LC. |
| *Alnus rhombifolia* Nutt.; BCN; Red List: LC. |
| ***Carpinus*** |
| *Carpinus caroliniana* Walter; Red List: LC; NOM-59: A; banked. |
| ***Ostrya*** |
| *Ostrya virginiana* (Mill.) K.Koch; CHIS, CHIH, COAH, COL, DGO, GTO, GRO, HGO, JAL, MEX, MICH, NAY, NLE, OAX, PUE, QRO, SLP, SIN, SON, TAB, TAMS, TLAX, VER; Red List: LC; NOM-59: Pr; banked. |
|  |
| **Bignoniaceae** |
| ***Amphitecna*** |
| *Amphitecna apiculata* A.H.Gentry; CAM, CHIS, GRO, OAX, PUE, QROO, TAB, VER, YUC. |
| *Amphitecna donnell-smithii* (Sprague) L.O.Williams; CHIS, TAB. |
| *Amphitecna macrophylla* (Seem.) Miers ex Baill.; CHIS, OAX, TAB, VER. |
| *Amphitecna regalis* (Linden) A.H.Gentry; CHIS, OAX, TAB, VER (endemic). |
| *Amphitecna sessilifolia* (Donn.Sm.) L.O.Williams; Red List: VU. |
| *Amphitecna silvicola* L.O.Williams; CHIS. |
| *Amphitecna steyermarkii* (A.H.Gentry) A.H.Gentry; CHIS, OAX, VER. |
| *Amphitecna tuxtlensis* A.H.Gentry; OAX, VER (endemic). |
| ***Astianthus*** |
| *Astianthus* *viminalis* (Kunth) Baill.; CHIS, COL, GRO, HGO, JAL, MEX, MICH, MOR, NAY, OAX, PUE, QRO, SLP, TAMS, VER; banked; useful (MEDICINES, FUELS, ENVIRONMENTAL USES, SOCIAL USES). |
| ***Chilopsis*** |
| *Chilopsis linearis* (Cav.) Sweet; BCN, CHIH, COAH, DGO, NLE, SLP, SON, TAMS, ZAC; Red List: LC; banked; useful. |
| ***Crescentia*** |
| *Crescentia alata* Kunth; BCN, BCS, CHIS, CHIH, COL, DGO, GRO, HGO, JAL, MEX, MICH, MOR, NAY, OAX, PUE, QRO, SLP, SIN, SON, TAB, TAMS, VER, ZAC; Red List: LC; banked; useful (FOOD, ANIMAL FOOD, MEDICINES, MATERIALS, FUELS). |
| *Crescentia cujete* L.; CAM, CHIS, GRO, HGO, MOR, NAY, OAX, PUE, QROO, SIN, TAB, TAMS, VER, YUC; Red List: LC; useful (MEDICINES, MATERIALS, ENVIRONMENTAL USES). |
| ***Godmania*** |
| *Godmania aesculifolia* (Kunth) Standl.; CAM, CHIS, GRO, JAL, MEX, MICH, NAY, OAX, QROO, TAB, VER, YUC; Red List: LC. |
| ***Handroanthus*** |
| *Handroanthus chrysanthus* (Jacq.) S.O.Grose; CAM, CHIS, CHIH, COL, DGO, GRO, JAL, MEX, MICH, NAY, OAX, PUE, QROO, SIN, SON, TAB, TAMS, VER, YUC, ZAC; banked. |
| *Handroanthus guayacan* (Seem.) S.O.Grose; CAM, CHIS, OAX, QROO, TAB, VER. |
| *Handroanthus impetiginosus* (Mart. ex DC.) Mattos; AGS, CAM, CHIS, CHIH, COAH, COL, DGO, GRO, JAL, MEX, MICH, MOR, NAY, OAX, PUE, SIN, SON, YUC; Red List: LC; banked; useful. |
| ***Parmentiera*** |
| *Parmentiera aculeata* (Kunth) Seem.; BCS, CAM, CHIS, COL, GRO, HGO, JAL, MEX, MICH, MOR, NAY, OAX, PUE, QRO, QROO, SLP, SIN, TAB, TAMS, VER, YUC; Red List: LC; banked; useful (FOOD, ANIMAL FOOD, MEDICINES, ENVIRONMENTAL USES). |
| *Parmentiera parviflora* Lundell; CHIS, TAB. |
| ***Roseodendron*** |
| *Roseodendron donnell-smithii* (Rose) Miranda; CAM, CHIS, COL, GRO, JAL, MEX, MICH, MOR, NAY, OAX, SIN, TAB, VER; banked; useful (MATERIALS). |
| ***Tabebuia*** |
| *Tabebuia rosea* (Bertol.) Bertero ex A.DC.; CAM, CHIS, COL, DGO, GRO, HGO, JAL, MEX, MICH, MOR, NAY, OAX, PUE, QRO, QROO, SLP, SIN, SON, TAB, TAMS, VER, YUC, ZAC; Red List: LC; banked; useful (ANIMAL FOOD, MEDICINES, MATERIALS, FUELS, ENVIRONMENTAL USES). |
| ***Tecoma*** |
| *Tecoma stans* (L.) Juss. ex Kunth; AGS, BCS, CAM, CHIS, CHIH, COAH, COL, CDMX, DGO, GTO, GRO, HGO, JAL, MEX, MICH, MOR, NAY, NLE, OAX, PUE, QRO, QROO, SLP, SIN, SON, TAB, TAMS, TLAX, VER, YUC, ZAC; banked; useful (ANIMAL FOOD, MEDICINES, POISONS, MATERIALS, FUELS, ENVIRONMENTAL USES, SOCIAL USES). |
|  |
| **Bixaceae** |
| ***Bixa*** |
| *Bixa orellana* L.; CAM, CHIS, COL, GRO, JAL, MEX, MICH, MOR, NAY, OAX, PUE, QROO, SIN, TAB, VER, YUC; Red List: LC; banked; useful (FOOD, ANIMAL FOOD, MEDICINES, POISONS, MATERIALS, FUELS). |
| ***Cochlospermum*** |
| *Cochlospermum vitifolium* (Willd.) Spreng.; BCS, CAM, CHIS, CHIH, COL, DGO, GRO, HGO, JAL, MEX, MICH, MOR, NAY, OAX, PUE, QROO, SLP, SIN, SON, TAB, TAMS, VER, YUC, ZAC; Red List: LC; banked; useful (MEDICINES). |
|  |
| **Boraginaceae** |
| ***Bourreria*** |
| *Bourreria andrieuxii* (A.DC.) Hemsl.; CHIS, GRO, MEX, MICH, MOR, OAX, PUE (endemic); banked. |
| *Bourreria hintonii* (La Llave & Lex.) I.M.Johnst.; GRO, JAL, MICH, OAX (endemic). |
| *Bourreria huanita* (Lex.) Hemsl.; CAM, CHIS, COL, GRO, JAL, MEX, MICH, OAX, QROO, TAB; useful (MEDICINES). |
| *Bourreria mollis* Standl.; VER (endemic). |
| *Bourreria motaguensis* Véliz, G.Campos & J.S.Mill.; CHIS, OAX. |
| *Bourreria pulchra* (Millsp.) Millsp. ex Greenm.; CAM, CHIS, QROO, YUC (endemic); banked. |
| *Bourreria rekoi* Standl.; OAX (endemic). |
| *Bourreria spathulata* (Miers) Hemsl.; GRO, OAX, PUE (endemic). |
| *Bourreria succulenta* Jacq.; OAX, QROO, YUC; Red List: LC. |
| *Bourreria superba* I.M.Johnst.; COL, GRO, JAL, MICH, NAY, SIN. |
| ***Cordia*** |
| *Cordia alliodora* (Ruiz & Pav.) Oken; CAM, CHIS, COAH, COL, DGO, GTO, GRO, HGO, JAL, MEX, MICH, MOR, NAY, OAX, PUE, QRO, QROO, SLP, SIN, SON, TAB, TAMS, VER, YUC; Red List: LC; banked; useful (FOOD, ANIMAL FOOD, MEDICINES, MATERIALS, FUELS, ENVIRONMENTAL USES, SOCIAL USES). |
| *Cordia bicolor* A.DC.; CHIS, OAX, VER; Red List: LC. |
| *Cordia boissieri* A.DC.; AGS, COAH, GTO, GRO, HGO, MOR, NLE, QRO, SLP, SIN, TAMS, VER; Red List: LC; banked; useful. |
| *Cordia colimensis* I.M.Johnst.; COL, JAL (endemic). |
| *Cordia cordiformis* I.M.Johnst.; CHIS, COL, JAL. |
| *Cordia dentata* Poir.; CAM, CHIS, COL, GRO, JAL, MEX, MICH, MOR, NAY, OAX, PUE, QRO, QROO, SLP, SIN, TAB, TAMS, VER, YUC; Red List: LC; banked. |
| *Cordia dichotoma* G.Forst.; CAM, TAB; Red List: LC; useful (FOOD, MEDICINES, MATERIALS, ENVIRONMENTAL USES). |
| *Cordia diversifolia* Pav. ex A.DC.; CAM, CHIS, COL, GRO, JAL, NAY, OAX, QROO, SIN, TAB, VER, YUC; Red List: LC. |
| *Cordia dodecandra* A.DC.; CAM, CHIS, COL, GRO, MICH, MOR, OAX, QROO, TAB, VER, YUC; useful (FOOD, MEDICINES, MATERIALS, ENVIRONMENTAL USES, SOCIAL USES). |
| *Cordia elaeagnoides* A.DC.; CHIS, COL, GRO, JAL, MEX, MICH, MOR, OAX, QROO, SIN (endemic); banked; useful (MEDICINES, MATERIALS, ENVIRONMENTAL USES). |
| *Cordia eriostigma* Pittier; CHIS, COL, NAY, OAX, TAB, VER; Red List: LC. |
| *Cordia gerascanthus* L.; CAM, CHIS, COL, GRO, JAL, MICH, NAY, OAX, PUE, QROO, SLP, TAB, TAMS, VER, YUC; useful (FUELS). |
| *Cordia gracilipes* I.M.Johnst.; GRO, OAX (endemic). |
| *Cordia guerkeana* Loes.; CAM, OAX, QROO (endemic). |
| *Cordia macvaughii* J.S.Mill.; GRO, JAL, MICH (endemic). |
| *Cordia megalantha* S.F.Blake; CHIS, OAX, TAB, VER; banked; useful (MATERIALS). |
| *Cordia morelosana* Standl.; COL, GRO, JAL, MEX, MICH, MOR, OAX, PUE (endemic); useful (MEDICINES). |
| *Cordia panamensis* L.Riley; CHIS; Red List: LC. |
| *Cordia prunifolia* I.M.Johnst.; CHIS, COL, GRO, JAL, NAY, OAX, VER; Red List: LC. |
| *Cordia salvadorensis* Standl.; CAM, CHIS, COL, GRO, JAL, MEX, MICH, NAY, OAX, SIN, VER; Red List: LC. |
| *Cordia sonorae* Rose; CHIH, DGO, GRO, JAL, MICH, NAY, OAX, PUE, SIN, SON (endemic). |
| *Cordia stellifera* I.M.Johnst.; CAM, CHIS, OAX, QROO, SLP, TAB, VER, YUC; Red List: LC; banked. |
| *Cordia stenoclada* I.M.Johnst.; CAM, CHIS, OAX, TAB, VER; Red List: LC. |
| *Cordia tinifolia* Willd. ex Roem. & Schult.; GRO, MEX, NAY, OAX (endemic). |
| *Cordia truncatifolia* Bartlett; CHIS, GRO, JAL, MEX, OAX. |
| ***Ehretia*** |
| *Ehretia anacua* (Terán & Berland.) I.M.Johnst.; COAH, GTO, GRO, HGO, JAL, MICH, NLE, QRO, SLP, TAMS, VER; Red List: LC; banked. |
| *Ehretia latifolia* Loisel.; CAM, CHIS, COL, GTO, GRO, HGO, JAL, MEX, MICH, MOR, OAX, PUE, QRO, QROO, TLAX, VER; Red List: LC; banked; useful (FUELS). |
| *Ehretia tinifolia* L.; CAM, CHIS, CHIH, GRO, HGO, JAL, MEX, MICH, MOR, NAY, OAX, PUE, QRO, QROO, SLP, SIN, SON, TAB, TAMS, VER, YUC; useful (ANIMAL FOOD, MEDICINES, MATERIALS, FUELS, ENVIRONMENTAL USES). |
| ***Lepidocordia*** |
| *Lepidocordia williamsii* (I.M.Johnst.) J.S.Mill.; CHIS, MICH, QROO. |
| ***Wigandia*** |
| *Wigandia urens* (Ruiz & Pav.) Kunth; AGS, CHIS, COL, CDMX, DGO, GTO, GRO, HGO, JAL, MEX, MICH, MOR, NAY, OAX, PUE, QRO, SLP, SIN, SON, TLAX, VER, YUC, ZAC; Red List: LC; banked; useful (ANIMAL FOOD, MEDICINES, SOCIAL USES). |
|  |
| **Brunelliaceae** |
| ***Brunellia*** |
| *Brunellia mexicana* Standl.; CHIS, GRO, HGO, MEX, OAX, PUE, VER; Red List: LC; banked. |
|  |
| **Burseraceae** |
| ***Beiselia*** |
| *Beiselia mexicana* Forman; MICH (endemic); banked. |
| ***Bursera*** |
| *Bursera altijuga* Rzed., Calderón & Medina; OAX, PUE (endemic). |
| *Bursera aptera* Ramírez; COL, GRO, HGO, JAL, MEX, MICH, MOR, OAX, PUE, VER (endemic); Red List: LC; banked; useful (MEDICINES, POISONS, MATERIALS, FUELS, ENVIRONMENTAL USES, SOCIAL USES). |
| *Bursera arida* (Rose) Standl.; OAX, PUE (endemic); Red List: VU; banked; useful (MEDICINES, MATERIALS, FUELS, ENVIRONMENTAL USES). |
| *Bursera ariensis* (Kunth) McVaugh & Rzed.; CHIS, COL, GRO, JAL, MEX, MICH, MOR, NAY, OAX, PUE (endemic); Red List: LC. |
| *Bursera aspleniifolia* Brandegee; COL, JAL, MEX, OAX, PUE, VER (endemic); Red List: VU. |
| *Bursera attenuata* (Rose) L.Riley; DGO, GRO, JAL, NAY, SIN, SON (endemic); Red List: LC. |
| *Bursera bicolor* (Willd. ex Schltdl.) Engl.; GRO, JAL, MEX, MICH, MOR, OAX, PUE (endemic); Red List: LC. |
| *Bursera biflora* (Rose) Standl.; OAX, PUE (endemic); Red List: NT; banked; useful (MATERIALS, FUELS, SOCIAL USES). |
| *Bursera bipinnata* (Moc. & Sessé ex DC.) Engl.; AGS, CHIS, CHIH, COL, DGO, GTO, GRO, HGO, JAL, MEX, MICH, MOR, NAY, OAX, PUE, SIN, SON, VER, ZAC; Red List: LC; useful (MEDICINES, MATERIALS, FUELS, ENVIRONMENTAL USES, SOCIAL USES). |
| *Bursera bolivarii* Rzed.; GRO, OAX, PUE (endemic); Red List: VU. |
| *Bursera bonetii* Rzed.; GRO, OAX (endemic). |
| *Bursera cerasiifolia* Brandegee; BCN, BCS (endemic); Red List: VU. |
| *Bursera chemapodicta* Rzed. & E.Ortíz; GRO (endemic); Red List: VU. |
| *Bursera cinerea* Engl.; OAX, PUE, VER (endemic); Red List: NT; useful (MATERIALS, FUELS). |
| *Bursera citronella* McVaugh & Rzed.; COL, GRO, JAL, MICH, OAX (endemic). |
| *Bursera collina* Brandegee; DGO, SIN (endemic); Red List: EN. |
| *Bursera confusa* (Rose) Engl.; CHIH, COL, GRO, JAL, MEX, MICH, NAY, SON (endemic). |
| *Bursera copallifera* (Moc. & Sessé ex DC.) Bullock; COL, DGO, GTO, GRO, HGO, JAL, MEX, MICH, MOR, NAY, OAX, PUE, VER, ZAC (endemic); Red List: LC; banked; useful (MEDICINES, MATERIALS, FUELS, ENVIRONMENTAL USES, SOCIAL USES). |
| *Bursera coyucensis* Bullock; GRO, JAL, MEX, MICH (endemic). |
| *Bursera crenata* Paul G.Wilson; COL, GRO, JAL, MEX, MICH (endemic). |
| *Bursera cuneata* (Schltdl.) Engl.; CAM, CDMX, GTO, GRO, HGO, JAL, MEX, MICH, MOR, OAX, PUE, QRO, SLP, ZAC (endemic); banked; useful (MATERIALS, FUELS). |
| *Bursera denticulata* McVaugh & Rzed.; COL, GRO, JAL, MICH, NAY (endemic). |
| *Bursera discolor* Rzed.; CHIH, COL, GRO, JAL, MEX, MICH, MOR, NAY, OAX, PUE, SON (endemic). |
| *Bursera epinnata* (Rose) Engl.; BCN, BCS, COL, JAL (endemic); banked. |
| *Bursera esparzae* Rzed., Calderón & Medina; OAX (endemic). |
| *Bursera excelsa* (Kunth) Engl.; AGS, CHIS, COL, DGO, GTO, GRO, JAL, MEX, MICH, MOR, NAY, OAX, QRO, SIN, VER, ZAC; Red List: LC. |
| *Bursera fagaroides* (Kunth) Engl.; AGS, BCN, BCS, CHIS, CHIH, COAH, COL, CDMX, DGO, GTO, GRO, HGO, JAL, MEX, MICH, MOR, NAY, NLE, OAX, PUE, QRO, SLP, SIN, SON, TAMS, VER, ZAC; banked; useful (ANIMAL FOOD, MATERIALS, FUELS, ENVIRONMENTAL USES, SOCIAL USES). |
| *Bursera filicifolia* Brandegee; BCS, SON (endemic); banked. |
| *Bursera fragilis* S.Watson; CHIH, DGO, SIN, SON (endemic). |
| *Bursera fragrantissima* Bullock; COL, GRO, MICH (endemic). |
| *Bursera galeottiana* Engl.; GTO, GRO, OAX, PUE, QRO, SLP (endemic); useful (ANIMAL FOOD, MATERIALS, FUELS, ENVIRONMENTAL USES). |
| *Bursera glabrifolia* (Kunth) Engl.; CHIS, COL, GRO, JAL, MEX, MICH, MOR, NAY, OAX, PUE, SON, VER (endemic); banked; useful (MATERIALS, FUELS). |
| *Bursera grandifolia* (Schltdl.) Engl.; BCS, CHIS, CHIH, COL, DGO, GTO, GRO, JAL, MEX, MICH, MOR, NAY, OAX, PUE, SIN, SON, VER, ZAC (endemic). |
| *Bursera graveolens* (Kunth) Triana & Planch.; CAM, CHIS, HGO, MEX, OAX, PUE, QRO, QROO, SLP, TAB, VER, YUC. |
| *Bursera heliae* Rzed. & Calderón; OAX (endemic). |
| *Bursera heteresthes* Bullock; CAM, CHIS, COL, GRO, JAL, MEX, MICH, OAX, QROO. |
| *Bursera hindsiana* (Benth.) Engl.; BCN, BCS, COL, JAL, SON, ZAC (endemic). |
| *Bursera hintonii* Bullock; GRO, MEX, MICH, OAX (endemic). |
| *Bursera infernidialis* Guevara & Rzed.; GRO, JAL, MEX, MICH, OAX (endemic). |
| *Bursera instabilis* McVaugh & Rzed.; COL, GRO, JAL, MICH, NAY, OAX, PUE, ZAC (endemic). |
| *Bursera isthmica* Rzed. & Calderón; OAX (endemic). |
| *Bursera jerzyi* Medina; OAX (endemic). |
| *Bursera kerberi* Engl.; BCS, CHIS, COL, GRO, JAL, MEX, MICH, NAY, OAX, ZAC (endemic). |
| *Bursera krusei* Rzed.; COL, GRO, JAL, MEX, MICH, MOR, NAY, OAX, ZAC (endemic). |
| *Bursera lancifolia* (Schltdl.) Engl.; BCS, CHIH, GRO, HGO, JAL, MEX, MICH, MOR, NAY, OAX, PUE, QRO, SLP, SIN, SON, VER (endemic); useful (MEDICINES). |
| *Bursera laurihuertae* Rzed. & Calderón; OAX (endemic); Red List: VU. |
| *Bursera laxiflora* S.Watson; BCN, BCS, CHIH, GRO, SIN, SON (endemic); useful (MEDICINES, MATERIALS, FUELS, ENVIRONMENTAL USES). |
| *Bursera linanoe* (La Llave) Rzed., Calderón & Medina; GRO, HGO, HGO, MEX, MICH, MOR, OAX, PUE, VER (endemic); banked. |
| *Bursera longipes* (Rose) Standl.; CHIS, GRO, MEX, MICH, MOR, OAX, PUE (endemic). |
| *Bursera macvaughiana* Cuevas & Rzed.; COL, JAL (endemic). |
| *Bursera madrigalii* Rzed. & Calderón; MEX, MICH (endemic). |
| *Bursera medranoana* Rzed. & E.Ortíz; HGO, QRO, VER (endemic); Red List: EN. |
| *Bursera microphylla* A.Gray; BCN, BCS, CHIH, SON, ZAC; banked; useful (MEDICINES). |
| *Bursera mirandae* C.A.Toledo; CHIS, GRO, MEX, OAX, PUE (endemic); useful (MATERIALS, FUELS, ENVIRONMENTAL USES). |
| *Bursera morelensis* Ramírez; DGO, GTO, GRO, HGO, JAL, MEX, MICH, MOR, OAX, PUE, QRO, SLP, VER, ZAC; Red List: LC; banked; useful (ANIMAL FOOD, MATERIALS, FUELS, ENVIRONMENTAL USES). |
| *Bursera multifolia* (Rose) Engl.; JAL, ZAC (endemic). |
| *Bursera multijuga* Engl.; AGS, COL, DGO, GRO, JAL, MICH, NAY, SIN, SON, ZAC (endemic). |
| *Bursera occulta* McVaugh & Rzed.; COL, MICH (endemic). |
| *Bursera ovalifolia* (Schltdl.) Engl.; CHIS, COL, GRO, JAL, MEX, MICH, OAX. |
| *Bursera palaciosii* Rzed. & Calderón; JAL (endemic). |
| *Bursera palmeri* S.Watson; AGS, COL, DGO, GTO, GRO, JAL, MEX, MICH, NAY, OAX, QRO, SLP, SIN, ZAC (endemic); useful (FUELS). |
| *Bursera paradoxa* Guevara & Rzed.; GRO, MICH (endemic). |
| *Bursera penicillata* (Sessé & Moc. ex DC.) Engl.; AGS, CHIH, COL, DGO, GTO, GRO, HGO, JAL, MEX, MICH, MOR, NAY, OAX, QROO, SLP, SIN, SON, VER, YUC, ZAC (endemic); useful (ENVIRONMENTAL USES). |
| *Bursera pontiveteris* Rzed., Calderón & Medina; OAX, PUE (endemic). |
| *Bursera ribana* Rzed. & Calderón; COL, JAL, MICH (endemic). |
| *Bursera roseana* Rzed., Calderón & Medina; AGS, COL, DGO, GRO, JAL, MEX, MICH, NAY, OAX, ZAC (endemic). |
| *Bursera rzedowskii* C.A.Toledo; GRO (endemic). |
| *Bursera sarcopoda* Paul G.Wilson; COL, GRO, JAL, MICH, OAX (endemic). |
| *Bursera sarukhanii* Guevara & Rzed.; GRO, JAL, MICH (endemic). |
| *Bursera schlechtendalii* Engl.; CAM, CHIS, COAH, COL, DGO, GTO, GRO, HGO, JAL, MEX, MICH, MOR, NAY, NLE, OAX, PUE, QRO, QROO, SLP, TAMS, VER, YUC, ZAC; banked; useful (MEDICINES). |
| *Bursera silviae* Rzed. & Calderón; OAX (endemic). |
| *Bursera simaruba* (L.) Sarg.; AGS, CAM, CHIS, CHIH, COL, DGO, GTO, GRO, HGO, JAL, MEX, MICH, MOR, NAY, OAX, PUE, QRO, QROO, SLP, SIN, SON, TAB, TAMS, VER, YUC, ZAC; banked; useful (ANIMAL FOOD, MEDICINES, MATERIALS, FUELS, ENVIRONMENTAL USES). |
| *Bursera simplex* Rzed. & Calderón; OAX (endemic). |
| *Bursera stenophylla* Sprague & L.Riley; CHIH, DGO, SIN, SON (endemic). |
| *Bursera submoniliformis* Engl.; GRO, HGO, MEX, MICH, MOR, OAX, PUE, VER (endemic); banked; useful (ANIMAL FOOD, MATERIALS, SOCIAL USES). |
| *Bursera suntui* C.A.Toledo; GRO, OAX, PUE (endemic). |
| *Bursera tecomaca* (DC.) Standl.; COL, CDMX, GRO, JAL, MEX, MICH (endemic). |
| *Bursera toledoana* Rzed. & Calderón; GRO, MICH (endemic). |
| *Bursera trifoliolata* Bullock; DGO, GRO, JAL, MEX, MICH, NAY (endemic); useful (MEDICINES). |
| *Bursera trimera* Bullock; COL, GRO, JAL, MEX, MICH (endemic). |
| *Bursera vazquezyanesii* Rzed. & Calderón; JAL (endemic). |
| *Bursera vejar-vazquezii* Miranda; GRO, MEX, MICH, MOR, OAX, PUE (endemic). |
| *Bursera velutina* Bullock; GRO, MEX, MICH, OAX, PUE (endemic). |
| *Bursera xochipalensis* Rzed.; GRO, HGO, MICH, OAX, PUE, VER (endemic). |
| *Bursera xolocotzii* Guevara; GRO, MICH (endemic). |
| ***Protium*** |
| *Protium copal* (Schltdl. & Cham.) Engl.; CAM, CHIS, GRO, HGO, OAX, PUE, QRO, QROO, SLP, TAB, TAMS, VER, YUC; useful (MATERIALS, SOCIAL USES). |
| *Protium costaricense* (Rose) Engl. |
| *Protium glabrum* (Rose) Engl.; CHIS, OAX, VER. |
| *Protium pittieri* (Rose) Engl.; OAX; Red List: VU; useful (ENVIRONMENTAL USES). |
|  |
| **Cactaceae** |
| ***Carnegiea*** |
| *Carnegiea gigantea* (Engelm.) Britton & Rose; BCN, CHIH, SON; Red List: LC; Cites: II. |
| ***Cephalocereus*** |
| *Cephalocereus apicicephalium* E.Y.Dawson; CHIS, OAX (endemic); Red List: LC; Cites: II. |
| *Cephalocereus columna-trajani* (Karw. ex Pfeiff.) K.Schum.; OAX, PUE (endemic); Red List: LC; Cites: II; banked; useful (FOOD, MATERIALS, FUELS, ENVIRONMENTAL USES). |
| *Cephalocereus euphorbioides* (Haw.) Britton & Rose; TAMS, VER (endemic); Red List: VU; Cites: II. |
| *Cephalocereus fulviceps* (F.A.C.Weber ex K.Schum.) H.E.Moore; OAX, PUE (endemic); Red List: LC; Cites: II. |
| *Cephalocereus macrocephalus* F.A.C.Weber ex K.Schum.; OAX, PUE (endemic); Cites: II; banked; useful (ANIMAL FOOD, FUELS, ENVIRONMENTAL USES). |
| *Cephalocereus mezcalaensis* Bravo; COL, GRO, JAL, MEX, MICH, MOR, OAX, PUE (endemic); Red List: LC; Cites: II; useful (FOOD, ANIMAL FOOD, FUELS, ENVIRONMENTAL USES). |
| *Cephalocereus nudus* E.Y.Dawson; COL, GRO, JAL, MICH, OAX (endemic); Red List: LC; Cites: II. |
| *Cephalocereus polylophus* (DC.) Britton & Rose; GTO, HGO, QRO, SLP, VER (endemic); Red List: VU; Cites: II. |
| *Cephalocereus sanchezmejoradae* (A.B.Lau) H.J.Tapia & S.Arias; OAX (endemic); Cites: II. |
| *Cephalocereus scoparius* (Poselg.) Britton & Rose; OAX, VER (endemic); Red List: LC; Cites: II. |
| *Cephalocereus senilis* (Haw.) Pfeiff.; GTO, HGO, QRO, SLP, TAMS, VER (endemic); Red List: EN; NOM-59: A; Cites: II. |
| *Cephalocereus tetetzo* (F.A.C.Weber ex J.M.Coult.) Diguet; OAX, PUE (endemic); Red List: LC; Cites: II; banked; useful (FOOD, ANIMAL FOOD, FUELS, ENVIRONMENTAL USES). |
| ***Escontria*** |
| *Escontria chiotilla* (F.A.C.Weber ex K.Schum.) Rose; GRO, MICH, OAX, PUE (endemic); Red List: LC; Cites: II; banked; useful (FOOD, ANIMAL FOOD, FUELS). |
| ***Lemaireocereus*** |
| *Lemaireocereus hollianus* (F.A.C.Weber ex J.M.Coult.) Britton & Rose; OAX, PUE (endemic); Red List: LC; banked. |
| ***Leuenbergeria*** |
| *Leuenbergeria lychnidiflora* (DC.) Lodé; GRO, OAX; Red List: LC. |
| ***Lophocereus*** |
| *Lophocereus gatesii* M.E.Jones; BCS (endemic). |
| *Lophocereus marginatus* (DC.) S.Arias & Terrazas; AGS, COL, CDMX, DGO, GTO, GRO, HGO, JAL, MEX, MICH, MOR, NLE, OAX, PUE, QRO, SLP, TAMS, TLAX, VER, ZAC (endemic). |
| *Lophocereus schottii* (Engelm.) Britton & Rose; BCN, BCS, SON; banked. |
| ***Mitrocereus*** |
| *Mitrocereus militaris* (Audot) Bravo; GRO, JAL, MICH (endemic); useful (FOOD, ANIMAL FOOD). |
| ***Myrtillocactus*** |
| *Myrtillocactus geometrizans* (Mart. ex Pfeiff.) Console; AGS, CDMX, DGO, GTO, GRO, HGO, JAL, MEX, MICH, MOR, NAY, NLE, OAX, PUE, QRO, SLP, SIN, TAMS, TLAX, VER, ZAC (endemic); Red List: LC; Cites: II; banked; useful (FOOD, ANIMAL FOOD, MATERIALS, FUELS, ENVIRONMENTAL USES). |
| *Myrtillocactus schenckii* (J.A.Purpus) Britton & Rose; OAX, PUE (endemic); Red List: LC; Cites: II; banked; useful (FOOD, ANIMAL FOOD, ENVIRONMENTAL USES). |
| ***Opuntia*** |
| *Opuntia auberi* Pfeiff.; CAM, CHIS, COL, GRO, HGO, JAL, MEX, MOR, OAX, PUE, QRO, SLP, VER (endemic); Cites: II. |
| *Opuntia excelsa* Sánchez-Mej.; COL, GRO, JAL, MICH, NAY, SIN (endemic); Red List: LC; NOM-59: Pr; Cites: II. |
| *Opuntia hyptiacantha* F.A.C.Weber; AGS, CDMX, GTO, HGO, JAL, MEX, NLE, OAX, PUE, QRO, SLP, TLAX, VER, ZAC (endemic); Red List: LC; Cites: II; banked; useful (FOOD). |
| *Opuntia inaperta* (Schott ex Griffiths) D.R.Hunt; CAM, CHIS, QROO, TAB, VER, YUC (endemic); Cites: II. |
| *Opuntia leucotricha* DC.; AGS, COAH, DGO, GTO, HGO, JAL, MICH, NLE, QRO, SLP, TAMS, ZAC (endemic); Red List: LC; Cites: II; banked. |
| *Opuntia pilifera* F.A.C.Weber; OAX, PUE, TLAX (endemic); Red List: LC; Cites: II; banked; useful (FOOD, ANIMAL FOOD, FUELS, ENVIRONMENTAL USES). |
| *Opuntia karwinskiana* Salm-Dyck; CHIS, COL, DGO, GRO, HGO, JAL, MEX, MICH, MOR, NAY, OAX, QRO, SLP, SIN, SON, TAMS, VER (endemic); Cites: II. |
| ***Pachycereus*** |
| *Pachycereus eichlamii* (Britton & Rose) D.R.Hunt; CHIS, YUC; Red List: DD; Cites: II. |
| *Pachycereus grandis* Rose; MEX, MICH, MOR, OAX, PUE (endemic); Red List: VU; Cites: II; banked; useful (FOOD, ANIMAL FOOD). |
| *Pachycereus pecten-aboriginum* (Engelm. ex S.Watson) Britton & Rose; BCS, CHIH, COL, DGO, GRO, JAL, MEX, MICH, NAY, OAX, PUE, SIN, SON, ZAC (endemic); Red List: LC; Cites: II; banked; useful (FOOD, ANIMAL FOOD, MEDICINES, MATERIALS, FUELS). |
| *Pachycereus pringlei* (S.Watson) Britton & Rose; BCN, BCS, DGO, SON (endemic); Red List: LC; Cites: II; banked; useful (MEDICINES). |
| *Pachycereus tepamo* Gama & S.Arias; GRO, MICH (endemic); Red List: LC; Cites: II. |
| *Pachycereus weberi* (J.M.Coult.) Backeb.; GRO, HGO, MEX, MICH, MOR, OAX, PUE, QRO, VER (endemic); Red List: LC; Cites: II; banked; useful (FOOD, ANIMAL FOOD, MATERIALS, FUELS). |
| ***Pilosocereus*** |
| *Pilosocereus alensis* (F.A.C.Weber ex Rol.-Goss.) Byles & G.D.Rowley; CHIH, COL, GRO, JAL, MEX, MICH, NAY, SIN, SON, ZAC (endemic); Red List: LC; Cites: II. |
| *Pilosocereus chrysacanthus* (F.A.C.Weber ex K.Schum.) Byles & G.D.Rowley; GRO, MEX, MICH, MOR, OAX, PUE, VER (endemic); Red List: LC; Cites: II; banked; useful (FOOD). |
| *Pilosocereus collinsii* (Britton & Rose) Byles & G.D.Rowley; OAX (endemic); Red List: LC; Cites: II. |
| *Pilosocereus gaumeri* (Britton & Rose) Backeb; CAM, QROO, YUC (endemic); Red List: LC; Cites: II. |
| *Pilosocereus leucocephalus* (Poselg.) Byles & G.D.Rowley; DGO, HGO, QRO, SLP (endemic); Red List: LC; Cites: II. |
| *Pilosocereus purpusii* (Britton & Rose) Byles & G.D.Rowley; COL, GRO, JAL, MICH, NAY, OAX, SIN, ZAC (endemic); Red List: LC; Cites: II. |
| *Pilosocereus quadricentralis* (E.Y.Dawson) Backeb.; CHIS, OAX (endemic); Red List: EN; Cites: II; banked. |
| ***Polaskia*** |
| *Polaskia chende* (Rol.-Goss.) A.C.Gibson & K.E.Horak; OAX, PUE (endemic); Red List: LC; Cites: II; banked; useful (FOOD, ANIMAL FOOD, ENVIRONMENTAL USES). |
| *Polaskia chichipe* (Rol.-Goss.) Backeb.; MICH, OAX, PUE (endemic); Red List: LC; Cites: II; banked; useful (FOOD, ANIMAL FOOD, ENVIRONMENTAL USES). |
| ***Stenocereus*** |
| *Stenocereus beneckei* (Ehrenb.) A.Berger & Buxb.; GRO, MEX, MOR, OAX, PUE (endemic); Red List: NT; Cites: II; banked. |
| *Stenocereus chacalapensis* (Bravo & T. MacDoug.) Buxb.; OAX (endemic); Red List: CR; Cites: II. |
| *Stenocereus chrysocarpus* Sánchez-Mej.; GRO, JAL, MICH (endemic); Red List: EN; Cites: II. |
| *Stenocereus dumortieri* (Scheidw.) Buxb.; AGS, COL, CDMX, GTO, GRO, HGO, JAL, MEX, MICH, MOR, NAY, OAX, PUE, QRO, SLP, VER, ZAC (endemic); Red List: LC; Cites: II; banked. |
| *Stenocereus fricii* Sánchez-Mej.; COL, GRO, JAL, MICH (endemic); Red List: LC; Cites: II. |
| *Stenocereus griseus* (Haw.) Buxb.; CHIS, GTO, GRO, HGO, JAL, MICH, MOR, NLE, OAX, PUE, QRO, SLP, TAMS, VER, YUC, ZAC; Red List: LC; Cites: II; useful (FOOD, ENVIRONMENTAL USES). |
| *Stenocereus kerberi* (K.Schum.) A.C.Gibson & K.E.Horak; COL, DGO, JAL, NAY, SIN (endemic); Red List: LC; Cites: II. |
| *Stenocereus martinezii* (J.G.Ortega) Buxb.; SIN (endemic); Red List: EN; Cites: II. |
| *Stenocereus montanus* (Britton & Rose) Buxb.; CHIH, COL, DGO, JAL, NAY, SIN, SON, ZAC (endemic); Red List: LC; Cites: II. |
| *Stenocereus pruinosus* (Otto ex Pfeiff.) Buxb.; CAM, CHIS, GTO, GRO, HGO, JAL, MEX, MICH, OAX, PUE, QRO, QROO, SLP, TAMS, VER, YUC (endemic); Red List: LC; Cites: II; banked; useful (FOOD, ANIMAL FOOD, FUELS, ENVIRONMENTAL USES). |
| *Stenocereus queretaroensis* (F.A.C.Weber ex Mathsson) Buxb.; AGS, COL, GTO, HGO, JAL, MEX, MICH, NAY, PUE, QRO, SLP, ZAC (endemic); Red List: LC; Cites: II; banked. |
| *Stenocereus quevedonis* (J.G.Ortega) Buxb.; COL, DGO, GRO, JAL, MICH, NAY, SIN (endemic); Red List: LC; Cites: II. |
| *Stenocereus thurberi* (Engelm.) Buxb.; BCN, BCS, CHIH, DGO, SIN, SON; Red List: LC; Cites: II; banked; useful (MEDICINES, SOCIAL USES). |
| *Stenocereus treleasei* (Rose) Backeb.; OAX, PUE (endemic); Red List: LC; Cites: II; banked; useful (FOOD, ANIMAL FOOD, ENVIRONMENTAL USES). |
| *Stenocereus zopilotensis* Arreola-Nava & Terrazas; (endemic); Cites: II; banked. |
|  |
| **Calophyllaceae** |
| ***Calophyllum*** |
| *Calophyllum* *brasiliense* Cambess.; CAM, CHIS, COL, GRO, JAL, MICH, NAY, OAX, PUE, QROO, TAB, VER, YUC; Red List: LC; NOM-59: A; useful (MATERIALS). |
| ***Marila*** |
| *Marila laxiflora* Rusby; VER; Red List: LC. |
|  |
| **Cannabaceae** |
| ***Aphananthe*** |
| *Aphananthe monoica* (Hemsl.) J.-F.Leroy; AGS, CHIS, CHIH, COL, DGO, GTO, GRO, HGO, JAL, MEX, MICH, MOR, NAY, NLE, OAX, PUE, QRO, SLP, SIN, SON, TAB, TAMS, VER, ZAC; Red List: LC. |
| ***Celtis*** |
| *Celtis caudata* Planch.; CHIS, COL, DGO, GTO, GRO, HGO, JAL, MEX, MICH, MOR, NLE, OAX, PUE, QRO, SLP, SIN, TAMS, VER, ZAC; Red List: LC; banked; useful (FOOD, MATERIALS). |
| *Celtis laevigata* Willd.; BCS, CHIH, COAH, DGO, GTO, MICH, NLE, QRO, SLP, SON, TAMS, VER; Red List: LC; banked. |
| *Celtis lindheimeri* Engelm. ex K.Koch; COAH, NLE (endemic); Red List: VU. |
| *Celtis trinervia* Lam.; CAM, QROO, YUC. |
| ***Lozanella*** |
| *Lozanella enantiophylla* (Donn.Sm.) Killip & C.V.Morton; CHIS, GRO, HGO, OAX, PUE, VER; banked. |
| ***Trema*** |
| *Trema micranthum* (L.) Blume; CAM, CHIS, COL, DGO, GTO, GRO, HGO, JAL, MEX, MICH, MOR, NAY, OAX, PUE, QRO, QROO, SLP, SIN, SON, TAB, TAMS, TLAX, VER, YUC, ZAC; Red List: LC; banked; useful (FOOD, ANIMAL FOOD, MEDICINES, MATERIALS, FUELS). |
|  |
| **Capparaceae** |
| ***Crateva*** |
| *Crateva tapia* L.; BCS, CAM, CHIS, COL, DGO, GRO, HGO, JAL, MEX, MICH, MOR, NAY, OAX, PUE, QRO, QROO, SLP, SIN, TAB, TAMS, VER, YUC; banked; useful (FOOD, MEDICINES, MATERIALS). |
| ***Morisonia*** |
| *Morisonia americana* L.; CHIS, COL, GRO, JAL, MICH, NAY, OAX, QRO, SLP, SIN, TAMS, VER; banked. |
| *Morisonia angustifolia* (Kunth) Christenh. & Byng; GRO, JAL, MICH, OAX (endemic). |
| *Morisonia asperifolia* (C.Presl) Christenh. & Byng; COL, GRO, JAL, MICH, NAY (endemic). |
| *Morisonia atamisquea* (Kuntze) Christenh. & Byng; BCN, BCS, SIN, SON. |
| *Morisonia calciphila* (Standl. & Steyerm.) Christenh. & Byng; CHIS. |
| *Morisonia discolor* (Donn.Sm.) Christenh. & Byng; CHIS, GRO, TAB, VER. |
| *Morisonia frondosa* (Jacq.) Christenh. & Byng; CHIS, GRO, OAX, QRO, SLP, TAB, TAMS, VER, YUC. |
| *Morisonia heydeana* (Donn.Sm.) Christenh. & Byng. |
| *Morisonia incana* (Kunth) Christenh. & Byng; CAM, CHIS, COL, GTO, GRO, HGO, JAL, MEX, MICH, NLE, OAX, PUE, QRO, QROO, SLP, TAMS, VER, YUC; banked; useful (ANIMAL FOOD, MATERIALS, FUELS). |
| *Morisonia lindeniana* (Cornejo & Iltis) Christenh. & Byng; CAM, QROO, YUC. |
| *Morisonia lundellii* (Standl.) Christenh. & Byng; CAM, CHIS, OAX, QROO. |
| *Morisonia mirifica* (Standl.) Christenh. & Byng. |
| *Morisonia mollicella* (Standl.) Christenh. & Byng; CHIS, COL, JAL, NAY, OAX, PUE, TAB, VER. |
| *Morisonia morenoi* (Cornejo & Iltis) Christenh. & Byng; MICH, OAX. |
| *Morisonia odoratissmia* (Jacq.) Christenh. & Byng; CHIS, OAX. |
| *Morisonia pachaca* (Kunth) Christenh. & Byng; CAM, GRO, QROO, YUC. |
| *Morisonia paradoxa* (Jacq.) Christenh. & Byng. |
| *Morisonia pringlei* (Briq.) Christenh. & Byng; CHIS, OAX, PUE, VER. |
| *Morisonia quintanarooensis* (Iltis & Cornejo) Christenh. & Byng; QROO (endemic). |
| *Morisonia quiriguensis* (Standl.) Christenh. & Byng; CHIS, COL, JAL, TAB, VER. |
| *Morisonia tuxtlensis* (Cornejo & Iltis) Christenh. & Byng; VER (endemic). |
| *Morisonia verrucosa* (Jacq.) Christenh. & Byng; CAM, CHIS, COL, GRO, JAL, MICH, NAY, OAX, QROO, SIN, VER. |
| ***Quadrella*** |
| *Quadrella indica* (L.) Iltis & Cornejo; CAM, CHIS, COL, GTO, GRO, JAL, MICH, NAY, OAX, QRO, QROO, SLP, SIN, VER, YUC, ZAC ; Red List: LC. |
| *Quadrella isthmensis* (Eichler) Hutch.; CAM, QROO, YUC. |
|  |
| **Caricaceae** |
| ***Carica*** |
| *Carica papaya* L.; AGS, BCS, CAM, CHIS, CHIH, COL, GRO, HGO, JAL, MEX, MICH, MOR, NAY, OAX, PUE, QRO, QROO, SLP, SIN, SON, TAB, TAMS, VER, YUC, ZAC; Red List: DD; useful (FOOD, ANIMAL FOOD, MEDICINES, POISONS, MATERIALS, SOCIAL USES). |
| ***Jacaratia*** |
| *Jacaratia mexicana* A.DC.; CAM, CHIS, COL, GRO, JAL, MEX, MICH, MOR, NAY, OAX, PUE, QROO, SIN, TAB, VER, YUC, ZAC; Red List: LC; banked; useful (FOOD). |
| ***Jarilla*** |
| *Jarilla heterophylla* (Cerv. ex La Llave) Rusby; COL, CDMX, GTO, GRO, HGO, JAL, MEX, MICH, OAX, PUE, QRO, SLP, ZAC (endemic); banked. |
| ***Vasconcellea*** |
| *Vasconcellea cauliflora* (Jacq.) A.DC.; CHIS, HGO, OAX, PUE, SIN, VER. |
|  |
| **Celastraceae** |
| ***Elaeodendron*** |
| *Elaeodendron xylocarpum* Vent. DC.; CAM, CHIS, COL, GRO, HGO, JAL, MICH, NAY, OAX, QRO, QROO, SLP, TAB, VER, YUC; banked; useful. |
| ***Euonymus*** |
| *Euonymus chiapensis* Lundell; CHIS, VER (endemic). |
| ***Gyminda*** |
| *Gyminda tonduzii* Loes.; CHIS, OAX, QROO. |
| ***Haydenoxylon*** |
| *Haydenoxylon haberianum* (Hammel) M.P.Simmons; VER. |
| ***Maytenus*** |
| *Maytenus belizensis* Standl.; CAM, QROO, VER, YUC. |
| *Maytenus chiapensis* Lundell; CHIS. |
| *Maytenus matudae* Lundell; CHIS, GRO (endemic); Red List: VU. |
| *Maytenus phyllanthoides* Benth.; BCN, BCS, CAM, COAH, HGO, JAL, NLE, PUE, QRO, QROO, SLP, SIN, SON, TAMS, VER, YUC, ZAC. |
| *Maytenus purpusii* Lundell; CHIS, VER, TAB. |
| *Maytenus schippii* Lundell; CAM, CHIS, COL, GRO, OAX, QROO, TAB, VER. |
| *Maytenus stipitata* Lundell; CHIS (endemic); Red List: VU. |
| *Maytenus tikalensis* Lundell. |
| *Maytenus wendtii* Lundell; OAX, VER (endemic). |
| ***Neopringlea*** |
| *Neopringlea viscosa* (Liebm.) Rose; CHIS, COL, GRO, JAL, MEX, MOR, OAX, PUE, QRO, SLP; banked; useful. |
| ***Wimmeria*** |
| *Wimmeria acuminata* L.O.Williams; CHIS (endemic); Red List: EN. |
| *Wimmeria bartlettii* Lundell; CHIS, HGO, MICH, OAX, PUE, QRO, SLP, TAB, VER; Red List: LC. |
| *Wimmeria concolor* Schltdl. & Cham.; CHIS, HGO, OAX, PUE, QRO, SLP, TAMS, VER; useful (MATERIALS, FUELS). |
| *Wimmeria confusa* Hemsl. |
| *Wimmeria lanceolata* Rose; COL, GRO, JAL, MEX, MICH, MOR, OAX, TLAX (endemic). |
| *Wimmeria lundelliana* Carnevali, R.Duno, J.L.Tapia & I.Ramírez; CAM, QROO (endemic). |
| *Wimmeria microphylla* Radlk.; AGS, DGO, GRO, JAL, NAY, OAX, PUE, VER, ZAC (endemic); banked; useful (ANIMAL FOOD). |
| *Wimmeria montana* Lundell; CHIS (endemic); Red List: EN. |
| *Wimmeria obtusifolia* Standl.; CAM, CHIS, QROO, VER (endemic). |
| *Wimmeria pubescens* Radlk.; CHIS, GRO, NAY, OAX, PUE, VER; useful (ANIMAL FOOD). |
| *Wimmeria serrulata* Radlk.; CHIS, GRO, MICH, MOR, OAX (endemic). |
| *Wimmeria sternii* Lundell; CHIS, OAX. |
| ***Zinowiewia*** |
| *Zinowiewia concinna* Lundell; COL, GRO, HGO, JAL, MEX, MICH, MOR, OAX, VER (endemic); NOM-59: P. |
| *Zinowiewia integerrima* (Turcz.) Turcz.; CHIS, GRO, MEX, MICH, MOR, OAX, PUE, SLP, VER. |
| *Zinowiewia rubra* Lundell; CHIS. |
|  |
| **Chloranthaceae** |
| ***Hedyosmum*** |
| *Hedyosmum mexicanum* C.Cordem.; CHIS, COL, DGO, GRO, JAL, MEX, MICH, MOR, NAY, OAX, PUE, SIN, VER; Red List: VU; banked; useful (ENVIRONMENTAL USES). |
|  |
| **Chrysobalanaceae** |
| ***Couepia*** |
| *Couepia polyandra* (Kunth) Rose; CAM, CHIS, COL, GTO, GRO, JAL, MICH, NAY, OAX, PUE, QRO, QROO, SIN, TAB, VER, YUC; useful (FOOD, SOCIAL USES). |
| ***Hirtella*** |
| *Hirtella americana* L.; CAM, CHIS, GRO, OAX, QROO, SLP, TAB, VER; Red List: LC. |
| *Hirtella triandra* Sw.; CHIS, OAX, PUE, TAB, VER. |
| ***Licania*** |
| *Licania arborea* Seem.; CHIS, GRO, JAL, MEX, MICH, MOR, OAX, PUE; NOM-59: A; useful (FOOD, MATERIALS). |
| *Licania gonzalezii* Miranda; COL, GRO, JAL, NAY (endemic). |
| *Licania hypoleuca* Benth.; CHIS, GRO, NAY, OAX, TAB, VER; Red List: LC. |
| *Licania mexicana* Lundell; SIN (endemic). |
| *Licania sparsipilis* S.F.Blake; CHIS, GRO, OAX, VER ; Red List: VU. |
| ***Moquilea*** |
| *Moquilea platypus* Hemsl.; CHIS, GTO, GRO, HGO, JAL, OAX, PUE, TAB, VER; Red List: LC. |
|  |
| **Clethraceae** |
| ***Clethra*** |
| *Clethra* × *parvifolia* Lundell; CHIS (endemic). |
| *Clethra* *alcoceri* Greenm.; CHIS, COL, GRO, HGO, JAL, MICH, OAX, PUE, TAB, VER (endemic); Red List: VU; banked. |
| *Clethra* *chiapensis* L.M.González; CHIS (endemic); Red List: EN. |
| *Clethra* *conzattiana* L.M.González; OAX (endemic); Red List: EN. |
| *Clethra* *fragrans* L.M.González & R.Delgad.; COL, JAL (endemic); Red List: EN. |
| *Clethra* *galeottiana* Briq.; GRO (endemic); Red List: LC. |
| *Clethra* *hartwegii* Britton; AGS, CHIS, CHIH, COL, DGO, GTO, GRO, JAL, MEX, MICH, MOR, NAY, OAX, QRO, SIN, SON, ZAC (endemic); Red List: LC. |
| *Clethra* *hirsutovillosa* S.Valencia & Cruz Durán; GRO (endemic); Red List: EN. |
| *Clethra* *luzmariae* L.M.González; OAX (endemic); Red List: EN. |
| *Clethra mexicana* DC.; AGS, CHIS, CHIH, COL, CDMX, DGO, GTO, GRO, HGO, JAL, MEX, MICH, MOR, NAY, OAX, PUE, QRO, SLP, SIN, SON, TAB, TAMS, VER; Red List: LC. |
| *Clethra* *occidentalis* (L.) Kuntze; CHIS, GRO, JAL, MEX, MICH, OAX, SLP, VER. |
| *Clethra* *oleoides* L.O.Williams; CHIS; Red List: VU. |
| *Clethra* *pachecoana* Standl. & Steyerm.; CHIS, OAX; Red List: VU. |
| *Clethra* *pringlei* S.Watson; CHIS, GRO, HGO, JAL, MEX, MICH, NLE, OAX, PUE, QRO, SLP, TAMS, VER (endemic); Red List: LC; banked. |
| *Clethra* *purpusii* L.M.González; CHIS, OAX (endemic); Red List: EN. |
| *Clethra* *rosei* Britton; CHIH, COL, DGO, GRO, JAL, MEX, MICH, NAY, OAX, SIN, SON, ZAC (endemic); Red List: LC. |
| *Clethra* *suaveolens* Turcz.; CHIS, OAX, QRO, SLP, VER; Red List: LC. |
| *Clethra* *tuxtlensis* L.M.González; VER (endemic); Red List: VU. |
|  |
| **Clusiaceae** |
| ***Clusia*** |
| *Clusia belizensis* Standl.; CHIS. |
| *Clusia flava* Jacq.; CAM, CHIS, OAX, QROO, TAB, TAMS, VER, YUC; Red List: LC. |
| *Clusia flavida* (Benth.) Pipoly; Red List: LC. |
| *Clusia guatemalensis* Hemsl.; CHIS, OAX, PUE, TAB, VER. |
| *Clusia lundellii* Standl.; CAM, CHIS, OAX, TAB, VER. |
| *Clusia massoniana* Lundell; CHIS, MEX, MICH, OAX; Red List: LC. |
| *Clusia minor* L.; CHIS, OAX, PUE, TAB, VER; Red List: LC. |
| *Clusia ovigera* Planch. & Triana; VER. |
| *Clusia pringlei* Lundell; COL, GRO, MICH (endemic). |
| *Clusia quadrangula* Bartlett; CHIS, OAX, VER; Red List: LC. |
| *Clusia salvinii* Donn.Sm.; CAM, CHIS, COL, DGO, GRO, JAL, MEX, MICH, MOR, NAY, OAX, QROO, SIN, TAB, TAMS, VER, YUC, ZAC; Red List: LC; useful (MEDICINES, MATERIALS, ENVIRONMENTAL USES). |
| ***Garcinia*** |
| *Garcinia intermedia* (Pittier) Hammel; CHIS, COL, GRO, JAL, MICH, NAY, OAX, PUE, QROO, TAB, VER; Red List: LC; banked; useful (FOOD, ANIMAL FOOD, MATERIALS). |
| *Garcinia macrophylla* Mart.; CHIS, JAL, OAX, TAB, VER; Red List: LC; useful (FOOD, ANIMAL FOOD, MATERIALS). |
| ***Symphonia*** |
| *Symphonia globulifera* L.f.; OAX, TAB, VER; Red List: LC; useful (MATERIALS). |
|  |
| **Combretaceae** |
| ***Conocarpus*** |
| *Conocarpus erectus* L.; BCN, BCS, CAM, CHIS, COL, GRO, JAL, MICH, NAY, OAX, PUE, QROO, SIN, SON, TAB, TAMS, VER, YUC; Red List: LC; NOM-59: A; banked; useful (FOOD, MEDICINES, MATERIALS, FUELS). |
| ***Laguncularia*** |
| *Laguncularia racemosa* (L.) C.F.Gaertn.; BCN, BCS, CAM, CHIS, COL, GRO, JAL, MICH, NAY, OAX, PUE, QROO, SLP, SIN, SON, TAB, TAMS, VER, YUC; Red List: LC; NOM-59: A; banked; useful (FUELS). |
| ***Terminalia*** |
| *Terminalia* *amazonia* (J.F.Gmel.) Exell; CAM, CHIS, OAX, QROO, TAB, VER; Red List: LC; useful (MATERIALS). |
| *Terminalia* *buceras* (L.) C.Wright; CAM, CHIS, GRO, MICH, OAX, PUE, QROO, SIN, TAB, VER, YUC. |
| *Terminalia* *lucida* Hoffmanns. ex Mart.; CHIS (endemic); useful (MATERIALS, ENVIRONMENTAL USES). |
| *Terminalia* *macrostachya* (Standl.) Alwan & Stace; CHIS, OAX, VER. |
| *Terminalia* *oblonga* (Ruiz & Pav.) Steud.; CHIS; Red List: LC; useful (ANIMAL FOOD, MATERIALS, ENVIRONMENTAL USES). |
|  |
| **Convolvulaceae** |
| ***Ipomoea*** |
| *Ipomoea* *arborescens* (Humb. & Bonpl. ex Willd.) G.Don; AGS, CHIS, CHIH, COL, DGO, GTO, GRO, HGO, JAL, MEX, MICH, MOR, NAY, OAX, PUE, QRO, SLP, SIN, SON, TAB, VER; banked; useful (ANIMAL FOOD, MEDICINES, POISONS, MATERIALS, FUELS, ENVIRONMENTAL USES). |
| *Ipomoea* *chilopsidis* Standl.; CHIH, DGO, SON (endemic). |
| *Ipomoea* *murucoides* Roem. & Schult.; AGS, CHIS, COL, CDMX, DGO, GTO, GRO, HGO, JAL, MEX, MICH, MOR, NAY, OAX, PUE, QRO, SLP, SIN, ZAC; Red List: LC; banked; useful (ANIMAL FOOD, MEDICINES, POISONS, SOCIAL USES). |
| *Ipomoea* *pauciflora* M.Martens & Galeotti; CHIS, COL, CDMX, GRO, HGO, JAL, MEX, MICH, MOR, OAX, PUE, QRO, ZAC; banked; useful (MEDICINES, POISONS, MATERIALS, FUELS, ENVIRONMENTAL USES). |
| *Ipomoea* *teotitlanica* McPherson; OAX (endemic); banked; useful. |
|  |
| **Cornaceae** |
| ***Cornus*** |
| *Cornus disciflora* Moc. & Sessé ex DC.; CHIS, CHIH, COL, CDMX, DGO, GTO, GRO, HGO, JAL, MEX, MICH, MOR, NAY, NLE, OAX, PUE, QRO, SLP, SIN, SON, TAMS, VER, ZAC; Red List: VU; banked; useful (FOOD, ANIMAL FOOD, MEDICINES, MATERIALS). |
| *Cornus excelsa* Kunth; CHIS, CHIH, COL, CDMX, DGO, GTO, GRO, HGO, JAL, MEX, MICH, MOR, NAY, NLE, OAX, PUE, QRO, SLP, SIN, TAMS, TLAX, VER; Red List: LC; banked. |
|  |
| **Crossosomataceae** |
| ***Crossosoma*** |
| *Crossosoma californicum* Nutt.; BCN. |
|  |
| **Cunoniaceae** |
| ***Weinmannia*** |
| *Weinmannia balbisiana* Kunth; CHIS, OAX; Red List: LC. |
| *Weinmannia pinnata* L.; CHIS, GRO, HGO, OAX, TAMS, VER; Red List: LC; useful (MATERIALS, FUELS). |
|  |
| **Cupressaceae** |
| ***Calocedrus*** |
| *Calocedrus decurrens* (Torr.) Florin; BCN, MEX; Red List: LC; NOM-59: A; useful (ENVIRONMENTAL USES, MATERIALS). |
| ***Hesperocyparis*** |
| *Hesperocyparis* *arizonica* (Greene) Bartel; AGS, BCN, CHIH, COAH, COL, DGO, HGO, JAL, MEX, MICH, NAY, NLE, SLP, SIN, SON, TAMS, VER, ZAC; Red List: LC; banked. |
| *Hesperocyparis* *guadalupensis* (S.Watson) Bartel; BCN; Red List: EN; NOM-59: P; banked. |
| *Hesperocyparis* *lusitanica* (Mill.) Bartel; AGS, CAM, CHIS, CHIH, COAH, COL, CDMX, DGO, GTO, GRO, HGO, JAL, MEX, MICH, MOR, NAY, NLE, OAX, PUE, QRO, QROO, SLP, SIN, SON, TAMS, TLAX, VER, YUC, ZAC; Red List: LC; NOM-59: Pr; banked; useful. |
| *Hesperocyparis* *stephensonii* (C.B.Wolf) Bartel. |
| ***Juniperus*** |
| *Juniperus* *angosturana* R.P.Adams; COAH, GTO, HGO, JAL, NLE, QRO, SLP, TAMS, VER, ZAC (endemic); Red List: VU. |
| *Juniperus* *ashei* J.Buchholz; COAH, NLE; Red List: LC. |
| *Juniperus* *blancoi* Martínez; DGO, JAL, MEX, MICH, SIN, SON, TLAX, ZAC (endemic); Red List: NT. |
| *Juniperus* *californica* Carrière; BCN; Red List: LC; banked. |
| *Juniperus* *coahuilensis* (Martínez) Gaussen ex R.P.Adams; CHIH, COAH, DGO, JAL, NLE, SON, ZAC; Red List: LC. |
| *Juniperus* *comitana* Martínez; CHIS; Red List: EN. |
| *Juniperus* *deppeana* Steud.; AGS, CHIS, CHIH, COAH, CDMX, DGO, GRO, HGO, JAL, MEX, MICH, MOR, NLE, OAX, PUE, QRO, SLP, SIN, SON, TAMS, TLAX, VER, ZAC; Red List: LC; banked; useful (ANIMAL FOOD, MEDICINES, MATERIALS, FUELS). |
| *Juniperus* *durangensis* Martínez; AGS, CHIH, DGO, JAL, SIN, SON, ZAC (endemic); Red List: LC. |
| *Juniperus* *flaccida* Schltdl.; AGS, CHIH, COAH, COL, CDMX, DGO, GTO, GRO, HGO, JAL, MEX, MICH, MOR, NAY, NLE, OAX, PUE, QRO, SLP, SIN, SON, TAMS, TLAX, VER, ZAC; Red List: LC; banked; useful (ANIMAL FOOD, MEDICINES, MATERIALS, FUELS, SOCIAL USES). |
| *Juniperus* *gamboana* Martínez; CHIS (endemic); Red List: EN. |
| *Juniperus* *jaliscana* Martínez; DGO, JAL, NAY (endemic); Red List: EN. |
| *Juniperus* *monosperma* (Engelm.) Sarg.; CHIH, COAH, DGO, GTO, HGO, NLE, QRO, SLP, SON, TAMS, ZAC; Red List: LC; useful (MATERIALS, FUELS). |
| *Juniperus* *pinchotii* Sudw.; CHIH, COAH, DGO, NLE; Red List: LC. |
| *Juniperus* *poblana* (Martínez) R.P.Adams; DGO, JAL, OAX, PUE (endemic); banked. |
| *Juniperus* *saltillensis* M.T.Hall; CHIH, COAH, DGO, NLE, SLP, ZAC; Red List: EN. |
| *Juniperus* *scopulorum* Sarg.; CHIH, COAH, SON; Red List: LC; banked; useful (ENVIRONMENTAL USES). |
| *Juniperus* *standleyi* Steyerm.; CHIS; Red List: EN. |
| ***Taxodium*** |
| *Taxodium* *distichum* var. *mexicanum* (Carrière) Gordon & Glend.; AGS, BCS, CHIS, CHIH, COAH, COL, CDMX, DGO, GTO, GRO, HGO, JAL, MEX, MICH, MOR, NAY, NLE, OAX, PUE, QRO, SLP, SIN, SON, TAB, TAMS, TLAX, VER, ZAC; Red List: LC; banked. |
|  |
| **Cyatheaceae** |
| ***Alsophila*** |
| *Alsophila* *firma* (Baker) D.S.Conant; CHIS, COL, GRO, HGO, JAL, MEX, OAX, PUE, QRO, SLP, TAMS, VER; Cites: II. |
| *Alsophila salvinii* Hook.; CHIS, OAX, VER; Cites: II. |
| ***Cyathea*** |
| *Cyathea divergens* Kunze; CHIS, GRO, HGO, OAX, PUE, VER; Cites: II. |
| *Cyathea fulva* Fée; CHIS, GRO, HGO, OAX, PUE, VER; Cites: II. |
| *Cyathea godmanii* Domin; CHIS, GRO, HGO, OAX, TAB, VER. |
| ***Dicksonia*** |
| *Dicksonia sellowiana* Hook.; CHIS, GRO, HGO, OAX, PUE, VER; Cites: II. |
| ***Sphaeropteris*** |
| *Sphaeropteris horrida* (Liebm.) R.M.Tryon; CHIS, GRO, OAX, PUE, VER; Cites: II. |
| *Sphaeropteris myosuroides* (Liebm.) R.M.Tryon; CHIS, OAX, PUE, TAB, VER. |
|  |
| **Cyrillaceae** |
| ***Cyrilla*** |
| *Cyrilla racemiflora* L.; OAX; Red List: LC; useful (ENVIRONMENTAL USES). |
|  |
| **Dichapetalaceae** |
| ***Dichapetalum*** |
| *Dichapetalum mexicanum* Prance; VER (endemic). |
| ***Tapura*** |
| *Tapura mexicana* Prance; COL, GRO, JAL, OAX (endemic). |
|  |
| **Dilleniaceae** |
| ***Curatella*** |
| *Curatella americana* L.; CAM, CHIS, COL, GRO, HGO, JAL, MEX, MICH, NAY, OAX, PUE, QROO, SIN, TAB, VER, YUC; useful (MEDICINES, MATERIALS, FUELS, ENVIRONMENTAL USES). |
|  |
| **Dipentodontaceae** |
| ***Perrottetia*** |
| *Perrottetia ovata* Hemsl.; CHIS, COL, DGO, GRO, HGO, JAL, OAX, PUE, QRO, SLP, SIN, VER; banked. |
|  |
| **Ebenaceae** |
| ***Diospyros*** |
| *Diospyros* *aequoris* Standl.; COL, DGO, JAL, MICH, NAY, OAX, SIN (endemic); banked. |
| *Diospyros* *alisu* B.Walln.; OAX (endemic). |
| *Diospyros* *anisandra* S.F.Blake; CAM, CHIS, QROO, TAB, YUC. |
| *Diospyros* *blepharophylla* Standl.; ND (endemic). |
| *Diospyros* *bumelioides* Standl.; CAM, CHIS, QROO, TAB, YUC. |
| *Diospyros* *californica* (Brandegee) I.M.Johnst.; BCS, DGO, SIN, SON. |
| *Diospyros* *conzattii* Standl.; CHIS, HGO, OAX, PUE, QRO, SLP, TAMS, VER. |
| *Diospyros* *intricata* (A.Gray) Standl.; BCS (endemic); banked; useful. |
| *Diospyros* *johnstoniana* Standl. & Steyerm.; GRO (endemic). |
| *Diospyros* *juruensis* A.C.Sm. |
| *Diospyros* *oaxacana* Standl.; CAM, COL, GRO, JAL, MEX, MICH, OAX, QROO, VER, YUC (endemic); useful (MATERIALS). |
| *Diospyros* *palmeri* Eastw.; GTO, HGO, NLE, QRO, SLP, TAMS, VER (endemic); banked. |
| *Diospyros* *rekoi* Standl.; GRO, MEXOAX (endemic). |
| *Diospyros* *rosei* Standl.; COL, GRO, JAL, MICH, NAY, SIN (endemic). |
| *Diospyros* *salicifolia* Humb. & Bonpl. ex Willd.; CAM, CHIS, COL, GRO, JAL, MEX, MICH, MOR, NAY, OAX, QROO, TAB, VER, YUC; banked. |
| *Diospyros* *sonorae* Standl.; CHIH, SIN, SON (endemic). |
| *Diospyros* *tetrasperma* Sw.; CAM, CHIS, QROO, YUC; banked; useful (FUELS). |
| *Diospyros* *texana* Scheele; CHIH, COAH, DGO, NLE, SLP, TAMS. |
| *Diospyros* *yatesiana* Standl.; CAM, CHIS, MICH, QROO, TAB, YUC. |
|  |
| **Elaeocarpaceae** |
| ***Sloanea*** |
| *Sloanea* *ampla* I.M.Johnst.; CHIS. |
| *Sloanea* *cruenta* Lundell; CHIS, GRO, OAX. |
| *Sloanea* *cuautitlanensis* Cuevas & J. L. Mendoza; (endemic). |
| *Sloanea* *guapilensis* Standl. |
| *Sloanea* *medusula* K.Schum. & Pittier; CHIS, OAX, VER; Red List: LC. |
| *Sloanea* *meianthera* Donn.Sm.; CHIS, OAX, VER. |
| *Sloanea mexicana* Standl.; COL, GRO, JAL, MICH, TAB (endemic). |
| *Sloanea* *petenensis* Standl. & Steyerm.; CHIS, OAX, TAB, VER. |
| *Sloanea* *terniflora* (Moc. & Sessé ex DC.) Standl.; CHIS, COL, DGO, GRO, JAL, NAY, OAX, TAB, VER; NOM-59: Pr. |
| *Sloanea* *tuerckheimii* Donn.Sm.; CHIS, OAX, TAB, VER; Red List: LC. |
|  |
| **Ericaceae** |
| ***Arbutus*** |
| *Arbutus* *arizonica* (A.Gray) Sarg.; AGS, CHIH, COAH, COL, DGO, GTO, JAL, MEX, MICH, NAY, NLE, QRO, SLP, SIN, SON, TAMS, ZAC; Red List: LC. |
| *Arbutus* *bicolor* S.González, M.González & P.D.Sørensen; CHIH, CDMX, DGO, GTO, HGO, MEX, MICH, PUE, QRO, SLP, SIN (endemic). |
| *Arbutus* *madrensis* S.González; DGO, JAL, NAY, SLP, SIN, ZAC (endemic). |
| *Arbutus* *tessellata* P.D.Sørensen; CHIH, COL, CDMX, DGO, GTO, HGO, JAL, MEX, MICH, NAY, QRO, SLP, SIN, TAMS, TLAX, VER, ZAC (endemic); useful (FUELS). |
| *Arbutus* *xalapensis* Kunth; AGS, BCS, CHIS, CHIH, COAH, COL, CDMX, DGO, GTO, GRO, HGO, JAL, MEX, MICH, MOR, NAY, NLE, OAX, PUE, QRO, SLP, SIN, SON, TAMS, TLAX, VER, ZAC; Red List: LR/cd; banked; useful (MEDICINES, MATERIALS, FUELS). |
| ***Bejaria*** |
| *Bejaria aestuans* Mutis; CHIS, COL, DGO, GRO, HGO, JAL, MICH, NAY, OAX, PUE, QRO, SIN, VER, ZAC; Red List: LC; banked. |
| ***Comarostaphylis*** |
| *Comarostaphylis* *discolor* (Hook.) Diggs; CHIS, COL, CDMX, DGO, GRO, HGO, JAL, MEX, MICH, MOR, OAX, PUE, QRO, SLP, TLAX, VER, ZAC; NOM-59: Pr. |
| *Comarostaphylis* *diversifolia* (Parry) Greene; BCN. |
| *Comarostaphylis* *glaucescens* (Kunth) Zucc. ex Klotzsch; AGS, COL, DGO, GTO, GRO, HGO, JAL, MEX, MICH, MOR, NAY, OAX, QRO, SLP, SIN, ZAC (endemic); banked. |
| *Comarostaphylis* *longifolia* (Benth.) Klotzsch; CHIS, COL, GRO, HGO, JAL, MEX, MICH, OAX, QRO (endemic). |
| *Comarostaphylis* *polifolia* (Kunth) Zucc. ex Klotzsch; AGS, CHIH, COAH, DGO, GTO, GRO, HGO, JAL, MEX, MICH, MOR, NAY, NLE, OAX, PUE, QRO, SLP, SIN, SON, TAMS, TLAX, VER, ZAC (endemic); banked; useful (MATERIALS, FUELS). |
| ***Lyonia*** |
| *Lyonia squamulosa* M.Martens & Galeotti; CHIS, COAH, GRO, HGO, NLE, OAX, PUE, QRO, SLP, TAMS, VER. |
| ***Vaccinium*** |
| *Vaccinium leucanthum* Schltdl.; CHIS, GTO, GRO, HGO, JAL, MEX, MICH, OAX, PUE, QRO, SLP, VER, ZAC; banked; useful (FOOD, MATERIALS). |
|  |
| **Erythroxylaceae** |
| ***Erythroxylum*** |
| *Erythroxylum* *bequaertii* Standl.; CAM, CHIS, GRO, OAX, QROO, YUC. |
| *Erythroxylum* *havanense* Jacq.; CHIS, COL, GRO, HGO, JAL, MEX, MICH, MOR, NAY, OAX, PUE, SLP, SIN, SON, VER, YUC; Red List: LC. |
| *Erythroxylum* *macrophyllum* Cav.; CHIS, OAX, TAB, VER; Red List: LC; useful (MEDICINES). |
| *Erythroxylum* *panamense* Turcz.; VER. |
| *Erythroxylum* *rotundifolium* Lunan; CAM, CHIS, CHIH, COL, DGO, GRO, JAL, MEX, MICH, MOR, NAY, OAX, PUE, QROO, SLP, VER, YUC, ZAC; banked; useful (MEDICINES, FUELS). |
|  |
| ***Euphorbia*ceae** |
| ***Acidocroton*** |
| *Acidocroton madrigalensis* Hanan-Alipi & V.W.Steinm.; TAB (endemic). |
| ***Adelia*** |
| *Adelia barbinervis* Schltdl. & Cham.; CAM, CHIS, COL, GTO, GRO, HGO, JAL, OAX, PUE, QRO, QROO, SLP, SIN, TAB, TAMS, VER, YUC; Red List: LC; useful (ANIMAL FOOD, MEDICINES, FUELS). |
| *Adelia oaxacana* (Müll.Arg.) Hemsl.; CAM, CHIS, COL, GRO, HGO, JAL, MICH, OAX, PUE, QRO, QROO, SLP, SIN, TAMS, VER, YUC (endemic); banked; useful. |
| ***Alchornea*** |
| *Alchornea latifolia* Sw.; CHIS, COL, GTO, GRO, HGO, JAL, NAY, OAX, PUE, QRO, QROO, SLP, TAB, TAMS, VER; Red List: LC; banked; useful (MATERIALS, ENVIRONMENTAL USES). |
| ***Bernardia*** |
| *Bernardia* *dodecandra* (Sessé ex Cav.) Govaerts; CAM, CHIS, GRO, HGO, OAX, PUE, QRO, QROO, SLP, TAB, TAMS, VER. |
| *Bernardia* *santanae* McVaugh; COL, JAL, MEX (endemic). |
| *Bernardia* *spongiosa* McVaugh; COL, JAL, SIN (endemic). |
| *Bernardia* *wilburii* McVaugh; COL, JAL (endemic). |
| ***Cnidoscolus*** |
| *Cnidoscolus* *autlanensis* Breckon; COL, GRO, JAL (endemic); Red List: EN; NOM-59: Pr. |
| *Cnidoscolus* *elasticus* Lundell; DGO, NAY, SIN (endemic); Red List: VU. |
| *Cnidoscolus* *multilobus* (Pax) I.M.Johnst.; CAM, CHIS, COAH, COL, DGO, GRO, HGO, JAL, MEX, MICH, MOR, NLE, OAX, PUE, QRO, QROO, SLP, SIN, TAB, TAMS, VER, YUC; Red List: LC; banked; useful (FOOD, MEDICINES). |
| *Cnidoscolus* *spinosus* Lundell; COL, JAL, MICH, NAY, ZAC (endemic); Red List: VU; banked. |
| *Cnidoscolus* *tepiquensis* (Costantin & Gallaud) Lundell; COL, JAL, NAY (endemic); Red List: VU. |
| ***Croton*** |
| *Croton* *arboreus* Millsp.; CAM, CHIS, OAX, QROO, SLP, VER, YUC; Red List: LC. |
| *Croton* *draco* Schltdl. & Cham.; CAM, CHIS, COL, DGO, GRO, HGO, JAL, MICH, MOR, NAY, OAX, PUE, QRO, QROO, SLP, SIN, TAB, TAMS, VER, YUC; Red List: LC; banked; useful (ANIMAL FOOD, MEDICINES, MATERIALS, FUELS, ENVIRONMENTAL USES, SOCIAL USES). |
| *Croton* *fragilis* Kunth. |
| *Croton* *glabellus* L.; CAM, CHIS, JAL, NAY, OAX, PUE, QROO, SLP, TAB, TAMS, VER, YUC; Red List: LC; useful (MEDICINES, FUELS, ENVIRONMENTAL USES, SOCIAL USES). |
| *Croton* *guatemalensis* Lotsy; CAM, CHIS, COL, GRO, JAL, OAX, SLP, VER, YUC; NOM-59: Pr. |
| *Croton* *niveus* Jacq.; CAM, CHIS, CHIH, COL, DGO, GTO, GRO, HGO, JAL, MEX, MICH, MOR, NAY, NLE, OAX, PUE, QRO, QROO, SLP, SIN, SON, TAMS, VER, YUC, ZAC; Red List: LC; useful (ANIMAL FOOD, MEDICINES, SOCIAL USES). |
| *Croton* *pseudoniveus* Lundell; CHIS, CHIH, COL, JAL, OAX, SIN, SON; Red List: LC. |
| *Croton* *reflexifolius* Kunth; CAM, CHIS, COL, CDMX, GTO, GRO, HGO, JAL, MEX, NAY, OAX, PUE, QRO, QROO, SLP, SIN, TAB, TAMS, VER, YUC; Red List: LC; useful (ANIMAL FOOD, MEDICINES, MATERIALS, FUELS). |
| ***Euphorbia*** |
| *Euphorbia* *calyculata* Kunth; COL, CDMX, GTO, GRO, JAL, MEX, MICH, MOR, OAX, PUE, SLP (endemic). |
| *Euphorbia* *colletioides* Benth.; CHIS, CHIH, COL, DGO, GTO, GRO, HGO, JAL, MEX, MICH, NAY, NLE, OAX, PUE, QRO, SLP, SIN, SON, TAMS, VER, YUC, ZAC; useful (MEDICINES). |
| *Euphorbia* *cotinifolia* L.; CHIS, COL, GRO, JAL, MEX, MICH, OAX, PUE, SIN, VER; Red List: LC; useful (ENVIRONMENTAL USES). |
| *Euphorbia* *pulcherrima* Willd. ex Klotzsch; CAM, CHIS, CHIH, COL, DGO, GTO, GRO, HGO, JAL, MEX, MICH, MOR, NAY, OAX, PUE, QRO, QROO, SLP, SIN, TAMS, VER, YUC; Red List: LC; useful (ENVIRONMENTAL USES). |
| *Euphorbia* *schlechtendalii* Boiss.; CAM, CHIS, COL, DGO, GRO, HGO, JAL, MEX, MICH, MOR, NAY, NLE, OAX, PUE, QRO, QROO, SLP, SIN, TAMS, VER, YUC, ZAC; banked; useful (MATERIALS). |
| *Euphorbia* *sinclairiana* Benth.; CHIS, OAX, VER; Red List: LC. |
| *Euphorbia* *tanquahuete* Sessé & Moc.; CHIS, COL, GTO, GRO, JAL, MEX, MICH, MOR, OAX, PUE, QRO, ZAC (endemic); banked. |
| ***Garcia*** |
| *Garcia* *nutans* Vahl ex Rohr; CAM, CHIS, COL, GTO, HGO, JAL, NAY, OAX, PUE, QRO, QROO, SLP, SIN, TAB, TAMS, VER, YUC; Red List: EN. |
| ***Gymnanthes*** |
| *Gymnanthes* *actinostemoides* Müll.Arg.; CHIS, COL, JAL, MEX, MICH, NAY, OAX, PUE, TAMS, VER. |
| *Gymnanthes* *longipes* Müll.Arg.; COAH, GTO, JAL, NLE, OAX, PUE, QRO, SLP, TAMS, VER (endemic). |
| *Gymnanthes* *lucida* Sw.; CAM, CHIS, GRO, HGO, QROO, TAB, VER, YUC; useful (MATERIALS). |
| *Gymnanthes* *riparia* (Schltdl.) Klotzsch; CHIS, HGO, MEX, OAX, PUE, QRO, SLP, TAMS, VER. |
| ***Hippomane*** |
| *Hippomane mancinella* L.; BCN, CAM, CHIS, COL, GRO, JAL, MEX, MICH, NAY, OAX, PUE, QROO, VER, YUC; Red List: LC; useful (POISONS). |
| ***Hura*** |
| *Hura polyandra* Baill.; CAM, CHIS, COL, DGO, GRO, HGO, JAL, MEX, MICH, MOR, NAY, OAX, PUE, QRO, QROO, SLP, SIN, SON, TAB, VER, YUC; Red List: LC; banked; useful (MEDICINES, MATERIALS). |
| ***Jatropha*** |
| *Jatropha* *alamanii* Müll.Arg.; CAM, CHIS, NAY, OAX, YUC (endemic); Red List: VU. |
| *Jatropha* *bartlettii* Wilbur; COL, JAL (endemic); Red List: EN. |
| *Jatropha* *chamelensis* Pérez-Jim.; COL, JAL, MICH, NAY (endemic); Red List: VU. |
| *Jatropha* *contrerasii* J.Jiménez Ram. & Mart.Gord.; GRO (endemic); Red List: EN. |
| *Jatropha* *conzattii* J.Jiménez Ram.; OAX (endemic); Red List: EN. |
| *Jatropha* *cordata* (Ortega) Müll.Arg.; AGS, BCN, BCS, CHIH, COL, DGO, JAL, MEX, MICH, NAY, PUE, SLP, SIN, SON, ZAC (endemic); banked; useful (MEDICINES). |
| *Jatropha* *elbae* J.Jiménez Ram.; GRO, OAX, PUE (endemic); Red List: EN. |
| *Jatropha* *galvanii* J.Jiménez Ram. & L.M.Contr.; GRO, JAL, MICH (endemic); Red List: EN. |
| *Jatropha* *gaumeri* Greenm.; CAM, CHIS, OAX, QROO, TAB, YUC; useful (MEDICINES). |
| *Jatropha* *mcvaughii* Dehgan & G.L.Webster; COL, DGO, JAL, NAY, SIN (endemic); Red List: EN. |
| *Jatropha* *ortegae* Standl.; COL, JAL, SIN (endemic); Red List: EN. |
| *Jatropha* *peltata* Sessé; COL, DGO, JAL, MICH, NAY, SIN (endemic). |
| *Jatropha* *pereziae* J.Jiménez Ram.; JAL, MICH (endemic); Red List: EN. |
| *Jatropha* *rufescens* Brandegee; PUE (endemic); Red List: VU. |
| *Jatropha* *sotoi-nunyezii* Fern.Casas & E.Martínez; TAMS, VER (endemic); Red List: EN. |
| *Jatropha* *stephani* J.Jiménez Ram. & Mart.Gord.; GRO, JAL, MICH (endemic); Red List: EN. |
| *Jatropha* *sympetala* S.F.Blake & Standl.; COL, GRO, JAL, NAY, OAX (endemic); Red List: VU. |
| ***Mabea*** |
| *Mabea occidentalis* Benth.; CHIS, COL, GRO, JAL, NAY, OAX, PUE, TAB, VER. |
| ***Manihot*** |
| *Manihot* *aesculifolia* (Kunth) Pohl; CAM, CHIS, CHIH, COL, GRO, JAL, MEX, MICH, MOR, NAY, OAX, QRO, QROO, SLP, SIN, SON, VER, YUC; Red List: LC. |
| *Manihot* *auriculata* McVaugh; JAL, NAY (endemic). |
| *Manihot* *caudata* Greenm.; AGS, CHIH, COAH, DGO, GTO, GRO, JAL, MICH, NAY, SIN, SON, ZAC (endemic). |
| *Manihot* *crassisepala* Pax & K.Hoffm.; COL, JAL, MEX, MICH, MOR (endemic). |
| *Manihot* *michaelis* McVaugh; COL, JAL, MICH, ZAC (endemic). |
| *Manihot* *oaxacana* D.J.Rogers & Appan; OAX (endemic). |
| *Manihot* *pauciflora* Brandegee; JAL, OAX, PUE (endemic); banked; useful. |
| *Manihot* *websteri* D.J.Rogers & Appan; OAX, PUE (endemic). |
| ***Omphalea*** |
| *Omphalea oleifera* Hemsl.; useful (FOOD, ENVIRONMENTAL USES, SOCIAL USES). |
| ***Pleradenophora*** |
| *Pleradenophora* *bilocularis* (S.Watson) Esser & A.L.Melo; BCN, BCS, CHIH, SON. |
| *Pleradenophora* *lottiae* (McVaugh) A.L.Melo & Esser; COL, GRO, JAL, MICH, MOR, OAX (endemic); banked. |
| *Pleradenophora* *tikalana* (Lundell) A.L.Melo & Esser; CHIH, DGO, NAY, SON (endemic). |
| *Pleradenophora* *tuerckheimiana* (Pax & K.Hoffm.) A.L.Melo & Esser; CHIS. |
| ***Sapium*** |
| *Sapium* *appendiculatum* (Müll.Arg.) Pax & K.Hoffm.; CHIS, CHIH, DGO, GRO, MICH, NAY, OAX, PUE, SIN, SON (endemic); banked. |
| *Sapium* *glandulosum* (L.) Morong; CAM, CHIS, OAX, QROO, TAB, VER, YUC; Red List: LC; useful (ANIMAL FOOD, MATERIALS). |
| *Sapium* *lateriflorum* Hemsl; CAM, CHIS, DGO, GRO, JAL, MEX, NAY, OAX, PUE, QROO, SIN, TAB, VER; Red List: LC; banked; useful (MEDICINES, POISONS). |
| *Sapium* *macrocarpum* Müll.Arg.; CAM, CHIS, COL, DGO, GTO, GRO, JAL, MEX, MICH, MOR, NAY, OAX, PUE, QRO, SLP, SIN, SON, TAMS, VER; NOM-59: A; banked. |
| ***Sebastiania*** |
| *Sebastiania glandulosa* (Sw.) Müll.Arg.; CAM, CHIS, PUE, QROO, VER, YUC. |
| *Sebastiania hintonii* Lundell; COL, GRO, JAL, MICH, MOR, NAY, OAX (endemic). |
| *Sebastiania pavoniana* (Müll.Arg.) Müll.Arg.; BCS, CHIS, CHIH, COL, DGO, GTO, GRO, HGO, JAL, MEX, MICH, MOR, NAY, OAX, PUE, QRO, SLP, SIN, SON, TAMS, VER, ZAC; Red List: LC; banked; useful (ANIMAL FOOD, MATERIALS, FUELS, ENVIRONMENTAL USES). |
| ***Tetrorchidium*** |
| *Tetrorchidium rotundatum* Standl.; CHIS, OAX, TAB, VER; NOM-59: A. |
|  |
| **Fabaceae** |
| ***Abarema*** |
| *Abarema idiopoda* (S.F.Blake) Barneby & J.W.Grimes; CAM, CHIS, OAX, TAB, VER, YUC; Red List: LC. |
| *Abarema zolleriana* (Standl. & Steyerm.) Barneby & J.W.Grimes; CHIS, OAX, VER. |
| ***Adenopodia*** |
| *Adenopodia patens* (Hook. & Arn.) J.R.Dixon ex Brenan; CHIS, COL, GRO, JAL, MICH, NAY, OAX (endemic). |
| ***Aeschynomene*** |
| *Aeschynomene amorphoides* Rose ex B.L.Rob.; COL, DGO, GRO, JAL, MICH, NAY, SIN, ZAC (endemic); banked. |
| ***Albizia*** |
| *Albizia* *adinocephala* (Donn.Sm.) Britton & Rose ex Record; CHIS, GRO, OAX; Red List: LC. |
| *Albizia* *niopoides* (Spruce ex Benth.) Burkart; CAM, CHIS, OAX, QROO, TAB, VER, YUC; Red List: LC. |
| *Albizia* *occidentalis* Brandegee; AGS, BCS, CHIS, COL, DGO, GTO, GRO, HGO, JAL, MEX, MICH, MOR, NAY, OAX, PUE, QRO, SLP, SIN, ZAC (endemic); banked; useful. |
| *Albizia* *sinaloensis* Britton & Rose; BCS, CHIH, DGO, NAY, SIN, SON (endemic). |
| *Albizia* *tomentosa* (Micheli) Standl.; CAM, CHIS, COL, GTO, GRO, HGO, JAL, MEX, MICH, NAY, OAX, PUE, QRO, QROO, SLP, SIN, TAB, VER, YUC; banked. |
| ***Andira*** |
| *Andira galeottiana* Standl.; CAM, CHIS, OAX, PUE, QROO, TAB, VER, YUC (endemic); Red List: VU; useful (MATERIALS). |
| *Andira inermis* (W.Wright) DC.; CAM, CHIS, COL, DGO, GRO, JAL, MEX, MICH, NAY, OAX, SIN, TAB, VER, YUC; Red List: LC; useful (ANIMAL FOOD, MEDICINES). |
| *Andira jaliscensis* R.T.Penn.; COL, JAL, MICH (endemic). |
| ***Apoplanesia*** |
| *Apoplanesia paniculata* C.Presl; CAM, CHIS, COL, GRO, JAL, MICH, NAY, OAX, QROO, VER, YUC; Red List: LC; banked; useful (MATERIALS, ENVIRONMENTAL USES). |
| ***Ateleia*** |
| *Ateleia* *albolutescens* Mohlenbr.; CHIS, OAX (endemic); useful (MEDICINES). |
| *Ateleia* *chiangii* J.Linares; SLP, ZAC (endemic); useful (MEDICINES). |
| *Ateleia* *chicoasensis* J.Linares; CHIS (endemic). |
| *Ateleia* *glabrata* J.Linares; CHIS, OAX (endemic). |
| *Ateleia* *gummifera* (Bertero ex DC.) D.Dietr.; Red List: EN. |
| *Ateleia* *hexandra* J.Linares; CHIS. |
| *Ateleia* *insularis* Standl.; NAY (endemic). |
| *Ateleia* *pterocarpa* Sessé & Moc. ex D.Dietr.; CAM, CHIS, COL, JAL, MICH, NAY, OAX, TAB, VER; banked; useful (ENVIRONMENTAL USES). |
| *Ateleia* *sousae* J.Linares; OAX (endemic). |
| *Ateleia* *standleyana* Mohlenbr.; NAY, SIN (endemic); banked. |
| *Ateleia* *tenorioi* J.Linares; CHIS (endemic). |
| *Ateleia* *tomentosa* Rudd; CHIS. |
| *Ateleia* *truncata* Mohlenbr.; GRO, MICH (endemic). |
| ***Balizia*** |
| *Balizia leucocalyx* (Britton & Rose) Barneby & J.W.Grimes; CHIS, TAB. |
| ***Bauhinia*** |
| *Bauhinia* *andrieuxii* Hemsl.; COL, GRO, JAL, MEX, MICH, MOR, NAY, OAX, PUE, SIN, VER (endemic). |
| *Bauhinia* *bartlettii* B.L.Turner; NLE, TAMS (endemic). |
| *Bauhinia* *chapulhuacania* Wunderlin; HGO, PUE, QRO, SLP, TAMS, VER (endemic). |
| *Bauhinia* *cookii* Rose; CHIS, COL, GRO, JAL, MICH, NAY, OAX, SIN, VER. |
| *Bauhinia* *coulteri* J.F.Macbr.; GTO, HGO, QRO, SLP, TAMS, VER (endemic). |
| *Bauhinia* *divaricata* L.; AGS, BCS, CAM, CHIS, COL, DGO, GRO, HGO, JAL, MEX, MICH, MOR, NAY, NLE, OAX, PUE, QRO, QROO, SLP, SIN, TAB, TAMS, VER, YUC; Red List: LC; useful (ANIMAL FOOD, MEDICINES, MATERIALS, ENVIRONMENTAL USES). |
| *Bauhinia* *erythrocalyx* Wunderlin; CAM, QROO, YUC. |
| *Bauhinia* *jenningsii* P.Wilson; CAM, CHIS, QROO, TAB, YUC. |
| *Bauhinia* *macranthera* Benth. ex Hemsl.; COAH, GTO, HGO, NLE, QRO, SLP, TAMS, VER (endemic). |
| *Bauhinia* *melastomatoidea* R.Torres; CHIS, OAX, TAB (endemic). |
| *Bauhinia* *pansamalana* Donn.Sm.; CHIS, TAB, VER. |
| *Bauhinia* *pauletia* Pers.; CHIS, COL, DGO, GRO, JAL, MEX, MICH, NAY, OAX, SIN, VER; Red List: LC; useful (MEDICINES). |
| *Bauhinia* *pringlei* S.Watson; CHIH, COL, GRO, JAL, MEX, MICH, NAY, SIN, SON, TAMS, ZAC (endemic). |
| *Bauhinia* *ramirezii* Reynoso; GRO, JAL, NAY (endemic). |
| *Bauhinia* *ramosissima* Benth. ex Hemsl.; CHIS, CHIH, COAH, DGO, HGO, MEX, NLE, QRO, SLP, TAMS, VER, ZAC (endemic). |
| *Bauhinia* *rubeleruziana* Donn.Sm.; CHIS, VER. |
| *Bauhinia* *seleriana* Harms; CHIS, MOR, OAX; Red List: LC. |
| *Bauhinia* *subrotundifolia* Cav.; COL, GRO, JAL, MEX, MICH, OAX (endemic); useful (MEDICINES). |
| *Bauhinia* *ungulata* L.; CAM, CHIS, CHIH, COL, DGO, GRO, HGO, JAL, MEX, MICH, NAY, OAX, QROO, SIN, TAB, VER, YUC; Red List: LC; banked; useful (MEDICINES). |
| *Bauhinia* *wunderlinii* R.Torres; CHIS, OAX, VER. |
| ***Brongniartia*** |
| *Brongniartia* *alamosana* Rydb.; CHIH, JAL, MICH, OAX, SIN, SON (endemic). |
| *Brongniartia* *cuneata* L.B.Sm. & B.G.Schub.; GRO, MEX (endemic). |
| *Brongniartia* *glabrata* Hook. & Arn.; CHIS, COL, GRO, HGO, JAL, NAY, OAX, QRO, SLP, SIN (endemic). |
| *Brongniartia* *guerrerensis* J.Jiménez Ram. & J.L.Contr.; GRO (endemic); Red List: EN. |
| *Brongniartia* *guiengolensis* O.Dorado & L.Torres-Colín; OAX (endemic). |
| *Brongniartia* *montalvoana* Dorado & D.M.Arias; GRO, MOR, PUE (endemic). |
| *Brongniartia* *pacifica* McVaugh; COL, JAL, MICH (endemic). |
| *Brongniartia* *proteranthera* L.B.Sm. & B.G.Schub.; GRO, MICH (endemic). |
| *Brongniartia* *suberea* Rose; GTO, GRO, OAX, PUE (endemic). |
| *Brongniartia* *trifoliata* Brandegee; BCS (endemic). |
| ***Caesalpinia*** |
| *Caesalpinia pulcherrima* (L.) Sw.; Red List: LC; banked; useful (MEDICINES, POISONS, ENVIRONMENTAL USES). |
| ***Calliandra*** |
| *Calliandra* *belizensis* (Britton & Rose) Standl.; CAM, QROO, YUC. |
| *Calliandra* *bijuga* Rose; CHIS, COL, GRO, JAL, MEX, MICH, NAY, OAX, VER (endemic). |
| *Calliandra* *caeciliae* Harms; CHIS, COL, GRO, JAL, MEX, MICH, MOR, NAY, OAX, PUE. |
| *Calliandra* *erythrocephala* H.M.Hern. & M.Sousa; GRO, OAX (endemic). |
| *Calliandra* *houstoniana* var. *calothyrsus* (Meisn.) Barneby; CAM, CHIS, COL, DGO, GRO, JAL, MICH, NAY, OAX, SIN, VER; banked; useful (ANIMAL FOOD, MATERIALS, FUELS). |
| *Calliandra* *laevis* Rose; COL, JAL, MICH, NAY, SIN, ZAC (endemic). |
| *Calliandra* *magdalenae* (Bertero ex DC.) Benth.; CHIS, GRO, MICH, NAY, OAX, VER; Red List: LC. |
| *Calliandra* *ricoana* H.M.Hern. & R.Duno; CHIS (endemic). |
| *Calliandra* *rubescens* (M.Martens & Galeotti) Standl.; CHIS, MICH, OAX, TAB, VER (endemic); Red List: LC. |
| *Calliandra* *tehuantepecensis* (L.Rico & M.Sousa) E.R.Souza & L.P.Queiroz; OAX (endemic). |
| *Calliandra* *trinervia* var. *arborea* (Standl.) Barneby; CHIS, GRO, OAX; Red List: LC. |
| ***Cassia*** |
| *Cassia grandis* L.f.; CAM, CHIS, COL, GRO, MEX, MICH, OAX, QROO, TAB, VER, YUC; Red List: LC; useful (ANIMAL FOOD, MEDICINES, ENVIRONMENTAL USES). |
| *Cassia hintonii* Sandwith; COL, GRO, JAL, MEX, MICH (endemic). |
| *Cassia moschata* Kunth; CHIS, OAX, TAB, VER; Red List: LC. |
| ***Cenostigma*** |
| *Cenostigma eriostachys* (Benth.) Gagnon & G.P.Lewis; CHIS, COL, DGO, GRO, JAL, MICH, NAY, OAX, SIN; banked; useful. |
| *Cenostigma gaumeri* (Greenm.) Gagnon & G.P.Lewis; useful (ANIMAL FOOD, MEDICINES, MATERIALS, FUELS, ENVIRONMENTAL USES). |
| ***Cercis*** |
| *Cercis canadensis* L.; CHIH, COAH, HGO, NLE, PUE, QRO, SLP, TAMS, VER; Red List: LR/lc; banked. |
| ***Chloroleucon*** |
| *Chloroleucon mangense* (Jacq.) Britton & Rose; BCS, CAM, CHIS, CHIH, COL, DGO, GRO, JAL, MICH, NAY, OAX, QROO, SIN, SON, VER, YUC; Red List: LC; banked. |
| ***Clitoria*** |
| *Clitoria glaberrima* Pittier; CHIS, TAB; Red List: LC. |
| ***Cojoba*** |
| *Cojoba* *arborea* (L.) Britton & Rose; CAM, CHIS, GRO, HGO, JAL, MICH, MOR, NLE, OAX, PUE, QRO, QROO, SLP, SIN, TAB, TAMS, VER; Red List: LC; useful (MATERIALS, FUELS, ENVIRONMENTAL USES). |
| *Cojoba* *escuintlensis* (Lundell) L.Rico; CHIS, OAX (endemic). |
| *Cojoba* *graciliflora* (S.F.Blake) Britton & Rose; CAM, CHIS, OAX, QROO, TAB, VER; Red List: LC. |
| *Cojoba* *rufescens* (Benth.) Britton & Rose; Red List: LC. |
| *Cojoba* *sophorocarpa* (Benth.) Britton & Rose; CHIS, TAB, VER; Red List: LC. |
| ***Conzattia*** |
| *Conzattia multiflora* (B.L.Rob.) Standl.; AGS, BCS, CHIH, COL, DGO, GTO, GRO, JAL, MEX, MICH, MOR, NAY, OAX, PUE, QRO, SLP, SIN, SON, TAB, TAMS, VER, ZAC (endemic); banked; useful (FOOD, FUELS). |
| ***Coulteria*** |
| *Coulteria* *cubensis* (Greenm.) Sotuyo & G.P.Lewis; banked. |
| *Coulteria* *glabra* (Britton & Rose) J.L.Contr., Sotuyo & G.P.Lewis; COL, GRO, JAL, MICH, OAX, SIN (endemic). |
| *Coulteria* *mollis* Kunth; CAM, CHIS, QROO, YUC; useful (ENVIRONMENTAL USES). |
| *Coulteria* *pumila* (Britton & Rose) Sotuyo & G.P.Lewis; SON (endemic). |
| *Coulteria* *velutina* (Britton & Rose) Sotuyo & G.P.Lewis; CHIS, COL, GRO, JAL, MICH, NAY, OAX, PUE, VER, YUC; banked; useful (ANIMAL FOOD, MATERIALS, FUELS). |
| *Coulteria* *pringlei* (Britton & Rose) J.L. Contr., Sotuyo & G.P. Lewis; GTO, HGO, OAX, PUE, QRO, SLP, TAMS (endemic); banked. |
| ***Coursetia*** |
| *Coursetia* *glandulosa* A.Gray; AGS, BCN, BCS, CAM, CHIH, COL, DGO, GRO, HGO, JAL, MEX, MICH, MOR, NAY, OAX, PUE, QRO, SLP, SIN, SON, ZAC; Red List: LC; banked; useful (ANIMAL FOOD, MEDICINES). |
| *Coursetia* *guatemalensis* Rudd; CHIS. |
| *Coursetia* *madrensis* Micheli; GRO, MEX, MICH, MOR, OAX, PUE (endemic). |
| *Coursetia* *mollis* B.L.Rob. & Greenm.; COL, DGO, GRO, JAL, MEX, MICH, NAY, OAX, SIN, ZAC (endemic). |
| *Coursetia* *oaxacensis* M.Sousa & Rudd; GRO, OAX (endemic). |
| *Coursetia* *paniculata* M.Sousa & Lavin; OAX (endemic). |
| *Coursetia* *planipetiolata* Micheli; GRO, OAX (endemic); Red List: EN. |
| *Coursetia* *polyphylla* Brandegee; GRO, OAX, VER. |
| *Coursetia* *robinioides* M.Sousa & Lavin; OAX (endemic). |
| ***Cynometra*** |
| *Cynometra hemitomophylla* (Donn.Sm.) Rose; CAM, VER. |
| *Cynometra oaxacana* Brandegee; CAM, CHIS, COL, GRO, JAL, MICH, OAX, TAB, VER. |
| *Cynometra retusa* Britton & Rose; CAM, CHIS, OAX, TAB, VER; Red List: LC. |
| ***Dalbergia*** |
| *Dalbergia* *calderonii* Standl.; CHIS, OAX; Cites: II. |
| *Dalbergia* *calycina* Benth.; CHIS, MEX, MICH, OAX; Red List: LC; Cites: II. |
| *Dalbergia* *congestiflora* Pittier; CHIS, COL, GRO, JAL, MEX, MICH, MOR, OAX, PUE, QRO, SLP (endemic); NOM-59: P; banked. |
| *Dalbergia* *ecastaphyllum* (L.) Taub.; TAB, VER; Cites: II. |
| *Dalbergia* *glabra* (Mill.) Standl.; CAM, CHIS, GRO, HGO, MEX, MICH, MOR, OAX, QRO, QROO, SLP, TAB, VER, YUC; Cites: II; banked. |
| *Dalbergia* *glomerata* Hemsl.; CHIS, GRO, HGO, MEX, MICH, MOR, OAX, TAB, VER (endemic); Red List: VU; Cites: II; useful (MATERIALS). |
| *Dalbergia* *granadillo* Pittier; CHIS, COL, GRO, JAL, MEX, MICH, NAY, OAX, VER (endemic); Cites: II. |
| *Dalbergia* *longepedunculata* J.Linares & M.Sousa; CHIS, OAX; Cites: II. |
| *Dalbergia* *luteola* J.Linares & M.Sousa; CHIS; Cites: II. |
| *Dalbergia* *melanocardium* Pittier; CHIS, VER; Cites: II. |
| *Dalbergia* *modesta* J.Linares & M.Sousa; CHIS, OAX (endemic); Cites: II. |
| *Dalbergia* *palo-escrito* Rzed. & Guridi-Gómez; HGO, QRO, SLP, VER (endemic); Cites: II. |
| *Dalbergia* *retusa* Hemsl.; CHIS; Red List: VU; Cites: II. |
| *Dalbergia* *rhachiflexa* J.Linares & M.Sousa; GRO, MICH (endemic); Cites: II. |
| *Dalbergia* *ruddiae* J.Linares & M.Sousa; Cites: II. |
| *Dalbergia* *stevensonii* Standl.; CHIS; Cites: II. |
| *Dalbergia* *tilarana* N.Zamora; Cites: II. |
| *Dalbergia* *tucurensis* Donn.Sm.; Cites: II. |
| ***Dalea*** |
| *Dalea schiblii* R.Medina & M.Sousa; OAX (endemic). |
| ***Dermatophyllum*** |
| *Dermatophyllum secundiflorum* (Ortega) Gandhi & Reveal; CHIH, COAH, DGO, GTO, HGO, NLE, OAX, PUE, QRO, SLP, TAMS, VER, ZAC; banked. |
| ***Dialium*** |
| *Dialium guianense* (Aubl.) Sandwith; CAM, CHIS, OAX, TAB, VER; Red List: LC; banked; useful (FOOD, ANIMAL FOOD, MEDICINES, MATERIALS). |
| ***Diphysa*** |
| *Diphysa* *americana* (Mill.) M.Sousa; CAM, CHIS, COL, GRO, HGO, JAL, MEX, MICH, MOR, NLE, OAX, PUE, QRO, QROO, SLP, TAB, TAMS, VER, YUC; Red List: LC; banked. |
| *Diphysa* *carthagenensis* Jacq.; CAM, CHIS, GRO, MEX, MICH, MOR, OAX, PUE, QROO, TAB, VER, YUC; Red List: LC; banked; useful (MEDICINES). |
| *Diphysa* *floribunda* Peyr.; CHIS, COL, GRO, JAL, MEX, MICH, MOR, OAX, PUE, VER; Red List: LC; useful (ANIMAL FOOD, MATERIALS). |
| *Diphysa* *humilis* Oerst.; CHIS, MOR, OAX, QROO, VER. |
| *Diphysa* *macrocarpa* Standl.; GRO, HGO, JAL, MEX, MICH, OAX, QRO, SLP, SIN, VER (endemic). |
| *Diphysa* *occidentalis* Rose; BCS, CHIH, COL, DGO, GRO, JAL, MICH, NAY, NLE, SIN, SON (endemic); Red List: LC; banked. |
| *Diphysa* *ormocarpoides* (Rudd) M.Sousa & R.Antonio; CHIS, GRO, MICH, OAX, PUE (endemic). |
| *Diphysa* *paucifoliolata* R.Antonio & M.Sousa; CAM, QROO. |
| *Diphysa* *puberulenta* Rydb.; AGS, CHIS, CHIH, COL, DGO, GRO, JAL, MEX, MICH, MOR, NAY, OAX, SIN, ZAC (endemic). |
| *Diphysa punctata* Rydb.; MOR, OAX, PUE (endemic). |
| *Diphysa* *spinosa* Rydb.; CHIS, MOR, OAX, PUE. |
| *Diphysa* *suberosa* S.Watson; AGS, CHIH, COL, DGO, GTO, GRO, HGO, JAL, MEX, MICH, MOR, NAY, OAX, PUE, QRO, SLP, SIN, SON, TAB, VER, ZAC (endemic); banked; useful. |
| *Diphysa* *villosa* Rydb.; JAL, MEX, MICH, MOR, OAX, PUE (endemic). |
| *Diphysa* *yucatanensis* Hanan-Alipi & M.Sousa; CAM, CHIS, QROO, TAB, YUC. |
| ***Dussia*** |
| *Dussia cuscatlanica* (Standl.) Standl. & Steyerm.; CHIS. |
| *Dussia mexicana* (Standl.) Harms; CHIS, OAX, PUE, VER. |
| ***Ebenopsis*** |
| *Ebenopsis ebano* (Berland.) Barneby & J.W.Grimes; COAH, DGO, JAL, NLE, QRO, QROO, SLP, SIN, TAMS, VER, YUC, ZAC; Red List: LC; banked. |
| ***Enterolobium*** |
| *Enterolobium cyclocarpum* (Jacq.) Griseb.; BCS, CAM, CHIS, COL, DGO, GRO, HGO, JAL, MEX, MICH, MOR, NAY, OAX, PUE, QRO, QROO, SLP, SIN, SON, TAB, TAMS, VER, YUC, ZAC; Red List: LC; banked; useful (ANIMAL FOOD, MATERIALS, FUELS). |
| *Enterolobium schomburgkii* (Benth.) Benth.; CHIS, OAX, TAB, VER; Red List: LC; NOM-59: A. |
| ***Erythrina*** |
| *Erythrina* *americana* Mill.; CAM, CHIS, CHIH, COL, CDMX, GTO, GRO, HGO, JAL, MEX, MICH, MOR, NLE, OAX, PUE, QRO, QROO, SLP, SIN, TAB, TAMS, TLAX, VER, YUC, ZAC (endemic); banked; useful (FOOD, MEDICINES, ENVIRONMENTAL USES). |
| *Erythrina* *berenices* Krukoff & Barneby; VER (endemic). |
| *Erythrina* *berteroana* Urb.; CAM, CHIS, OAX, TAB, VER; useful (FOOD, MEDICINES, POISONS, ENVIRONMENTAL USES). |
| *Erythrina* *breviflora* Moc. & Sessé ex DC.; COL, CDMX, GTO, GRO, HGO, JAL, MEX, MICH, MOR, OAX, PUE (endemic). |
| *Erythrina* *caribaea* Krukoff & Barneby; CAM, CHIS, OAX, PUE, TAB, VER. |
| *Erythrina* *chiapasana* Krukoff; CHIS, OAX, VER (endemic). |
| *Erythrina* *flabelliformis* Kearney; AGS, BCS, CHIH, COL, DGO, HGO, JAL, MEX, MICH, MOR, NLE, PUE, QRO, SLP, SIN, SON, TAMS, VER, ZAC; Red List: LC; banked; useful (ANIMAL FOOD, MEDICINES). |
| *Erythrina* *florenciae* Krukoff & Barneby; CHIS, OAX (endemic). |
| *Erythrina* *folkersii* Krukoff & Moldenke; CHIS, GRO, OAX, TAB, VER; Red List: LC; banked; useful (FOOD, ENVIRONMENTAL USES). |
| *Erythrina* *goldmanii* Standl.; CHIS, OAX (endemic). |
| *Erythrina* *herbacea* L.; CHIS, HGO, JAL, MICH, NLE, OAX, PUE, QRO, SLP, TAB, TAMS, VER; Red List: LC; useful (ENVIRONMENTAL USES). |
| *Erythrina* *lanata* Rose; CHIS, COL, DGO, GTO, GRO, JAL, MEX, MICH, MOR, NAY, OAX, SIN, VER, ZAC (endemic). |
| *Erythrina* *macrophylla* DC.; CHIS, TAB, VER. |
| *Erythrina mexicana* Krukoff; CHIS, GRO, MEX, OAX, PUE, SLP, TAB, VER; Red List: LC. |
| *Erythrina* *oaxacana* (Krukoff) Barneby; OAX (endemic). |
| *Erythrina* *oliviae* Krukoff; CHIS, MICH, MOR, OAX, PUE (endemic). |
| *Erythrina* *pudica* Krukoff & Barneby; CHIS (endemic). |
| *Erythrina* *standleyana* Krukoff; CAM, CHIS, HGO, OAX, PUE, QRO, QROO, SLP, TAB, TAMS, VER, YUC; Red List: LC; banked. |
| *Erythrina* *tajumulcensis* Krukoff & Barneby; CHIS. |
| *Erythrina* *tuxtlana* Krukoff & Barneby; CHIS, OAX, VER (endemic); Red List: VU. |
| ***Erythrostemon*** |
| *Erythrostemon* *acapulcensis* (Standl.) Gagnon & G.P.Lewis; (endemic). |
| *Erythrostemon* *caladenia* (Standl.) Gagnon & G.P.Lewis; (endemic). |
| *Erythrostemon* *coccineus* (G.P.Lewis & J.L.Contr.) Gagnon & G.P.Lewis; OAX (endemic). |
| *Erythrostemon* *epifanioi* (J.L.Contr.) Gagnon & G.P.Lewis; GRO (endemic). |
| *Erythrostemon* *exostemma* (Moc. & Sessé ex DC.) Gagnon & G.P.Lewis; SLP, TAMS, VER. |
| *Erythrostemon* *hintonii* (Sandwith) Gagnon & G.P.Lewis; GRO, MICH, OAX, PUE (endemic). |
| *Erythrostemon* *hughesii* (G.P.Lewis) Gagnon & G.P.Lewis; JAL, OAX (endemic). |
| *Erythrostemon* *macvaughii* (J.L.Contr. & G.P.Lewis) Gagnon & G.P.Lewis; GRO, MICH (endemic). |
| *Erythrostemon* *melanadenius* (Rose) Gagnon & G.P.Lewis; (endemic); banked; useful (ANIMAL FOOD, FUELS). |
| *Erythrostemon* *mexicanus* (A.Gray) Gagnon & G.P.Lewis; useful (MEDICINES). |
| *Erythrostemon* *nelsonii* (Britton & Rose) Gagnon & G.P.Lewis; GRO, OAX (endemic). |
| *Erythrostemon* *oyamae* (Sotuyo & G.P.Lewis) Gagnon & G.P.Lewis; GRO, OAX, PUE. |
| *Erythrostemon* *palmeri* (S.Watson) Gagnon & G.P.Lewis; (endemic). |
| *Erythrostemon* *robinsonianus* (Britton & Rose) Gagnon & G.P.Lewis; (endemic). |
| *Erythrostemon* *yucatanensis* (Greenm.) Gagnon & G.P.Lewis; banked. |
| ***Eysenhardtia*** |
| *Eysenhardtia* *adenostylis* Baill.; CHIS, OAX. |
| *Eysenhardtia* *byei* Cruz Durán & M.Sousa; CHIH, SON (endemic). |
| *Eysenhardtia* *officinalis* R.Cruz & M.Sousa; HGO, SLP, TAMS, VER (endemic). |
| *Eysenhardtia* *orthocarpa* (A.Gray) S.Watson; CHIH, DGO, JAL, SIN, SON; Red List: LC. |
| *Eysenhardtia* *platycarpa* Pennell & Saff.; CHIS, COL, DGO, GTO, GRO, JAL, MEX, MICH, NAY, OAX, PUE, QRO, SIN, TAMS (endemic). |
| *Eysenhardtia* *polystachya* (Ortega) Sarg.; AGS, CHIS, CHIH, COAH, COL, CDMX, DGO, GTO, GRO, HGO, JAL, MEX, MICH, MOR, NAY, NLE, OAX, PUE, QRO, SLP, SIN, SON, TAMS, TLAX, VER, ZAC; Red List: LC; banked; useful (ANIMAL FOOD, MEDICINES, MATERIALS, FUELS). |
| *Eysenhardtia* *punctata* Pennell; AGS, CHIH, COL, DGO, GTO, GRO, HGO, JAL, MEX, MICH, NAY, OAX, PUE, QRO, SON, ZAC (endemic); banked. |
| *Eysenhardtia* *subcoriacea* Pennell; HGO, OAX, PUE, QRO, VER (endemic). |
| *Eysenhardtia* *texana* Scheele; CHIH, COAH, DGO, HGO, NLE, QRO, SLP, TAMS, VER; Red List: LC. |
| ***Gleditsia*** |
| *Gleditsia aquatica* Marshall; TAMS; Red List: LC; useful (ENVIRONMENTAL USES). |
| *Gleditsia triacanthos* L.; COAH, NLE, SON, TAMS; Red List: LC. |
| ***Gliricidia*** |
| *Gliricidia maculata* (Kunth) Steud.; CAM, CHIS, QROO, TAB, YUC; banked; useful (MATERIALS, ENVIRONMENTAL USES). |
| *Gliricidia robusta* (M.Sousa & Lavin) Lavin; CHIS (endemic). |
| *Gliricidia sepium* (Jacq.) Steud.; CAM, CHIS, COL, DGO, GRO, HGO, JAL, MEX, MICH, MOR, NAY, OAX, PUE, QRO, QROO, SLP, SIN, SON, TAB, TAMS, VER, YUC, ZAC; banked; useful (FOOD, ANIMAL FOOD, MEDICINES, POISONS, MATERIALS, FUELS, ENVIRONMENTAL USES, SOCIAL USES). |
| ***Haematoxylum*** |
| *Haematoxylum* *brasiletto* H.Karst.; BCS, CAM, CHIS, CHIH, COL, DGO, GRO, JAL, MEX, MICH, MOR, NAY, OAX, PUE, QROO, SIN, SON, TAB, TAMS, VER, YUC, ZAC; Red List: LC; banked; useful (MEDICINES, MATERIALS). |
| *Haematoxylum* *calakmulense* Cruz Durán & M.Sousa; CAM, QROO, YUC (endemic). |
| *Haematoxylum* *campechianum* L.; CAM, CHIS, QROO, TAB, VER, YUC; Red List: LC; banked; useful (MEDICINES, MATERIALS). |
| *Haematoxylum* *sousanum* Cruz Durán & J.Jiménez Ram.; GRO, OAX, PUE (endemic). |
| ***Harpalyce*** |
| *Harpalyce* *arborescens* A.Gray; GTO, GRO, HGO, OAX, QRO, QROO, SLP, TAB, TAMS, VER, YUC (endemic); Red List: LC. |
| *Harpalyce mexicana* Rose; JAL, NAY, ZAC (endemic). |
| *Harpalyce* *rupicola* Donn.Sm.; CAM, QROO, TAB, YUC. |
| *Harpalyce* *sousae* Arroyo; GRO, MEX, OAX, PUE (endemic). |
| *Harpalyce* *torresii* São-Mateus & M.Sousa. |
| *Harpalyce* *yucatanense* Miranda ex São-Mateus & M.Sousa; (endemic). |
| ***Havardia*** |
| *Havardia* *acatlensis* (Benth.) Britton & Rose; (endemic); banked; useful. |
| *Havardia* *albicans* (Kunth) Britton & Rose; CAM, CHIS, GRO, QROO, TAB, VER, YUC; banked; useful (FUELS). |
| *Havardia* *campylacantha* (L.Rico & M.Sousa) Barneby & J.W.Grimes; GRO, JAL, MICH, OAX; Red List: LC; banked. |
| *Havardia mexicana* (Rose) Britton & Rose; BCS, CHIH, SIN, SON (endemic); banked; useful. |
| *Havardia* *pallens* (Benth.) Britton & Rose; BCS, CHIS, CHIH, COAH, DGO, GTO, HGO, MEX, MICH, MOR, NLE, OAX, PUE, QRO, QROO, SLP, SIN, SON, TAMS, VER, YUC; Red List: LC; banked; useful. |
| *Havardia* *sonorae* (S.Watson) Britton & Rose; BCS, DGO, SIN, SON (endemic); banked. |
| ***Hesperothamnus*** |
| *Hesperothamnus pentaphyllus* (Harms) Harms; OAX, PUE (endemic). |
| ***Heteroflorum*** |
| *Heteroflorum sclerocarpum* M.Sousa; GRO, MICH, OAX (endemic). |
| ***Hymenaea*** |
| *Hymenaea courbaril* L.; CAM, CHIS, CHIH, COL, GRO, JAL, MEX, MICH, MOR, NAY, OAX, SIN, TAB, VER; Red List: LC; banked; useful (FOOD, ANIMAL FOOD, MEDICINES, MATERIALS, FUELS, SOCIAL USES). |
| ***Inga*** |
| *Inga* × *andersonii* McVaugh; JAL, NAY (endemic); Red List: VU. |
| *Inga* *acrocephala* Steud.; CHIS, OAX, VER; useful (FOOD). |
| *Inga* *affinis* DC.; CAM, CHIS, OAX, SLP, TAB, TAMS, VER. |
| *Inga* *alba* (Sw.) Willd.; CHIS, OAX, VER. |
| *Inga* *appendiculata* M.Sousa; VER (endemic). |
| *Inga* *barbourii* Standl.; CHIS, OAX, VER. |
| *Inga* *belizensis* Standl.; CHIS, TAB. |
| *Inga* *cabrerae* M.Sousa; CHIS. |
| *Inga* *calcicola* M.Sousa; OAX (endemic); Red List: VU. |
| *Inga* *calderonii* Standl.; CHIS. |
| *Inga* *chiapensis* Miranda ex M.Sousa; CHIS, VER (endemic); Red List: VU. |
| *Inga* *colimana* Padilla, Cuevas & Solís; COL (endemic). |
| *Inga* *dasycarpa* M.Sousa; CHIS. |
| *Inga* *densiflora* Benth.; CHIS; Red List: LC; useful (FOOD, ANIMAL FOOD). |
| *Inga* *flexuosa* Schltdl.; CHIS, COL, DGO, GRO, JAL, MEX, MICH, MOR, NAY, OAX, PUE, QRO, SLP, SIN, VER. |
| *Inga* *huastecana* M.Sousa; HGO, PUE, QRO, SLP, VER (endemic). |
| *Inga* *inicuil* Schltdl. & Cham. ex G.Don; CHIS, MEX, MICH, MOR, OAX, PUE, QROO, TAB, VER, YUC; Red List: LC; useful (FOOD, ANIMAL FOOD, MEDICINES, MATERIALS, FUELS, ENVIRONMENTAL USES). |
| *Inga* *ismaelis* M.Sousa; OAX, VER (endemic); Red List: VU. |
| *Inga* *lactifera* M.Sousa; CHIS, OAX, VER. |
| *Inga* *lacustris* M.Sousa; VER (endemic); Red List: EN. |
| *Inga* *laevigata* M.Martens & Galeotti; OAX, VER; useful (FOOD). |
| *Inga* *laurina* (Sw.) Willd.; CHIS, COL, GRO, JAL, MICH, NAY, OAX, SIN, TAB, VER; Red List: LC. |
| *Inga* *marginata* Willd.; CHIS, VER; Red List: LC. |
| *Inga mexicana* (T.D.Penn.) M.Sousa; CHIS, OAX, PUE, TAB, VER. |
| *Inga* *nobilis* Willd.; CHIS, OAX, VER; useful (FOOD, MEDICINES, MATERIALS, FUELS). |
| *Inga* *oerstediana* Benth.; CHIS, GRO, HGO, OAX, PUE, QRO, SLP, TAB, VER; Red List: LC; banked; useful (FOOD, MATERIALS, ENVIRONMENTAL USES). |
| *Inga* *paterno* Harms; CHIS, GRO, OAX, PUE, QROO, TAB, VER; Red List: LC; useful (ENVIRONMENTAL USES). |
| *Inga* *pinetorum* Pittier; CHIS, OAX, TAB, VER; Red List: LC. |
| *Inga* *punctata* Willd.; CAM, CHIS, GRO, HGO, OAX, PUE, TAB, VER; Red List: LC; useful (FOOD, ANIMAL FOOD). |
| *Inga* *sapindoides* Willd.; CHIS, GRO, NAY, OAX, PUE, TAB, VER; Red List: LC. |
| *Inga* *sinacae* M.Sousa & Ibarra-Manr.; CHIS, OAX, TAB, VER (endemic); Red List: EN. |
| *Inga* *spectabilis* (Vahl) Willd.; OAX, TAB; Red List: LC; useful (FOOD, MATERIALS). |
| *Inga* *thibaudiana* DC.; CHIS; useful (FOOD, ANIMAL FOOD, ENVIRONMENTAL USES). |
| *Inga* *vera* Willd.; CAM, CHIS, COAH, COL, DGO, GRO, HGO, JAL, MEX, MICH, MOR, NAY, NLE, OAX, PUE, QRO, QROO, SLP, SIN, TAB, TAMS, VER; Red List: LC; useful (FOOD, ANIMAL FOOD, MATERIALS, FUELS, ENVIRONMENTAL USES). |
| *Inga* *xalapensis* Benth.; CHIS, OAX, PUE, QRO, SLP, VER (endemic). |
| ***Lachesiodendron*** |
| *Lachesiodendron viridiflorum* (Kunth) P.G.Ribeiro, L.P.Queiroz & Luckow; CAM, CHIS, GRO, OAX, PUE, QROO. |
| ***Lecointea*** |
| *Lecointea amazonica* Ducke; CHIS; Red List: LC. |
| ***Lennea*** |
| *Lennea melanocarpa* (Schltdl.) Vatke ex Harms; CAM, OAX, PUE, QROO, VER; useful (ANIMAL FOOD, ENVIRONMENTAL USES, SOCIAL USES). |
| *Lennea modesta* (Standl. & Steyerm.) Standl. & Steyerm.; CHIS, OAX, TAB, VER. |
| *Lennea viridiflora* Seem.; CHIS, COL, JAL, MICH, OAX, VER; Red List: VU. |
| ***Leptolobium*** |
| *Leptolobium panamense* (Benth.) Sch.Rodr. & A.M.G.Azevedo; CAM, CHIS, GRO, HGO, MICH, OAX, QROO, TAB, VER, YUC; Red List: LC. |
| ***Leucaena*** |
| *Leucaena* *collinsii* Britton & Rose; CHIS, OAX; banked; useful (MEDICINES). |
| *Leucaena* *confertiflora* Zárate; OAX, PUE (endemic); useful (FOOD, ANIMAL FOOD, ENVIRONMENTAL USES). |
| *Leucaena* *cuspidata* Standl.; CHIS, HGO, OAX, PUE, QRO, SLP, VER (endemic); Red List: VU; useful (ENVIRONMENTAL USES). |
| *Leucaena* *diversifolia* (Schltdl.) Benth.; CHIS, GTO, GRO, JAL, MEX, MOR, OAX, PUE, SLP, SIN, TAB, VER; banked; useful (FOOD, ANIMAL FOOD). |
| *Leucaena* *esculenta* (Moc. & Sessé ex DC.) Benth.; AGS, CAM, CHIS, CHIH, COL, GTO, GRO, HGO, JAL, MEX, MICH, MOR, OAX, PUE, QRO, SLP, VER, YUC, ZAC; banked; useful (FOOD, ANIMAL FOOD, MEDICINES, MATERIALS, FUELS, ENVIRONMENTAL USES). |
| *Leucaena* *greggii* S.Watson; COAH, NLE; Red List: VU. |
| *Leucaena* *involucrata* Zárate; SON (endemic); Red List: EN. |
| *Leucaena* *lanceolata* S.Watson; BCS, CAM, CHIS, CHIH, COL, DGO, GRO, JAL, MICH, MOR, NAY, OAX, PUE, SIN, SON, TAB, VER (endemic); banked; useful (FOOD, ANIMAL FOOD). |
| *Leucaena* *leucocephala* (Lam.) de Wit; AGS, BCN, BCS, CAM, CHIS, CHIH, COAH, COL, CDMX, DGO, GRO, HGO, JAL, MEX, MICH, MOR, NAY, NLE, OAX, PUE, QRO, QROO, SLP, SIN, SON, TAB, TAMS, TLAX, VER, YUC, ZAC; banked; useful (FOOD, ANIMAL FOOD, MEDICINES, MATERIALS, FUELS, ENVIRONMENTAL USES). |
| *Leucaena* *macrophylla* Benth.; CHIS, COL, DGO, GTO, GRO, JAL, MEX, MICH, MOR, NAY, OAX, PUE, SIN, VER, ZAC (endemic); banked; useful (FOOD). |
| *Leucaena* *pulverulenta* Benth.; COAH, GTO, HGO, NLE, OAX, PUE, QRO, SLP, TAMS, VER; Red List: LC; banked; useful (FOOD, ANIMAL FOOD, ENVIRONMENTAL USES). |
| *Leucaena* *retusa* Benth.; CHIH, COAH. |
| ***Libidibia*** |
| *Libidibia coriaria* (Jacq.) Schltdl.; BCS, CHIS, COL, GRO, JAL, MEX, MICH, MOR, NAY, OAX, PUE, SIN, VER; Red List: LC; banked; useful (MEDICINES). |
| *Libidibia sclerocarpa* (Standl.) Britton & Rose; COL, DGO, GRO, JAL, MICH, NAY, OAX, QROO, SIN, SON (endemic). |
| ***Lonchocarpus*** |
| *Lonchocarpus* *acuminatus* (Schltdl.) M.Sousa; CHIS, GRO, HGO, JAL, OAX, PUE, TAB, VER; Red List: LC. |
| *Lonchocarpus* *andrieuxii* M. Sousa; MEX, MOR, PUE (endemic). |
| *Lonchocarpus* *angusticarpus* M.Sousa; CHIS, OAX (endemic). |
| *Lonchocarpus* *argyrotrichus* Harms; GRO, MEX, MICH, MOR, OAX, PUE (endemic). |
| *Lonchocarpus* *atropurpureus* Benth.; CHIS, GRO, JAL, MICH, NAY, OAX, VER; Red List: LC. |
| *Lonchocarpus* *balsensis* M.Sousa & J.C.Soto; GRO, MICH (endemic). |
| *Lonchocarpus* *barbatus* M.Sousa, E.Martínez & Ramos; VER (endemic). |
| *Lonchocarpus* *berriozabalensis* Miranda ex M.Sousa; CHIS. |
| *Lonchocarpus* *brachyanthus* M.Sousa; MICH (endemic). |
| *Lonchocarpus* *castilloi* Standl.; CAM, CHIS, QROO, TAB, YUC; Red List: LC; useful (MATERIALS). |
| *Lonchocarpus* *caudatus* Pittier; CHIS, COL, GRO, HGO, JAL, MEX, MICH, MOR, OAX, PUE, QRO, SLP, VER (endemic); Red List: LC. |
| *Lonchocarpus* *cochleatus* Pittier; CHIS, COL, GRO, JAL, MICH, NAY, OAX, QROO (endemic). |
| *Lonchocarpus* *comitensis* Pittier; CHIS (endemic). |
| *Lonchocarpus* *congestiflorus* M.Sousa & J.Linares; CHIS. |
| *Lonchocarpus* *constrictus* Pittier; CHIS, COL, GRO, JAL, MICH, OAX (endemic). |
| *Lonchocarpus* *crassicalyx* M.Sousa; OAX (endemic). |
| *Lonchocarpus* *emarginatus* Pittier; GRO, MOR, OAX (endemic). |
| *Lonchocarpus* *epigaeus* M.Sousa; GRO, MEX, MICH (endemic). |
| *Lonchocarpus* *eriocarinalis* Micheli; COL, GRO, JAL, MICH, OAX, PUE (endemic); banked. |
| *Lonchocarpus* *eriophyllus* Benth.; GRO, JAL, MEX, MICH, MOR, OAX, PUE (endemic); useful (MEDICINES). |
| *Lonchocarpus* *foveolatus* M.Sousa; CHIS (endemic). |
| *Lonchocarpus* *galeottianus* Harms; OAX (endemic). |
| *Lonchocarpus* *grandifoliolatus* M.Sousa; OAX (endemic). |
| *Lonchocarpus* *guatemalensis* Benth.; CAM, CHIS, COL, DGO, GRO, JAL, MICH, NAY, OAX, PUE, QROO, SLP, SIN, TAB, VER, YUC; Red List: LC; useful (POISONS, MATERIALS). |
| *Lonchocarpus* *gyroides* M.Sousa & Cruz Durán; GRO (endemic). |
| *Lonchocarpus* *heptaphyllus* (Poir.) DC.; CHIS, TAB, VER; Red List: LC. |
| *Lonchocarpus* *hermannii* M.Sousa; CHIH, COL, GRO, JAL, MICH, MOR, NAY, OAX, PUE, SIN, SON (endemic); banked. |
| *Lonchocarpus* *hidalgensis* Lundell; HGO, NLE, PUE, QRO, SLP, TAMS, VER (endemic); banked. |
| *Lonchocarpus* *hintonii* Sandwith; COL, GRO, JAL, MEX, MICH, NAY, OAX, SIN, VER (endemic). |
| *Lonchocarpus* *hondurensis* Benth.; CAM, CHIS, OAX, QROO, TAB, VER, YUC; Red List: LC. |
| *Lonchocarpus* *huetamoensis* M.Sousa & J.C.Soto; GRO, MICH (endemic). |
| *Lonchocarpus* *isthmensis* M.Sousa; CHIS, OAX (endemic). |
| *Lonchocarpus* *jaliscensis* Pittier; JAL, NAY, ZAC (endemic). |
| *Lonchocarpus* *kerberi* Harms; (endemic). |
| *Lonchocarpus* *lanceolatus* Benth.; CHIS, COL, DGO, GRO, JAL, MICH, NAY, OAX, SIN, VER; Red List: LC. |
| *Lonchocarpus* *latimarginatus* M.Sousa; CHIS, OAX, TAB, VER (endemic). |
| *Lonchocarpus* *lineatus* Pittier; CAM, CHIS, OAX, PUE, TAB, VER; Red List: LC. |
| *Lonchocarpus* *longipedunculatus* M.Sousa & J.C.Soto; GRO, JAL, MICH (endemic). |
| *Lonchocarpus* *longistylus* Pittier; useful (MEDICINES). |
| *Lonchocarpus* *luteomaculatus* Pittier; CAM, CHIS, OAX, QROO, TAB, VER, YUC; Red List: LC. |
| *Lonchocarpus* *magallanesii* M.Sousa; COL, JAL, MICH (endemic). |
| *Lonchocarpus* *major* M.Sousa; COL, MICH (endemic). |
| *Lonchocarpus* *martinezii* M.Sousa; CHIS (endemic). |
| *Lonchocarpus* *michoacanicus* M.Sousa; MICH (endemic). |
| *Lonchocarpus* *minimiflorus* Donn.Sm.; CHIS, OAX; Red List: LC. |
| *Lonchocarpus* *minor* M.Sousa; COL, JAL, NAY (endemic); banked. |
| *Lonchocarpus* *molinae* Standl. & L.O.Williams; CHIS, OAX, VER (endemic); Red List: CR. |
| *Lonchocarpus* *morenoi* M.Sousa; CHIS. |
| *Lonchocarpus* *multifoliolatus* M.Sousa; CHIS, OAX, TAB, VER (endemic). |
| *Lonchocarpus* *mutans* M.Sousa; COL, JAL, MICH, NAY, OAX, SIN (endemic). |
| *Lonchocarpus* *obovatus* Benth.; GRO, OAX, PUE (endemic); banked; useful. |
| *Lonchocarpus* *oliganthus* F.J.Herm.; CHIS, VER; Red List: LC. |
| *Lonchocarpus* *orizabensis* Lundell; OAX, VER (endemic). |
| *Lonchocarpus* *palmeri* Rose; (endemic). |
| *Lonchocarpus* *parviflorus* Benth.; CAM, CHIS, JAL, MICH, OAX, QROO, SIN (endemic); Red List: LC. |
| *Lonchocarpus* *pedunculatus* M.Sousa; CHIS (endemic). |
| *Lonchocarpus* *phaseolifolius* Benth.; CHIS, OAX; Red List: LC. |
| *Lonchocarpus* *pittieri* M.Sousa; COL, JAL, MICH, OAX (endemic). |
| *Lonchocarpus* *plicatus* M.Sousa; VER (endemic). |
| *Lonchocarpus* *punctatus* Kunth; CAM, CHIS, CHIH, COL, DGO, GRO, HGO, JAL, MICH, MOR, NAY, OAX, PUE, QROO, SIN, SON, TAB, TAMS, VER, YUC; Red List: LC. |
| *Lonchocarpus* *purpureus* Pittier ; CHIS, VER. |
| *Lonchocarpus* *robustus* Pittier; CHIS, OAX, TAB, VER. |
| *Lonchocarpus* *rugosus* Benth.; CAM, CHIS, COL, GRO, HGO, JAL, MEX, MICH, MOR, OAX, PUE, QRO, QROO, SLP, TAB, TAMS, VER, YUC; Red List: LC; banked; useful (MATERIALS). |
| *Lonchocarpus* *salvadorensis* Pittier; CHIS, COL, GTO, GRO, JAL, MICH, NAY, OAX, TAB; Red List: LC. |
| *Lonchocarpus* *sanctuarii* Standl. & L.O.Williams; CHIS; Red List: CR. |
| *Lonchocarpus* *santarosanus* Donn.Sm.; CHIS, OAX, QRO, SLP, TAB, VER; Red List: VU; useful (MATERIALS, FUELS). |
| *Lonchocarpus* *schiedeanus* (Schltdl.) Harms; CHIS, COL, JAL, NAY, OAX, SIN, TAB, VER. |
| *Lonchocarpus* *schubertiae* M.Sousa; GRO, MEX, MICH (endemic). |
| *Lonchocarpus* *septentrionalis* M.Sousa; OAX, VER (endemic). |
| *Lonchocarpus* *sinaloensis* (Gentry) F.J.Herm.; COL, DGO, JAL, SIN (endemic). |
| *Lonchocarpus* *spectabilis* F.J.Herm.; GRO, MOR, OAX (endemic). |
| *Lonchocarpus* *sumiderensis* M.Sousa; CHIS (endemic). |
| *Lonchocarpus* *sylvicola* M.Sousa; CHIS (endemic). |
| *Lonchocarpus* *tenorioi* M.Sousa; GRO, MICH (endemic). |
| *Lonchocarpus* *tuxtepecensis* M.Sousa; OAX, VER (endemic). |
| *Lonchocarpus* *verrucosus* M.Sousa; CHIS, OAX, VER. |
| *Lonchocarpus* *vittatus* M.Sousa; CHIS, VER. |
| *Lonchocarpus* *yucatanensis* Pittier; CAM, QROO, YUC; useful (MATERIALS, SOCIAL USES). |
| *Lonchocarpus* *wendtii* M. Sousa ; OAX, TAB, VER. |
| ***Lupinus*** |
| *Lupinus jaimehintonianus* B.L.Turner; OAX (endemic). |
| *Lupinus reflexus* Rose; COL, CDMX, JAL, MEX, MICH, OAX, SLP (endemic). |
| ***Lysiloma*** |
| *Lysiloma acapulcense* (Kunth) Benth.; AGS, CAM, CHIS, CHIH, COL, DGO, GTO, GRO, HGO, JAL, MEX, MICH, MOR, NAY, OAX, PUE, QRO, QROO, SLP, SIN, SON, TAB, TAMS, VER, YUC, ZAC; Red List: LC; banked; useful (FOOD, ANIMAL FOOD, MATERIALS, FUELS, ENVIRONMENTAL USES). |
| *Lysiloma auritum* (Schltdl.) Benth.; CHIS, GRO, OAX, TAB, VER; Red List: LC. |
| *Lysiloma candidum* Brandegee; BCN, BCS, SON (endemic); banked. |
| *Lysiloma divaricatum* (Jacq.) J.F.Macbr.; AGS, BCN, BCS, CAM, CHIS, CHIH, COL, DGO, GTO, GRO, HGO, JAL, MEX, MICH, MOR, NAY, OAX, PUE, QRO, SLP, SIN, SON, TAB, TAMS, VER, ZAC; Red List: LC; banked; useful (FOOD, ANIMAL FOOD, MEDICINES, MATERIALS, FUELS, ENVIRONMENTAL USES). |
| *Lysiloma latisiliquum* (L.) Benth.; CAM, CHIS, QROO, TAB, VER, YUC; Red List: LC; banked; useful (MATERIALS). |
| *Lysiloma tergeminum* Benth.; COL, GRO, JAL, MEX, MICH, MOR, NAY, OAX, PUE (endemic); banked. |
| *Lysiloma watsonii* Rose; CHIH, SIN, SON; Red List: LC; banked; useful (MEDICINES). |
| ***Machaerium*** |
| *Machaerium* *biovulatum* Micheli; CAM, CHIS, HGO, MEX, MICH, OAX, QRO, SLP, TAB, VER; Red List: LC. |
| *Machaerium* *chiapense* Brandegee; CHIS, OAX, TAB, VER. |
| *Machaerium* *conzattii* Rudd; OAX (endemic). |
| *Machaerium* *pittieri* J.F.Macbr.; CHIS, GRO, HGO, OAX, VER. |
| *Machaerium* *seemannii* Benth. ex Seem.; CAM, CHIS, QROO, TAB; Red List: LC. |
| ***Mariosousa*** |
| *Mariosousa* *acatlensis* (Benth.) Seigler & Ebinger; AGS, CHIS, COL, DGO, GTO, GRO, JAL, MEX, MICH, MOR, NAY, OAX, PUE, SIN, VER, ZAC (endemic). |
| *Mariosousa* *centralis* (Britton & Rose) Seigler & Ebinger; CAM, CHIS, OAX, QROO, TAB, YUC. |
| *Mariosousa* *compacta* (Rose) Seigler & Ebinger; MEX, OAX, PUE, VER (endemic); banked; useful. |
| *Mariosousa* *coulteri* (Benth.) Seigler & Ebinger; BCS, CHIS, CHIH, COAH, COL, DGO, GTO, GRO, HGO, JAL, MEX, MICH, MOR, NLE, OAX, PUE, QRO, SLP, SIN, SON, TAMS, VER, ZAC; banked; useful. |
| *Mariosousa* *dolichostachya* (S.F.Blake) Seigler & Ebinger; CAM, QROO, YUC; Red List: LC; banked. |
| *Mariosousa* *durangensis* (Britton & Rose) Seigler & Ebinger; DGO, SLP (endemic). |
| *Mariosousa* *mammifera* (Schltdl.) Seigler & Ebinger; COL, GTO, HGO, JAL, MICH, MOR, NLE, OAX, PUE, QRO, SLP, TAMS (endemic). |
| *Mariosousa* *millefolia* (S.Watson) Seigler & Ebinger; CHIH, SIN, SON. |
| *Mariosousa* *russelliana* (Britton & Rose) Seigler & Ebinger; NAY, SIN, SON (endemic). |
| *Mariosousa* *salazarii* (Britton & Rose) Seigler & Ebinger; GRO, JAL, MEX, MICH, MOR, OAX, PUE (endemic). |
| *Mariosousa* *sericea* (M.Martens & Galeotti) Seigler & Ebinger; OAX, PUE (endemic). |
| *Mariosousa* *usumacintensis* (Lundell) Seigler & Ebinger; CAM, CHIS, OAX, QROO, TAB, VER, YUC. |
| *Mariosousa* *willardiana* (Rose) Seigler & Ebinger; BCN, BCS, SIN, SON. |
| ***Microlobius*** |
| *Microlobius foetidus* (Jacq.) M.Sousa & G.Andrade; GRO, JAL, MEX, MICH, MOR, OAX, PUE, SIN (endemic); banked. |
| ***Mimosa*** |
| *Mimosa* *acantholoba* (Humb. & Bonpl. ex Willd.) Poir.; CHIS, COL, GRO, JAL, MICH, NAY, OAX, SIN; Red List: LC; banked. |
| *Mimosa* *aculeaticarpa* Ortega; AGS, BCN, BCS, CHIS, CHIH, COAH, COL, CDMX, DGO, GTO, GRO, HGO, JAL, MEX, MICH, MOR, NAY, NLE, OAX, PUE, QRO, SLP, SIN, SON, TAMS, TLAX, VER, ZAC; Red List: LC; banked. |
| *Mimosa* *arenosa* (Willd.) Poir.; COL, GRO, JAL, MICH, OAX; banked. |
| *Mimosa* *bahamensis* Benth.; CAM, CHIS, QROO, TAB, VER, YUC; banked; useful (FUELS). |
| *Mimosa* *benthamii* J.F.Macbr.; AGS, COL, DGO, GTO, GRO, JAL, MEX, MICH, MOR, NAY, OAX, PUE, SLP, SIN, ZAC (endemic); banked; useful (MATERIALS). |
| *Mimosa* *brandegeei* B.L.Rob.; BCS, COL, JAL, MICH, SIN (endemic); banked. |
| *Mimosa* *costenya* McVaugh; COL, JAL, NAY (endemic). |
| *Mimosa* *galeottii* Benth.; AGS, COL, DGO, GTO, GRO, JAL, MEX, MICH, MOR, NAY, OAX, PUE, SLP (endemic). |
| *Mimosa* *goldmanii* B.L.Rob.; CHIS, GRO, MOR, OAX, PUE (endemic). |
| *Mimosa* *leucaenoides* Benth.; GTO, HGO, QRO, SLP, TAMS, VER (endemic); banked. |
| *Mimosa* *mollis* Benth.; DGO, GRO, OAX, PUE (endemic); banked. |
| *Mimosa* *palmeri* Rose; CHIH, GRO, JAL, MICH, NAY, SIN, SON (endemic). |
| *Mimosa* *platycarpa* Benth.; CHIS, OAX. |
| *Mimosa* *rhododactyla* B.L.Rob.; DGO, GRO, MICH, MOR, NAY, PUE, SIN (endemic); banked. |
| *Mimosa* *rosei* B.L.Rob.; COL, GRO, JAL, MICH, NAY, ZAC (endemic). |
| *Mimosa* *sotoi* R.Grether & V.W.Steinm.; MICH (endemic). |
| *Mimosa* *tenuiflora* (Willd.) Poir.; CHIS, HGO, OAX; Red List: LC; useful (MEDICINES). |
| ***Muellera*** |
| *Muellera* *monilis* (L.) M.J.Silva & A.M.G.Azevedo; TAB, VER. |
| *Muellera* *unifoliolata* (Benth.) M.Sousa; OAX, PUE, TAB, VER (endemic). |
| ***Myrospermum*** |
| *Myrospermum frutescens* Jacq.; CAM, CHIS, COL, GRO, JAL, MEX, MICH, MOR, OAX; Red List: LC. |
| *Myrospermum sousanum* A.Delgado & M.C.Johnst.; NLE. |
| ***Myroxylon*** |
| *Myroxylon balsamum* (L.) Harms; CAM, CHIS, DGO, GRO, HGO, MEX, MICH, MOR, NAY, OAX, QROO, SIN, TAB, VER, YUC; useful (MEDICINES, MATERIALS). |
| ***Olneya*** |
| *Olneya tesota* A.Gray; BCN, BCS, SIN, SON; Red List: NT; NOM-59: Pr; banked; useful. |
| ***Ormosia*** |
| *Ormosia* *carinata* N.Zamora; GRO, OAX, VER. |
| *Ormosia* *isthmensis* Standl.; CHIS, OAX, PUE, TAB, VER; NOM-59: P; banked. |
| *Ormosia* *macrocalyx* Ducke; CHIS, TAB, VER; NOM-59: P. |
| *Ormosia* *oaxacana* Rudd; CHIS, GRO, OAX (endemic). |
| *Ormosia* *velutina* Rudol. |
| ***Parkinsonia*** |
| *Parkinsonia aculeata* L.; BCN, BCS, CAM, CHIS, CHIH, COAH, COL, DGO, GTO, GRO, HGO, JAL, MEX, MICH, MOR, NAY, NLE, OAX, PUE, QRO, QROO, SLP, SIN, SON, TAB, TAMS, VER, YUC, ZAC; Red List: LC; banked; useful (MEDICINES, ENVIRONMENTAL USES). |
| *Parkinsonia microphylla* Torr.; BCN, BCS, SIN, SON; banked. |
| *Parkinsonia praecox* (Ruiz & Pav.) Hawkins; BCS, CHIS, CHIH, COL, DGO, GTO, GRO, JAL, MEX, MICH, MOR, NAY, OAX, PUE, SIN, SON, TAMS, VER, ZAC; banked; useful (FOOD, ANIMAL FOOD, MEDICINES, FUELS). |
| *Parkinsonia texana* (A.Gray) S.Watson; COAH, DGO, NLE, SLP, TAMS. |
| ***Peltogyne*** |
| *Peltogyne mexicana* Martínez; GRO. |
| ***Peltophorum*** |
| *Peltophorum dubium* (Spreng.) Taub.; Red List: LC. |
| ***Piptadenia*** |
| *Piptadenia flava* (Spreng. ex DC.) Benth.; CAM, CHIS, COL, GRO, JAL, MEX, MICH, MOR, OAX, PUE, QROO, YUC; useful (MATERIALS, FUELS). |
| *Piptadenia obliqua* (Pers.) J.F.Macbr.; CHIS, COL, GRO, JAL, MICH, NAY, OAX, SIN; banked. |
| ***Piscidia*** |
| *Piscidia carthagenensis* Jacq.; CHIS, COL, GRO, JAL, MEX, MICH, OAX; banked. |
| *Piscidia* *grandifolia* (Donn.Sm.) I.M.Johnst.; CHIS, COL, GRO, JAL, MEX, MICH, MOR, OAX, PUE, VER; Red List: LC; banked; useful (MATERIALS). |
| *Piscidia mollis* Rose; CHIH, SIN, SON (endemic). |
| *Piscidia piscipula* (L.) Sarg.; CAM, CHIS, GRO, HGO, JAL, MEX, MICH, NAY, NLE, OAX, PUE, QRO, QROO, SLP, TAB, TAMS, VER, YUC; banked; useful (MEDICINES, MATERIALS, FUELS). |
| ***Pithecellobium*** |
| *Pithecellobium* *dulce* (Roxb.) Benth.; AGS, BCN, BCS, CAM, CHIS, CHIH, COAH, COL, DGO, GTO, GRO, HGO, JAL, MEX, MICH, MOR, NAY, NLE, OAX, PUE, QRO, QROO, SLP, SIN, SON, TAB, TAMS, VER, YUC, ZAC; Red List: LC; banked; useful (FOOD, ANIMAL FOOD, MEDICINES, MATERIALS, FUELS). |
| *Pithecellobium* *furcatum* Benth.; CHIS, OAX, TAB, VER. |
| *Pithecellobium* *hymenaeifolium* (Humb. & Bonpl. ex Willd.) Benth.; CHIS, OAX, TAB, VER. |
| *Pithecellobium* *keyense* Britton; CAM, QROO, VER, YUC. |
| *Pithecellobium* *lanceolatum* (Humb. & Bonpl. ex Willd.) Benth.; CAM, CHIS, COL, GRO, HGO, JAL, MICH, NAY, OAX, PUE, QRO, QROO, SLP, SIN, TAB, TAMS, VER, YUC; useful (MATERIALS, FUELS). |
| *Pithecellobium* *macrandrium* Donn.Sm.; CHIS, TAB. |
| *Pithecellobium* *oblongum* Benth.; GRO, JAL, MICH, OAX; Red List: LC. |
| *Pithecellobium* *unguis-cati* (L.) Benth.; CAM, COL, GRO, JAL, MICH, MOR, NAY, OAX, QRO, QROO, SLP, SIN, SON, TAB, TAMS, VER, YUC; useful (MEDICINES). |
| *Pithecellobium* *winzerlingii* Britton & Rose; CAM, QROO, TAB, VER. |
| ***Platymiscium*** |
| *Platymiscium* *calyptratum* M.Sousa & Klitg.; HGO, VER (endemic). |
| *Platymiscium* *dimorphandrum* Donn.Sm.; CHIS, GRO, HGO, OAX, TAB, VER; Red List: LC. |
| *Platymiscium* *jejunum* Klitg.; GRO, OAX. |
| *Platymiscium* *lasiocarpum* Sandwith; COL, GRO, JAL, MEX, MICH, OAX (endemic); NOM-59: P. |
| *Platymiscium* *trifoliolatum* Benth.; CHIH, JAL, NAY, SIN, SON, ZAC (endemic). |
| *Platymiscium* *yucatanum* Standl.; CAM, CHIS, OAX, QROO, TAB, VER, YUC; useful (MATERIALS). |
| ***Poeppigia*** |
| *Poeppigia procera* (Poepp. ex Spreng.) C.Presl; CHIS, COL, GRO, JAL, MEX, MICH, OAX. |
| ***Prosopidastrum*** |
| *Prosopidastrum mexicanum* (Dressler) Burkart; BCN (endemic). |
| ***Prosopis*** |
| *Prosopis* *articulata* S.Watson; BCN, BCS, SON; banked; useful. |
| *Prosopis* *glandulosa* Torr.; BCN, BCS, CHIH, COAH, DGO, NAY, NLE, QROO, SLP, SIN, SON, TAMS, VER, YUC, ZAC; banked; useful (ANIMAL FOOD, MEDICINES). |
| *Prosopis* *juliflora* (Sw.) DC.; AGS, BCS, CHIS, CHIH, COAH, COL, GTO, GRO, HGO, JAL, MEX, MICH, MOR, NAY, NLE, OAX, PUE, QRO, QROO, SLP, SIN, SON, TAMS, VER, YUC, ZAC; banked; useful (FOOD, ANIMAL FOOD, MEDICINES, MATERIALS, FUELS). |
| *Prosopis* *laevigata* (Humb. & Bonpl. ex Willd.) M.C.Johnst.; AGS, CHIS, CHIH, COAH, COL, CDMX, DGO, GTO, GRO, HGO, JAL, MEX, MICH, MOR, NAY, NLE, OAX, PUE, QRO, SLP, TAMS, VER, ZAC; Red List: LR/lc; banked; useful (FOOD, ANIMAL FOOD, MEDICINES, MATERIALS, FUELS, ENVIRONMENTAL USES). |
| *Prosopis* *palmeri* S.Watson; BCS (endemic). |
| *Prosopis* *pubescens* Benth.; BCN, CHIH, SON; Red List: LC; banked. |
| *Prosopis* *tamaulipana* Burkart; NLE, SLP, TAMS, VER (endemic). |
| *Prosopis* *velutina* Wooton; BCN, BCS, CHIH, COAH, MICH, SON; Red List: LC; banked. |
| ***Pseudosamanea*** |
| *Pseudosamanea guachapele* (Kunth) Harms; CAM, CHIS, OAX, TAB, VER; Red List: LC. |
| ***Psorothamnus*** |
| *Psorothamnus spinosus* (A.Gray) Barneby; BCN, BCS, SON. |
| ***Pterocarpus*** |
| *Pterocarpus* *acapulcensis* Rose; CHIS, GRO, JAL, MICH, NAY, OAX, PUE, TAB, VER (endemic); Red List: VU; useful (MATERIALS). |
| *Pterocarpus* *michelianus* N.Zamora; GRO, OAX. |
| *Pterocarpus* *officinalis* Jacq.; CHIS, OAX, VER, YUC; Red List: NT. |
| *Pterocarpus* *orbiculatus* DC.; COL, GRO, JAL, MEX, MICH, MOR, NAY, OAX, PUE, VER (endemic). |
| *Pterocarpus* *rohrii* Vahl; CHIS, MICH, OAX, QROO, TAB, VER; banked; useful (MATERIALS, ENVIRONMENTAL USES). |
| ***Robinia*** |
| *Robinia neomexicana* A.Gray; CHIH, SON; Red List: LC; banked. |
| ***Samanea*** |
| *Samanea saman* (Jacq.) Merr.; Red List: LC; banked. |
| ***Schizolobium*** |
| *Schizolobium parahyba* (Vell.) S.F.Blake; CHIS, OAX, QROO, TAB, VER; banked; useful (MATERIALS, FUELS). |
| ***Senegalia*** |
| *Senegalia* *gaumeri* (S.F.Blake) Britton & Rose; CAM, QROO, YUC; Red List: LR/nt; banked; useful (ANIMAL FOOD, MATERIALS, FUELS, ENVIRONMENTAL USES). |
| *Senegalia* *macilenta* (Rose) Britton & Rose; COAH, COL, DGO, GRO, JAL, MEX, MICH, MOR, NAY, NLE, OAX, PUE, SIN (endemic); banked. |
| *Senegalia* *micrantha* Britton & Rose; GTO, HGO, NLE, QRO, SLP, TAMS, VER (endemic). |
| *Senegalia* *mirandae* (L.Rico) Seigler & Ebinger; CHIS, OAX (endemic). |
| *Senegalia* *occidentalis* (Rose) Britton & Rose; BCN, BCS, CHIH, HGO, QRO, SLP, SIN, SON, ZAC (endemic). |
| *Senegalia* *peninsularis* Britton & Rose; BCS (endemic); banked; useful. |
| *Senegalia* *picachensis* (Brandegee) Britton & Rose; CHIS, CHIH, COL, DGO, GRO, JAL, MEX, MICH, MOR, OAX, PUE; useful (MEDICINES). |
| *Senegalia* *polyphylla* (DC.) Britton & Rose; Red List: LC. |
| *Senegalia* *subangulata* (Rose) Britton & Rose; GRO, HGO, OAX, PUE, QRO, SLP (endemic); banked; useful (ANIMAL FOOD, FUELS). |
| ***Senna*** |
| *Senna* *andrieuxii* (Benth.) H.S.Irwin & Barneby; GRO, OAX, PUE (endemic); banked; useful. |
| *Senna* *atomaria* (L.) H.S.Irwin & Barneby; BCS, CAM, CHIS, CHIH, COL, DGO, GTO, GRO, HGO, JAL, MEX, MICH, MOR, NAY, OAX, PUE, QRO, QROO, SLP, SIN, SON, TAB, TAMS, VER, YUC; Red List: LC; banked; useful (ANIMAL FOOD, MATERIALS, FUELS). |
| *Senna* *bacillaris* (L.f.) H.S.Irwin & Barneby; Red List: LC. |
| *Senna* *multifoliolata* (Paul G.Wilson) H.S.Irwin & Barneby; COL, GRO, JAL, MICH, OAX (endemic); Red List: VU. |
| *Senna* *multijuga* (Rich.) H.S.Irwin & Barneby; CHIS, HGO, MEX, MICH, MOR, OAX, PUE, TAB, VER (endemic); useful (ENVIRONMENTAL USES). |
| *Senna* *nicaraguensis* (Benth.) H.S.Irwin & Barneby; CHIS, COL, GRO, HGO, JAL, MEX, MICH, MOR, OAX, SIN, VER; Red List: LC. |
| *Senna* *papillosa* (Britton & Rose) H.S.Irwin & Barneby; CAM, CHIS, OAX, PUE, QROO, TAB, VER; Red List: LC; useful (MEDICINES). |
| *Senna* *peralteana* (Kunth) H.S.Irwin & Barneby; CAM, CHIS, OAX, QROO, TAB, YUC. |
| *Senna* *polyantha* (Collad.) H.S.Irwin & Barneby; (endemic); banked; useful. |
| *Senna* *racemosa* (Mill.) H.S.Irwin & Barneby; CAM, CHIS, COL, GRO, HGO, JAL, MEX, MICH, MOR, OAX, QRO, QROO, SLP, VER, YUC; banked; useful. |
| *Senna* *skinneri* (Benth.) H.S.Irwin & Barneby; CHIS, COL, GRO, JAL, MEX, MICH, MOR, OAX, PUE; Red List: LC; useful (MEDICINES). |
| *Senna* *sousana* H.S.Irwin & Barneby; OAX (endemic). |
| *Senna* *spectabilis* (DC.) H.S.Irwin & Barneby; Red List: LC; useful (MEDICINES). |
| *Senna* *wislizeni* (A.Gray) H.S.Irwin & Barneby; CHIH, COAH, COL, DGO, GTO, GRO, HGO, JAL, MEX, MICH, MOR, NAY, NLE, OAX, PUE, QRO, SLP, SON, TAMS, VER, ZAC; banked; useful. |
| ***Sesbania*** |
| *Sesbania longifolia* DC.; GTO, JAL, MICH, QRO, SLP, TAMS. |
| ***Styphnolobium*** |
| *Styphnolobium* *burseroides* M.Sousa, Rudd & Medrano; MOR, OAX, PUE (endemic). |
| *Styphnolobium* *conzattii* (Standl.) M.Sousa & Rudd; OAX (endemic). |
| *Styphnolobium* *parviflorum* M.Sousa & Rudd; VER (endemic). |
| *Styphnolobium* *protantherum* M.Sousa & Rudd; JAL (endemic). |
| *Styphnolobium* *sporadicum* M.Sousa & Rudd; CHIS, OAX. |
| ***Swartzia*** |
| *Swartzia* *cubensis* (Britton & P.Wilson) Standl.; CAM, CHIS, QROO, TAB, VER, YUC. |
| *Swartzia* *guatemalensis* (Donn.Sm.) Pittier; CHIS, OAX, TAB, VER; Red List: LC. |
| *Swartzia mexicana* M.Sousa & R.Grether; OAX. |
| *Swartzia* *myrtifolia* Sm.; CHIS, OAX, TAB, VER; Red List: LC. |
| *Swartzia* simplex (Sw.) Spreng.; CHIS, COL, GRO, JAL, MICH, NAY, OAX, TAB; Red List: LC; useful (MEDICINES, MATERIALS). |
| ***Tara*** |
| *Tara cacalaco* (Bonpl.) Molinari & Sánchez Och.; CAM, CHIS, CHIH, COL, CDMX, DGO, GRO, HGO, JAL, MEX, MICH, MOR, NAY, OAX, PUE, SIN, VER, YUC; banked; useful (MEDICINES, MATERIALS). |
| *Tara vesicaria* (L.) Molinari, Sánchez Och. & Mayta; CAM, CHIS, QROO, TAB, VER, YUC; banked; useful (MATERIALS). |
| ***Vachellia*** |
| *Vachellia* *bilimekii* (J.F.Macbr.) Seigler & Ebinger; GRO, MEX, MOR, OAX, PUE (endemic); banked; useful (ANIMAL FOOD, FUELS). |
| *Vachellia* *brandegeeana* (I.M.Johnst.) Seigler & Ebinger; BCS (endemic); banked. |
| *Vachellia* *californica* (Brandegee) Seigler & Ebinger; BCS, CAM, CHIS, CHIH, QROO, SON, YU (endemic); banked; useful. |
| *Vachellia* *campeachiana* (Mill.) Seigler & Ebinger; AGS, BCN, BCS, CAM, CHIS, CHIH, COAH, COL, DGO, GRO, JAL, MEX, MICH, MOR, NAY, OAX, PUE, QROO, SLP, SIN, SON, TAMS, VER, YUC, ZAC; banked; useful (ANIMAL FOOD, MEDICINES, MATERIALS, FUELS, ENVIRONMENTAL USES). |
| *Vachellia* *chiapensis* (Saff.) Seigler & Ebinger; CAM, CHIS, OAX, QROO, TAB, VER; Red List: LR/lc. |
| *Vachellia* *collinsii* (Saff.) Seigler & Ebinger; CAM, CHIS, GRO, MICH, NAY, OAX, QROO, TAB, VER, YUC; banked; useful (ANIMAL FOOD, MEDICINES, POISONS, FUELS, ENVIRONMENTAL USES, SOCIAL USES). |
| *Vachellia* *cookii* (Saff.) Seigler & Ebinger; CHIS, TAB. |
| *Vachellia* *cornigera* (L.) Seigler & Ebinger; CAM, CHIS, COL, GRO, HGO, JAL, MOR, OAX, PUE, QRO, QROO, SLP, SIN, TAB, TAMS, VER, YUC, ZAC; banked; useful (FOOD, ANIMAL FOOD, MEDICINES, POISONS, FUELS, ENVIRONMENTAL USES). |
| *Vachellia* *farnesiana* (L.) Wight & Arn.; AGS, BCN, BCS, CAM, CHIS, CHIH, COAH, COL, DGO, GTO, GRO, HGO, JAL, MEX, MICH, MOR, NAY, NLE, OAX, PUE, QRO, QROO, SLP, SIN, SON, TAB, TAMS, VER, YUC, ZAC; banked; useful (ANIMAL FOOD, MEDICINES, MATERIALS, FUELS, ENVIRONMENTAL USES, SOCIAL USES). |
| *Vachellia* *gentlei* (Standl.) Seigler & Ebinger; CAM, CHIS, QROO, TAB, YUC. |
| *Vachellia* *globulifera* (Saff.) Seigler & Ebinger; CAM, CHIS, OAX, QROO, VER, YUC. |
| *Vachellia* *hindsii* (Benth.) Seigler & Ebinger; CHIS, COL, DGO, GRO, JAL, MEX, MICH, NAY, OAX, PUE, SIN, YUC; useful (FUELS, ENVIRONMENTAL USES). |
| *Vachellia* *janzenii* (Ebinger & Seigler) Seigler & Ebinger; CHIS, TAB (endemic). |
| *Vachellia* *macracantha* (Humb. & Bonpl. ex Willd.) Seigler & Ebinger; CAM, CHIS, COL, GRO, JAL, MEX, MICH, NAY, OAX, PUE, QROO, SIN, VER, YUC; banked; useful (ANIMAL FOOD, FUELS, ENVIRONMENTAL USES). |
| *Vachellia* *mayana* (Lundell) Seigler & Ebinger; CHIS, OAX, TAB, VER. |
| *Vachellia* *pennatula* (Schltdl. & Cham.) Seigler & Ebinger; AGS, CAM, CHIS, CHIH, COL, DGO, GTO, GRO, HGO, JAL, MEX, MICH, MOR, NAY, OAX, PUE, QRO, QROO, SLP, SIN, SON, TAMS, VER, YUC, ZAC; banked; useful (ANIMAL FOOD, MEDICINES, MATERIALS, FUELS, ENVIRONMENTAL USES). |
| *Vachellia* *rigidula* (Benth.) Seigler & Ebinger; CHIH, COAH, GTO, GRO, HGO, JAL, MEX, MICH, MOR, NAY, NLE, QRO, SLP, SON, TAMS, VER, ZAC; Red List: LC; banked. |
| *Vachellia* *schaffneri* (S.Watson) Seigler & Ebinger; AGS, BCN, BCS, CHIS, CHIH, COAH, COL, CDMX, DGO, GTO, HGO, JAL, MEX, MICH, NLE, OAX, PUE, QRO, SLP, SON, TAMS, TLAX, VER, ZAC; banked; useful (ANIMAL FOOD, FUELS, ENVIRONMENTAL USES). |
| *Vachellia* *sphaerocephala* (Cham. & Schltdl.) Seigler & Ebinger; COL, GRO, MICH, OAX, SLP, TAB, TAMS, VER, YUC (endemic). |
| ***Vatairea*** |
| *Vatairea lundellii* (Standl.) Killip; CAM, CHIS, OAX, QROO, TAB, VER; NOM-59: P; banked. |
| ***Zapoteca*** |
| *Zapoteca portoricensis* (Jacq.) H.M.Hern.; CHIS, COL, GTO, GRO, HGO, JAL, MEX, OAX, PUE, QRO, SLP, SIN, TAB, TAMS, VER, YUC, ZAC; banked. |
| *Zapoteca tetragona* (Willd.) H.M.Hern.; CHIS, COL, GRO, JAL, MEX, MICH, MOR, NAY, OAX, PUE, SIN, TAB, TAMS, VER; Red List: LC. |
| ***Zygia*** |
| *Zygia* *cognata* (Schltdl.) Britton & Rose; CAM, CHIS, OAX, QROO, TAB, VER, YUC; Red List: LC. |
| *Zygia* *conzattii* (Standl.) Britton & Rose; CAM, CHIS, GRO, OAX, QROO, TAB, VER, YUC. |
| *Zygia* *inaequalis* (Humb. & Bonpl. ex Willd.) Pittier; TAB; Red List: LC. |
| *Zygia* *latifolia* (L.) Fawc. & Rendle; TAB, VER. |
| *Zygia* *longifolia* (Humb. & Bonpl. ex Willd.) Britton & Rose; CHIS, OAX, TAB, VER; Red List: LC; useful (ANIMAL FOOD). |
| *Zygia* *paucijugata* (Lundell) L.Rico; OAX, TAB, VER. |
| *Zygia* *peckii* (B.L.Rob.) Britton & Rose; CHIS, TAB, VER; Red List: LC. |
| *Zygia* *turneri* (McVaugh) Barneby & J.W.Grimes; COL, JAL, MICH (endemic). |
| *Zygia* *unifoliolata* (Benth.) Pittier; CHIS, OAX, TAB, VER; Red List: LC. |
|  |
| **Fagaceae** |
| ***Fagus*** |
| *Fagus* *grandifolia* Ehrh.; HGO, PUE, SLP, TAMS, VER; Red List: LC. |
| ***Quercus*** |
| *Quercus* *acatenangensis* Trel.; Red List: LC. |
| *Quercus* *aculcingensis* Trel.; (endemic). |
| *Quercus* *acutifolia* Née; CHIS, COAH, COL, CDMX, DGO, GTO, GRO, HGO, JAL, MEX, MICH, MOR, NAY, NLE, OAX, PUE, QRO, SLP, SIN, TAMS, TLAX, VER, ZAC; Red List: VU; useful (MATERIALS, FUELS). |
| *Quercus* *aerea* Trel.; (endemic). |
| *Quercus* *affinis* Scheidw.; CHIS, COAH, CDMX, GTO, GRO, HGO, JAL, MEX, MICH, MOR, NLE, OAX, PUE, QRO, SLP, TAMS, VER (endemic); Red List: LC. |
| *Quercus* *agrifolia* Née; BCN; Red List: LC. |
| *Quercus* *ajoensis* C.H.Mull.; BCN, SON; Red List: VU. |
| *Quercus* *albocincta* Trel.; BCS, CHIH, DGO, SIN, SON (endemic); Red List: LC. |
| *Quercus* *alpescens* Trel.; HGO, NLE, QRO, VER (endemic); Red List: DD. |
| *Quercus* *aristata* Hook. & Arn.; AGS, COL, DGO, GTO, GRO, JAL, MEX, NAY, OAX, QRO, SIN, SON, VER, ZAC (endemic); Red List: LC. |
| *Quercus* *arizonica* Sarg.; BCS, CHIH, COAH, DGO, NAY, NLE, SIN, SON, TAMS; Red List: LC. |
| *Quercus* *barrancana* Spellenb.; CHIH, SON (endemic). |
| *Quercus* *benthamii* A.DC.; CHIS, CHIH, COAH, GRO, OAX; Red List: NT. |
| *Quercus* *brandegeei* Goldman; BCS (endemic); Red List: EN; banked. |
| *Quercus* *canbyi* Trel.; CHIH, COAH, NLE, SLP, TAMS ; Red List: LC. |
| *Quercus* *carmenensis* C.H.Mull.; COAH (endemic); Red List: EN. |
| *Quercus* *castanea* Née; AGS, CHIS, CHIH, COAH, COL, CDMX, DGO, GTO, GRO, HGO, JAL, MEX, MICH, MOR, NAY, NLE, OAX, PUE, QRO, SLP, SIN, SON, TAMS, TLAX, VER, ZAC; Red List: LC; banked; useful (MATERIALS, FUELS). |
| *Quercus* *cedrosensis* C.H.Mull.; BCN (endemic); Red List: VU; banked. |
| *Quercus* *chihuahuensis* Trel.; AGS, CHIH, DGO, GTO, HGO, JAL, NAY, NLE, QRO, SLP, SIN, SON, ZAC (endemic); Red List: LC. |
| *Quercus* *chrysolepis* Liebm.; BCN, CHIH, SON; Red List: LC. |
| *Quercus* *coahuilensis* Nixon & C.H.Müll.; CHIH, COAH (endemic); Red List: DD. |
| *Quercus* *coffeicolor* Trel.; COL, DGO, HGO, JAL, NAY, QRO, SLP, SIN, ZAC (endemic); Red List: DD. |
| *Quercus* *convallata* Trel.; DGO, JAL, NAY, ZAC (endemic); Red List: LC. |
| *Quercus* *conzattii* Trel.; DGO, GRO, JAL, NAY, OAX, ZAC (endemic); Red List: LR/lc; useful (ANIMAL FOOD, MATERIALS, FUELS). |
| *Quercus* *cortesii* Liebm.; CHIS, COL, GRO, JAL, OAX, PUE, VER. |
| *Quercus* *crassifolia* Bonpl.; AGS, CHIS, CHIH, COAH, COL, CDMX, DGO, GTO, GRO, HGO, JAL, MEX, MICH, MOR, NAY, NLE, OAX, PUE, QRO, SLP, SIN, SON, TAMS, TLAX, VER, ZAC; Red List: LC; banked; useful (FOOD, MEDICINES, MATERIALS, FUELS). |
| *Quercus* *crassipes* Bonpl.; AGS, CHIS, COL, COAH, CDMX, DGO, GTO, GRO, HGO, JAL, MEX, MICH, MOR, NAY, NLE, OAX, PUE, QRO, SLP, TAMS, TLAX, VER, ZAC (endemic); Red List: LC; useful (FOOD, MEDICINES, MATERIALS, FUELS). |
| *Quercus* *crispifolia* Trel.; CHIS, OAX. |
| *Quercus* *crispipilis* Trel.; CHIS. |
| *Quercus* *cualensis* L.M.González; JAL (endemic). |
| *Quercus* *delgadoana* S.Valencia, Nixon & L.M.Kelly; CHIS, HGO, OAX, PUE, QRO, SLP, TAB, VER (endemic); Red List: EN. |
| *Quercus* *depressipes* Trel.; CHIH, DGO, GTO, HGO, JAL, NLE, QRO, SLP, SIN, SON, TAMS, ZAC; Red List: LC. |
| *Quercus* *deserticola* Trel.; AGS, CHIS, CHIH, COL, CDMX, DGO, GTO, GRO, HGO, JAL, MEX, MICH, MOR, OAX, PUE, QRO, SLP, SIN, SON, TAMS, TLAX, VER, ZAC (endemic); Red List: LC; useful (MATERIALS, FUELS). |
| *Quercus* *devia* Goldman; BCS (endemic); Red List: VU. |
| *Quercus* *diversifolia* Née; COAH, HGO, MEX, MOR, NLE, OAX, SLP, TAMS, VER (endemic); Red List: EN. |
| *Quercus* *dumosa* Nutt.; BCN, CHIH, JAL, MICH; Red List: EN; banked. |
| *Quercus* *durifolia* Seemen ex Loes.; CHIH, COAH, DGO, NLE, OAX, QRO, SLP, SIN, SON, TAMS, ZAC (endemic); Red List: NT. |
| *Quercus* *eduardi* Trel.; AGS, CHIH, COAH, DGO, GTO, HGO, JAL, MEX, MICH, NAY, NLE, OAX, PUE, QRO, SLP, SIN, TAMS, VER, ZAC (endemic); Red List: LC. |
| *Quercus* *edwardsiae* C.H.Mull.; NLE (endemic); Red List: DD. |
| *Quercus* *elliptica* Née; CHIS, COL, DGO, GRO, HGO, JAL, MEX, MICH, NAY, OAX, PUE, QRO, SLP, SIN, VER; Red List: LC; useful (MEDICINES). |
| *Quercus* *emoryi* Torr.; BCN, CHIH, COAH, DGO, JAL, NLE, SLP, SON, ZAC; Red List: LC. |
| *Quercus* *engelmannii* Greene; BCN; Red List: EN. |
| *Quercus* *flocculenta* C.H.Mull.; (endemic); Red List: EN. |
| *Quercus* *frutex* Trel.; CHIH, CDMX, GTO, HGO, JAL, MEX, MICH, OAX, PUE, QRO, SLP, SON, TLAX, VER, ZAC (endemic); Red List: LC; useful (MATERIALS, FUELS). |
| *Quercus* *fulva* Liebm.; AGS, CHIH, COAH, DGO, GTO, JAL, MICH, NAY, NLE, OAX, QRO, SLP, SIN, SON, ZAC (endemic); Red List: LR/lc. |
| *Quercus* *furfuracea* Liebm.; MICH, OAX, PUE, QRO, SLP, TAMS (endemic). |
| *Quercus* *fusiformis* Small; COAH, NLE, TAMS, VER; Red List: LC. |
| *Quercus* *gambelii* Nutt.; CHIH, COAH, NLE, SON, VER; Red List: LC. |
| *Quercus* *gentryi* C.H.Mull.; AGS, CHIH, COL, DGO, GTO, JAL, MICH, NAY, QRO, SIN, ZAC (endemic); Red List: LC. |
| *Quercus* *germana* Schltdl. & Cham.; COAH, GTO, HGO, JAL, NLE, OAX, PUE, QRO, SLP, TAMS, VER (endemic); Red List: LC. |
| *Quercus* *glabrescens* Benth.; CHIS, CDMX, GTO, GRO, HGO, JAL, MEX, MICH, MOR, OAX, PUE, QRO, SLP, SIN, TLAX, VER, ZAC; Red List: LC; useful (MATERIALS, FUELS). |
| *Quercus* *glaucescens* Bonpl.; COL, GRO, JAL, MEX, MICH, NAY, OAX, QRO, SLP, SIN, TAMS, VER (endemic). |
| *Quercus* *glaucoides* M.Martens & Galeotti; CHIS, CHIH, COAH, COL, CDMX, DGO, GTO, GRO, HGO, JAL, MEX, MICH, MOR, NLE, OAX, PUE, QRO, SLP, SIN, SON, TAMS, TLAX, VER; Red List: LC; useful (ANIMAL FOOD, MATERIALS, FUELS). |
| *Quercus* *grahamii* Benth.; GRO, HGO, JAL, MEX, MICH, OAX, PUE (endemic); Red List: DD. |
| *Quercus* *gravesii* Sudw.; CHIH, COAH, COL, JAL, NLE; Red List: LR/lc. |
| *Quercus* *greggii* (A.DC.) Trel.; COAH, DGO, HGO, JAL, MEX, MICH, NAY, NLE, OAX, PUE, QRO, SLP, TAMS, TLAX, VER (endemic); Red List: LC; useful (MATERIALS, FUELS). |
| *Quercus* *grisea* Liebm.; AGS, CHIH, COAH, DGO, GTO, HGO, JAL, NLE, OAX, QRO, SLP, SIN, SON, TAMS, VER, ZAC; Red List: LC. |
| *Quercus* *hintonii* E.F.Warb.; GRO, MEX, MICH (endemic); Red List: EN. |
| *Quercus* *hintoniorum* Nixon & C.H.Müll.; COAH, NLE, SLP, TAMS (endemic); Red List: VU. |
| *Quercus* *hirtifolia* M.L.Vázquez, S.Valencia & Nixon; HGO, PUE, VER (endemic); Red List: EN. |
| *Quercus* *hypoleucoides* A.Camus; CHIH, COAH, DGO, MEX, SIN, SON, ZAC; Red List: LC. |
| *Quercus* *hypoxantha* Trel.; CHIH, COAH, GTO, NLE, QRO, SLP (endemic); Red List: DD. |
| *Quercus* *ignaciensis* C.H.Mull.; SON (endemic). |
| *Quercus* *iltisii* L.M.González; COL, JAL (endemic). |
| *Quercus* *insignis* M.Martens & Galeotti; CHIS, COL, GTO, GRO, HGO, JAL, MEX, MICH, NAY, OAX, PUE, VER; Red List: EN. |
| *Quercus* *intricata* Trel.; CHIH, COAH, DGO, NLE, OAX, SLP, TAMS, ZAC; Red List: LC. |
| *Quercus* *invaginata* Trel.; COAH, NLE, SLP (endemic); Red List: DD. |
| *Quercus* *jonesii* Trel.; (endemic); Red List: LC. |
| *Quercus* *laceyi* Small; COAH, NLE, SLP, TAMS (endemic); Red List: LC. |
| *Quercus* *laeta* Liebm.; AGS, CHIH, COAH, COL, CDMX, DGO, GTO, GRO, HGO, JAL, MEX, MICH, MOR, NAY, NLE, OAX, PUE, QRO, SLP, SIN, SON, TAMS, TLAX, VER, ZAC (endemic); Red List: LC; banked; useful (MATERIALS, FUELS). |
| *Quercus* *lancifolia* Schltdl. & Cham.; CHIS, COL, HGO, JAL, MEX, NAY, OAX, PUE, SLP, TAMS, VER; Red List: LC. |
| *Quercus* *laurina* Bonpl.; AGS, CHIS, COAH, COL, CDMX, DGO, GTO, GRO, HGO, JAL, MEX, MICH, MOR, NAY, NLE, OAX, PUE, QRO, SLP, SIN, TAMS, TLAX, VER, ZAC; Red List: LC; useful (ANIMAL FOOD, MEDICINES, MATERIALS, FUELS). |
| *Quercus* *liebmannii* Oerst. ex Trel.; GRO, MEX, MICH, OAX, PUE (endemic); Red List: LR/lc; useful (MATERIALS, FUELS). |
| *Quercus* *macdougallii* Martínez; OAX (endemic); Red List: VU. |
| *Quercus* *magnoliifolia* Née; AGS, BCS, CHIS, COAH, COL, CDMX, DGO, GTO, GRO, HGO, JAL, MEX, MICH, MOR, NAY, OAX, PUE, QRO, SLP, SIN, TAMS, VER, ZAC; Red List: LC; useful (ANIMAL FOOD, MATERIALS, FUELS). |
| *Quercus* *martinezii* C.H.Mull.; AGS, CHIS, COL, GTO, GRO, JAL, MEX, MICH, NAY, OAX, QRO, SLP (endemic); Red List: LR/lc. |
| *Quercus* *mcvaughii* Spellenb.; CHIH, DGO, JAL, SLP, SIN, SON (endemic); Red List: NT. |
| *Quercus mexicana* Bonpl.; CHIS, COAH, CDMX, GTO, HGO, JAL, MEX, MICH, MOR, NAY, NLE, OAX, PUE, QRO, SLP, SIN, TAMS, TLAX, VER (endemic); Red List: LC; useful (MATERIALS, FUELS). |
| *Quercus* *microphylla* Née; AGS, CHIH, COAH, CDMX, DGO, GTO, HGO, JAL, MEX, NAY, NLE, OAX, PUE, QRO, SLP, SIN, TAMS, TLAX, VER, ZAC (endemic); Red List: LC; useful (MATERIALS, FUELS). |
| *Quercus* *miquihuanensis* Nixon & C.H.Müll.; NLE, TAMS (endemic); Red List: EN. |
| *Quercus* *muehlenbergii* Engelm.; CHIH, COAH, NLE, SON, TAMS; Red List: LC. |
| *Quercus* *nixoniana* S.Valencia & Lozada-Pérez; COL, GRO, JAL, OAX (endemic). |
| *Quercus* *oblongifolia* Torr.; BCN, BCS, CHIH, COAH, DGO, SIN, SON, ZAC; Red List: LC. |
| *Quercus* *obtusata* Bonpl.; AGS, CHIS, COL, CDMX, DGO, GTO, GRO, HGO, JAL, MEX, MICH, MOR, NAY, NLE, OAX, PUE, QRO, SLP, SIN, TAMS, TLAX, VER, ZAC (endemic); Red List: LC; useful (ANIMAL FOOD, MATERIALS, FUELS). |
| *Quercus* *oleoides* Schltdl. & Cham.; CHIS, HGO, NLE, OAX, PUE, QRO, SLP, TAB, TAMS, VER, YUC; Red List: NT; useful (MEDICINES, FUELS). |
| *Quercus* *opaca* Trel.; HGO, NLE, SLP, TAMS, VER (endemic); Red List: DD. |
| *Quercus* *pachucana* Zav.-Cháv.; (endemic). |
| *Quercus* *palmeri* Engelm.; BCN, BCS, CHIH, SON. |
| *Quercus* *peduncularis* Née; CHIS, COL, GTO, GRO, HGO, JAL, MEX, MICH, NAY, OAX, PUE, QRO, SLP, SIN, VER, ZAC; Red List: LC; useful (FUELS, ENVIRONMENTAL USES). |
| *Quercus* *peninsularis* Trel.; BCN (endemic); Red List: DD. |
| *Quercus* *perpallida* Trel.; CHIH, SON (endemic); Red List: DD. |
| *Quercus* *pinnativenulosa* C.H.Mull.; COAH, HGO, NLE, OAX, QRO, SLP, TAMS, VER (endemic). |
| *Quercus* *planipocula* Trel.; COL, GRO, JAL, MEX, MICH, NAY, OAX, SLP, SIN, TAMS (endemic); Red List: LR/lc. |
| *Quercus* *polymorpha* Schltdl. & Cham.; CHIS, COAH, GTO, HGO, JAL, MEX, MICH, MOR, NLE, OAX, PUE, QRO, SLP, SIN, TAMS, VER; Red List: LC; useful (MATERIALS, FUELS). |
| *Quercus* *potosina* Trel.; AGS, CHIH, COAH, COL, DGO, GTO, HGO, JAL, MEX, NLE, OAX, PUE, QRO, SLP, TAMS, VER, ZAC (endemic); Red List: LC. |
| *Quercus* *praeco* Trel.; COL, DGO, HGO, JAL, NAY, QRO, SLP, ZAC (endemic); Red List: LR/lc. |
| *Quercus* *pringlei* Seemen ex Loes.; CHIH, COAH, DGO, HGO, NLE, QRO, SLP, TAMS, VER, ZAC (endemic); Red List: LC. |
| *Quercus* *pungens* Liebm.; CHIH, COAH, DGO, GTO, NLE, QRO, SLP, SON, TAMS; Red List: LC. |
| *Quercus* *purulhana* Trel.; CHIS; Red List: VU. |
| *Quercus* *resinosa* Liebm.; AGS, COL, DGO, GTO, GRO, HGO, JAL, MEX, MICH, NAY, OAX, PUE, QRO, SLP, SIN, SON, TAMS, ZAC (endemic); Red List: LC. |
| *Quercus* *rugosa* Née; AGS, BCS, CHIS, CHIH, COAH, COL, CDMX, DGO, GTO, GRO, HGO, JAL, MEX, MICH, MOR, NAY, NLE, OAX, PUE, QRO, SLP, SIN, SON, TAMS, TLAX, VER, ZAC; Red List: LC; useful (FOOD, ANIMAL FOOD, MEDICINES, MATERIALS, FUELS). |
| *Quercus* *runcinatifolia* Trel. & C.H.Müll.; NLE (endemic). |
| *Quercus* *rysophylla* Weath.; (endemic); Red List: DD. |
| *Quercus* *salicifolia* Née; CHIS, COL, DGO, GRO, HGO, JAL, MEX, MICH, MOR, NAY, OAX, SLP, SIN, VER, ZAC; Red List: LC; useful (ANIMAL FOOD, MATERIALS). |
| *Quercus* *saltillensis* Trel.; COAH, NLE, SLP, TAMS, ZAC (endemic). |
| *Quercus* *sapotifolia* Liebm.; CHIS, GRO, HGO, OAX, PUE, SLP, TAMS, VER; Red List: LC; useful (MATERIALS, FUELS, ENVIRONMENTAL USES). |
| *Quercus* *scytophylla* Liebm.; CHIS, CHIH, COL, DGO, GRO, HGO, JAL, MEX, MICH, NAY, OAX, PUE, SIN, SON, VER, ZAC (endemic); Red List: LC. |
| *Quercus* *sebifera* Trel.; CHIS, CHIH, DGO, HGO, NLE, OAX, PUE, QRO, SLP, SON, TAMS, VER (endemic); Red List: LR/lc; useful (MATERIALS, FUELS). |
| *Quercus* *segoviensis* Liebm.; CHIS, MICH, OAX; Red List: LC. |
| *Quercus* *sideroxyla* Bonpl.; AGS, CHIH, COAH, DGO, GTO, HGO, JAL, MEX, MICH, MOR, NAY, NLE, OAX, PUE, QRO, SLP, SIN, SON, TAMS, VER, ZAC (endemic); Red List: LC. |
| *Quercus* *sinuata* Walter; COAH, NLE, TAMS; Red List: LC. |
| *Quercus* *skinneri* Benth.; CHIS, HGO, OAX, TAMS, VER; Red List: VU; useful (MATERIALS, FUELS). |
| *Quercus* *splendens* Née; COL, DGO, GRO, JAL, MEX, MICH, MOR, NAY, OAX, SIN, TAMS, VER, ZAC (endemic); Red List: LC; useful (FOOD, ANIMAL FOOD, MEDICINES, MATERIALS, FUELS). |
| *Quercus* *striatula* Trel.; (endemic); Red List: LC. |
| *Quercus* *subspathulata* Trel.; CHIH, COL, DGO, GTO, GRO, HGO, JAL, MEX, MICH, NAY, OAX, PUE, QRO, SIN, SON, ZAC (endemic); Red List: LC. |
| *Quercus* *tarahumara* Spellenb., J.D.Bacon & Breedlove; CHIH, DGO, SIN, SON (endemic); Red List: LC. |
| *Quercus* *tardifolia* C.H.Mull.; Red List: DD. |
| *Quercus* *tinkhamii* C.H.Mull.; COAH, DGO, GTO, HGO, JAL, NLE, QRO, SLP, TAMS, VER, ZAC (endemic). |
| *Quercus* *tomentella* Jord.; BCN; Red List: EN. |
| *Quercus* *toumeyi* Sarg.; CHIH, DGO, NLE, SIN, SON; Red List: LR/lc. |
| *Quercus* *tuberculata* Liebm.; BCS, CHIH, COAH, DGO, JAL, MEX, MICH, NAY, NLE, SIN, SON, ZAC (endemic); Red List: LC. |
| *Quercus* *tuitensis* L.M.González; JAL (endemic). |
| *Quercus* *turbinella* Greene; BCN, BCS, SON; Red List: LC. |
| *Quercus* *undata* Trel.; (endemic); Red List: DD. |
| *Quercus* *urbani* Trel.; CHIH, DGO, GRO, JAL, MEX, MICH, NAY, OAX, QRO, SIN, SON, ZAC (endemic); Red List: LC; useful (MATERIALS, FUELS). |
| *Quercus* *uxoris* McVaugh; AGS, COL, GRO, JAL, MICH, OAX (endemic); Red List: LC. |
| *Quercus* *vaseyana* Buckley; COAH, NLE, TAMS (endemic); Red List: LC. |
| *Quercus* *verde* C.H.Mull.; CHIH, COAH, NLE (endemic). |
| *Quercus* *vicentensis* Trel.; CHIS, COL, GRO, JAL, MICH, OAX (endemic); Red List: VU. |
| *Quercus* *viminea* Trel.; AGS, CHIH, DGO, GTO, JAL, NAY, SLP, SIN, SON, ZAC (endemic); Red List: LC. |
| *Quercus* *wislizeni* A.DC.; BCN; Red List: LC. |
| *Quercus* *xalapensis* Bonpl.; CHIS, CHIH, COL, DGO, GRO, HGO, JAL, MEX, MICH, NAY, OAX, PUE, QRO, SLP, SIN, SON, TAMS, VER (endemic); Red List: LC; useful (MATERIALS, FUELS, ENVIRONMENTAL USES). |
| *Quercus* *xylina* Scheidw.; COL, CDMX, DGO, GTO, HGO, JAL, MEX, MICH, NAY, SLP, SIN (endemic); Red List: NT. |
|  |
| **Fouquieriaceae** |
| ***Fouquieria*** |
| *Fouquieria* *columnaris* (Kellogg) Kellogg ex Curran; BCN, BCS, SON (endemic); Red List: VU; Cites: II; banked. |
| *Fouquieria* *diguetii* (Tiegh.) I.M.Johnst.; BCN, BCS, SIN, SON (endemic); Red List: VU; banked. |
| *Fouquieria* *fasciculata* (Willd. ex Roem. & Schult.) Nash; HGO, QRO, SLP, VER (endemic); Red List: VU; NOM-59: A; Cites: I; banked. |
| *Fouquieria* *formosa* Kunth; CHIS, COL, CDMX, GRO, HGO, JAL, MEX, MICH, MOR, OAX, PUE, QRO (endemic); Red List: LC; banked; useful (ANIMAL FOOD, MEDICINES, MATERIALS, FUELS, ENVIRONMENTAL USES). |
| *Fouquieria* *leonilae* Miranda; GRO, OAX (endemic); Red List: EN. |
| *Fouquieria* *macdougalii* Nash; CHIH, SIN, SON (endemic); Red List: LC; banked. |
| *Fouquieria* *ochoterenae* Miranda; OAX, PUE (endemic); Red List: EN; banked; useful. |
| *Fouquieria* *purpusii* Brandegee; OAX, PUE (endemic); Red List: EN; Cites: I; banked; useful (ANIMAL FOOD, FUELS, ENVIRONMENTAL USES). |
| *Fouquieria* *splendens* Engelm.; BCN, BCS, CHIH, COAH, DGO, GTO, HGO, JAL, NLE, QRO, SLP, SON, TAMS, VER, ZAC; banked; useful (MEDICINES). |
|  |
| **Garryaceae** |
| ***Garrya*** |
| *Garrya* *glaberrima* Wangerin; COAH, GTO, JAL, NLE, QRO, SLP, TAMS (endemic). |
| *Garrya* *laurifolia* Benth.; AGS, CHIS, CHIH, COAH, COL, CDMX, DGO, GTO, GRO, HGO, JAL, MEX, MICH, MOR, NAY, NLE, OAX, PUE, QRO, SLP, SIN, TAMS, SON, TLAX, VER, ZAC; banked; useful (ANIMAL FOOD). |
| *Garrya* *longifolia* Rose; CHIS, COL, CDMX, DGO, GTO, GRO, JAL, MEX, MICH, MOR, NAY, OAX, PUE, SIN, ZAC (endemic). |
| *Garrya* *ovata* Benth.; AGS, CHIH, COAH, DGO, GTO, HGO, JAL, NLE, OAX, PUE, QRO, SLP, SON, TAMS, VER, ZAC; banked; useful (MEDICINES, FUELS). |
|  |
| **Hamamelidaceae** |
| ***Hamamelis*** |
| *Hamamelis virginiana* var. *mexicana* (Standl.) C.Lane; COAH, NLE (endemic); Red List: LC. |
| ***Matudaea*** |
| *Matudaea trinervia* Lundell; CHIS, COL, JAL, MEX, OAX, PUE, VER; Red List: VU; NOM-59: A. |
| ***Molinadendron*** |
| *Molinadendron sinaloense* (Standl. & Gentry) P.K.Endress; SIN (endemic). |
|  |
| **Hernandiaceae** |
| ***Gyrocarpus*** |
| *Gyrocarpus americanus* Jacq.; CHIS, OAX, PUE; Red List: LC; useful (MEDICINES, MATERIALS). |
| *Gyrocarpus jatrophifolius* Domin; CAM, CHIS, COL, DGO, GRO, JAL, MEX, MICH, MOR, NAY, OAX, PUE, QROO, SIN, VER, YUC, ZAC; banked; useful (MATERIALS, ENVIRONMENTAL USES). |
| *Gyrocarpus mocinoi* Espejo; CHIS, OAX, PUE; Red List: VU; banked; useful. |
| ***Hernandia*** |
| *Hernandia didymantha* Donn.Sm.; CHIS; Red List: NT; useful (ENVIRONMENTAL USES). |
| *Hernandia stenura* Standl.; CHIS, VER; Red List: NT. |
| *Hernandia wendtii* Espejo; CHIS, OAX, PUE, VER. |
|  |
| **Hypericaceae** |
| ***Vismia*** |
| *Vismia baccifera* (L.) Planch. & Triana; CHIS, GRO, OAX, PUE, TAB, VER; Red List: LC; banked; useful (ANIMAL FOOD, MEDICINES, ENVIRONMENTAL USES). |
| *Vismia camparaguey* Sprague & L.Riley; CHIS, OAX, PUE, TAB, VER; Red List: LC. |
|  |
| **Icacinaceae** |
| ***Calatola*** |
| *Calatola costaricensis* Standl.; CHIS, OAX, VER; Red List: LC; useful (ANIMAL FOOD, MEDICINES, ENVIRONMENTAL USES). |
| *Calatola laevigata* Standl.; CHIS, COL, JAL, OAX, PUE, VER; banked. |
| *Calatola mollis* Standl.; CHIS, OAX, PUE, VER (endemic). |
| *Calatola uxpanapensis* Vera-Cal. & T.Wendt; VER. |
| ***Mappia*** |
| *Mappia mexicana* B.L.Rob. & Greenm.; SLP, TAMS, VER (endemic). |
| *Mappia racemosa* Jacq.; CHIS, OAX, PUE, TAB, VER; Red List: VU. |
| ***Oecopetalum*** |
| *Oecopetalum greenmanii* Standl. & Steyerm.; CHIS, TAB. |
| *Oecopetalum mexicanum* Greenm. & C.H.Thomps.; CHIS, OAX, TAB, VER; banked. |
| ***Ottoschulzia*** |
| *Ottoschulzia pallida* Lundell; CAM, CHIS, QROO. |
|  |
| **Iteaceae** |
| ***Pterostemon*** |
| *Pterostemon rotundifolius* Ramírez; OAX, PUE (endemic); Red List: LC; banked; useful (FOOD, ANIMAL FOOD, FUELS). |
|  |
| **Juglandaceae** |
| ***Alfaroa*** |
| *Alfaroa costaricensis* Standl.; CHIS, VER; Red List: LC. |
| *Alfaroa guatemalensis* (Standl.) L.O.Williams & A.R.Molina; CHIS. |
| ***Carya*** |
| *Carya ovata* (Mill.) K.Koch; COAH, GTO, HGO, MICH, NLE, PUE, QRO, SLP, TAMS, VER; Red List: LC. |
| *Carya palmeri* W.E.Manning; COAH, HGO, NLE, OAX, QRO, SLP, TAMS, VER (endemic); banked. |
| ***Juglans*** |
| *Juglans hirsuta* W.E.Manning; COAH, HGO, JAL, NLE, PUE, QRO, SLP, VER, ZAC (endemic). |
| *Juglans major* (Torr.) A.Heller; CHIH, COAH, COL, DGO, GTO, GRO, HGO, JAL, MEX, MICH, MOR, NAY, NLE, OAX, QRO, SIN, SON, TAMS, VER; Red List: LC; NOM-59: A. |
| *Juglans microcarpa* Berland.; CHIH, COAH, NLE; Red List: LC. |
| *Juglans mollis* Engelm.; COAH, GTO, GRO, HGO, JAL, MEX, MOR, NLE, OAX, PUE, QRO, SLP, TAMS, VER (endemic); banked; useful (MEDICINES, MATERIALS). |
| *Juglans pyriformis* Liebm.; CHIS, CHIH, DGO, GRO, HGO, JAL, MEX, MICH, OAX, PUE, SON, TAMS, VER (endemic). |
| ***Oreomunnea*** |
| *Oreomunnea mexicana* (Standl.) J.-F.Leroy; CHIS, OAX, VER. |
|  |
| **Lacistemataceae** |
| ***Lacistema*** |
| *Lacistema aggregatum* (P.J.Bergius) Rusby; CHIS, GRO, OAX, PUE, SIN, TAB, TAMS, VER; banked. |
|  |
| **Lamiaceae** |
| ***Aegiphila*** |
| *Aegiphila costaricensis* Moldenke; CHIS, OAX, TAB, VER; Red List: LC. |
| ***Callicarpa*** |
| *Callicarpa acuminata* Kunth; CAM, CHIS, COAH, HGO, JAL, MICH, NLE, OAX, PUE, QRO, QROO, SLP, TAB, TAMS, VER, YUC; Red List: LC; useful (MEDICINES). |
| ***Salvia*** |
| *Salvia sessei* Benth.; COL, CDMX, GRO, JAL, MEX, MICH, MOR, OAX, PUE, TAMS (endemic); banked; useful (MEDICINES). |
| ***Vitex*** |
| *Vitex* *gaumeri* Greenm.; CAM, CHIS, GRO, MEX, MICH, MOR, QROO, SIN, TAB, VER, YUC; Red List: EN; useful (MATERIALS). |
| *Vitex* *hemsleyi* Briq.; CHIS, COL, GRO, JAL, MEX, MICH, MOR, NAY, OAX. |
| *Vitex* *mollis* Kunth; BCN, BCS, CHIH, COL, DGO, GRO, JAL, MEX, MICH, MOR, NAY, OAX, PUE, SIN, SON, VER, ZAC (endemic); banked; useful (FOOD, ANIMAL FOOD, MEDICINES). |
| *Vitex* *pyramidata* B.L.Rob.; CHIS, CHIH, COL, DGO, GRO, JAL, MEX, MICH, MOR, NAY, OAX, PUE, SIN, SON, YUC, ZAC (endemic); banked; useful (MEDICINES). |
|  |
| **Lauraceae** |
| ***Aiouea*** |
| *Aiouea* *amplexicaulis* (Schltdl. & Cham.) R.Rohde; GRO, OAX, VER (endemic). |
| *Aiouea* *areolata* (Lundell) R.Rohde; CHIS, OAX. |
| *Aiouea* *bractefoliacea* (Lorea-Hern.) R.Rohde; QRO, SLP, TAMS (endemic). |
| *Aiouea* *breedlovei* (Lundell) R.Rohde; CHIS, OAX (endemic). |
| *Aiouea* *chiapensis* (Lundell) R.Rohde; CHIS (endemic). |
| *Aiouea* *cinnamomoidea* (Lorea-Hern.) R.Rohde & Lorea-Hern.; GRO, OAX (endemic). |
| *Aiouea* *effusa* (Meisn.) R.Rohde & Rohwer; HGO, OAX, PUE, QRO, SLP, TAMS, VER. |
| *Aiouea* *elegans* (van der Werff) Rohwer; CHIS, GRO, OAX (endemic). |
| *Aiouea* *glossophylla* (Lorea-Hern.) R.Rohde; DGO, NAY (endemic). |
| *Aiouea* *grisebachii* (Lorea-Hern.) Rohwer; CHIS, OAX, TAB, VER. |
| *Aiouea hartmanii* (I.M.Johnst.) R.Rohde; CHIH, COL, DGO, JAL, MICH, NAY, SON (endemic). |
| *Aiouea* *inconspicua* van der Werff; CHIS, OAX, TAB, VER (endemic). |
| *Aiouea* *leptophylla* (Lorea-Hern.) R.Rohde; VER (endemic). |
| *Aiouea* *longipes* (I.M.Johnst.) R.Rohde; HGO, PUE, VER (endemic). |
| *Aiouea* *maya* Lorea-Hern.; CHIS. |
| *Aiouea* *montana* (Sw.) R.Rohde. |
| *Aiouea* *neurophylla* (Mez & Pittier) R.Rohde; OAX, TAB. |
| *Aiouea* *pachypoda* (Nees) R.Rohde; COAH, COL, GTO, GRO, HGO, JAL, MEX, MICH, NAY, NLE, OAX, PUE, QRO, SLP, TAMS, VER (endemic). |
| *Aiouea* *padiformis* (Standl. & Steyerm.) R.Rohde; GRO, JAL. |
| *Aiouea* *salicifolia* (Nees) R.Rohde; GTO, HGO, QRO, SLP, VER (endemic). |
| *Aiouea* *zapatae* (Lorea-Hern.) R.Rohde; CHIS, GRO, OAX. |
| ***Beilschmiedia*** |
| *Beilschmiedia* *anay* (S.F.Blake) Kosterm.; CHIS, OAX, PUE, VER. |
| *Beilschmiedia* *angustielliptica* Lorea-Hern.; GRO (endemic). |
| *Beilschmiedia* *hondurensis* Kosterm.; CHIS, OAX, VER. |
| *Beilschmiedia manantlanensis* Cuevas & Cochrane; COL, JAL (endemic). |
| *Beilschmiedia mexicana* (Mez) Kosterm.; CHIS, GRO, HGO, OAX, PUE, QRO, SLP, VER. |
| *Beilschmiedia* *ovalioides* Sachiko Nishida; CHIS, OAX. |
| *Beilschmiedia* *ovalis* (S.F.Blake) C.K.Allen; CHIS, OAX. |
| *Beilschmiedia* *riparia* Miranda; CHIS, GRO, JAL, MICH, SLP, OAX, VER. |
| *Beilschmiedia* *steyermarkii* C.K.Allen; CHIS. |
| *Beilschmiedia* *zapoteoides* (Lundell) Kosterm.; CHIS (endemic). |
| ***Damburneya*** |
| *Damburneya* *ambigens* (S.F.Blake) Trofimov; CHIS, CHIH, GRO, MICH, NAY, OAX, TAB, VER. |
| *Damburneya* *coriacea* (Sw.) Trofimov & Rohwer; CAM, CHIS, OAX, QROO, SLP, TAB, TAMS, VER, YUC. |
| *Damburneya* *gentlei* (Lundell) Trofimov; CHIS, OAX, PUE, TAB, VER. |
| *Damburneya* *leucocome* (Rohwer) Trofimov & Rohwer; CHIS (endemic); Red List: EN. |
| *Damburneya* *longicaudata* (Lundell) Trofimov & Rohwer; CAM, CHIS, OAX, VER. |
| *Damburneya* *martinicensis* (Mez) Trofimov; CHIS, COL, GRO, JAL, TAB. |
| *Damburneya* *matudae* (Lundell) Trofimov & Rohwer; CHIS, OAX, VER ; Red List: VU. |
| *Damburneya* *nitida* (Mez) Trofimov & Rohwer; CHIS, GRO, OAX, QROO, TAB, VER. |
| *Damburneya* *purpurea* (Ruiz & Pav.) Trofimov; CHIS. |
| *Damburneya* *rudis* (C.K.Allen) Trofimov & Rohwer; CHIS; Red List: VU. |
| *Damburneya* *salicifolia* (Kunth) Trofimov & Rohwer; CAM, CHIS, COL, GRO, HGO, JAL, MEX, MICH, MOR, NAY, NLE, OAX, PUE, QRO, QROO, SLP, SIN, TAB, TAMS, VER, YUC. |
| *Damburneya* *salicina* (C.K.Allen) Trofimov & Rohwer; CHIS; Red List: NT. |
| ***Licaria*** |
| *Licaria* *brittoniana* C.K.Allen & L.E.Greg.; CHIS, OAX. |
| *Licaria* *campechiana* (Standl.) Kosterm.; CAM, CHIS, QRO, QROO, SLP, VER, YUC. |
| *Licaria* *capitata* (Cham. & Schltdl.) Kosterm.; CHIS, GRO, HGO, OAX, PUE, QRO, SLP, TAB, VER. |
| *Licaria* *chinanteca* Lorea-Hern.; OAX (endemic). |
| *Licaria* *excelsa* Kosterm.; CHIS, GRO, OAX, PUE, VER. |
| *Licaria* *glaberrima* (Lundell) C.K.Allen; CHIS (endemic). |
| *Licaria mexicana* (Brandegee) Kosterm.; HGO, VER (endemic). |
| *Licaria* *misantlae* (Brandegee) Kosterm.; CAM, CHIS, OAX, QROO, VER. |
| *Licaria* *multinervis* H.W.Kurz; CHIS. |
| *Licaria* *peckii* (I.M.Johnst.) Kosterm.; CAM, CHIS, OAX, QROO, TAB, VER; Red List: LC. |
| *Licaria* *phymatosa* Lorea-Hern.; GRO (endemic). |
| *Licaria* *quercina* Lorea-Hern.; GRO (endemic). |
| *Licaria* *siphonantha* Lorea-Hern.; GRO (endemic). |
| *Licaria* *triandra* (Sw.) Kosterm. |
| *Licaria* *urceolata* Lundell; GRO, OAX, QRO. |
| *Licaria* *velutina* van der Werff; CHIS, OAX, VER (endemic); Red List: VU. |
| ***Litsea*** |
| *Litsea glaucescens* Kunth; AGS, CHIS, CHIH, COAH, COL, CDMX, DGO, GTO, GRO, HGO, JAL, MEX, MICH, MOR, NAY, NLE, OAX, PUE, QRO, SLP, SIN, SON, TAB, TAMS, VER, ZAC; NOM-59: P; banked; useful (FOOD, MEDICINES, MATERIALS, ENVIRONMENTAL USES). |
| *Litsea parvifolia* (Hemsl.) Mez; COAH, NLE, SLP (endemic). |
| *Litsea pringlei* Bartlett; COAH, HGO, NLE, SLP, TAMS, VER (endemic). |
| ***Nectandra*** |
| *Nectandra* *belizensis* (Lundell) C.K.Allen; CHIS. |
| *Nectandra* *cissiflora* Nees; CHIS, VER. |
| *Nectandra* *cuspidata* Nees & Mart.; CHIS, OAX, PUE, TAB, VER. |
| *Nectandra* *hihua* (Ruiz & Pav.) Rohwer; CAM, CHIS, CHIH, COL, DGO, GRO, HGO, JAL, MEX, MICH, MOR, NAY, NLE, OAX, PUE, SIN, SON, TAB, TAMS, VER; Red List: LC. |
| *Nectandra* *rubriflora* (Mez) C.K.Allen; CHIS, OAX, PUE, TAB, VER. |
| *Nectandra* *turbacensis* (Kunth) Nees; CHIS, OAX, VER. |
| *Nectandra* *villosa* Nees & Mart.; CHIS, HGO, OAX, PUE, SLP, TAB, VER; useful (MATERIALS). |
| ***Ocotea*** |
| *Ocotea* *acuminatissima* (Lundell) Rohwer; CHIS, GRO, JAL, NAY, OAX, VER. |
| *Ocotea* *atacta* Lorea-Hern.; GRO, OAX (endemic). |
| *Ocotea* *bernoulliana* Mez; CAM, CHIS, GRO, OAX, TAB, VER. |
| *Ocotea* *betazensis* (Mez) van der Werff; CHIS, OAX (endemic). |
| *Ocotea* *botrantha* Rohwer; CHIS, OAX. |
| *Ocotea* *bourgeauviana* (Mez) van der Werff; CHIS, HGO, OAX, TAB, VER. |
| *Ocotea* *candidovillosa* Lorea-Hern.; GRO, OAX (endemic). |
| *Ocotea* *cernua* (Nees) Mez; CAM, CHIS, OAX, SIN, TAB, VER; Red List: LC; useful (MATERIALS). |
| *Ocotea* *chiapensis* (Lundell) Standl. & Steyerm.; CHIS, GRO, OAX, VER. |
| *Ocotea* *chrysobalanoides* (Lundell) Lundell; CHIS (endemic). |
| *Ocotea* *congregata* van der Werff; CHIS (endemic). |
| *Ocotea* *corrugata* van der Werff; OAX, VER (endemic). |
| *Ocotea* *dendrodaphne* Mez; CHIS, OAX, TAB, VER; Red List: LC. |
| *Ocotea* *disjuncta* Lorea-Hern.; GRO, OAX, VER (endemic). |
| *Ocotea* *heribertoi* T.Wendt; CHIS, OAX, VER (endemic). |
| *Ocotea* *heydeana* (Mez & Donn.Sm.) Bernardi; CHIS, OAX. |
| *Ocotea* *iridescens* Lorea-Hern. & van der Werff; OAX (endemic). |
| *Ocotea* *klotzschiana* (Nees) Hemsl.; HGO, OAX, PUE, QRO, SLP, VER (endemic). |
| *Ocotea laetevirens* Standl. & Steyerm.; CHIS, OAX, VER. |
| *Ocotea* *leucoxylon* (Sw.) Laness.; CHIS, OAX, PUE, VER. |
| *Ocotea* *macrophylla* Kunth; CHIS, GRO, HGO, OAX, PUE, TAB, VER; banked. |
| *Ocotea* *magnifolia* (Lundell) Lundell; CHIS, OAX, TAB, VER. |
| *Ocotea* *matudae* Lundell; CHIS (endemic). |
| *Ocotea* *oblonga* (Meisn.) Mez; CHIS, OAX; useful (ENVIRONMENTAL USES). |
| *Ocotea* *parvula* (Lundell) van der Werff; CHIS, OAX, VER (endemic). |
| *Ocotea* *platyphylla* (Lundell) Rohwer; CHIS, OAX, VER. |
| *Ocotea* *psychotrioides* Kunth; GRO, HGO, JAL, OAX, PUE, QRO, SLP, VER (endemic). |
| *Ocotea* *puberula* (Rich.) Nees; CHIS, HGO, OAX, PUE, VER; Red List: LR/lc. |
| *Ocotea* *purpurea* (Mez) van der Werff; CHIS, OAX. |
| *Ocotea* *rovirosae* Lorea-Hern. & van der Werff; OAX, TAB, VER (endemic). |
| *Ocotea* *salvinii* Mez; CAM, CHIS, HGO, QRO, SLP, TAB, TAMS, VER, YUC. |
| *Ocotea* *sarcodes* Lorea-Hern.; CHIS, OAX (endemic). |
| *Ocotea* *sauroderma* Lorea-Hern.; OAX (endemic). |
| *Ocotea* *sinuata* (Mez) Rohwer; CHIS, OAX. |
| *Ocotea* *standleyi* C.K.Allen; CHIS, OAX, VER. |
| *Ocotea* *subalata* Lundell; CHIS. |
| *Ocotea* *subtriplinervia* (Meisn.) Hemsl.; VER (endemic). |
| *Ocotea* *tampicensis* (Meisn.) Hemsl.; HGO, NLE, OAX, QRO, SLP, TAMS, VER (endemic). |
| *Ocotea* *tonii* (Lundell) van der Werff; CHIS, GRO, OAX (endemic). |
| *Ocotea* *truncata* Lundell; CHIS. |
| *Ocotea* *uxpanapana* T.Wendt & van der Werff; CHIS, OAX, VER (endemic); Red List: VU. |
| *Ocotea* *veraguensis* (Meisn.) Mez; CHIS, DGO, GRO, JAL, MICH, NAY, OAX, PUE, QROO, SLP, SIN, VER; Red List: LC. |
| *Ocotea* *verticillata* Rohwer; OAX, VER (endemic). |
| *Ocotea* *zoque* Lorea-Hern.; OAX (endemic). |
| ***Persea*** |
| *Persea* *albida* Kosterm.; CHIS, OAX; Red List: EN. |
| *Persea* *americana* Mill.; CAM, CHIS, CHIH, COAH, COL, DGO, GRO, HGO, JAL, MEX, MICH, MOR, NAY, NLE, OAX, PUE, QRO, QROO, SLP, SIN, SON, TAB, TAMS, VER, YUC, ZAC; Red List: LC; banked; useful (FOOD, MEDICINES, MATERIALS, FUELS). |
| *Persea* *brevipetiolata* van der Werff; OAX, VER (endemic). |
| *Persea* *chamissonis* Mez; CHIS, GTO, HGO, OAX, PUE, QRO, SLP, VER (endemic); Red List: EN. |
| *Persea* *chrysantha* Lorea-Hern.; GRO, MICH (endemic). |
| *Persea* *cinerascens* S.F.Blake; OAX, PUE, VER (endemic); Red List: EN. |
| *Persea* *donnell-smithii* Mez; CHIS, GRO, MICH, OAX, TAMS, VER; Red List: VU. |
| *Persea* *hintonii* C.K.Allen; COL, GRO, HGO, JAL, MEX, MICH, NAY, PUE, SIN, TAMS, VER (endemic); Red List: VU. |
| *Persea* *liebmannii* Mez; CHIS, CHIH, COAH, DGO, GTO, GRO, HGO, JAL, MICH, NAY, NLE, OAX, PUE, QRO, SLP, SIN, SON, TAMS, VER, ZAC; Red List: LC. |
| *Persea* *longipes* (Schltdl.) Meisn.; (endemic); Red List: EN. |
| *Persea* *obscura* Lorea-Hern.; OAX (endemic). |
| *Persea* *pallescens* (Mez) Lorea-Hern.; HGO, OAX, PUE, QRO, SLP, VER; Red List: EN. |
| *Persea* *purpusii* L.E.Kopp; HGO, QRO, SLP (endemic); Red List: DD. |
| *Persea* *rufescens* Lundell; CHIS, OAX (endemic); Red List: EN. |
| *Persea* *schiedeana* Nees; CAM, CHIS, OAX, PUE, SLP, QROO, TAB, TAMS, VER; Red List: EN; useful (FOOD). |
| *Persea* *vesticula* Standl. & Steyerm.; CHIS, OAX; Red List: LC. |
| ***Phoebe*** |
| *Phoebe* *mollis* Mez; CHIS, GRO. |
| ***Umbellularia*** |
| *Umbellularia* *californica* (Hook. & Arn.) Nutt.; BCN; Red List: LC; useful (MATERIALS). |
|  |
| **Lecythidaceae** |
| ***Eschweilera*** |
| *Eschweilera mexicana* T.Wendt, S.A.Mori & Prance; OAX, VER (endemic); Red List: VU. |
|  |
| **Lythraceae** |
| ***Ginoria*** |
| *Ginoria* *nudiflora* (Hemsl.) Koehne; CHIS, MICH, OAX, VER (endemic); Red List: VU. |
|  |
| **Magnoliaceae** |
| ***Magnolia*** |
| *Magnolia* *guerrerensis* J.Jiménez Ram., K.Vega & Cruz Durán; GRO (endemic); Red List: EN. |
| *Magnolia* *iltisiana* Vazquez; COL, GRO, JAL, MICH (endemic); Red List: VU; NOM-59: A. |
| *Magnolia* *jaliscana* A.Vázquez & R.Guzmán; COL, JAL (endemic); Red List: EN. |
| *Magnolia* *krusei* J.Jiménez Ram. & Cruz Durán; GRO, OAX (endemic); Red List: EN. |
| *Magnolia* *lacandonica* A.Vázquez, Pérez-Farr. & Mart.-Camilo; CHIS; Red List: CR. |
| *Magnolia* *lopezobradorii* A.Vázquez; VER (endemic); Red List: DD. |
| *Magnolia* *mayae* A.Vázquez & Pérez-Farr.; CHIS (endemic); Red List: CR. |
| *Magnolia* *montebelloensis* A.Vázquez & Pérez-Farr.; (endemic). |
| *Magnolia* *nuevoleonensis* A.Vázquez & Domínguez-Yescas; (endemic); Red List: EN. |
| *Magnolia* *oaxacensis* A.Vázquez; OAX (endemic); Red List: EN. |
| *Magnolia* *pacifica* Vazquez; CHIH, DGO, JAL, NAY, SIN, SON, ZAC (endemic); Red List: EN. |
| *Magnolia* *perezfarrerae* A.Vázquez & Gómez-Domínguez; CHIS (endemic); Red List: EN. |
| *Magnolia* *poasana* (Pittier) Dandy; CHIS; Red List: NT; useful (ENVIRONMENTAL USES). |
| *Magnolia* *pugana* (Iltis & A.Vazquez) A.Vazquez & Carvajal; JAL, ZAC (endemic); Red List: EN. |
| *Magnolia* *rzedowskiana* A.Vázquez, Domínguez-Yescas & R.Pedraza; HGO, QRO, SLP, VER (endemic); Red List: EN. |
| *Magnolia* *schiedeana* Schltdl.; CHIS, CHIH, COL, DGO, GRO, HGO, JAL, MICH, NAY, NLE, OAX, PUE, QRO, SLP, SIN, SON, TAMS, VER, ZAC (endemic); Red List: VU; NOM-59: A. |
| *Magnolia* *sharpii* V.V.Miranda; CHIS (endemic); Red List: EN. |
| *Magnolia* *sinacacolinii* A.Vázquez; VER (endemic); Red List: DD. |
| *Magnolia* *tamaulipana* Vazquez; NLE, TAMS (endemic); Red List: EN. |
| *Magnolia* *vazquezii* Cruz Durán & K.Vega; GRO, OAX (endemic); Red List: EN. |
| *Magnolia* *yoroconte* Dandy; CHIS, VER; Red List: VU; useful (MATERIALS, ENVIRONMENTAL USES). |
| *Magnolia* *zoquepopolucae* A.Vázquez; VER (endemic); Red List: DD. |
| *Magnolia* *yajlachhi* A. Vázquez & Domínguez-Yescas; (endemic). |
|  |
| ***Malpighia*ceae** |
| ***Bunchosia*** |
| *Bunchosia* *breedlovei* W.R.Anderson; CHIS (endemic). |
| *Bunchosia* *canescens* (W.T.Aiton) DC.; CAM, CHIS, DGO, GRO, MEX, MICH, MOR, OAX, PUE, QROO, SIN (endemic). |
| *Bunchosia* *cruciana* W.R.Anderson; VER (endemic). |
| *Bunchosia* *glandulosa* (Cav.) DC.; CAM, QROO, YUC; Red List: LC; useful (MEDICINES). |
| *Bunchosia* *gracilis* Nied.; CHIS, OAX. |
| *Bunchosia* *guatemalensis* Nied.; CAM, CHIS, OAX, QROO, TAB, VER. |
| *Bunchosia* *hedraiophylla* W.R.Anderson; OAX (endemic). |
| *Bunchosia* *lindeniana* A.Juss.; CAM, CHIS, COL, DGO, GRO, HGO, JAL, MEX, MICH, MOR, OAX, PUE, QRO, QROO, SLP, SIN, TAB, TAMS, VER, YUC; Red List: LC; banked. |
| *Bunchosia* *macrophylla* Rose; CHIS, OAX. |
| *Bunchosia* *mcvaughii* W.R.Anderson; COL, GRO, JAL, NAY (endemic). |
| *Bunchosia* *nitida* (Jacq.) A.Rich.; GRO, OAX. |
| *Bunchosia* *palmeri* S.Watson; COL, DGO, GRO, JAL, MEX, MICH, MOR, NAY, OAX, PUE, SLP, SIN, TAMS, VER, ZAC (endemic); banked; useful (FOOD). |
| *Bunchosia* *praecox* W.R.Anderson; JAL, SIN (endemic). |
| *Bunchosia* *strigosa* Schltdl.; COL, JAL, NAY, OAX (endemic). |
| *Bunchosia* *swartziana* Griseb.; CAM, CHIS, QROO, TAB, VER, YUC; Red List: LC; useful (MEDICINES, SOCIAL USES). |
| ***Byrsonima*** |
| *Byrsonima crassifolia* (L.) Kunth; CAM, CHIS, COL, DGO, GRO, HGO, JAL, MEX, MICH, MOR, NAY, OAX, PUE, QROO, SLP, SIN, TAB, TAMS, VER, YUC, ZAC; Red List: LC; banked; useful (FOOD, ANIMAL FOOD, MEDICINES, MATERIALS, FUELS, ENVIRONMENTAL USES). |
| *Byrsonima oaxacana* A.Juss.; OAX (endemic). |
| ***Lasiocarpus*** |
| *Lasiocarpus ferrugineus* Gentry; COL, DGO, JAL, MOR, NAY, OAX, PUE, SIN (endemic); banked; useful. |
| *Lasiocarpus ovalifolius* Nied.; (endemic). |
| *Lasiocarpus salicifolius* Liebm.; CHIS, GRO, MOR, OAX, PUE, SIN (endemic); banked. |
| ***Malpighia*** |
| *Malpighia* *davilae* W.R.Anderson; OAX, PUE (endemic). |
| *Malpighia* *galeottiana* A.Juss.; GRO, JAL, MEX, MICH, OAX, PUE, SLP (endemic); banked; useful (FOOD). |
| *Malpighia* *glabra* L.; CAM, CHIS, COAH, GRO, HGO, JAL, MEX, NAY, NLE, OAX, PUE, QRO, QROO, SLP, SIN, SON, TAB, TAMS, VER, YUC; useful (MEDICINES). |
| *Malpighia* *incana* Mill.; CAM, YUC. |
| *Malpighia* *leticiana* (W.R.Anderson) W.R.Anderson & C.Davis; OAX (endemic). |
| *Malpighia* *lundellii* C.V.Morton; CAM, QROO, TAB, YUC. |
| *Malpighia* *macrocarpa* F.K.Mey.; CHIS, VER (endemic). |
| *Malpighia mexicana* A.Juss.; CHIS, COL, DGO, GTO, GRO, JAL, MEX, MICH, MOR, NAY, OAX, PUE, VER, ZAC (endemic); useful (FOOD, MEDICINES). |
| *Malpighia* *novogaliciana* W.R.Anderson; COL, JAL, NAY, OAX (endemic). |
| *Malpighia* *rzedowskii* W.R.Anderson; COL, JAL, MICH, OAX (endemic). |
| *Malpighia* *sessilifolia* W.R.Anderson; OAX, VER (endemic). |
| *Malpighia* *spathulifolia* F.K.Mey.; CAM, QROO, YUC. |
| *Malpighia* *wendtii* W.R.Anderson; CHIS, VER. |
| *Malpighia* *wilburiorum* W.R.Anderson; COL, JAL (endemic). |
| *Malpighia* *yucatanaea* F.K.Mey.; CAM, YUC (endemic). |
|  |
| **Malvaceae** |
| ***Abutilon*** |
| *Abutilon* *bastardioides* Baker f. ex Rose; COL, JAL (endemic). |
| *Abutilon* *grandidentatum* Fryxell; AGS, OAX, SIN, ZAC (endemic). |
| *Abutilon* *haenkeanum* C.Presl; CHIS, COL, GRO, JAL, MEX, MICH, MOR, NAY, OAX, SIN, ZAC (endemic). |
| *Abutilon* *tehuantepecense* Fryxell; OAX (endemic). |
| ***Apeiba*** |
| *Apeiba tibourbou* Aubl.; CHIS, GRO, HGO, JAL, NAY, OAX, PUE, VER; Red List: LC; banked. |
| ***Bakeridesia*** |
| *Bakeridesia* *amoena* Fryxell; OAX (endemic). |
| *Bakeridesia* *bakeriana* (Rose) D.M.Bates; COL, GRO, JAL, OAX (endemic); banked. |
| *Bakeridesia* *gloriosa* D.M.Bates; CHIS, OAX, VER (endemic). |
| *Bakeridesia* *guerrerensis* Donnell; GRO (endemic). |
| *Bakeridesia* *huastecana* Donnell; SLP, TAMS, VER (endemic). |
| *Bakeridesia* *integerrima* (Hook.) D.M.Bates; CHIS, GRO, HGO, OAX, QRO, SLP, SON, TAMS, VER, YUC. |
| *Bakeridesia* *jaliscana* Donnell; COL, JAL (endemic). |
| *Bakeridesia* *nelsonii* (Rose) D.M.Bates; CHIS. |
| *Bakeridesia* *notolophium* (A.Gray) Hochr.; CHIS, NLE, PUE, QRO, SLP, VER, ZAC (endemic); useful (SOCIAL USES). |
| *Bakeridesia* *parvifolia* Donnell; COL, JAL (endemic). |
| *Bakeridesia* *pittieri* (Donn.Sm.) D.M.Bates; CHIS, OAX. |
| *Bakeridesia* *zapoteca* Donnell; OAX (endemic). |
| ***Bernoullia*** |
| *Bernoullia flammea* Oliv.; CAM, CHIS, COL, GRO, JAL, MICH, NAY, OAX, QROO, TAB, VER; Red List: LC; useful (FOOD, MATERIALS). |
| *Bernoullia jaliscana* Miranda & McVaugh; COL, JAL (endemic). |
| ***Callianthe*** |
| *Callianthe jaliscana* (Standl.) Donnell; JAL (endemic). |
| *Callianthe purpusii* (Standl.) Donnell. |
| ***Carpodiptera*** |
| *Carpodiptera cubensis* Griseb. |
| ***Ceiba*** |
| *Ceiba* *acuminata* (S.Watson) Rose; AGS, BCS, CHIS, CHIH, COL, DGO, GTO, GRO, JAL, MEX, MICH, OAX, QRO, SIN, SON, TAMS, VER, ZAC (endemic); banked. |
| *Ceiba* *aesculifolia* (Kunth) Britten & Baker f.; AGS, CAM, CHIS, CHIH, COL, CDMX, DGO, GTO, GRO, HGO, JAL, MEX, MICH, MOR, NAY, OAX, PUE, QRO, QROO, SIN, SON, TAB, VER, YUC, ZAC; banked; useful (FOOD, MEDICINES, MATERIALS). |
| *Ceiba* *pentandra* (L.) Gaertn.; AGS, BCS, CAM, CHIS, CHIH, COL, GRO, HGO, JAL, MEX, MICH, MOR, NAY, OAX, PUE, QRO, QROO, SLP, SIN, SON, TAB, TAMS, VER, YUC, ZAC; Red List: LC; banked; useful (MEDICINES, MATERIALS, SOCIAL USES). |
| *Ceiba* *schottii* Britten & Baker f.; CHIS, CAM, QROO, YUC (endemic). |
| ***Chiranthodendron*** |
| *Chiranthodendron pentadactylon* Larreat.; CHIS, GRO, HGO, MEX, MICH, MOR, OAX, PUE, VER; Red List: LC; useful (MEDICINES). |
| ***Dendrosida*** |
| *Dendrosida* *batesii* J.E.Fryxell; CHIS (endemic). |
| *Dendrosida* *breedlovei* Fryxell; CHIS, OAX (endemic); NOM-59: A. |
| *Dendrosida* *parviflora* Fryxell; OAX (endemic). |
| *Dendrosida* *sharpiana* (Miranda) J.E.Fryxell; CHIS, GRO, MICH, OAX (endemic). |
| ***Gossypium*** |
| *Gossypium* *aridum* (Rose & Standl.) Skovst.; COL, DGO, GRO, JAL, MICH, NAY, OAX, PUE, SIN, VER, ZAC (endemic); Red List: VU. |
| *Gossypium* *gossypioides* (Ulbr.) Standl.; OAX, PUE (endemic); Red List: VU; banked; useful. |
| *Gossypium* *laxum* L.Ll.Phillips; GRO, MICH (endemic); Red List: EN. |
| *Gossypium* *lobatum* Gentry; GRO, JAL, MICH (endemic); Red List: EN. |
| *Gossypium* *schwendimanii* Fryxell & S.D.Koch; GRO, MICH (endemic); Red List: EN. |
| *Gossypium* *trilobum* (Moc. & Sessé ex DC.) Skovst.; HGO, JAL, MEX, MICH, MOR, QRO, SIN (endemic); Red List: EN. |
| ***Guazuma*** |
| *Guazuma ulmifolia* Lam.; CAM, CHIS, CHIH, COL, DGO, GTO, GRO, HGO, JAL, MEX, MICH, MOR, NAY, OAX, PUE, QRO, QROO, SLP, SIN, SON, TAB, TAMS, VER, YUC, ZAC; banked; useful (FOOD, ANIMAL FOOD, MEDICINES, MATERIALS, FUELS, ENVIRONMENTAL USES, SOCIAL USES). |
| ***Hampea*** |
| *Hampea* *breedlovei* Fryxell; CHIS (endemic); Red List: VU. |
| *Hampea* *integerrima* Schltdl.; OAX, TAB, VER. |
| *Hampea* *longipes* Miranda; CHIS, OAX, VER. |
| *Hampea mexicana* Fryxell; CHIS, COL, JAL, OAX (endemic). |
| *Hampea* *montebellensis* Fryxell; CHIS (endemic); Red List: EN; NOM-59: A. |
| *Hampea* *nutricia* Fryxell; CHIS, HGO, PUE, TAB, VER (endemic); banked; useful (FOOD, MATERIALS). |
| *Hampea* *rovirosae* Standl.; CAM, CHIS, TAB, VER. |
| *Hampea* *stipitata* S.Watson; CAM, CHIS, JAL, OAX, VER; Red List: LC. |
| *Hampea* *tomentosa* (C.Presl) Standl.; CHIS, COL, GRO, JAL, MICH, OAX, QROO, VER, YUC. |
| *Hampea* *trilobata* Standl.; CAM, CHIS, QROO, TAB, VER, YUC; useful (ANIMAL FOOD, MATERIALS). |
| ***Helicteres*** |
| *Helicteres baruensis* Jacq.; CAM, CHIS, COL, GRO, JAL, MICH, NAY, OAX, PUE, QROO, SLP, SIN, SON, TAB, TAMS, VER, YUC; Red List: LC; banked; useful (MATERIALS). |
| ***Heliocarpus*** |
| *Heliocarpus* *americanus* L.; AGS, CAM, CHIS, COL, GTO, GRO, HGO, JAL, MEX, MICH, MOR, OAX, PUE, QRO, QROO, SLP, TAB, TAMS, VER, YUC, ZAC; Red List: LC; banked; useful (ANIMAL FOOD, MEDICINES, MATERIALS). |
| *Heliocarpus* *appendiculatus* Turcz.; CHIS, COL, DGO, GRO, HGO, JAL, MICH, OAX, PUE, QRO, SLP, SIN, TAB, VER; Red List: LC; banked; useful (MATERIALS). |
| *Heliocarpus* *attenuatus* S.Watson; CHIH, SIN, SON (endemic). |
| *Heliocarpus* *donnellsmithii* Rose; CAM, CHIS, COL, GRO, HGO, JAL, MEX, MICH, MOR, NAY, OAX, PUE, QRO, QROO, SLP, SIN, TAB, TAMS, VER, YUC, ZAC. |
| *Heliocarpus* *occidentalis* Rose; CHIS, CHIH, COL, DGO, GRO, JAL, MEX, MICH, MOR, NAY, OAX, SIN, SON, ZAC; banked; useful (ANIMAL FOOD, MATERIALS, ENVIRONMENTAL USES). |
| *Heliocarpus* *pallidus* Rose; CHIS, COL, GRO, JAL, MEX, MICH, MOR, NAY, OAX, VER (endemic); banked. |
| *Heliocarpus* *palmeri* S.Watson; CHIH, COL, JAL, SIN, SON, ZAC (endemic). |
| *Heliocarpus* *terebinthinaceus* (DC.) Hochr.; banked; useful (ANIMAL FOOD). |
| *Heliocarpus* *velutinus* Rose; GRO, JAL, MEX, MICH, MOR, OAX, PUE (endemic). |
| ***Hibiscus*** |
| *Hibiscus tiliaceus* L.; CAM, CHIS, COL, GRO, JAL, MICH, NAY, OAX, PUE, QROO, SLP, SIN, TAB, TAMS, VER; Red List: LC. |
| ***Luehea*** |
| *Luehea candida* (Moc. & Sessé ex DC.) Mart.; CAM, CHIS, COL, DGO, GRO, JAL, MICH, NAY, OAX, QROO, SIN, TAB, VER, YUC; banked. |
| *Luehea seemannii* Triana & Planch.; CHIS, OAX; useful (ANIMAL FOOD, MATERIALS, FUELS, ENVIRONMENTAL USES). |
| *Luehea speciosa* Willd.; CAM, CHIS, COL, NAY, OAX, QROO, TAB, VER, YUC; Red List: LC; useful (MATERIALS). |
| ***Malvaviscus*** |
| *Malvaviscus lanceolatus* Rose; CHIS, COL, GRO, JAL, MOR, NAY, OAX, PUE, VER. |
| ***Melochia*** |
| *Melochia oaxacana* Dorr & L.C.Barnett; OAX (endemic). |
| *Melochia tomentosa* L.; BCN, BCS, CAM, CHIS, CHIH, COL, DGO, GRO, HGO, JAL, MEX, MICH, MOR, NAY, NLE, OAX, PUE, QROO, SLP, SIN, SON, TAB, TAMS, VER, YUC, ZAC; banked; useful (MEDICINES). |
| ***Mortoniodendron*** |
| *Mortoniodendron* *guatemalense* Standl. & Steyerm.; CHIS, OAX, TAB, VER; NOM-59: P. |
| *Mortoniodendron* *ocotense* Ishiki & T.Wendt; CHIS (endemic). |
| *Mortoniodendron* *palaciosii* Miranda; CHIS, OAX, VER (endemic). |
| *Mortoniodendron* *pentagonum* (Donn.Sm.) Miranda. |
| *Mortoniodendron* *ruizii* Miranda; CHIS, TAB. |
| *Mortoniodendron* *sulcatum* Lundell; CHIS, VER. |
| *Mortoniodendron* *uxpanapense* Dorr & T.Wendt; OAX, VER (endemic). |
| *Mortoniodendron* *vestitum* Lundell; CHIS, TAB. |
| ***Ochroma*** |
| *Ochroma pyramidale* (Cav. ex Lam.) Urb.; CHIS, OAX, TAB, VER; useful (MATERIALS). |
| ***Pachira*** |
| *Pachira aquatica* Aubl.; CAM, CHIS, GRO, JAL, MICH, NAY, OAX, PUE, QROO, SIN, TAB, VER, YUC; Red List: LC; banked; useful (FOOD, ENVIRONMENTAL USES). |
| ***Phymosia*** |
| *Phymosia rosea* (DC.) Kearney; CHIS, CHIH, COL, GRO, JAL, MEX, MICH, MOR, OAX, PUE, VER; NOM-59: Pr; useful (ENVIRONMENTAL USES). |
| *Phymosia umbellata* (Cav.) Kearney; CHIS, CDMX, GTO, HGO, MEX, PUE, QRO, SLP, TAMS, VER; banked; useful (ENVIRONMENTAL USES). |
| ***Physodium*** |
| *Physodium adenodes* (Goldberg) Fryxell; COL, DGO, GRO, JAL, MEX, MICH, NAY, OAX, SIN, ZAC (endemic). |
| ***Pseudobombax*** |
| *Pseudobombax ellipticum* (Kunth) Dugand; CAM, CHIS, COL, DGO, GTO, GRO, HGO, JAL, MEX, MICH, MOR, NAY, NLE, OAX, PUE, QRO, QROO, SLP, SIN, TAB, TAMS, VER, YUC, ZAC; banked; useful (MEDICINES, MATERIALS, ENVIRONMENTAL USES). |
| *Pseudobombax palmeri* (S.Watson) Dugand; BCS, CHIH, COL, JAL, NAY, SLP, SIN, SON, VER, ZAC (endemic); useful (MEDICINES). |
| ***Quararibea*** |
| *Quararibea* *funebris* (La Llave) Vischer; CAM, CHIS, HGO, OAX, PUE, QROO, TAB, VER, YUC; useful (MATERIALS). |
| *Quararibea* *lopezperaltae* Gallardo-Hern. & Lorea-Hern.; OAX, VER (endemic). |
| *Quararibea* *mayanum* Lorea-Hern. & Gallardo-Hern.; OAX (endemic). |
| *Quararibea* *yunckeri* Standl.; CHIS, OAX, TAB, VER; Red List: CR. |
| ***Robinsonella*** |
| *Robinsonella* *brevituba* Fryxell; CHIS, OAX, VER (endemic); Red List: VU. |
| *Robinsonella* *chiangii* Fryxell; OAX, PUE, VER (endemic); useful (ENVIRONMENTAL USES). |
| *Robinsonella* *cordata* Rose & Baker f.; CHIS, COL, DGO, GRO, JAL, MICH, MOR, OAX, PUE. |
| *Robinsonella* *densiflora* Fryxell; VER. |
| *Robinsonella* *discolor* Rose & Baker f.; GTO, HGO, NAY, OAX, QRO, SLP, TAMS, VER (endemic). |
| *Robinsonella* *glabrifolia* Fryxell; CHIS. |
| *Robinsonella* *hintonii* Fryxell; GRO, JAL, MEX, MICH, NAY (endemic). |
| *Robinsonella* *lindeniana* (Turcz.) Rose & Baker f.; CHIS, OAX, TAB, VER; useful (ENVIRONMENTAL USES). |
| *Robinsonella* *macvaughii* Fryxell; COL, JAL, MICH (endemic). |
| *Robinsonella* *mirandae* Gómez Pompa; CHIS, OAX, TAB, VER (endemic); Red List: VU; banked; useful (MATERIALS). |
| *Robinsonella* *pilosa* Rose; SLP, TAMS, VER. |
| *Robinsonella* *pilosissima* Fryxell; CHIS (endemic). |
| *Robinsonella* *samaricarpa* Fryxell; CHIS, OAX, VER (endemic); Red List: VU. |
| *Robinsonella* *speciosa* Fryxell; CHIS, COL, JAL, OAX; useful (ANIMAL FOOD). |
| ***Sterculia*** |
| *Sterculia apetala* (Jacq.) H.Karst.; CHIS, OAX, TAB, VER; Red List: LC; banked; useful (MATERIALS). |
| *Sterculia xolocotzii* T.Wendt & E.L.Taylor; OAX, VER (endemic). |
| ***Theobroma*** |
| *Theobroma angustifolium* DC.; CHIS, TAB. |
| *Theobroma cacao* L.; useful (FOOD, MEDICINES, POISONS, MATERIALS, SOCIAL USES). |
| ***Thespesia*** |
| *Thespesia populnea* (L.) Sol. ex Corrêa; CHIS, CAM, QROO, SLP, TAMS, VER, YUC; Red List: LC. |
| ***Tilia*** |
| *Tilia americana* L.; CHIS, CHIH, COAH, COL, DGO, GRO, HGO, JAL, MEX, MICH, MOR, NAY, NLE, OAX, PUE, QRO, SLP, SIN, SON, TAMS, VER; Red List: LC; NOM-59: P; useful (MEDICINES, MATERIALS, ENVIRONMENTAL USES). |
| ***Trichospermum*** |
| *Trichospermum galeottii* (Turcz.) Kosterm.; CHIS, COAH, COL, GRO, JAL, MEX, MICH, NAY, OAX, PUE, SLP, SIN, VER (endemic); Red List: LC; banked; useful (ENVIRONMENTAL USES). |
| *Trichospermum mexicanum* (DC.) Baill.; CHIS, OAX, VER; banked; useful (MATERIALS). |
| ***Triumfetta*** |
| *Triumfetta* *gonophora* W.W.Thomas & McVaugh; COL, JAL (endemic). |
| *Triumfetta* *guerrerensis* Gual, S.Peralta & Diego; GRO, OAX (endemic). |
| *Triumfetta* *indurata* W.W.Thomas & McVaugh; JAL (endemic). |
| *Triumfetta* *mexiae* C.V.Morton & Lay; GRO, MEX, MICH (endemic). |
| *Triumfetta* *paniculata* Hook. & Arn.; CHIS, COL, DGO, GRO, JAL, MICH, NAY, OAX, SIN (endemic). |
| *Triumfetta* *acahuizotlanensis* Gonz.-Martínez, J. Jiménez Ram. & Rios-Carr.; (endemic). |
|  |
| **Melastomataceae** |
| ***Bellucia*** |
| *Bellucia grossularioides* (L.) Triana; CHIS, OAX, TAB, VER; Red List: LC; useful (FOOD). |
| ***Conostegia*** |
| *Conostegia xalapensis* D.Don; CHIS, COL, DGO, GRO, HGO, JAL, MEX, MICH, MOR, NAY, OAX, PUE, QRO, SLP, SIN, TAB, TAMS, VER, YUC; Red List: LC; banked; useful (MEDICINES). |
| ***Miconia*** |
| *Miconia* *argentea* DC.; CAM, CHIS, OAX, PUE, QROO, TAB, VER, YUC; Red List: LC. |
| *Miconia* *glaberrima* Naudin; CHIS, COL, GRO, HGO, JAL, MEX, MICH, MOR, OAX, PUE, SIN, VER; Red List: LC; banked. |
| *Miconia mexicana* Naudin; CHIS, GRO, HGO, JAL, OAX, PUE, VER. |
| *Miconia* *rzedowskii* de Santiago; GRO, OAX (endemic). |
|  |
| **Meliaceae** |
| ***Cedrela*** |
| *Cedrela* *angustifolia* DC.; MICH, VER; useful (ENVIRONMENTAL USES). |
| *Cedrela* *discolor* S.F.Blake; DGO, GRO (endemic). |
| *Cedrela* *oaxacensis* C.DC. & Rose; CHIS, GRO, MEX, MOR, OAX, PUE (endemic); useful (MEDICINES, MATERIALS). |
| *Cedrela* *odorata* L.; AGS, CAM, CHIS, CHIH, COL, DGO, GTO, GRO, HGO, JAL, MEX, MICH, MOR, NAY, NLE, OAX, PUE, QRO, QROO, SLP, SIN, SON, TAB, TAMS, VER, YUC, ZAC; Red List: VU; NOM-59: Pr; Cites: III; banked; useful (FOOD, MEDICINES, MATERIALS, ENVIRONMENTAL USES). |
| *Cedrela* *salvadorensis* Standl.; CHIS, COL, DGO, GRO, JAL, MEX, MICH, MOR, NAY, OAX, PUE, SIN; banked. |
| *Cedrela* *tonduzii* C.DC.; CHIS, VER; Red List: LC; useful (ENVIROMENTAL USES, MEDICINES, MATERIALS). |
| ***Guarea*** |
| *Guarea glabra* Vahl; CAM, CHIS, COL, CDMX, DGO, GRO, HGO, JAL, MEX, MICH, MOR, NAY, OAX, PUE, QROO, SIN, TAB, VER; Red List: LC; banked. |
| *Guarea mexicana* Coronado; CHIS. |
| ***Swietenia*** |
| *Swietenia humilis* Zucc.; CAM, CHIS, COL, DGO, GRO, JAL, MEX, MICH, MOR, NAY, OAX, PUE, QROO, SLP, SIN, VER; Red List: VU; Cites: II; banked; useful (MEDICINES). |
| *Swietenia macrophylla* King; CAM, CHIS, COL, GTO, GRO, HGO, JAL, MEX, MICH, MOR, NAY, OAX, PUE, QRO, QROO, SIN, TAB, VER, YUC; Red List: VU; Cites: II/NC; banked; useful (MEDICINES). |
| ***Trichilia*** |
| *Trichilia* *americana* (Sessé & Moc.) T.D.Penn.; AGS, CAM, CHIS, CHIH, COL, DGO, GRO, JAL, MEX, MICH, MOR, NAY, OAX, PUE, QROO, SLP, SIN, SON, TAB, VER, YUC, ZAC; banked. |
| *Trichilia* *breviflora* S.F.Blake & Standl.; CHIS, MICH, OAX, VER; Red List: EN. |
| *Trichilia* *chirriactensis* (Standl. & Steyerm.) T.D.Penn.; CHIS; Red List: VU. |
| *Trichilia* *glabra* L.; CAM, CHIS, OAX, QROO, VER, YUC; useful (MEDICINES). |
| *Trichilia* *hirta* L.; CAM, CHIS, CHIH, COL, DGO, GRO, HGO, JAL, MEX, MICH, MOR, NAY, OAX, PUE, QRO, QROO, SLP, SIN, SON, TAB, TAMS, VER, YUC, ZAC; banked; useful (ENVIRONMENTAL USES). |
| *Trichilia* *martiana* C.DC.; CAM, CHIS, GRO, JAL, MICH, NAY, OAX, TAB, VER. |
| *Trichilia* *minutiflora* Standl.; CAM, CHIS, JAL, NAY, OAX, PUE, QROO, TAB, VER, YUC. |
| *Trichilia* *moschata* Sw.; CAM, CHIS, COL, JAL, OAX, QROO, TAB, VER; Red List: LC. |
| *Trichilia* *oligantha* C.DC.; VER (endemic). |
| *Trichilia* *pallida* Sw.; CAM, CHIS, OAX, QROO, TAB, VER; useful (FOOD, ANIMAL FOOD, MATERIALS). |
| *Trichilia* *pleeana* (A.Juss.) C.DC.; CHIS, VER (endemic); useful (ENVIRONMENTAL USES). |
| *Trichilia* *trifolia* L.; CAM, CHIS, COL, GRO, JAL, MEX, MICH, NAY, OAX, PUE, QROO, SLP, SIN, TAMS, VER, YUC. |
|  |
| **Menispermaceae** |
| ***Hyperbaena*** |
| *Hyperbaena* *ilicifolia* Standl.; COL, GRO, JAL, MICH (endemic). |
| *Hyperbaena* *jalcomulcensis* E.Pérez & Cast.-Campos; VER (endemic); Red List: VU. |
| *Hyperbaena mexicana* Miers; CAM, CHIS, GRO, MICH, OAX, QROO, TAB, VER, YUC; Red List: LC. |
| *Hyperbaena* *standleyi* Mathias & W.T.Theob.; CHIS, OAX. |
| *Hyperbaena* *winzerlingii* Standl.; CAM, QROO, YUC. |
|  |
| **Monimiaceae** |
| ***Mollinedia*** |
| *Mollinedia* *jerzyi* Lorence; OAX (endemic). |
| *Mollinedia* *oaxacana* Lorence; OAX (endemic). |
| *Mollinedia* *pallida* Lundell; CHIS. |
| *Mollinedia* *torresiorum* Lorence; OAX (endemic). |
| *Mollinedia* *viridiflora* Tul.; CHIS, GRO, OAX, PUE, TAB, VER. |
|  |
| **Moraceae** |
| ***Brosimum*** |
| *Brosimum alicastrum* Sw.; CAM, CHIS, COL, DGO, GRO, HGO, JAL, MEX, MICH, MOR, NAY, OAX, PUE, QRO, QROO, SLP, SIN, SON, TAB, TAMS, VER, YUC, ZAC; useful (FOOD, ANIMAL FOOD, MEDICINES, MATERIALS). |
| *Brosimum costaricanum* Liebm.; CHIS, TAB, VER. |
| *Brosimum guianense* (Aubl.) Huber ex Ducke; CHIS, OAX, TAB, VER; Red List: LC. |
| ***Castilla*** |
| *Castilla elastica* Cerv.; CAM, CHIS, COL, GRO, HGO, JAL, MEX, MICH, NAY, OAX, PUE, QRO, QROO, SLP, SIN, TAB, TAMS, VER, YUC; useful (MEDICINES, MATERIALS, FUELS, ENVIRONMENTAL USES, SOCIAL USES). |
| ***Clarisia*** |
| *Clarisia biflora* Ruiz & Pav.; CHIS, OAX, VER; Red List: LC; useful (ENVIRONMENTAL USES, ANIMAL FOOD, MATERIALS, FUELS, MEDICINES). |
| *Clarisia racemosa* Ruiz & Pav.; OAX; Red List: LC. |
| ***Ficus*** |
| *Ficus* *americana* Aubl.; CAM, CHIS, HGO, PUE, QRO, QROO, SLP, TAB, VER; Red List: LC. |
| *Ficus* *apollinaris* Dugand; CAM, CHIS, GRO, OAX, PUE, VER. |
| *Ficus* *aurea* Nutt.; CAM, CHIS, COL, GRO, HGO, JAL, MEX, MICH, MOR, NAY, OAX, PUE, QRO, QROO, SLP, TAB, TAMS, VER, YUC, ZAC; banked. |
| *Ficus* *cahuitensis* C.C.Berg. |
| *Ficus* *citrifolia* Mill.; CAM, CHIS, COL, DGO, GRO, JAL, MEX, MICH, NAY, OAX, QROO, TAB, VER, YUC; Red List: LC. |
| *Ficus* *colubrinae* Standl.; CHIS, OAX, TAB, VER. |
| *Ficus* *costaricana* (Liebm.) Miq.; Red List: LC. |
| *Ficus* *cotinifolia* Kunth; CAM, CHIS, CHIH, COL, DGO, GTO, GRO, HGO, JAL, MEX, MICH, MOR, NAY, OAX, PUE, QRO, QROO, SLP, SIN, SON, TAB, TAMS, VER, YUC, ZAC; Red List: LC; banked; useful (FOOD, ANIMAL FOOD, MEDICINES, POISONS, MATERIALS, ENVIRONMENTAL USES). |
| *Ficus* *crassinervia* Desf. ex Willd.; CAM, CHIS, OAX, QROO, TAB, VER, YUC; Red List: LC. |
| *Ficus* *crocata* (Miq.) Mart. ex Miq.; CAM, CHIS, COL, DGO, GRO, JAL, MEX, MICH, MOR, NAY, OAX, PUE, QRO, QROO, SLP, SIN, SON, TAB, TAMS, VER, YUC, ZAC; Red List: LC; banked. |
| *Ficus* *insipida* Willd.; CAM, CHIS, CHIH, COL, DGO, GRO, HGO, JAL, MEX, MICH, MOR, NAY, OAX, PUE, QRO, SLP, SIN, SON, TAB, TAMS, VER, ZAC; Red List: LC; banked; useful (MEDICINES). |
| *Ficus* *lapathifolia* (Liebm.) Miq.; CHIS, GRO, JAL, MEX, NAY, OAX, PUE, TAB, VER (endemic); Red List: VU. |
| *Ficus* *maxima* Mill.; CAM, CHIS, CHIH, COL, DGO, GRO, HGO, JAL, MEX, MICH, MOR, NAY, OAX, PUE, QRO, QROO, SLP, SIN, SON, TAB, VER, YUC; Red List: LC; useful (ANIMAL FOOD, MATERIALS). |
| *Ficus* *membranacea* C.Wright; COL, DGO, GRO, HGO, JAL, MEX, MICH, MOR, NAY, OAX, QRO, SLP, SIN, ZAC; useful (ANIMAL FOOD). |
| *Ficus* *obtusifolia* Kunth; CAM, CHIS, COL, GRO, HGO, JAL, MEX, MICH, MOR, NAY, OAX, PUE, QRO, QROO, SLP, SIN, TAB, TAMS, VER, YUC; Red List: LC; banked; useful (MEDICINES). |
| *Ficus* *paraensis* (Miq.) Miq.; CHIS, OAX, TAB, VER; Red List: LC; useful (ANIMAL FOOD). |
| *Ficus* *pertusa* L.f.; CAM, CHIS, CHIH, COL, DGO, GTO, GRO, HGO, JAL, MEX, MICH, MOR, NAY, OAX, PUE, QRO, QROO, SLP, SIN, SON, TAB, TAMS, VER, YUC, ZAC; Red List: LC; banked. |
| *Ficus* *petiolaris* Kunth; AGS, BCN, BCS, CHIS, CHIH, COL, DGO, GTO, GRO, JAL, MEX, MICH, MOR, NAY, OAX, PUE, SIN, SON, VER, ZAC (endemic); banked; useful (FOOD, MEDICINES, MATERIALS, ENVIRONMENTAL USES). |
| *Ficus* *popenoei* Standl.; CHIS; Red List: LC. |
| *Ficus* *pringlei* S.Watson; COL, GRO, JAL, MICH, NAY, OAX, ZAC (endemic). |
| *Ficus* *rzedowskiana* Carvajal & Cuevas-Figueroa; CHIS, GRO, HGO, OAX, PUE, QRO, SLP, VER (endemic); banked. |
| *Ficus* *trigonata* L.; CAM, CHIS, CHIH, COL, DGO, GRO, HGO, JAL, MEX, MICH, MOR, NAY, OAX, PUE, QRO, QROO, SLP, SIN, SON, TAB, TAMS, VER, YUC, ZAC; Red List: LC; useful (FOOD, ENVIRONMENTAL USES). |
| *Ficus* *turrialbana* W.C.Burger; CAM, CHIS, GRO, JAL, OAX, PUE, QROO, TAB, VER; Red List: LC. |
| *Ficus* *velutina* Humb. & Bonpl. ex Willd.; CAM, CHIS, COL, DGO, GRO, HGO, JAL, MEX, MICH, MOR, NAY, OAX, PUE, SLP, SIN, VER, ZAC; Red List: LC; banked. |
| *Ficus* *yoponensis* Desv.; CAM, CHIS, GRO, OAX, PUE, QROO, TAB, VER; Red List: LC; useful (ANIMAL FOOD, MEDICINES, MATERIALS, ENVIRONMENTAL USES). |
| ***Maclura*** |
| *Maclura tinctoria* (L.) D.Don ex Steud.; CAM, CHIS, CHIH, COL, DGO, GRO, HGO, JAL, MICH, NAY, OAX, PUE, QRO, QROO, SLP, SIN, SON, TAB, TAMS, VER, YUC; Red List: LC; banked; useful (MATERIALS). |
| ***Morus*** |
| *Morus celtidifolia* Kunth; CHIS, CHIH, COAH, COL, CDMX, DGO, GTO, HGO, JAL, MEX, MICH, MOR, NLE, OAX, PUE, QRO, SLP, SIN, SON, TAMS, VER, YUC, ZAC; banked; useful (FOOD, ANIMAL FOOD, MEDICINES, MATERIALS, ENVIRONMENTAL USES). |
| *Morus insignis* Bureau; CHIS; useful (ANIMAL FOOD, MATERIALS). |
| ***Poulsenia*** |
| *Poulsenia armata* (Miq.) Standl.; CHIS, OAX, TAB, VER; Red List: LC; banked; useful (FOOD, MATERIALS). |
| ***Pseudolmedia*** |
| *Pseudolmedia* *glabrata* (Liebm.) C.C.Berg; CAM, CHIS, GRO, HGO, MICH, OAX, PUE, QROO, TAB, VER, YUC; Red List: LC; useful (FOOD, ANIMAL FOOD, MATERIALS). |
| *Pseudolmedia spuria* (Sw.) Griseb.; CAM, CHIS, OAX, QROO. |
| ***Sorocea*** |
| *Sorocea trophoides* W.C.Burger; TAB, VER; Red List: LC. |
| ***Trophis*** |
| *Trophis* *cuspidata* Lundell; CHIS, HGO, OAX, PUE, VER. |
| *Trophis mexicana* (Liebm.) Bureau; CHIS, COL, GRO, HGO, JAL, MICH, NAY, OAX, PUE, TAB, VER; Red List: LC; useful (FOOD). |
| *Trophis* *noraminervae* Cuevas & Carvajal; COL, JAL (endemic). |
| *Trophis* *racemosa* (L.) Urb.; CAM, CHIS, CHIH, COL, DGO, GRO, HGO, JAL, MEX, MICH, MOR, NAY, OAX, PUE, QRO, QROO, SLP, SIN, SON, TAB, TAMS, VER, YUC; Red List: LC; useful (MATERIALS). |
| ***Urostigma*** |
| *Urostigma fuscescens* Liebm.; VER (endemic). |
|  |
| **Muntingiaceae** |
| ***Dicraspidia*** |
| *Dicraspidia donnell-smithii* Standl.; OAX. |
| ***Muntingia*** |
| *Muntingia calabura* L.; CAM, CHIS, COL, GRO, HGO, JAL, MEX, MICH, MOR, NAY, OAX, PUE, QRO, QROO, SLP, TAB, TAMS, VER, YUC; banked; useful (FOOD, ANIMAL FOOD, MEDICINES, MATERIALS, FUELS). |
|  |
| **Myricaceae** |
| ***Myrica*** |
| *Myrica cerifera* L.; CAM, CHIS, COL, DGO, GRO, HGO, JAL, MEX, MICH, NAY, OAX, PUE, QRO, QROO, SLP, TAB, TAMS, VER, YUC; banked. |
|  |
| **Myristicaceae** |
| ***Compsoneura*** |
| *Compsoneura sprucei* (A.DC.) Warb.; CHIS, TAB, VER; Red List: LC. |
| ***Virola*** |
| *Virola guatemalensis* (Hemsl.) Warb.; CHIS, OAX, TAB, VER; useful (FOOD, MATERIALS). |
| *Virola koschnyi* Warb.; CHIS; useful (ANIMAL FOOD, ENVIRONMENTAL USES, MEDICINES, POISONS, MATERIALS, SOCIAL USES). |
|  |
| **Myrtaceae** |
| ***Calycorectes*** |
| *Calycorectes mexicanus* O.Berg; HGO, OAX, QRO, SLP, VER (endemic). |
| ***Calyptranthes*** |
| *Calyptranthes* *chytraculia* (L.) Sw.; CHIS, OAX, QROO, VER, YUC; Red List: LC. |
| *Calyptranthes* *fluviatilis* Lundell; CHIS, OAX, QROO, TAB, VER. |
| *Calyptranthes* *hylobates* Standl. ex Amshoff; CHIS, OAX, PUE. |
| *Calyptranthes* *williamsii* Standl.; CAM, CHIS, COL, JAL, NAY, OAX, QROO, SIN, VER, YUC. |
| ***Chamguava*** |
| *Chamguava gentlei* (Lundell) Landrum; CHIS. |
| *Chamguava schippii* (Standl.) Landrum; CHIS, GRO. |
| ***Eugenia*** |
| *Eugenia* *acapulcensis* Steud.; CAM, CHIS, COL, DGO, GRO, HGO, JAL, MEX, MICH, MOR, NAY, OAX, PUE, QROO, SLP, SIN, SON, TAB, TAMS, VER, YUC; Red List: LC; banked. |
| *Eugenia* *aeruginea* DC.; CAM, CHIS, GRO, OAX, QROO, TAB, VER. |
| *Eugenia* *alnifolia* McVaugh; JAL, MEX, MICH (endemic). |
| *Eugenia* *amatenangensis* Lundell; CHIS. |
| *Eugenia* *axillaris* (Sw.) Willd.; CAM, CHIS, GRO, NAY, OAX, QRO, QROO, SLP, VER, YUC. |
| *Eugenia* *biflora* (L.) DC.; CHIS, QROO; useful (ENVIRONMENTAL USES). |
| *Eugenia* *breedlovei* Barrie; CHIS (endemic). |
| *Eugenia* *bumelioides* Standl.; CHIS, TAB. |
| *Eugenia* *cantuana* Lundell; MOR (endemic). |
| *Eugenia* *capuli* (Schltdl. & Cham.) Hook. & Arn.; CAM, CHIS, COL, GRO, HGO, JAL, MEX, MICH, MOR, NAY, OAX, PUE, QRO, QROO, SLP, SIN, TAB, TAMS, VER, YUC; Red List: LC; useful (FOOD, MATERIALS, ENVIRONMENTAL USES). |
| *Eugenia* *capulioides* Lundell; CHIS. |
| *Eugenia* *chiapensis* Lundell; CHIS. |
| *Eugenia* *choapamensis* Standl.; CHIS, OAX, PUE, TAB, VER. |
| *Eugenia* *coetzalensis* Durán-Esp. & Cast.-Campos; VER (endemic). |
| *Eugenia* *farameoides* A.Rich.; CHIS, OAX, QROO, TAB, VER. |
| *Eugenia* *flavoviridis* Lundell; CHIS. |
| *Eugenia* *foetida* Pers.; CAM, CHIS, OAX, QROO, YUC. |
| *Eugenia* *galalonensis* (C.Wright ex Griseb.) Krug & Urb.; CAM, CHIS, OAX, QROO, TAB, VER. |
| *Eugenia* *gaumeri* Standl.; CAM, QROO, YUC; Red List: LC. |
| *Eugenia* *guatemalensis* Donn.Sm.; CHIS, DGO, GRO, MEX, OAX, SIN, VER. |
| *Eugenia* *hypargyrea* Standl.; CHIS, VER; Red List: LC. |
| *Eugenia* *inirebensis* P.E.Sánchez; VER (endemic). |
| *Eugenia* *karwinskyana* O.Berg; CHIS, HGO, PUE, QRO, QROO, SLP, TAB, VER, YUC (endemic). |
| *Eugenia* *laevis* O.Berg; CAM, CHIS, QROO, YUC. |
| *Eugenia* *letreroana* Lundell; CHIS. |
| *Eugenia* *macrocarpa* Schltdl. & Cham.; CHIS, GRO, HGO, NAY, OAX, SLP, TAMS, VER (endemic); Red List: VU. |
| *Eugenia* *mozomboensis* P.E.Sánchez; VER (endemic); Red List: EN. |
| *Eugenia* *oerstediana* O.Berg; CAM, CHIS, COL, GRO, HGO, JAL, MEX, MOR, NAY, OAX, PUE, QRO, QROO, SLP, TAB, TAMS, VER. |
| *Eugenia* *ovandensis* Lundell; CHIS (endemic). |
| *Eugenia* *percivalii* Lundell; CHIS, VER. |
| *Eugenia* *pleurocarpa* Standl.; COL, GRO, JAL, NAY (endemic). |
| *Eugenia* *praeterita* McVaugh; CHIS, VER (endemic). |
| *Eugenia* *queretaroana* Sánchez-Cháv. & Zamudio; (endemic). |
| *Eugenia* *rhombea* (O.Berg) Krug & Urb.; CHIS, GRO, OAX, QROO, VER, YUC. |
| *Eugenia* *riograndis* Lundell; CAM, CHIS, TAB. |
| *Eugenia* *rubella* Lundell; CHIS. |
| *Eugenia* *salamensis* Donn.Sm.; COL, DGO, GRO, JAL, MICH, NAY, OAX, SIN; Red List: EN. |
| *Eugenia* *savannarum* Standl. & Steyerm.; CHIS. |
| *Eugenia* *siltepecana* Lundell; CHIS. |
| *Eugenia* *sotoesparzae* P.E.Sánchez; OAX, PUE, VER (endemic). |
| *Eugenia* *standleyi* McVaugh; CHIS, OAX (endemic). |
| *Eugenia* *symphoricarpos* McVaugh; TAMS, VER (endemic). |
| *Eugenia* *teapensis* McVaugh; CHIS, TAB, VER (endemic). |
| *Eugenia* *tikalana* Lundell; CAM, CHIS, QROO; Red List: LC. |
| *Eugenia* *toledinensis* Lundell; CHIS, QROO. |
| *Eugenia* *tonii* Lundell; CHIS (endemic). |
| *Eugenia* *trikii* Lundell; CAM, CHIS, QROO; Red List: LC. |
| *Eugenia* *trunciflora* (Schltdl. & Cham.) G.Don; CHIS, HGO, OAX, PUE, SLP, TAB, VER. |
| *Eugenia* *uliginosa* Lundell; CHIS, OAX (endemic). |
| *Eugenia* *uxpanapensis* P.E.Sánchez & L.M.Ortega; VER (endemic); Red List: EN. |
| *Eugenia* *venezuelensis* O.Berg; CHIS, GRO, JAL, MICH, OAX, SLP, TAB, TAMS, VER; Red List: LC. |
| *Eugenia* *verapazensis* Lundell; CHIS. |
| *Eugenia* *vesca* Lundell; CHIS. |
| *Eugenia* *winzerlingii* Standl.; CAM, CHIS, QROO, TAB, YUC; Red List: LC. |
| *Eugenia* *xalapensis* (Kunth) DC.; CHIS, GRO, HGO, OAX, QRO, SLP, TAMS, VER. |
| *Eugenia* *xilitlensis* McVaugh; QRO, SLP, VER (endemic). |
| ***Mosiera*** |
| *Mosiera contrerasii* (Lundell) Landrum; CAM, QROO. |
| *Mosiera ehrenbergii* (O.Berg) Landrum; HGO, QRO, SLP, TAMS, VER (endemic). |
| ***Myrcia*** |
| *Myrcia* *amazonica* DC.; Red List: LC. |
| *Myrcia* *calderonii* (Standl.) A.R.Lourenço & Sánchez-Cháv.; CHIS. |
| *Myrcia* *chiapensis* (Lundell) A.R.Lourenço & Sánchez-Cháv.; CHIS, VER. |
| *Myrcia* *contrerasii* (Lundell) A.R.Lourenço & Sánchez-Cháv.; CHIS. |
| *Myrcia* *karlingii* (Standl.) A.R.Lourenço & Sánchez-Cháv.; CAM, CHIS, QROO, TAB, VER. |
| *Myrcia* *karwinskyana* (O.Berg) A.R.Lourenço & Sánchez-Cháv.; CHIS, PUE, TAB, VER (endemic). |
| *Myrcia* *macrantha* (Standl. & Steyerm.) A.R.Lourenço & Sánchez-Cháv.; CHIS. |
| *Myrcia* *mayana* (Lundell) A.R.Lourenço & Sánchez-Cháv.; CHIS. |
| *Myrcia* *megistophylla* (Standl.) A.R.Lourenço & Sánchez-Cháv.; CHIS, OAX, VER. |
| *Myrcia* *millspaughii* (Urb.) A.R.Lourenço & Sánchez-Cháv.; CHIS, OAX, QROO, TAB, VER, YUC. |
| *Myrcia* *paxillata* (McVaugh) A.R.Lourenço & Sánchez-Cháv.; CHIS, VER. |
| *Myrcia* *perlaevigata* (Lundell) A.R.Lourenço & Sánchez-Cháv.; CHIS. |
| *Myrcia* *schlechtendaliana* (O.Berg) A.R.Lourenço & Sánchez-Cháv.; CAM, CHIS, COL, DGO, GRO, HGO, NAY, OAX, PUE, SIN, VER. |
| *Myrcia* *splendens* (Sw.) DC.; CHIS, MICH, OAX, TAB, VER. |
| *Myrcia* *tenuipes* (McVaugh) A.R.Lourenço & Sánchez-Cháv.; OAX, PUE, VER (endemic). |
| *Myrcia* *tonii* (Lundell) A.R.Lourenço & Sánchez-Cháv.; CHIS. |
| *Myrcia* *zuzygium* (L.) A.R.Lourenço & E.Lucas; MICH, NAY. |
| ***Myrcianthes*** |
| *Myrcia*nthes *fragrans* (Sw.) McVaugh; CAM, CHIS, COL, DGO, GRO, HGO, JAL, MEX, MICH, MOR, NAY, OAX, QRO, QROO, SLP, SIN, TAB, TAMS, VER, YUC; Red List: LC; useful (FOOD, MATERIALS). |
| ***Myrcia*ria** |
| *Myrcia*ria *floribunda* (H.West ex Willd.) O.Berg; CAM, CHIS, GRO, OAX, QROO, TAB, VER, YUC; Red List: LC. |
| *Myrcia*ria *ibarrae* Lundell; CAM, QROO. |
| ***Pimenta*** |
| *Pimenta dioica* (L.) Merr.; useful (FOOD, MEDICINES, POISONS, MATERIALS, FUELS). |
| ***Psidium*** |
| *Psidium* *friedrichsthalianum* (O.Berg) Nied.; CHIS, OAX, VER; Red List: LC. |
| *Psidium* *guajava* L.; BCS, CAM, CHIS, CHIH, COAH, COL, DGO, GTO, GRO, HGO, JAL, MEX, MICH, MOR, NAY, NLE, OAX, PUE, QRO, QROO, SLP, SIN, SON, TAB, TAMS, VER, YUC, ZAC; Red List: LC; banked; useful (FOOD, ANIMAL FOOD, MEDICINES, POISONS, MATERIALS, FUELS). |
| *Psidium* *guineense* Sw.; CAM, CHIS, COL, DGO, GRO, JAL, MICH, NAY, OAX, QRO, QROO, SLP, SIN, TAB, VER, YUC; Red List: LC; useful (MEDICINES). |
| *Psidium* *oligospermum* Mart. ex DC.; AGS, BCS, CAM, CHIS, CHIH, COL, DGO, GRO, HGO, JAL, MEX, MICH, MOR, NAY, OAX, PUE, QRO, QROO, SLP, SIN, SON, TAB, TAMS, TLAX, VER, YUC, ZAC; useful (FOOD, MATERIALS). |
| *Psidium* *salutare* (Kunth) O.Berg; CHIS, GRO, NAY, OAX, QROO, TAB, VER, YUC; Red List: LC; useful (FOOD). |
| ***Ugni*** |
| *Ugni myricoides* (Kunth) O.Berg; CHIS, HGO, OAX, PUE, VER; Red List: LC; banked. |
|  |
| **Nyctaginaceae** |
| ***Guapira*** |
| *Guapira petenensis* (Lundell) Lundell; CHIS, COL, GRO, JAL, MEX, MICH, MOR, OAX, PUE, QROO, VER. |
| ***Neea*** |
| *Neea psychotrioides* Donn.Sm.; CAM, CHIS, COL, GRO, HGO, JAL, MICH, OAX, PUE, QRO, QROO, SLP, SIN, TAB, TAMS, VER, YUC; useful (MATERIALS). |
| ***Pisonia*** |
| *Pisonia capitata* (S.Watson) Standl.; BCS, CHIH, COL, DGO, GRO, JAL, MICH, NAY, SIN, SON, VER (endemic); useful (MEDICINES). |
|  |
| **Nyssaceae** |
| ***Nyssa*** |
| *Nyssa sylvatica* Marshall; CHIS, HGO, NLE, PUE, TAMS, VER; Red List: LC. |
|  |
| **Ochnaceae** |
| ***Ouratea*** |
| *Ouratea* *acuminata* (DC.) Engl.; GRO, OAX. |
| *Ouratea* *curvata* (A.St.-Hil.) Engl. ex Dwyer; CHIS, VER. |
| *Ouratea* *gigantophylla* (Erhard) Engl. |
| *Ouratea* *jaliscensis* McVaugh; JAL (endemic). |
| *Ouratea* *jurgensenii* (Planch.) Engl.; CHIS, OAX (endemic). |
| *Ouratea* *lucens* (Kunth) Engl.; CAM, CHIS, GRO, NAY, OAX, QROO, TAB, VER, YUC. |
| *Ouratea mexicana* (Bonpl.) Engl.; CHIS, COL, GRO, JAL, MICH, NAY, OAX, SIN. |
|  |
| **Olacaceae** |
| ***Heisteria*** |
| *Heisteria acuminata* (Bonpl.) Engl.; CHIS. |
| *Heisteria macrophylla* Oerst.; CHIS. |
| *Heisteria media* S.F.Blake; CHIS, VER. |
| ***Ximenia*** |
| *Ximenia americana* L.; CAM, CHIS, COL, GRO, HGO, JAL, MICH, NAY, OAX, PUE, QROO, TAB, VER, YUC, ZAC; Red List: LC; banked; useful (MEDICINES). |
|  |
| **Oleaceae** |
| ***Chionanthus*** |
| *Chionanthus ligustrinus* (Sw.) Pers.; CHIS, VER; useful (ENVIRONMENTAL USES). |
| *Chionanthus oblanceolatus* (B.L.Rob.) P.S.Green; CHIS, MICH, OAX, TAB, VER; Red List: LC. |
| *Chionanthus panamensis* (Standl.) Stearn; CHIS, VER; Red List: LC. |
| ***Forestiera*** |
| *Forestiera* *cartaginensis* Donn.Sm.; CHIS. |
| *Forestiera* *durangensis* Standl.; DGO, GTO, HGO, OAX, PUE, QRO, SIN, TAMS, ZAC (endemic). |
| *Forestiera* *macrocarpa* Brandegee; BCS (endemic). |
| *Forestiera* *phillyreoides* (Benth.) Torr.; AGS, BCS, CHIH, COAH, COL, DGO, GTO, GRO, HGO, JAL, MEX, MICH, MOR, NLE, OAX, PUE, QRO, SLP, SON, TLAX, VER, ZAC; banked. |
| *Forestiera* *reticulata* Torr.; CHIS, COAH, COL, GTO, GRO, HGO, JAL, MEX, NLE, OAX, PUE, QRO, SLP, TAMS, VER. |
| *Forestiera* *rhamnifolia* Griseb.; CHIS, COL, JAL, NAY, OAX, QROO, SIN, SON, TAB, VER. |
| ***Fraxinus*** |
| *Fraxinus* *berlandieriana* A.DC.; Red List: LC; useful (MEDICINES). |
| *Fraxinus* *cuspidata* Torr.; CHIH, COAH, DGO, NLE, SLP, TAMS; Red List: LC. |
| *Fraxinus* *dipetala* Hook. & Arn.; BCN, BCS; Red List: LC; banked. |
| *Fraxinus* *dubia* (Willd. ex Schult. & Schult.f.) P.S.Green & M.Nee; CHIS, OAX, QRO, SLP, TAMS, VER (endemic); banked; useful (MATERIALS, ENVIRONMENTAL USES). |
| *Fraxinus* *gooddingii* Little; CHIH, SON; Red List: LC. |
| *Fraxinus* *greggii* A.Gray; CHIH, COAH, DGO, HGO, JAL, NLE, QRO, SLP, SON, TAMS, VER, ZAC; Red List: LC; banked. |
| *Fraxinus* *papillosa* Lingelsh.; AGS, CHIH, HGO, JAL, SON, ZAC. |
| *Fraxinus* *pringlei* Lingelsh.; CHIH, GRO, HGO, MICH, OAX, SON, VER (endemic); Red List: LC. |
| *Fraxinus* *purpusii* Brandegee; CHIS, GRO, JAL, MEX, MICH, OAX, PUE, SLP, TAMS; Red List: LC; useful (FUELS). |
| *Fraxinus* *rufescens* Lingelsh.; DGO, GTO, HGO, QRO, SLP, VER (endemic); Red List: LC. |
| *Fraxinus* *uhdei* (Wenz.) Lingelsh.; AGS, CHIS, COAH, COL, CDMX, DGO, GTO, GRO, HGO, JAL, MEX, MICH, MOR, NAY, NLE, OAX, PUE, QRO, SLP, SIN, TLAX, VER, ZAC; Red List: LC; banked; useful (ANIMAL FOOD, MEDICINES, MATERIALS, FUELS, ENVIRONMENTAL USES). |
| *Fraxinus* *velutina* Torr.; AGS, BCN, BCS, CHIS, CHIH, COAH, DGO, NLE, PUE, SLP, SON, TAMS, VER, ZAC; Red List: LC; banked. |
|  |
| **Onagraceae** |
| ***Fuchsia*** |
| *Fuchsia arborescens* Sims; CHIS, COL, DGO, GTO, GRO, HGO, JAL, MEX, MICH, MOR, OAX, PUE, SIN, VER; banked; useful (ANIMAL FOOD). |
| ***Hauya*** |
| *Hauya elegans* Moc. & Sessé ex DC.; CHIS, COL, GTO, GRO, HGO, JAL, MEX, MICH, MOR, OAX, PUE, QRO, SLP, VER; Red List: LC; banked. |
|  |
| **Opiliaceae** |
| ***Agonandra*** |
| *Agonandra obtusifolia* Standl.; CHIS, GTO, OAX, PUE, QRO, QROO, SLP, TAMS, VER, YUC (endemic); banked; useful (FUELS, ENVIRONMENTAL USES). |
| *Agonandra racemosa* (DC.) Standl.; CHIS, CHIH, COL, DGO, GTO, GRO, JAL, MEX, MICH, MOR, NAY, OAX, PUE, QRO, QROO, SLP, SIN, SON, TAMS, VER, YUC, ZAC; banked; useful (MATERIALS, FUELS). |
|  |
| **Papaveraceae** |
| ***Bocconia*** |
| *Bocconia* *glaucifolia* Hutch.; CHIS. |
| *Bocconia* *gracilis* Hutch.; CHIS. |
| *Bocconia* *hintoniorum* B.L.Turner; OAX (endemic). |
| *Bocconia* *integrifolia* Bonpl.; HGO, OAX, VER; banked. |
| *Bocconia* *vulcanica* Donn.Sm.; CHIS. |
|  |
| **Pentaphylacaceae** |
| ***Cleyera*** |
| *Cleyera* *cernua* (Tul.) Kobuski; OAX, VER (endemic); Red List: DD. |
| *Cleyera* *integrifolia* (Benth.) Choisy; CHIS, COL, DGO, GRO, HGO, JAL, MEX, MICH, MOR, NAY, OAX, SIN, VER, ZAC (endemic); Red List: LC. |
| *Cleyera* *serrulata* Choisy; HGO, VER (endemic). |
| *Cleyera* *theoides* (Sw.) Choisy; CHIS, HGO, OAX, PUE, QRO, SLP, VER; banked. |
| *Cleyera* *velutina* B.M.Barthol.; GRO, MICH, OAX (endemic). |
| ***Freziera*** |
| *Freziera candicans* Tul.; CHIS, GRO, OAX. |
| *Freziera grisebachii* Krug & Urb.; CHIS, OAX, VER. |
| *Freziera guatemalensis* (Donn.Sm.) Kobuski; CHIS, OAX, VER. |
| ***Symplococarpon*** |
| *Symplococarpon flavifolium* Lundell; CHIS (endemic). |
| *Symplococarpon purpusii* (Brandegee) Kobuski; CHIS, COL, GRO, JAL, MEX, MICH, NAY, OAX, VER. |
| ***Ternstroemia*** |
| *Ternstroemia* *dentisepala* B.M.Barthol.; COL, DGO, JAL, NAY, OAX, SIN (endemic). |
| *Ternstroemia* *huasteca* B.M.Barthol.; HGO, PUE, QRO, SLP, VER (endemic); banked. |
| *Ternstroemia* *lineata* DC.; AGS, CHIS, COL, DGO, GRO, HGO, JAL, MEX, MICH, MOR, NAY, OAX, SIN, TAMS, VER; Red List: LC; useful (MEDICINES, MATERIALS). |
| *Ternstroemia* *maltbya* Rose; JAL, NAY, SIN (endemic). |
| *Ternstroemia* *sylvatica* Schltdl. & Cham.; CDMX, GTO, GRO, HGO, JAL, MEX, MICH, MOR, OAX, PUE, QRO, SLP, TAMS, VER; useful (MEDICINES). |
| *Ternstroemia* *tepezapote* Schltdl. & Cham.; CAM, CHIS, GRO, HGO, MEX, MICH, OAX, PUE, QRO, QROO, SLP, TAB, VER; banked. |
|  |
| **Petenaeaceae** |
| ***Petenaea*** |
| *Petenaea cordata* Lundell; CHIS, TAB; Red List: EN. |
|  |
| **Phyllanthaceae** |
| ***Astrocasia*** |
| *Astrocasia neurocarpa* (Müll.Arg.) I.M.Johnst. ex Standl.; GRO, HGO, NAY, OAX, PUE, QRO, SLP, TAMS (endemic). |
| *Astrocasia peltata* Standl.; COL, GRO, JAL, NAY, SIN (endemic). |
| ***Hieronyma*** |
| *Hieronyma alchorneoides* Allemão; CHIS; Red List: LC; useful (ANIMAL FOOD, MEDICINES, ENVIRONMENTAL USES). |
| ***Margaritaria*** |
| *Margaritaria nobilis* L.f.; CAM, CHIS, COL, GRO, JAL, MEX, MICH, NAY, OAX, QRO, QROO, SLP, SIN, TAB, TAMS, VER, YUC; useful (FOOD, ANIMAL FOOD, MATERIALS). |
| ***Meineckia*** |
| *Meineckia bartlettii* (Standl.) G.L.Webster; CHIS, COL, JAL. |
| ***Phyllanthus*** |
| *Phyllanthus* *acuminatus* Vahl; BCS, CAM, CHIS, COL, GRO, HGO, JAL, MICH, NAY, OAX, QROO, SLP, SIN, SON, TAB, TAMS, VER, YUC. |
| *Phyllanthus* *adenodiscus* Müll.Arg.; CHIS, HGO, JAL, NAY, PUE, QRO, SLP, TAMS, VER, ZAC (endemic). |
| *Phyllanthus* *barbarae* M.C.Johnst.; QRO, SLP, TAMS (endemic). |
| *Phyllanthus* *botryanthus* Müll.Arg.; COL, JAL. |
| *Phyllanthus* *coalcomanensis* Croizat; COL, GRO, MICH, NAY, SIN (endemic). |
| *Phyllanthus* *elsiae* Urb.; CAM, CHIS, CHIH, COL, GRO, JAL, MICH, NAY, OAX, QROO, SIN, TAMS. |
| *Phyllanthus* *grandifolius* L.; CAM, CHIS, COL, CDMX, GRO, HGO, JAL, MEX, MICH, NAY, OAX, QRO, QROO, SLP, TAMS, VER, YUC, ZAC; useful (MEDICINES, MATERIALS). |
| *Phyllanthus* *gypsicola* McVaugh; COL, JAL (endemic). |
| *Phyllanthus* *mickelii* McVaugh; COL, JAL (endemic). |
| *Phyllanthus* *oaxacanus* Brandegee; OAX, PUE (endemic). |
| *Phyllanthus* *petaloideus* Paul G.Wilson; MEX (endemic). |
| *Phyllanthus* *purpusii* Brandegee; CHIS, OAX. |
| *Phyllanthus* *tuerckheimii* G.L.Webster; CHIS, OAX. |
| ***Savia*** |
| *Savia sessiliflora* (Sw.) Willd.; CAM, COL, HGO, JAL, NAY, QRO, QROO, SLP, TAMS, VER, YUC. |
|  |
| **Phyllonomaceae** |
| ***Phyllonoma*** |
| *Phyllonoma laticuspis* (Turcz.) Engl.; CHIS, DGO, GRO, HGO, JAL, MEX, MICH, MOR, OAX, PUE, QRO, SLP, SIN, VER; banked; useful (MEDICINES). |
|  |
| ***Picramnia*ceae** |
| ***Alvaradoa*** |
| *Alvaradoa amorphoides* Liebm.; CAM, CHIS, CHIH, COL, DGO, GRO, JAL, MEX, MICH, MOR, NAY, OAX, PUE, QROO, SIN, SON, VER, YUC, ZAC; banked; useful (ANIMAL FOOD, MEDICINES, MATERIALS, FUELS, ENVIRONMENTAL USES). |
| ***Picramnia*** |
| *Picramnia* *antidesma* Sw.; CAM, CHIS, COL, GRO, HGO, JAL, MEX, MICH, MOR, NAY, OAX, PUE, QRO, QROO, SLP, SIN, TAB, VER, YUC; useful (MEDICINES). |
| *Picramnia* *deflexa* W.W.Thomas; CHIS (endemic). |
| *Picramnia* *guerrerensis* W.W.Thomas; CHIS, COL, GRO, JAL, MEX, MICH, SIN (endemic). |
| *Picramnia* *hirsuta* W.W.Thomas; CHIS, OAX, VER. |
| *Picramnia* *polyantha* (Benth.) Planch.; CHIS, COAH, GRO, NLE, OAX, PUE, QRO, SLP, VER; useful (MEDICINES). |
| *Picramnia* *teapensis* Tul.; CHIS, OAX, PUE, QROO, TAB, VER, YUC. |
| *Picramnia* *thomasii* Gonz.-Martínez & J.Jiménez Ram.; GRO (endemic). |
| *Picramnia* *xalapensis* Planch.; HGO, OAX, PUE, QRO, SLP, VER (endemic); useful (MEDICINES). |
|  |
| **Picrodendraceae** |
| ***Piranhea*** |
| *Piranhea mexicana* (Standl.) Radcl.-Sm.; COL, JAL, NAY, SIN (endemic); banked; useful (MATERIALS). |
|  |
| **Pinaceae** |
| ***Abies*** |
| *Abies* *concolor* (Gordon & Glend.) Lindl. ex Hildebr.; BCN, CHIH, SON; Red List: LC; NOM-59: Pr; useful (MATERIALS). |
| *Abies* *durangensis* Martínez; CHIH, COAH, DGO, JAL, NLE, SIN, SON, TAMS, ZAC (endemic); Red List: LC; useful (MATERIALS). |
| *Abies* *hickelii* Flous & Gaussen; CHIS, GRO, OAX, VER; Red List: EN. |
| *Abies* *hidalgensis* Debreczy, I.Rácz & Guízar; HGO, VER (endemic); Red List: VU. |
| *Abies* *religiosa* (Kunth) Schltdl. & Cham.; AGS, CHIS, COL, CDMX, DGO, GTO, GRO, HGO, JAL, MEX, MICH, MOR, NLE, OAX, PUE, QRO, SLP, SIN, TAMS, TLAX, VER, ZAC; Red List: LC; banked; useful (ENVIRONMENTAL USES). |
| *Abies* *vejarii* Martínez; COAH, HGO, NLE, TAMS, VER (endemic); Red List: NT. |
| ***Picea*** |
| *Picea chihuahuana* Martínez; CHIH, COAH, DGO, JAL, NLE, SIN, SON, TAMS, ZAC (endemic); Red List: EN; useful (ENVIRONMENTAL USES). |
| *Picea engelmannii* Parry ex Engelm.; CHIH, COAH, NLE, SON, TAM; Red List: LC. |
| ***Pinus*** |
| *Pinus* *arizonica* Engelm.; CHIH, COAH, DGO, NAY, NLE, SLP, SIN, SON, TAMS, ZAC; Red List: LC. |
| *Pinus* *attenuata* Lemmon; BCN; Red List: LC. |
| *Pinus* *ayacahuite* Ehrenb. ex Schltdl.; AGS, CHIS, CHIH, COAH, CDMX, DGO, GTO, GRO, HGO, JAL, MEX, MICH, MOR, NLE, OAX, PUE, QRO, SLP, SIN, SON, TAMS, TLAX, VER, ZAC; Red List: LC; banked. |
| *Pinus* *caribaea* Morelet; CAM, QROO; Red List: LC; useful (MATERIALS, FUELS, MEDICINES, FOOD, ENVIRONMENTAL USES). |
| *Pinus* *cembroides* Zucc.; AGS, BCN, BCS, CHIH, COAH, CDMX, DGO, GTO, HGO, JAL, MEX, MICH, NAY, NLE, PUE, QRO, SLP, SIN, SON, TAMS, TLAX, VER, ZAC; Red List: LC; banked; useful (FOOD, MATERIALS, FUELS). |
| *Pinus* *contorta* Douglas ex Loudon; BCN, CAM, YUC; Red List: LC; NOM-59: Pr; banked; useful (MATERIALS). |
| *Pinus* *coulteri* D.Don; BCN; Red List: NT; NOM-59: P. |
| *Pinus* *culminicola* Andresen & Beaman; COAH, NLE, SLP (endemic); Red List: EN. |
| *Pinus* *devoniana* Lindl.; AGS, CHIS, COL, CDMX, DGO, GTO, GRO, HGO, JAL, MEX, MICH, MOR, NAY, NLE, OAX, PUE, QRO, SLP, SIN, TLAX, VER, ZAC; Red List: LC. |
| *Pinus* *douglasiana* Martínez; CHIS, CHIH, COL, DGO, GTO, GRO, JAL, MEX, MICH, MOR, NAY, OAX, QRO, SLP, SIN, SON, TLAX, ZAC (endemic); Red List: LC; banked. |
| *Pinus* *durangensis* Martínez; AGS, CHIH, COL, DGO, GTO, JAL, MEX, MICH, NAY, QRO, SLP, SIN, SON, ZAC (endemic); Red List: NT. |
| *Pinus* *edulis* Engelm.; CHIH; Red List: LC. |
| *Pinus* *engelmannii* Carrière; AGS, CHIH, COAH, DGO, JAL, NLE, SIN, SON, TAMS, ZAC; Red List: LC. |
| *Pinus* *flexilis* E.James; CHIH, COAH, DGO, NLE, SLP, TAMS; Red List: LC. |
| *Pinus* *georginae* Pérez de la Rosa; JAL (endemic). |
| *Pinus* *greggii* Engelm. ex Parl.; COAH, HGO, NLE, PUE, QRO, SLP, TAMS, VER (endemic); Red List: VU; banked. |
| *Pinus* *hartwegii* Lindl.; AGS, CHIS, CHIH, COAH, COL, CDMX, DGO, GTO, GRO, HGO, JAL, MEX, MICH, MOR, NAY, NLE, OAX, PUE, QRO, SLP, TAMS, TLAX, VER, ZAC; Red List: LC; banked; useful (MEDICINES, MATERIALS, FUELS). |
| *Pinus* *herrerae* Martínez; CHIH, COL, DGO, GRO, JAL, MEX, MICH, OAX, SIN, SON (endemic); Red List: LC; banked; useful (MATERIALS, FUELS). |
| *Pinus* *jaliscana* Pérez de la Rosa; COL, JAL, NAY (endemic); Red List: NT. |
| *Pinus* *jeffreyi* Balf.; BCN; Red List: LC; NOM-59: Pr; banked. |
| *Pinus* *lambertiana* Douglas; BCN; Red List: LC. |
| *Pinus* *lawsonii* Roezl ex Gordon; CDMX, GTO, GRO, JAL, MEX, MICH, MOR, NAY, OAX, PUE, QRO, VER (endemic); Red List: LC; useful (MATERIALS, FUELS). |
| *Pinus* *leiophylla* Schiede ex Schltdl. & Cham.; AGS, CHIS, CHIH, COL, CDMX, DGO, GTO, GRO, HGO, JAL, MEX, MICH, MOR, NAY, OAX, PUE, QRO, SLP, SIN, SON, TLAX, VER, ZAC; Red List: LC. |
| *Pinus* *lumholtzii* B.L.Rob. & Fernald; AGS, CHIH, COL, DGO, GTO, JAL, MICH, NAY, QRO, SIN, SON, ZAC (endemic); Red List: NT; banked. |
| *Pinus* *luzmariae* Pérez de la Rosa; DGO, JAL, NAY, SIN, ZAC (endemic); Red List: LC. |
| *Pinus* *maximartinezii* Rzed.; DGO, JAL, ZAC (endemic); Red List: EN. |
| *Pinus* *maximinoi* H.E.Moore; CHIS, CHIH, COL, DGO, GRO, HGO, JAL, MEX, MICH, MOR, NAY, OAX, PUE, SIN, SON, TLAX, VER; Red List: LC; banked; useful (ENVIRONMENTAL USES). |
| *Pinus* *monophylla* Torr. & Frém.; BCN; Red List: LC; NOM-59: Pr. |
| *Pinus* *montezumae* Lamb.; CHIS, CHIH, COAH, COL, CDMX, DGO, GTO, GRO, HGO, JAL, MEX, MICH, MOR, NAY, NLE, OAX, PUE, QRO, SLP, SIN, SON, TAB, TAMS, TLAX, VER, ZAC; Red List: LC; useful (MEDICINES, MATERIALS, FUELS, ENVIRONMENTAL USES). |
| *Pinus* *muricata* D.Don; BCN; Red List: VU; banked. |
| *Pinus* *nelsonii* Shaw; COAH, NLE, QRO, SLP, TAMS, ZAC (endemic); Red List: EN. |
| *Pinus* *oocarpa* Schiede; AGS, CHIS, CHIH, COL, CDMX, DGO, GTO, GRO, HGO, JAL, MEX, MICH, MOR, NAY, NLE, OAX, PUE, QRO, SLP, SIN, SON, TAMS, TLAX, VER, ZAC; Red List: LC; banked; useful (MEDICINES, MATERIALS, FUELS). |
| *Pinus* *patula* Schiede ex Schltdl. & Cham.; CHIS, COAH, COL, CDMX, GRO, HGO, JAL, MEX, MICH, MOR, NAY, NLE, OAX, PUE, QRO, SLP, SIN, TAMS, TLAX, VER; Red List: LC; banked; useful (ENVIRONMENTAL USES). |
| *Pinus* *pinceana* Gordon & Glend.; COAH, DGO, HGO, NLE, QRO, SLP, TAMS, VER, ZAC (endemic); Red List: LC; NOM-59: P. |
| *Pinus* *ponderosa* Douglas ex C.Lawson; CHIH, COAH, DGO, SON, ZAC; Red List: LC. |
| *Pinus* *praetermissa* Styles & McVaugh; COL, DGO, JAL, MICH, NAY, SIN, ZAC (endemic); Red List: NT. |
| *Pinus* *pringlei* Shaw; DGO, GRO, HGO, JAL, MEX, MICH, MOR, NAY, OAX, PUE, QRO, TLAX (endemic); Red List: LC; useful (MATERIALS, FUELS). |
| *Pinus* *pseudostrobus* Lindl.; CHIS, CHIH, COAH, COL, CDMX, DGO, GTO, GRO, HGO, JAL, MEX, MICH, MOR, NAY, NLE, OAX, PUE, QRO, SLP, SIN, SON, TAMS, TLAX, VER; Red List: LC; banked; useful (ENVIRONMENTAL USES). |
| *Pinus* *quadrifolia* Parl. ex Sudw.; BCN; Red List: LC; NOM-59: Pr; banked; useful. |
| *Pinus* *radiata* D.Don; BCN, BCS; Red List: EN; useful (ENVIRONMENTAL USES). |
| *Pinus* *rzedowskii* Madrigal & M.Caball.; MICH (endemic); Red List: VU. |
| *Pinus* *strobiformis* Engelm.; CHIH, COAH, DGO, GTO, JAL, NLE, SLP, SIN, SON, TAMS, ZAC; Red List: LC; banked. |
| *Pinus* *strobus* var. *chiapensis* Martínez; CHIS, GRO, OAX, PUE, VER; banked. |
| *Pinus* *tecunumanii* F.Schwerdtf. ex Eguiluz & J.P.Perry; CHIS, OAX; Red List: VU. |
| *Pinus* *teocote* Schiede ex Schltdl. & Cham.; AGS, CHIS, CHIH, COAH, CDMX, DGO, GTO, GRO, HGO, JAL, MEX, MICH, MOR, NAY, NLE, OAX, PUE, QRO, SLP, SIN, SON, TAMS, TLAX, VER, ZAC; Red List: LC; banked. |
| ***Pseudotsuga*** |
| *Pseudotsuga* *macrocarpa* (Vasey) Mayr; BCN; Red List: NT. |
| *Pseudotsuga menziesii* (Mirb.) Franco; CHIH, COAH, DGO, GRO, HGO, MEX, NLE, OAX, PUE, QRO, SLP, SIN, SON, TAMS, TLAX, VER, ZAC; Red List: LC; NOM-59: Pr; useful (ENVIRONMENTAL USES). |
|  |
| **Piperaceae** |
| ***Piper*** |
| *Piper rosei* C.DC.; COL, JAL, SIN (endemic). |
|  |
| **Platanaceae** |
| ***Platanus*** |
| *Platanus* *gentryi* Nixon & J.M.Poole; CHIH, SIN, SON (endemic). |
| *Platanus* *lindeniana* M.Martens & Galeotti. |
| *Platanus mexicana* Moric.; CHIS, COAH, CDMX, GTO, HGO, MEX, NLE, OAX, PUE, QRO, SLP, TAMS, VER; banked; useful (MEDICINES, MATERIALS, FUELS, ENVIRONMENTAL USES). |
| *Platanus* *occidentalis* L.; COAH, NLE, SLP; Red List: LC. |
| *Platanus* *racemosa* Nutt.; BCN, CHIH, SIN, SON; banked; useful. |
| *Platanus* *rzedowskii* Nixon & J.M.Poole; COAH, NLE, QRO, TAMS, SLP, VER (endemic). |
|  |
| **Plocospermataceae** |
| ***Plocosperma*** |
| *Plocosperma buxifolium* Benth.; GRO, JAL, MICH, OAX, PUE, VER; Red List: LC; banked; useful (ANIMAL FOOD, MATERIALS, FUELS, ENVIRONMENTAL USES). |
|  |
| **Podocarpaceae** |
| ***Podocarpus*** |
| *Podocarpus matudae* Lundell; AGS, CHIS, COL, GRO, HGO, JAL, MICH, NAY, OAX, PUE, QRO, SLP, TAMS, VER; Red List: VU; NOM-59: Pr. |
| *Podocarpus oleifolius* D.Don; CHIS, OAX, VER; Red List: LC; useful (ENVIRONMENTAL USES). |
|  |
| **Polygonaceae** |
| ***Coccoloba*** |
| *Coccoloba* *acapulcensis* Standl.; CAM, CHIS, GRO, JAL, MICH, OAX, PUE, QROO, TAB, YUC. |
| *Coccoloba* *acuminata* Kunth; CHIS, QROO; useful (ENVIRONMENTAL USES). |
| *Coccoloba* *barbadensis* Jacq.; CAM, CHIS, COL, GRO, HGO, JAL, MEX, MICH, MOR, NAY, OAX, PUE, QRO, QROO, SLP, SIN, TAB, TAMS, VER, YUC; Red List: LC; useful (FOOD, MATERIALS). |
| *Coccoloba* *belizensis* Standl.; CAM, CHIS, QROO, VER; Red List: LC. |
| *Coccoloba* *caracasana* Meisn.; CHIS. |
| *Coccoloba* *chiapensis* Standl.; CHIS, OAX, VER (endemic). |
| *Coccoloba* *cozumelensis* Hemsl.; CAM, CHIS, GRO, QROO, TAB, YUC; Red List: LC. |
| *Coccoloba* *diversifolia* Jacq.; CHIS, OAX, QROO, SLP, VER, YUC. |
| *Coccoloba* *escuintlensis* Lundell; CHIS, VER. |
| *Coccoloba* *floresii* Ortiz-Díaz & Arnelas; CHIS (endemic). |
| *Coccoloba* *goldmanii* Standl.; CHIH, SIN, SON (endemic); banked. |
| *Coccoloba* *hirtella* Lundell; OAX, VER. |
| *Coccoloba* *humboldtii* Meisn.; CAM, CHIS, OAX, QROO, SLP, TAB, TAMS, VER, YUC (endemic); Red List: LC. |
| *Coccoloba* *jurgensenii* Lindau; COL, JAL, NAY, OAX (endemic). |
| *Coccoloba* *lapathifolia* Standl.; GRO (endemic). |
| *Coccoloba* *lehmannii* Lindau; CHIS, TAB; useful (ENVIRONMENTAL USES). |
| *Coccoloba* *liebmannii* Lindau; CHIS, COL, GRO, JAL, MICH, OAX, VER. |
| *Coccoloba* *lindaviana* R.A.Howard; CHIS, VER. |
| *Coccoloba* *lindeniana* (Benth.) Lindau; CHIS, TAB, VER; Red List: CR. |
| *Coccoloba* *montana* Standl.; CHIS, GRO, MICH, OAX, QROO, TAB, VER. |
| *Coccoloba* *ortizii* R.A.Howard; QROO, YUC (endemic). |
| *Coccoloba* *reflexiflora* Standl.; CAM, QROO, YUC. |
| *Coccoloba* *spicata* Lundell; CAM, CHIS, PUE, QROO, TAB, VER, YUC; Red List: LC; useful (ANIMAL FOOD, MATERIALS, FUELS). |
| *Coccoloba* *swartzii* Meisn.; QROO; useful (ENVIRONMENTAL USES). |
| *Coccoloba* *tuerckheimii* Donn.Sm.; CHIS, TAB, VER; Red List: LC. |
| *Coccoloba* *uvifera* (L.) L.; CAM, OAX, QROO, SIN, TAB, TAMS, VER, YUC; useful (FOOD). |
| *Coccoloba* *venosa* L.; CHIS, COL, GRO, JAL, OAX; useful (FOOD, ANIMAL FOOD, MEDICINES, MATERIALS, FUELS, SOCIAL USES). |
| ***Gymnopodium*** |
| *Gymnopodium* *floribundum* Rolfe; CAM, CHIS, OAX, QROO, TAB, VER, YUC; Red List: LC; useful (ANIMAL FOOD). |
| ***Neomillspaughia*** |
| *Neomillspaughia emarginata* (H.Gross) S.F.Blake; CAM, QROO, YUC; Red List: LC; useful (ANIMAL FOOD, FUELS). |
| ***Podopterus*** |
| *Podopterus cordifolius* Rose & Standl.; COL, GRO, JAL, MICH, OAX (endemic). |
| *Podopterus guatemalensis* S.F.Blake; OAX, VER. |
| *Podopterus mexicanus* Bonpl.; CAM, CHIS, COL, GRO, HGO, JAL, MICH, NAY, OAX, PUE, QRO, QROO, SLP, TAMS, VER, YUC; banked. |
| ***Ruprechtia*** |
| *Ruprechtia costata* Meisn.; GRO, OAX, VER. |
| *Ruprechtia fusca* Fernald; COL, GRO, JAL, MEX, MICH, MOR, OAX, PUE, SIN, TAB, VER (endemic); banked; useful (FOOD, MATERIALS, FUELS). |
| *Ruprechtia laevigata* Pendry; JAL, OAX, VER, YUC (endemic). |
| *Ruprechtia pallida* Standl.; COL, GRO, JAL, MICH. |
| *Ruprechtia standleyana* Cocucci; GRO, MEX, MICH, MOR, OAX, PUE (endemic); banked. |
|  |
| **Primulaceae** |
| ***Ardisia*** |
| *Ardisia* *austin-smithii* Lundell; CHIS, OAX. |
| *Ardisia* *breedlovei* Lundell; CHIS (endemic). |
| *Ardisia* *compressa* Kunth; CAM, CHIS, COL, DGO, GRO, HGO, JAL, MEX, MICH, MOR, NAY, OAX, PUE, QRO, QROO, SLP, SIN, TAB, TAMS, VER; Red List: LC; useful (FOOD, MEDICINES). |
| *Ardisia* *copeyana* Standl.; CHIS, OAX; Red List: LC. |
| *Ardisia* *escallonioides* Schltdl. & Cham.; CAM, CHIS, COL, GRO, HGO, JAL, MEX, MICH, OAX, PUE, QRO, QROO, SLP, TAMS, TAB, TAMS, VER, YUC; useful (FOOD). |
| *Ardisia* *hintonii* Lundell; CHIS, DGO, JAL, MICH, OAX, SLP, VER. |
| *Ardisia* *hyalina* Lundell; SLP, VER (endemic). |
| *Ardisia* *liebmannii* Oerst.; CHIS, HGO, OAX, PUE, VER (endemic). |
| *Ardisia* *revoluta* Kunth; CAM, CHIS, CHIH, COL, DGO, GRO, HGO, JAL, MEX, MICH, MOR, NAY, OAX, PUE, QRO, QROO, SLP, SIN, SON, TAB, VER, YUC. |
| *Ardisia* *tacanensis* Lundell; CHIS. |
| *Ardisia* *tuerckheimii* Donn.Sm.; CHIS, OAX, TAB, VER. |
| *Ardisia* *venosa* Mast. ex Donn.Sm.; AGS, CHIS, COL, GRO, HGO, JAL, MEX, MICH, OAX, VER. |
| *Ardisia* *verapazensis* Donn.Sm.; CHIS, OAX, TAB, VER. |
| ***Bonellia*** |
| *Bonellia macrocarpa* (Cav.) B.Ståhl & Källersjö; BCN, BCS, CAM, CHIS, CHIH, COL, DGO, GRO, JAL, MEX, MICH, MOR, NAY, OAX, PUE, QRO, QROO, SLP, SIN, SON, TAB, TAMS, VER, YUC; Red List: LC; banked. |
| ***Ctenardisia*** |
| *Ctenardisia purpusii* (Brandegee) Lundell; CHIS (endemic). |
| ***Myrsine*** |
| *Myrsine coriacea* (Sw.) R.Br. ex Roem. & Schult.; CAM, CHIS, CHIH, COL, DGO, GRO, HGO, JAL, MEX, MICH, MOR, NAY, NLE, OAX, PUE, QRO, QROO, SLP, SIN, SON, TAMS, VER; banked. |
| *Myrsine juergensenii* (Mez) Ricketson & Pipoly; CAM, CHIS, COL, GRO, JAL, MEX, MICH, MOR, NAY, OAX, PUE, QROO, TAB, VER. |
| ***Parathesis*** |
| *Parathesis* *calzadae* Lundell; OAX, VER (endemic). |
| *Parathesis* *chiapensis* Fernald; CHIS, OAX, VER. |
| *Parathesis* *cintalapana* Lundell; CHIS (endemic). |
| *Parathesis* *kochii* Lundell; OAX, VER (endemic). |
| *Parathesis* *leptopa* Lundell; CHIS, GRO, HGO, OAX, QRO, SLP, VER. |
| *Parathesis* *macronema* Bullock; CHIS, GRO, JAL, MICH, OAX, PUE, VER (endemic). |
| *Parathesis* *minutiflora* Lundell; OAX (endemic). |
| *Parathesis* *oerstediana* Mez; OAX (endemic). |
| *Parathesis* *psychotrioides* Lundell; CHIS, HGO, OAX, PUE, TAB, VER (endemic). |
| *Parathesis* *rekoi* Standl.; OAX (endemic). |
| *Parathesis* *villosa* Lundell; CHIS, COL, DGO, GRO, JAL, MEX, MICH, NAY, OAX, SIN, VER (endemic). |
| *Parathesis* *vulgata* Lundell; CHIS, GRO, OAX; Red List: EN. |
| *Parathesis* *wendtii* Lundell; OAX, VER (endemic). |
| ***Stylogyne*** |
| *Stylogyne turbacensis* (Kunth) Mez; CHIS, OAX, TAB, VER; Red List: LC. |
|  |
| **Proteaceae** |
| ***Roupala*** |
| *Roupala mexicana* K.S.Edwards & Prance; OAX, TAB, VER (endemic). |
| *Roupala montana* Aubl.; CHIS, GRO, HGO, OAX, QRO, SLP, TAB, VER; useful (MEDICINES). |
|  |
| **Putranjivaceae** |
| ***Drypetes*** |
| *Drypetes brownii* Standl.; CAM, CHIS, QROO, TAB, VER; Red List: LC. |
| *Drypetes gentryi* Monach.; CHIH, COL, GRO, JAL, MICH, NAY, SIN, SON (endemic). |
| *Drypetes lateriflora* (Sw.) Krug & Urb.; CAM, CHIS, CHIH, COL, GTO, GRO, HGO, JAL, MEX, OAX, PUE, QRO, QROO, SLP, SON, TAB, TAMS, VER, YUC. |
|  |
| **Resedaceae** |
| ***Forchhammeria*** |
| *Forchhammeria* *hintonii* Paul G.Wilson; GRO, OAX (endemic). |
| *Forchhammeria* *macrocarpa* Standl.; GRO, MOR, OAX, PUE (endemic); banked; useful (ANIMAL FOOD, MATERIALS, FUELS). |
| *Forchhammeria* *pallida* Liebm.; COL, GRO, JAL, MEX, MICH, OAX (endemic). |
| *Forchhammeria* *sessilifolia* Standl.; COL, JAL, MICH, NAY (endemic). |
| *Forchhammeria* *trifoliata* Radlk. ex Millsp.; CAM, CHIS, OAX, QROO, SLP, TAB, TAMS, VER, YUC. |
| *Forchhammeria* *watsonii* Rose; BCN, BCS, SIN, SON (endemic); banked. |
|  |
| **Rhamnaceae** |
| ***Adolphia*** |
| *Adolphia californica* S.Watson; BCN; banked. |
| ***Ceanothus*** |
| *Ceanothus* *caeruleus* Lag.; AGS, CHIS, CHIH, COAH, COL, CDMX, DGO, GTO, GRO, HGO, JAL, MEX, MICH, MOR, NAY, NLE, OAX, PUE, QRO, QROO, SLP, SIN, SON, TAMS, TLAX, VER, YUC, ZAC; useful (MEDICINES, ENVIRONMENTAL USES). |
| *Ceanothus* *crassifolius* Torr.; BCN. |
| *Ceanothus* *cyaneus* Eastw.; BCN; useful (ENVIRONMENTAL USES). |
| *Ceanothus* *leucodermis* Greene; BCN; banked. |
| *Ceanothus* *oliganthus* Nutt.; BCN; banked. |
| *Ceanothus* *spinosus* Nutt.; BCN. |
| ***Colubrina*** |
| *Colubrina* *arborescens* (Mill.) Sarg.; CAM, CHIS, GRO, QROO, TAB, VER, YUC; banked. |
| *Colubrina* *elliptica* (Sw.) Brizicky & W.L.Stern; CAM, CHIS, COL, COAH, DGO, GTO, GRO, HGO, JAL, MEX, MICH, NAY, OAX, PUE, QRO, QROO, SLP, TAMS, VER, YUC, ZAC. |
| *Colubrina* *greggii* S.Watson; BCN, CAM, CHIS, COAH, DGO, GTO, GRO, HGO, JAL, MEX, MOR, NLE, OAX, PUE, QRO, QROO, SLP, TAMS, VER, YUC, ZAC; banked; useful (MATERIALS). |
| *Colubrina* *heteroneura* (Griseb.) Standl.; CAM, CHIS, COL, DGO, GTO, GRO, HGO, JAL, MICH, NAY, OAX, QRO, SLP, SIN, TAMS, VER, YUC. |
| *Colubrina* *triflora* Brongn. ex Sweet; AGS, BCS, CHIS, CHIH, COL, DGO, GTO, GRO, JAL, MEX, MICH, MOR, NAY, OAX, PUE, QRO, SLP, SIN, SON, TAMS, VER, ZAC; banked; useful (MEDICINES). |
| *Colubrina* *yucatanensis* (M.C.Johnst.) G.L.Nesom; CAM, QROO, YUC. |
| ***Frangula*** |
| *Frangula* *capreifolia* (Schltdl.) Grubov; CHIS, COL, GRO, HGO, JAL, MICH, NAY, OAX, PUE, QRO, SLP, TAMS, VER; Red List: LC; banked. |
| *Frangula* *chimalapensis* (R.Fernández) A.Pool; OAX (endemic). |
| *Frangula* *discolor* (Donn.Sm.) Grubov; CHIS, COL, JAL, NAY, OAX; Red List: LC. |
| *Frangula* *grandiflora* A.Pool; CHIS. |
| *Frangula* *hintonii* (M.C.Johnst. & L.A.Johnst.) A.Pool; COL, GRO, JAL, MEX, MICH, MOR, OAX, ZAC (endemic). |
| *Frangula* *wendtii* (Ishiki) A.Pool; OAX (endemic). |
| ***Karwinskia*** |
| *Karwinskia* *calderonii* Standl.; CAM, CHIS, GRO, OAX, QROO, SIN, YUC. |
| *Karwinskia* *humboldtiana* (Willd. ex Schult.) Zucc.; AGS, BCN, BCS, CAM, CHIS, CHIH, COAH, COL, DGO, GTO, GRO, HGO, JAL, MEX, MICH, MOR, NAY, NLE, OAX, PUE, QRO, QROO, SLP, SIN, SON, TAMS, VER, YUC, ZAC; banked; useful. |
| *Karwinskia* *johnstonii* R.Fern.; GRO, JAL, MEX, MICH, MOR (endemic). |
| *Karwinskia* *latifolia* Standl.; AGS, COL, DGO, JAL, MICH, NAY, SIN, ZAC (endemic); useful (MEDICINES, POISONS). |
| *Karwinskia* *mollis* Schltdl.; GTO, GRO, HGO, JAL, MEX, MICH, NLE, OAX, PUE, QRO, SLP, TAMS, VER, ZAC (endemic); banked; useful (ANIMAL FOOD, FUELS, ENVIRONMENTAL USES). |
| *Karwinskia* *rzedowskii* R.Fern.; DGO, JAL, NAY, ZAC (endemic). |
| *Karwinskia* *tehuacana* R.Fern. & N.Waksman; MICH, PUE (endemic). |
| *Karwinskia* *umbellata* (Cav.) Schltdl.; GRO, MOR, OAX, PUE (endemic). |
| ***Krugiodendron*** |
| *Krugiodendron ferreum* (Vahl) Urb.; CAM, CHIS, GTO, GRO, HGO, MEX, OAX, PUE, QRO, QROO, SLP, TAMS, VER, YUC; useful (MEDICINES, MATERIALS). |
| ***Rhamnus*** |
| *Rhamnus biglandulosa* Sessé & Moc.; GRO (endemic). |
| *Rhamnus crocea* Nutt; BCN, BCS, CHIH, SON; banked. |
| ***Sarcomphalus*** |
| *Sarcomphalus* *amole* (Sessé & Moc.) Hauenschild; BCS, CHIS, CHIH, COAH, COL, DGO, GTO, GRO, HGO, JAL, MEX, MICH, MOR, NAY, NLE, OAX, PUE, QRO, SLP, SIN, SON, TAMS, VER, ZAC (endemic); banked; useful (ANIMAL FOOD, MEDICINES, MATERIALS, FUELS, ENVIRONMENTAL USES, SOCIAL USES). |
| *Sarcomphalus* *guatemalensis* (Hemsl.) Hauenschild; CHIS, OAX. |
| *Sarcomphalus* *mexicanus* (Rose) Hauenschild; CHIS, COL, GRO, JAL, MICH, OAX, PUE, QROO, SIN, VER, ZAC (endemic); banked; useful. |
| *Sarcomphalus* *obtusifolius* (Hook. ex Torr. & A.Gray) Hauenschild; BCN, BCS, CHIH, COAH, DGO, NLE, SLP, SIN, SON, TAMS, VER, ZAC; banked; useful. |
| *Sarcomphalus* *pedunculatus* (Brandegee) Hauenschild; GRO, OAX, PUE (endemic); banked; useful (MATERIALS, SOCIAL USES). |
| *Sarcomphalus* *yucatanensis* (Standl.) Hauenschild; CAM, QROO, YUC (endemic). |
| ***Ziziphus*** |
| *Ziziphus acuminata* Benth.; GRO, MOR, OAX. |
|  |
| **Rhizophoraceae** |
| ***Rhizophora*** |
| *Rhizophora* × *harrisonii* Leechm.; CHIS, TAMS, VER; useful (ENVIRONMENTAL USES). |
| *Rhizophora* *mangle* L.; BCN, BCS, CAM, CHIS, COL, GRO, JAL, MICH, NAY, OAX, PUE, QROO, SIN, SON, TAB, TAMS, VER, YUC; Red List: LC; NOM-59: A; banked; useful (ANIMAL FOOD, MEDICINES, MATERIALS, FUELS). |
|  |
| **Rosaceae** |
| ***Adenostoma*** |
| *Adenostoma sparsifolium* Torr.; BCN; banked. |
| ***Amelanchier*** |
| *Amelanchier utahensis* Koehne; BCN, CHIH; useful (ENVIRONMENTAL USES). |
| ***Cercocarpus*** |
| *Cercocarpus fothergilloides* Kunth; COAH, GRO, HGO, MEX, NLE, OAX, PUE, QRO, SLP, TAMS, VER, ZAC (endemic); banked; useful. |
| *Cercocarpus pringlei* (C.K.Schneid.) Rydb.; GRO, HGO, OAX, PUE, QRO, VER (endemic); banked. |
| *Cercocarpus macrophyllus* C.K. Schneid.; AGS, COAH, COL, CDMX, DGO, GTO, GRO, HGO, JAL, MEX, MICH, NAY, NLE, OAX, PUE, QRO, SLP, SIN, TAMS, VER, ZAC (endemic); Red List: LC; banked. |
| ***Crataegus*** |
| *Crataegus* *aurescens* J.B.Phipps; COAH, NLE (endemic). |
| *Crataegus* *baroussana* Eggl.; COAH, NLE, TAMS (endemic); banked. |
| *Crataegus* *crus-gall*i L.; COAH, NLE; Red List: LC; useful (ENVIRONMENTAL USES). |
| *Crataegus* *cuprina* J.B.Phipps; COAH, NLE (endemic). |
| *Crataegus* *gracilior* J.B.Phipps; CHIS, COL, CDMX, GTO, GRO, HGO, JAL, MEX, MICH, NLE, OAX, PUE, QRO, SLP, TAMS, VER (endemic); banked; useful (FOOD). |
| *Crataegus* *grandifolia* J.B.Phipps; COAH, NLE (endemic). |
| *Crataegus* greggiana Eggl.; COAH, NLE, TAMS. |
| *Crataegus* *johnstonii* J.B.Phipps; COAH (endemic). |
| *Crataegus mexicana* Moc. & Sessé ex DC.; AGS, CHIS, COAH, COL, CDMX, DGO, GTO, GRO, HGO, JAL, MEX, MICH, MOR, NLE, OAX, PUE, QRO, SLP, SIN, TAMS, TLAX, VER, ZAC; Red List: LC; banked; useful (FOOD). |
| *Crataegus* *rosei* Eggl.; CHIH, COAH, DGO, GTO, HGO, JAL, NLE, QRO, SLP, SIN, TAMS, VER (endemic); banked. |
| *Crataegus* *sulfurea* J.B.Phipps; COAH, NLE (endemic). |
| *Crataegus* *tracyi* Ashe ex Eggl.; COAH, NLE, SLP. |
| *Crataegus* *uniflora* Münchh.; COAH, TAMS. |
| *Crataegus* *lindenii* Stapf; (endemic). |
| ***Geum*** |
| *Geum mexicanum* Rydb.; MEX, MOR (endemic). |
| ***Heteromeles*** |
| *Heteromeles arbutifolia* (Lindl.) M.Roem.; BCN, BCS; banked. |
| ***Holodiscus*** |
| *Holodiscus* *argenteus* (L.f.) Maxim.; CHIS, OAX; Red List: LC. |
| *Holodiscus* *australis* A.Heller; SLP, ZAC. |
| *Holodiscus* *microphyllus* Rydb.; BCN. |
| *Holodiscus* *orizabae* Ley; VER (endemic). |
| ***Lindleya*** |
| *Lindleya mespiloides* Kunth; AGS, CHIH, COAH, DGO, GTO, HGO, NLE, OAX, PUE, QRO, SLP, TAMS, VER, ZAC (endemic); banked. |
| ***Malacomeles*** |
| *Malacomeles denticulata* (Kunth) Decne.; AGS, CHIS, CHIH, COAH, CDMX, DGO, GTO, HGO, JAL, MEX, NLE, OAX, PUE, QRO, SLP, TAMS, TLAX, VER, ZAC; banked. |
| *Malacomeles paniculata* (Rehder) J.B.Phipps; COAH, NLE, SLP, TAMS, VER (endemic). |
| ***Photinia*** |
| *Photinia* *guerreris* J.B.Phipps; GRO (endemic). |
| *Photinia* *matudae* Lundell; CHIS, VER. |
| *Photinia mexicana* Hemsl.; CHIS, JAL, MICH, OAX, QRO, SLP, VER (endemic). |
| *Photinia* *microcarpa* Standl.; CHIS, GRO, JAL, MEX, MICH, OAX, QRO, SLP, VER. |
| *Photinia* *oblongifolia* Standl.; JAL, MICH, NAY (endemic). |
| ***Pourthiaea*** |
| *Pourthiaea arguta* (Wall. ex Lindl.) Decne.; COL, JAL (endemic). |
| ***Prunus*** |
| *Prunus* *annularis* Koehne; CHIS, HGO, VER; Red List: LC. |
| *Prunus* *barbata* Koehne; CHIS, GRO, MICH. |
| *Prunus* *brachybotrya* Zucc.; CHIS, COL, CDMX, GRO, HGO, JAL, MEX, MICH, MOR, NLE, OAX, PUE, QRO, SLP, SIN, TAMS, VER, ZAC. |
| *Prunus* *chiapensis* Standl. & L.O.Williams ex Ant.Molina; CHIS. |
| *Prunus* *cortapico* Kerber ex Koehne; CHIS, COL, GRO, JAL, MEX, MICH, MOR, OAX, PUE, SIN, VER, ZAC. |
| *Prunus* *erythroxylon* Koehne; COL, DGO, GRO, MICH, NAY, VER (endemic). |
| *Prunus* *ferruginea* (DC. ex Ser.) Steud.; DGO, GRO, JAL, MEX, MICH, MOR, NAY, SIN, ZAC (endemic). |
| *Prunus* *gentryi* Standl.; CHIH, HGO, SIN, SON, VER (endemic). |
| *Prunus* *guatemalensis* I.M.Johnst.; CHIS, OAX. |
| *Prunus* *ilicifolia* (Nutt. ex Hook. & Arn.) D.Dietr.; BCN, BCS; banked; useful (ENVIRONMENTAL USES). |
| *Prunus* *lyonii* (Eastw.) Sarg.; BCN, BCS. |
| *Prunus* *matudae* Lundell; CHIS, HGO, OAX, PUE, VER (endemic). |
| *Prunus* *myrtifolia* (L.) Urb.; CAM, QROO. |
| *Prunus* *occidentalis* Sw.; CHIS. |
| *Prunus* *ochoterenae* D.Ramírez; MOR (endemic). |
| *Prunus* *rhamnoides* Koehne; CHIS, CHIH, DGO, GTO, GRO, HGO, JAL, MEX, MICH, OAX, SLP, SON, TAMS, TLAX, VER. |
| *Prunus* *salasii* Standl.; CHIS. |
| *Prunus* *samydoides* Schltdl.; HGO, PUE, QRO, SLP, VER (endemic). |
| *Prunus* *serotina* Ehrh.; AGS, BCS, CHIS, CHIH, COAH, COL, CDMX, DGO, GTO, GRO, HGO, JAL, MEX, MICH, MOR, NAY, NLE, OAX, PUE, QRO, SLP, SIN, SON, TAMS, TLAX, VER, ZAC; Red List: LC; banked; useful (FOOD, MEDICINES, POISONS, MATERIALS, FUELS, SOCIAL USES). |
| *Prunus* *tartarea* Lundell; CHIS, OAX (endemic). |
| *Prunus* *tetradenia* Koehne; CHIS, COL, HGO, JAL, MEX, MICH, NAY, OAX, PUE, QRO, SLP, TAMS, VER (endemic). |
| *Prunus* *tuberculata* Koehne; CHIS, OAX (endemic). |
| *Prunus* *zinggii* Standl.; CHIH, COL, GRO, JAL, MICH, MOR, OAX, SON (endemic). |
| ***Vauquelinia*** |
| *Vauquelinia australis* Standl.; OAX, PUE (endemic); banked. |
| *Vauquelinia californica* (Torr.) Sarg.; BCN, BCS, CHIH, COAH, DGO, SON. |
| *Vauquelinia corymbosa* Corrêa ex Bonpl.; CHIH, COAH, DGO, GTO, HGO, NLE, QRO, SLP, TAMS, VER, ZAC; banked; useful. |
|  |
| **Rubiaceae** |
| ***Alibertia*** |
| *Alibertia edulis* (Rich.) A.Rich. ex DC.; CAM, CHIS, OAX, PUE, QROO, SIN, TAB, VER, YUC. |
| ***Alseis*** |
| *Alseis hondurensis* Standl.; CHIS, OAX, VER. |
| *Alseis yucatanensis* Standl.; CAM, CHIS, QROO, TAB, YUC; useful (MATERIALS, ENVIRONMENTAL USES). |
| ***Amaioua*** |
| *Amaioua glomerulata* (Lam. ex Poir.) Delprete & C.H.Perss.; CHIS, OAX, TAB, VER. |
| ***Appunia*** |
| *Appunia guatemalensis* Donn.Sm.; CHIS, QROO, TAB, VER; Red List: LC. |
| ***Arachnothryx*** |
| *Arachnothryx* *atravesadensis* (Lorence) Borhidi; OAX (endemic). |
| *Arachnothryx* *bertieroides* (Standl.) Borhidi; CHIS. |
| *Arachnothryx* *buddleioides* (Benth.) Planch.; CHIS, COL, GRO, JAL, MEX, MICH, NAY, OAX, TAB, VER. |
| *Arachnothryx* *capitellata* (Hemsl.) Borhidi; CHIS, GRO, HGO, JAL, OAX, PUE, VER; banked; useful (MATERIALS). |
| *Arachnothryx* *chimalaparum* Lorence ex Borhidi; OAX (endemic). |
| *Arachnothryx* *ginetteae* (Lorence) Borhidi; OAX (endemic). |
| *Arachnothryx* *guerrerensis* (Lorence) Borhidi; GRO (endemic). |
| *Arachnothryx* *izabalensis* (Standl. & Steyerm.) Borhidi; OAX, VER. |
| *Arachnothryx* *jaliscensis* Borhidi & E.Martínez; JAL (endemic). |
| *Arachnothryx* *jurgensenii* (Hemsl.) Borhidi; CHIS, COL, GTO, GRO, JAL, MEX, MICH, NAY, OAX, SIN. |
| *Arachnothryx* *laniflora* (Benth.) Planch.; CHIS, GRO, OAX, VER. |
| *Arachnothryx* *lineolata* Borhidi; CHIS (endemic). |
| *Arachnothryx* *linguiformis* (Hemsl.) Borhidi; CHIS. |
| *Arachnothryx* *macrocalyx* (Standl. & Steyerm.) Borhidi; CHIS, OAX. |
| *Arachnothryx* *michoacana* Borhidi; MICH (endemic). |
| *Arachnothryx* *pauciflora* Borhidi; CHIS (endemic). |
| *Arachnothryx* *purpurea* (Lorence) Borhidi; CHIS, OAX (endemic). |
| *Arachnothryx* *pyramidalis* (Lundell) Borhidi; CHIS, GRO, OAX (endemic). |
| *Arachnothryx* *rufescens* (B.L.Rob.) Borhidi; CHIS, OAX. |
| *Arachnothryx* *rzedowskii* (Lorence) Borhidi; GRO (endemic). |
| *Arachnothryx* *sanchezii* Borhidi & Salas-Mor.; OAX (endemic). |
| *Arachnothryx* *scabra* (Hemsl.) Borhidi; CHIS, GRO, OAX (endemic). |
| *Arachnothryx* *secundiflora* (B.L.Rob.) Borhidi; CHIS, OAX, TAB, VER. |
| *Arachnothryx* *septicidalis* (B.L.Rob.) Borhidi; CHIS, OAX, VER. |
| *Arachnothryx* *sousae* Borhidi; CHIS (endemic). |
| *Arachnothryx* *stachyoidea* (Donn.Sm.) Borhidi; CHIS, OAX, TAB, VER. |
| *Arachnothryx* *tacanensis* (Lundell) Borhidi; CHIS. |
| *Arachnothryx* *tenorioi* (Lorence) Borhidi; GRO, OAX (endemic). |
| *Arachnothryx* *tuxtlensis* (Lorence & Cast.-Campos) Borhidi; VER (endemic). |
| *Arachnothryx* *uxpanapensis* (Lorence & Cast.-Campos) Borhidi; OAX, VER (endemic). |
| *Arachnothryx* *wendtii* (Lorence & Cast.-Campos) Borhidi; OAX, VER (endemic). |
| *Arachnothryx* *axillaris* Torr.-Montúfar & Ochot.-Booth; (endemic). |
| ***Balmea*** |
| *Balmea stormiae* Martínez; CHIS, COL, GRO, JAL, MEX, MICH, NAY, OAX, PUE, TAB, VER; Red List: EN; NOM-59: Pr; Cites: I. |
| ***Bertiera*** |
| *Bertiera guianensis* Aubl.; CHIS, OAX, TAB, VER; useful (MEDICINES). |
| ***Blepharidium*** |
| *Blepharidium guatemalense* Standl.; CAM, CHIS, TAB; Red List: EN. |
| ***Calycophyllum*** |
| *Calycophyllum candidissimum* (Vahl) DC.; CAM, CHIS, GRO, OAX, PUE, QROO, VER; banked; useful (ANIMAL FOOD, MEDICINES, MATERIALS, ENVIRONMENTAL USES). |
| ***Chiococca*** |
| *Chiococca alba* (L.) Hitchc.; BCS, CAM, CHIS, COAH, COL, DGO, GTO, GRO, HGO, JAL, MEX, MICH, MOR, NAY, NLE, OAX, PUE, QRO, QROO, SLP, SIN, SON, TAB, TAMS, VER, YUC, ZAC; Red List: LC; banked; useful (MEDICINES, FUELS, SOCIAL USES). |
| *Chiococca phaenostemon* Schltdl.; CHIS, GRO, OAX, PUE, SLP, VER, YUC. |
| *Chiococca sessilifolia* Miranda; CHIS, OAX, VER (endemic). |
| ***Chione*** |
| *Chione venosa* (Sw.) Urb.; CHIS, HGO, MICH, NLE, OAX, PUE, QRO, SLP, TAB, TAMS, VER. |
| ***Chomelia*** |
| *Chomelia* *barbata* Standl.; COL, JAL, NAY, OAX, SIN, VER (endemic). |
| *Chomelia* *brachypoda* Donn.Sm.; CHIS, OAX. |
| *Chomelia* *breedlovei* Borhidi; CHIS, OAX (endemic). |
| *Chomelia* *crassifolia* Borhidi; OAX, TAB (endemic). |
| *Chomelia* *longituba* (Borhidi) Borhidi; CHIS, VER. |
| *Chomelia* *pringlei* S.Watson; QRO, SLP, VER (endemic). |
| *Chomelia* *spinosa* Jacq.; CHIS, GRO, OAX, TAB, VER. |
| *Chomelia* *tenuiflora* Benth.; VER; Red List: LC. |
| ***Cosmibuena*** |
| *Cosmibuena matudae* (Standl.) L.O.Williams; CHIS. |
| ***Cosmocalyx*** |
| *Cosmocalyx spectabilis* Standl.; CAM, GRO, MICH, QROO, YUC (endemic). |
| ***Coussarea*** |
| *Coussarea imitans* L.O.Williams; CHIS, OAX, VER; Red List: EN. |
| *Coussarea mexicana* Standl.; CHIS, OAX, TAB, VER (endemic); Red List: EN. |
| *Coussarea rafa-torresii* Borhidi; OAX (endemic); Red List: CR. |
| ***Coutaportla*** |
| *Coutaportla guatemalensis* (Standl.) Lorence; CHIS, VER. |
| ***Coutarea*** |
| *Coutarea hexandra* (Jacq.) K.Schum.; CAM, CHIS, GTO, GRO, OAX, QROO, TAB, VER; useful (MATERIALS). |
| ***Deppea*** |
| *Deppea* *chimalaparum* Borhidi & E.Martínez; OAX (endemic). |
| *Deppea* *grandiflora* Schltdl.; CHIS, GRO, JAL, OAX, SLP, TAB, VER. |
| *Deppea* *guerrerensis* Dwyer & Lorence; GRO, JAL, MEX, MICH, OAX (endemic). |
| *Deppea* *keniae* Borhidi & Saynes; OAX (endemic). |
| *Deppea* *nitida* Borhidi & Salas-Mor.; OAX (endemic). |
| *Deppea* *oaxacana* Lorence; GRO, OAX (endemic). |
| *Deppea* *pauciflora* Borhidi & E.Martínez; NLE, TAMS (endemic). |
| *Deppea* *rupicola* Borhidi & K.Velasco; OAX (endemic). |
| *Deppea* *scoti* (J.H.Kirkbr.) Lorence; OAX, PUE (endemic). |
| *Deppea* *serboi* Borhidi & K.Velasco; OAX (endemic). |
| *Deppea* *sousae* Borhidi; GRO (endemic). |
| *Deppea* *splendens* Breedlove & Lorence; CHIS, OAX (endemic). |
| ***Donnellyanthus*** |
| *Donnellyanthus* *deamii* (Donn.Sm.) Borhidi; CHIS, OAX. |
| ***Exostema*** |
| *Exostema caribaeum* (Jacq.) Schult.; CAM, CHIS, COL, GTO, GRO, HGO, JAL, MEX, MICH, MOR, NLE, OAX, PUE, QRO, QROO, SLP, TAB, TAMS, VER, YUC, ZAC; useful (MEDICINES). |
| ***Faramea*** |
| *Faramea* *brachysiphon* Standl.; CHIS, OAX, VER. |
| *Faramea* *oaxacensis* Borhidi; OAX (endemic). |
| *Faramea* *occidentalis* (L.) A.Rich.; CHIS, COL, GRO, JAL, NAY, NLE, OAX, PUE, QRO, SLP, TAB, VER. |
| *Faramea* *schultesii* Standl.; CHIS, OAX, VER (endemic). |
| ***Genipa*** |
| *Genipa americana* L.; AGS, CAM, CHIS, COL, GRO, JAL, MICH, NAY, OAX, SIN, TAB, VER, ZAC; useful (MEDICINES, MATERIALS, SOCIAL USES). |
| ***Glossostipula*** |
| *Glossostipula concinna* (Standl.) Lorence; CHIS, COL, GTO, GRO, JAL, OAX, QRO, VER. |
| ***Gonzalagunia*** |
| *Gonzalagunia tacanensis* Lundell; CHIS, GRO, TAB, VER. |
| *Gonzalagunia thyrsoidea* (Donn.Sm.) B.L.Rob.; CHIS, PUE, TAB. |
| ***Guettarda*** |
| *Guettarda* *combsii* Urb.; CAM, CHIS, QROO, TAB, VER, YUC; useful (MATERIALS). |
| *Guettarda* *crispiflora* Vahl; CHIS; Red List: LC. |
| *Guettarda* *dealbata* M.Martens & Galeotti; OAX (endemic). |
| *Guettarda* *elliptica* Sw.; CAM, CHIS, COL, GRO, JAL, MEX, MICH, NAY, OAX, QROO, SIN, TAB, VER, YUC. |
| *Guettarda* *elongata* Borhidi, K.Velasco & Vásq.-Mart.; OAX (endemic). |
| *Guettarda* *filipes* Standl.; CAM, DGO, GRO, JAL, MICH, NAY, OAX, QROO, SIN, YUC (endemic). |
| *Guettarda* *foliacea* Standl.; CHIS, TAB. |
| *Guettarda* *guerrerensis* Borhidi; GRO, JAL (endemic). |
| *Guettarda* *quadrifida* Borhidi & Reyes-García; CHIS (endemic). |
| *Guettarda* *subcapitata* C.M.Taylor; CAM, CHIS, OAX, QROO. |
| ***Hamelia*** |
| *Hamelia* *barbata* Standl.; CHIS, OAX, TAB, VER. |
| *Hamelia* *calycosa* Donn.Sm.; CHIS, COL, GRO, JAL, MICH, NAY, OAX, TAB, VER; Red List: LC. |
| *Hamelia* *longipes* Standl.; CHIS, GRO, OAX, TAB, VER. |
| *Hamelia* *patens* Jacq.; CAM, CHIS, COL, CDMX, DGO, GRO, HGO, JAL, MEX, MICH, MOR, NAY, NLE, OAX, PUE, QRO, QROO, SLP, SIN, TAB, TAMS, VER, YUC; Red List: LC; banked; useful (MEDICINES). |
| *Hamelia* *rostrata* Bartl. ex DC.; CHIS, COL, DGO, GRO, JAL, MEX, MICH, MOR, NAY, OAX, SIN, VER (endemic); banked. |
| *Hamelia* *rovirosae* Wernham; CAM, CHIS, TAB, VER; NOM-59: Pr. |
| *Hamelia* *xorullensis* Kunth; CHIS, CHIH, COL, CDMX, DGO, GRO, JAL, MEX, MICH, MOR, NAY, OAX, SIN, SON, ZAC (endemic). |
| ***Hintonia*** |
| *Hintonia latiflora* (Sessé & Moc. ex DC.) Bullock; AGS, CAM, CHIS, CHIH, COL, DGO, GRO, JAL, MEX, MICH, MOR, NAY, OAX, PUE, SIN, SON, VER, ZAC; banked; useful (MEDICINES). |
| *Hintonia lumana* (Baill.) Bullock; CHIS, VER. |
| ***Hoffmannia*** |
| *Hoffmannia cauliflora* Hemsl.; CHIS. |
| ***Machaonia*** |
| *Machaonia acuminata* Bonpl.; CAM, CHIS, COL, JAL, MICH, OAX, PUE, QROO, SLP, SIN, TAB, TAMS, VER, YUC; Red List: LC. |
| *Machaonia erythrocarpa* (Standl.) Borhidi; CHIS, COL, HGO, JAL, MICH, NAY, OAX, PUE, SIN, VER. |
| *Machaonia lindeniana* Baill.; CAM, CHIS, QROO, TAB, YUC; Red List: LC; useful (MEDICINES). |
| ***Morinda*** |
| *Morinda panamensis* Seem.; CHIS, OAX, QROO, TAB, VER; Red List: LC. |
| ***Nernstia*** |
| *Nernstia mexicana* (Zucc. & Mart. ex DC.) Urb.; HGO, QRO, SLP, VER (endemic); banked. |
| ***Palicourea*** |
| *Palicourea* *berteroana* (DC.) Borhidi; CHIS, OAX, PUE, TAMS, VER; useful (MEDICINES). |
| *Palicourea* *brachiata* (Sw.) Borhidi; CHIS, OAX, PUE, VER; useful (MEDICINES, MATERIALS). |
| *Palicourea* *breedlovei* (Lorence) Lorence; CHIS (endemic). |
| *Palicourea* *elata* (Sw.) Borhidi; CHIS, OAX, PUE, TAB, VER. |
| *Palicourea* *faxlucens* (Lorence & Dwyer) Lorence; OAX, TAB, VER (endemic). |
| *Palicourea* *galeottiana* M.Martens; CAM, CHIS, GRO, HGO, OAX, PUE, QRO, QROO, SLP, TAB, TAMS, VER, YUC (endemic). |
| *Palicourea* *glomerulata* (Donn.Sm.) Borhidi; CHIS. |
| *Palicourea* *guianensis* Aubl.; CAM, CHIS, OAX, TAB, VER. |
| *Palicourea* *hebeclada* (DC.) Borhidi; OAX, QROO, VER. |
| *Palicourea* *juarezana* (C.M.Taylor & Lorence) Borhidi; CHIS, OAX (endemic). |
| *Palicourea* *leucantha* D.A.Sm.; OAX. |
| *Palicourea* *megalantha* (Lorence) Lorence; CHIS, OAX, VER (endemic). |
| *Palicourea* *neopurpusii* C.M.Taylor; CHIS. |
| *Palicourea* *padifolia* (Willd. ex Schult.) C.M.Taylor & Lorence; CHIS, GRO, HGO, OAX, PUE, SLP, TAB, VER; banked. |
| *Palicourea* *perotensis* (Cast.-Campos) Borhidi; VER (endemic). |
| *Palicourea* *pubescens* (Sw.) Borhidi; CAM, CHIS, COL, GRO, JAL, MICH, NAY, OAX, PUE, QRO, QROO, SLP, TAB, VER, YUC. |
| *Palicourea* *simiarum* (Standl.) Borhidi; CHIS, GRO, HGO, OAX, PUE, TAB, VER. |
| *Palicourea* *sousae* (Lorence & Dwyer) Lorence; OAX, VER (endemic). |
| *Palicourea* *tetragona* (Donn.Sm.) C.M.Taylor; CHIS, GRO, OAX, PUE, SLP, TAB, VER; Red List: LC. |
| *Palicourea* *thornei* (Lorence) Lorence; CHIS (endemic). |
| *Palicourea* *triphylla* DC.; CHIS, TAB, VER. |
| *Palicourea* *veracruzensis* (Lorence & Dwyer) Borhidi; CHIS, OAX, PUE, TAB, VER. |
| *Palicourea* *violacea* (Aubl.) A.Rich.; useful (MEDICINES). |
| ***Pittoniotis*** |
| *Pittoniotis protracta* (Bartl. ex DC.) Griseb. |
| *Pittoniotis trichantha* Griseb.; CHIS. |
| ***Pogonopus*** |
| *Pogonopus exsertus* (Oerst.) Oerst.; CHIS, VER; Red List: LC. |
| ***Posoqueria*** |
| *Posoqueria coriacea* M.Martens & Galeotti; CHIS, GRO, OAX, TAB, VER; Red List: LC. |
| *Posoqueria latifolia* (Rudge) Schult.; CHIS, GRO, OAX, TAB, VER; Red List: LC; useful (MATERIALS, FUELS). |
| ***Pseudomiltemia*** |
| *Pseudomiltemia davidsonii* Mart.-Camilo & Lorence; CHIS (endemic). |
| ***Psychotria*** |
| *Psychotria* *calophylla* Standl.; OAX, QROO, VER. |
| *Psychotria* *carthagenensis* Jacq.; CAM, CHIS, OAX, QROO, SLP, TAB, TAMS, VER. |
| *Psychotria* *clivorum* Standl. & Steyerm.; CHIS, OAX, TAB, VER. |
| *Psychotria* *diegoae* Borhidi; QRO, SLP (endemic). |
| *Psychotria* *dwyeri* C.W.Ham.; OAX, VER (endemic). |
| *Psychotria* *flava* Oerst. ex Standl.; CHIS, GRO, OAX, PUE, QRO, SLP, TAB, VER. |
| *Psychotria* *grandis* Sw.; CHIS, GRO, OAX, TAB, VER, YUC. |
| *Psychotria* *hidalgensis* Borhidi; HGO, PUE, QRO, SLP, TAMS (endemic). |
| *Psychotria* *limonensis* K.Krause; CAM, CHIS, JAL, MICH, NAY, OAX, PUE, QRO, SLP, TAB, VER. |
| *Psychotria* *lorenciana* C.M.Taylor; CHIS. |
| *Psychotria* *lundellii* Standl.; CHIS, VER. |
| *Psychotria* *mexiae* Standl.; CHIS, GRO, JAL, NAY, OAX, PUE, SON, VER. |
| *Psychotria* *micrantha* Kunth; CAM, CHIS, VER. |
| *Psychotria* *nervosa* Sw.; CAM, CHIS, GTO, OAX, QRO, QROO, SLP, TAB, VER, YUC. |
| *Psychotria* *oaxacensis* Borhidi & Salas-Mor.; OAX (endemic). |
| *Psychotria* *officinalis* (Aubl.) Raeusch. ex Sandwith; CHIS, TAB, VER. |
| *Psychotria* *panamensis* Standl.; CHIS, GRO, OAX, PUE, TAB, VER. |
| *Psychotria* *papantlensis* (Oerst.) Hemsl.; CHIS, MICH, QRO, SLP, TAB, TAMS, VER, YUC (endemic). |
| *Psychotria* *pleuropoda* Donn.Sm.; CHIS, TAB. |
| *Psychotria* *sarapiquensis* Standl.; CHIS, OAX, VER (endemic). |
| *Psychotria* *trichotoma* M.Martens & Galeotti; CHIS, GRO, HGO, JAL, OAX, PUE, TAB, VER; Red List: LC. |
| *Psychotria* *viridis* Ruiz & Pav.; CHIS, TAB. |
| ***Randia*** |
| *Randia* *aciculiflora* Borhidi & Saynes; OAX (endemic). |
| *Randia* *aculeata* L.; CAM, CHIS, COL, DGO, GRO, HGO, JAL, MEX, MICH, MOR, NAY, NLE, OAX, PUE, QRO, QROO, SLP, SIN, TAB, TAMS, VER, YUC; useful (MEDICINES, FUELS). |
| *Randia* *alvarocamposii* Borhidi & E.Martínez; OAX (endemic). |
| *Randia* *armata* (Sw.) DC.; BCN, BCS, CAM, CHIS, COL, DGO, GRO, JAL, MICH, NAY, OAX, PUE, QROO, SIN, TAB, TAMS, VER, YUC; useful (ANIMAL FOOD). |
| *Randia* *brachysiphon* Borhidi & Salas-Mor.; OAX (endemic). |
| *Randia* *canescens* Greenm.; COL, GTO, GRO, MICH, MOR, OAX, QRO (endemic). |
| *Randia* *capitata* DC.; BCS, CAM, CHIS, CHIH, COL, DGO, GTO, GRO, HGO, JAL, MEX, MICH, MOR, NAY, NLE, OAX, PUE, QRO, SLP, SIN, SON, TAB, TAMS, VER, ZAC; banked; useful (MEDICINES). |
| *Randia* *coronata* Borhidi; GRO (endemic). |
| *Randia* *crucis* Borhidi & Salas-Mor.; OAX (endemic). |
| *Randia* *dionisi* Borhidi & Salas-Mor.; OAX (endemic). |
| *Randia* *grandifolia* (Donn.Sm.) Standl.; CHIS, VER; Red List: LC. |
| *Randia* *hypoleuca* Borhidi & E.Martínez; MICH, OAX (endemic). |
| *Randia* *keniae* Borhidi & Salas-Mor.; (endemic). |
| *Randia* *laevigatoides* Borhidi; GRO, MICH, OAX (endemic). |
| *Randia* *longiloba* Hemsl.; CAM, CHIS, QROO, YUC; useful (MEDICINES). |
| *Randia* *lorenceana* J.Jiménez Ram. & Cruz Durán; GRO, OAX (endemic). |
| *Randia* *matudae* Lorence & Dwyer; CHIS, OAX, VER. |
| *Randia* *mayana* Lundell. |
| *Randia* *mendozae* Govaerts ex Borhidi; GRO, MICH (endemic). |
| *Randia* *mixe* Borhidi & E.Martínez; OAX (endemic). |
| *Randia* *nodifolia* Borhidi & García Gonz.; OAX. |
| *Randia* *pascualii* Borhidi & Salas-Mor.; OAX (endemic). |
| *Randia* *petenensis* Lundell; CHIS, OAX, VER; useful (MEDICINES). |
| *Randia* *pringlei* A.Gray; CHIH, COAH, DGO, HGO, NLE, QRO, SON, TAMS, ZAC (endemic). |
| *Randia* *pterocarpa* Lorence & Dwyer; OAX, VER (endemic). |
| *Randia* *sepium* Borhidi & E.Martínez; CHIS (endemic). |
| *Randia* *serboi* Borhidi & Saynes; OAX (endemic). |
| *Randia* *similis* Borhidi & Salas-Mor; OAX (endemic). |
| *Randia* *tubericollis* Borhidi, E.Martínez & A.Nava; JAL, OAX (endemic). |
| *Randia* *veracruzana* Borhidi & E.Martínez; VER (endemic). |
| ***Rogiera*** |
| *Rogiera amoena* Planch.; CHIS, COL, GTO, GRO, JAL, MICH, OAX, SIN, VER,. |
| *Rogiera breedlovei* (Lorence) Borhidi; CHIS (endemic). |
| *Rogiera cordata* (Benth.) Planch.; CHIS, GRO, JAL, OAX, VER. |
| *Rogiera edwardsii* (Standl.) Borhidi; CHIS, OAX. |
| ***Ronabea*** |
| *Ronabea latifolia* Aubl.; CHIS, VER; Red List: LC. |
| ***Rondeletia*** |
| *Rondeletia belizensis* Standl.; CHIS. |
| *Rondeletia chinajensis* Standl. & Steyerm. |
| ***Rudgea*** |
| *Rudgea cornifolia* (Kunth) Standl.; CHIS, GRO, NAY, OAX, TAB, VER, YUC; Red List: LC. |
| ***Simira*** |
| *Simira mexicana* (Bullock) Steyerm.; CHIS, CDMX, GRO, MEX, JAL, MICH, MOR (endemic); useful (MEDICINES). |
| *Simira rhodoclada* (Standl.) Steyerm.; CHIS, MOR, OAX, PUE, TAB, TAMS, VER. |
| *Simira salvadorensis* (Standl.) Steyerm.; CAM, CHIS, GRO, JAL, MICH, OAX, QROO, TAB, VER, YUC; banked. |
| ***Solenandra*** |
| *Solenandra mexicana* (A.Gray) Borhidi; CAM, CHIS, COL, GRO, HGO, JAL, MICH, OAX, QRO, QROO, SLP, TAB, TAMS, VER, YUC. |
| ***Sommera*** |
| *Sommera* *arborescens* Schltdl.; CHIS, COL, OAX, PUE, VER (endemic); banked. |
| *Sommera* *chiapensis* Brandegee; CHIS, PUE, VER. |
| *Sommera* *fusca* Oerst. ex Standl.; OAX (endemic). |
| *Sommera* *grandis* (Bartl. ex DC.) Standl.; CHIS, COL, DGO, GRO, JAL, MICH, NAY, OAX, SIN, VER. |
| *Sommera* *guatemalensis* Standl.; CHIS, OAX, VER. |
| *Sommera* *parva* Lorence; CHIS (endemic). |
| ***Stenostomum*** |
| *Stenostomum aromaticum* (Cast.-Campos & Lorence) Borhidi; OAX, VER (endemic); Red List: EN. |
| *Stenostomum lucidum* (Sw.) C.F.Gaertn.; CAM, CHIS, QROO, TAB, VER, YUC; Red List: LC. |
| ***Tocoyena*** |
| *Tocoyena pittieri* (Standl.) Standl.; Red List: VU. |
| ***Warszewiczia*** |
| *Warszewiczia uxpanapensis* (Lorence) C.M.Taylor; CHIS, OAX, TAB, VER (endemic). |
|  |
| **Rutaceae** |
| ***Amyris*** |
| *Amyris* *attenuata* Standl.; CHIS, QROO, VER. |
| *Amyris* *chiapensis* Lundell; CHIS (endemic). |
| *Amyris* *elemifera* L.; CAM, CHIS, COL, GRO, JAL, MICH, OAX, PUE, QROO, SLP, TAB, TAMS, VER, YUC. |
| *Amyris* *jorgemeavei* Hern.-Barón, Espejo, Pérez-García, Cerros & López-Ferr.; (endemic). |
| *Amyris* *madrensis* S.Watson; COAH, COL, GTO, JAL, NLE, SLP, TAMS, VER, ZAC. |
| *Amyris* *marshii* Standl.; COAH, NLE (endemic). |
| *Amyris mexicana* Lundell; COL, JAL, MICH (endemic). |
| *Amyris* *purpusii* P.Wilson; VER (endemic). |
| *Amyris* *rekoi* S.F.Blake; CHIS, COL, GRO, HGO, JAL, MEX, MOR, NAY, OAX, PUE, QRO, SLP, VER (endemic). |
| ***Angostura*** |
| *Angostura granulosa* (Kallunki) Kallunki; NAY; Red List: LC. |
| ***Casimiroa*** |
| *Casimiroa edulis* La Llave; AGS, BCS, CHIS, CHIH, COL, CDMX, DGO, GTO, GRO, HGO, JAL, MEX, MICH, MOR, NAY, OAX, PUE, QRO, SLP, SIN, SON, TAB, TAMS, TLAX, VER, ZAC; banked; useful (FOOD, MEDICINES). |
| *Casimiroa greggii* (S.Watson) F.Chiang; COAH, HGO, NLE, QRO, SLP, TAMS, VER (endemic). |
| *Casimiroa microcarpa* Lundell; CHIS. |
| *Casimiroa pringlei* (S.Watson) Engl.; COAH, DGO, NLE, SLP, TAMS (endemic); banked. |
| ***Decatropis*** |
| *Decatropis paucijuga* (Donn.Sm.) Loes.; CHIS; Red List: EN. |
| ***Decazyx*** |
| *Decazyx esparzae* F.Chiang; CHIS, OAX, TAB, VER (endemic); Red List: CR. |
| ***Esenbeckia*** |
| *Esenbeckia* *berlandieri* Baill.; CAM, CHIS, CHIH, COAH, COL, GTO, GRO, HGO, JAL, MICH, NAY, NLE, OAX, QRO, QROO, SLP, SIN, TAMS, VER, YUC, ZAC; useful (ENVIRONMENTAL USES). |
| *Esenbeckia* *bicolor* Ramos; CHIS (endemic). |
| *Esenbeckia* *collina* subsp. conspecta Kaastra; COL, JAL, MICH (endemic). |
| *Esenbeckia* *dorantesii* Ramos & E.Martínez; VER (endemic). |
| *Esenbeckia* *feddemae* Kaastra; COL, JAL, MICH. |
| *Esenbeckia* *flava* Brandegee; BCS (endemic); banked; useful. |
| *Esenbeckia* *hartmanii* B.L.Rob. & Fernald; CHIH, SIN, SON (endemic); banked. |
| *Esenbeckia* *macrantha* Rose; OAX, PUE, SLP, VER (endemic); banked; useful (MATERIALS). |
| *Esenbeckia* *nesiotica* Standl.; COL, JAL, MICH, NAY (endemic); useful (ENVIRONMENTAL USES). |
| *Esenbeckia* *pentaphylla* (Macfad.) Griseb. |
| *Esenbeckia* *stephani* Ramos; OAX (endemic). |
| *Esenbeckia* *vazquezii* Ramos & E.Martínez; MOR (endemic). |
| *Esenbeckia* *velutina* Ramos; GRO (endemic). |
| ***Helietta*** |
| *Helietta lottiae* F.Chiang; COL, GRO, JAL, OAX (endemic). |
| *Helietta lucida* Brandegee; OAX, PUE (endemic); banked. |
| *Helietta parvifolia* Benth.; COAH, GTO, HGO, NLE, PUE, QRO, SLP, TAMS, VER. |
| ***Megastigma*** |
| *Megastigma balsense* F.Chiang & J.Jiménez Ram.; GRO, OAX (endemic). |
| *Megastigma chiangii* J.Jiménez Ram. & Cruz Durán; GRO (endemic). |
| ***Peltostigma*** |
| *Peltostigma guatemalense* (Standl. & Steyerm.) Gereau; CHIS, TAB, VER. |
| *Peltostigma pteleoides* (Hook.) Walp.; CHIS, DGO, HGO, JAL, MICH, SLP, SIN, VER. |
| ***Pilocarpus*** |
| *Pilocarpus goudotianus* Tul. |
| ***Polyaster*** |
| *Polyaster boronioides* Benth. & Hook.f.; GTO, HGO, OAX, PUE, QRO, TAMS, VER (endemic). |
| ***Ptelea*** |
| *Ptelea trifoliata* L.; AGS, CHIH, COAH, COL, DGO, GTO, GRO, HGO, JAL, MEX, MICH, MOR, NLE, OAX, PUE, QRO, SLP, SON, TAMS, VER, ZAC; banked; useful (SOCIAL USES). |
| ***Stauranthus*** |
| *Stauranthus conzattii* Rose & Standl.; OAX (endemic). |
| *Stauranthus perforatus* Liebm.; CHIS, OAX, VER. |
| ***Zanthoxylum*** |
| *Zanthoxylum* *acuminatum* (Sw.) Sw.; CAM, CHIS, OAX, QROO, QRO, SLP, TAB, TAMS, VER; Red List: LC. |
| *Zanthoxylum* *arborescens* Rose; BCS, CHIS, COL, DGO, HGO, JAL, MICH, MOR, NAY, OAX, PUE, SIN, SON, VER (endemic); banked; useful. |
| *Zanthoxylum* *caribaeum* Lam.; CAM, CHIS, COL, DGO, GRO, JAL, MOR, NAY, OAX, QRO, QROO, SLP, SIN, TAB, TAMS, VER, YUC; useful (MEDICINES, MATERIALS). |
| *Zanthoxylum* *clava-herculis* L.; COAH, HGO, QRO, SLP, TAMS, VER; banked. |
| *Zanthoxylum* *ekmanii* (Urb.) Alain; CHIS, OAX, TAB, VER. |
| *Zanthoxylum* *fagara* (L.) Sarg.; AGS, BCS, CAM, CHIS, CHIH, COAH, COL, DGO, GTO, GRO, HGO, JAL, MEX, MICH, MOR, NAY, NLE, OAX, PUE, QRO, QROO, SLP, SIN, SON, TAB, TAMS, VER, YUC, ZAC; banked; useful (MEDICINES). |
| *Zanthoxylum* *limoncello* Planch. & Oerst.; CHIS, GRO, JAL, MEX, MICH, MOR, OAX, PUE, VER; Red List: LC; banked. |
| *Zanthoxylum* *melanostictum* Schltdl. & Cham.; CHIS, COL, GRO, JAL, OAX, PUE, TAB, VER; Red List: LC; banked. |
| *Zanthoxylum* *quassiifolium* (Donn.Sm.) Standl. & Steyerm.; CHIS, VER. |
| *Zanthoxylum* *rhoifolium* Lam.; CHIS, OAX, TAB; Red List: LC. |
| *Zanthoxylum* *riedelianum* Engl.; CHIS, OAX, PUE, QROO, TAB, VER, YUC; Red List: LC; banked; useful (MATERIALS). |
| *Zanthoxylum* *schreberi* (J.F.Gmel.) Reynel ex C.Nelson; CHIS, SIN, NAY. |
|  |
| **Sabiaceae** |
| ***Meliosma*** |
| *Meliosma* *alba* (Schltdl.) Walp.; COAH, HGO, NLE, PUE, QRO, SLP, TAMS, VER; banked. |
| *Meliosma* *dentata* (Liebm.) Urb.; CHIS, CHIH, COL, CDMX, DGO, GRO, HGO, JAL, MEX, MICH, MOR, NAY, OAX, QRO, SLP, SIN, TAMS, VER. |
| *Meliosma* *echeverriae* J.Menjívar, Cerén & J.F.Morales. |
| *Meliosma* *glabrata* (Liebm.) Urb.; OAX, VER; Red List: LC. |
| *Meliosma* *grandifolia* (Liebm.) Urb.; CHIS, OAX, VER; Red List: LC. |
| *Meliosma* *idiopoda* S.F.Blake; CHIS, COL, JAL, OAX, VER. |
| *Meliosma mexicana* V.W.Steinm.; QRO, SLP, TAMS (endemic). |
| *Meliosma* *nesites* I.M.Johnst.; COL, JAL (endemic). |
| *Meliosma* *seleriana* Urb.; CHIS. |
| *Meliosma* *starkii* E.Ramos; (endemic). |
|  |
| **Salicaceae** |
| ***Abatia*** |
| *Abatia mexicana* Standl.; GRO, HGO, OAX, PUE, SLP, VER (endemic). |
| ***Bartholomaea*** |
| *Bartholomaea sessiliflora* (Standl.) Standl. & Steyerm.; CHIS, OAX. |
| ***Casearia*** |
| *Casearia* *aculeata* Jacq.; CAM, CHIS, COL, GRO, HGO, JAL, MEX, MICH, NAY, OAX, PUE, QRO, QROO, SLP, SIN, TAB, TAMS, VER, YUC; Red List: LC; useful (FOOD, MEDICINES, MATERIALS). |
| *Casearia* *bartlettii* Lundell; CHIS, MICH, TAB. |
| *Casearia* *corymbosa* Kunth; VER; banked. |
| *Casearia* *sanchezii* J. Linares & D. Angulo F.; CHIS. |
| *Casearia* *sylvestris* Sw.; CAM, CHIS, COL, DGO, GRO, JAL, MICH, NAY, OAX, PUE, QRO, QROO, SLP, SIN, TAB, VER, YUC; Red List: LC. |
| *Casearia* *tacanensis* Lundell; CHIS, OAX, VER; banked. |
| *Casearia* *tremula* (Griseb.) Griseb. ex C.Wright; CAM, CHIS, COL, GRO, JAL, MEX, MICH, NAY, OAX, PUE, QROO, SIN. |
| ***Homalium*** |
| *Homalium racemosum* Jacq.; CHIS, GRO, JAL, MICH, OAX, VER; useful (MATERIALS). |
| ***Laetia*** |
| *Laetia thamnia* L.; CAM, CHIS, QROO, TAB, VER, YUC. |
| ***Lunania*** |
| *Lunania mexicana* Brandegee; CHIS, OAX, VER; useful (ENVIRONMENTAL USES). |
| ***Mayna*** |
| *Mayna suaveolens* (H.Karst. & Triana) Warb.; VER; Red List: EN. |
| ***Olmediella*** |
| *Olmediella betschleriana* (Göpp.) Loes.; CHIS, VER. |
| ***Pleuranthodendron*** |
| *Pleuranthodendron lindenii* (Turcz.) Sleumer; CHIS, HGO, OAX, PUE, QRO, SLP, TAB, TAMS, VER; useful (FUELS). |
| ***Populus*** |
| *Populus* × *acuminata* Rydb.; CHIH, SON. |
| *Populus* *alba* L.; Red List: LC; useful (ANIMAL FOOD, MEDICINES, MATERIALS, ENVIRONMENTAL USES). |
| *Populus* *angustifolia* E.James; CHIH, COAH, SON; Red List: LC; useful (ENVIRONMENTAL USES). |
| *Populus* *brandegeei* C.K.Schneid.; BCS, CHIH, SON (endemic). |
| *Populus* *deltoides* W.Bartram ex Marshall; AGS, CHIH, COAH, DGO, JAL, NLE, SON, VER, ZAC; Red List: LC. |
| *Populus* *fremontii* S.Watson; AGS, BCN, BCS, CHIS, CHIH, COAH, CDMX, DGO, GTO, HGO, JAL, MEX, NLE, PUE, QRO, SLP, SON, TAMS, ZAC; useful (MEDICINES). |
| *Populus* *guzmanantlensis* A.Vázquez & R.Cuevas; COL, JAL (endemic); Red List: EN; NOM-59: Pr. |
| *Populus mexicana* Wesm.; CHIS, COAH, DGO, HGO, JAL, NLE, OAX, PUE, QRO, SLP, SIN, SON, TAMS, VER, ZAC (endemic); useful (MATERIALS, ENVIRONMENTAL USES). |
| *Populus* *simaroa* Rzed.; GRO, JAL, MEX, MICH, OAX (endemic); NOM-59: Pr. |
| *Populus* *tremuloides* Michx.; AGS, BCN, CHIH, COAH, DGO, GTO, HGO, JAL, MEX, MICH, NLE, QRO, SLP, SIN, SON, TAMS, VER, ZAC; Red List: LC. |
| *Populus* *trichocarpa* Torr. & A.Gray ex Hook.; BCN; Red List: LC; useful (MATERIALS). |
| *Populus* *primaveralepensis* A. Vázquez, Muñiz-Castro & Zuno; (endemic). |
| ***Prockia*** |
| *Prockia crucis* P.Browne ex L.; CAM, CHIS, COL, GRO, JAL, MEX, MICH, MOR, NAY, OAX, PUE, QROO, SLP, SIN, TAMS, VER, YUC. |
| *Prockia krusei* J.Jiménez Ram. & Cruz Durán; GRO. |
| *Prockia oaxacana* J.Jiménez Ram. & Cruz Durán; OAX (endemic). |
| ***Salix*** |
| *Salix* × *wrightii* Andersson; CHIH, SON. |
| *Salix* *aeruginosa* E.Carranza; MEX, MICH (endemic). |
| *Salix* *bonplandiana* Kunth; AGS, BCN, BCS, CAM, CHIS, CHIH, COAH, COL, CDMX, DGO, GTO, GRO, HGO, JAL, MEX, MICH, MOR, NAY, NLE, OAX, PUE, QRO, SLP, SIN, SON, TAB, TLAX, VER, ZAC; Red List: LC; banked; useful (FOOD, MEDICINES, MATERIALS, ENVIRONMENTAL USES). |
| *Salix* *cana* M.Martens & Galeotti; COL, CDMX, HGO, JAL, MEX, MICH, MOR, OAX, PUE, TLAX, VER (endemic). |
| *Salix* *exigua* Nutt.; BCN, CHIH, COAH, DGO, SON, TAMS, VER; Red List: LC. |
| *Salix* *gooddingii* C.R.Ball; BCN, BCS, CHIH, COAH, COL, GRO, JAL, NLE, OAX, SIN, SON; Red List: LC. |
| *Salix* *hartwegii* Benth.; DGO, GRO, HGO, JAL, MEX, MICH, MOR, NAY (endemic). |
| *Salix* *humboldtiana* Willd.; AGS, CAM, CHIS, COAH, COL, CDMX, DGO, GTO, GRO, HGO, JAL, MEX, MICH, MOR, NAY, NLE, OAX, PUE, QRO, SLP, SON, TAB, TAMS, TLAX, VER, ZAC; Red List: LC; banked; useful (ANIMAL FOOD, MEDICINES, MATERIALS, FUELS, ENVIRONMENTAL USES). |
| *Salix* *jaliscana* M.E.Jones; CHIH, COL, DGO, JAL, MEX, MICH, NAY, NLE, OAX, SIN, SON, ZAC (endemic). |
| *Salix* *laevigata* Bebb; BCN, SON. |
| *Salix* *lasiolepis* Benth.; AGS, BCN, BCS, CHIH, COAH, DGO, GTO, HGO, JAL, MEX, MOR, NLE, QRO, SLP, SIN, SON, TAMS, VER, ZAC; Red List: LC. |
| *Salix mexicana* Seemen; GTO, HGO, MEX, PUE, TLAX, VER (endemic). |
| *Salix* *nigra* Marshall; CHIH, COAH, DGO, NAY, NLE, OAX, PUE, SLP, SIN, TAMS, ZAC; Red List: LC; useful (ANIMAL FOOD, MEDICINES, MATERIALS, ENVIRONMENTAL USES). |
| *Salix* *paradoxa* Kunth; COAH, COL, CDMX, DGO, GTO, GRO, HGO, JAL, MEX, MICH, MOR, NLE, OAX, PUE, QRO, SLP, TAMS, TLAX, VER, ZAC (endemic). |
| *Salix* *riskindii* M.C.Johnst.; COAH (endemic). |
| *Salix* *scouleriana* Barratt ex Hook.; COAH, SON; Red List: LC. |
| *Salix* *taxifolia* Kunth; AGS, BCN, BCS, CHIS, CHIH, COAH, COL, DGO, GTO, GRO, HGO, JAL, MEX, MICH, MOR, NAY, NLE, OAX, PUE, QRO, SLP, SIN, SON, TAMS, VER, ZAC; Red List: LC. |
| ***Samyda*** |
| *Samyda mexicana* Rose; COL, GRO, JAL, MICH, OAX (endemic). |
| *Samyda yucatanensis* Standl.; CAM, QROO, YUC (endemic). |
| ***Xylosma*** |
| *Xylosma* *characantha* Standl. |
| *Xylosma* *ciliatifolia* (Clos) Eichler. |
| *Xylosma* *flexuosa* (Kunth) Hemsl.; CAM, CHIS, COAH, COL, GTO, GRO, HGO, JAL, MEX, MICH, MOR, NAY, NLE, OAX, PUE, QRO, QROO, SLP, SIN, SON, TAB, TAMS, VER, YUC; Red List: LC; banked. |
| *Xylosma* *intermedia* (Seem.) Triana & Planch.; CHIS, COL, GTO, GRO, JAL, MEX, MICH, OAX, SIN, VER. |
| *Xylosma* *oligandra* Donn.Sm.; CHIS, OAX, VER; Red List: LC. |
| *Xylosma* *panamensis* Turcz.; CHIS, COL, GRO, MICH, NAY, OAX, PUE, QROO, TAB, VER. |
| *Xylosma* *quichensis* Donn.Sm.; CHIS, OAX, PUE, VER. |
| ***Zuelania*** |
| *Zuelania guidonia* (Sw.) Britton & Millsp.; CAM, CHIS, HGO, OAX, PUE, QRO, QROO, SLP, TAB, TAMS, VER, YUC; useful (MATERIALS, SOCIAL USES). |
|  |
| **Sapindaceae** |
| ***Acer*** |
| *Acer binzayedii* Y.L.Vargas-Rodr.; (endemic); Red List: CR. |
| *Acer negundo* L.; AGS, CHIS, CHIH, COAH, COL, CDMX, DGO, GRO, HGO, JAL, MEX, MICH, NLE, OAX, PUE, QRO, SLP, SON, TAMS, TLAX, VER; Red List: LC; NOM-59: Pr; useful (MATERIALS, ENVIRONMENTAL USES). |
| *Acer saccharum* var. *sinuosum* (Rehder) Sarg.; CHIH, SON. |
| ***Aesculus*** |
| *Aesculus parryi* A.Gray; BCN (endemic); banked. |
| ***Allophylus*** |
| *Allophylus* *camptostachys* Radlk.; CAM, CHIS, JAL, NAY, OAX, QROO, TAB, VER, YUC, ZAC; Red List: LC. |
| *Allophylus* *cominia* (L.) Sw.; CAM, CHIS, GRO, MEX, MICH, OAX, QROO, TAB, VER, YUC; useful (ANIMAL FOOD, MEDICINES, MATERIALS, ENVIRONMENTAL USES). |
| *Allophylus* *psilospermus* Radlk.; CAM, CHIS, OAX, QROO, VER, YUC. |
| *Allophylus* *racemosus* Sw.; CHIS, TAB, VER. |
| ***Averrhoidium*** |
| *Averrhoidium spondioides* (Standl.) Acev.-Rodr. & Ferrucci; COL, GRO, JAL, NAY (endemic). |
| ***Billia*** |
| *Billia hippocastanum* Peyr.; CHIS, GRO, OAX, PUE, VER; banked. |
| ***Cupania*** |
| *Cupania* *dentata* Moc. & Sessé ex DC.; CAM, CHIS, COL, GRO, HGO, JAL, MICH, NAY, OAX, PUE, QRO, QROO, SLP, SIN, TAB, TAMS, VER, YUC; banked; useful (ANIMAL FOOD, MEDICINES, MATERIALS, FUELS). |
| *Cupania* *glabra* Sw.; CAM, CHIS, COL, GRO, HGO, JAL, MEX, MICH, NAY, OAX, PUE, QROO, SLP, SIN, TAB, TAMS, VER, YUC; Red List: LC; banked; useful (MATERIALS, FUELS). |
| *Cupania* *juglandifolia* A.Rich.; CHIS, VER; Red List: LC. |
| *Cupania* *mayana* Lundell; CHIS, TAB, VER. |
| *Cupania* *mollis* Standl.; CHIS, OAX, VER; Red List: LC. |
| *Cupania* *rufescens* Triana & Planch.; CHIS, PUE, VER; Red List: LC. |
| *Cupania* *spectabilis* Radlk.; CHIS, TAB, VER. |
| ***Dodonaea*** |
| *Dodonaea viscosa* Jacq.; AGS, BCN, BCS, CHIS, CHIH, COAH, COL, CDMX, DGO, GTO, GRO, HGO, JAL, MEX, MICH, MOR, NAY, NLE, OAX, PUE, QRO, QROO, SLP, SIN, SON, TAMS, TLAX, VER, YUC, ZAC; Red List: LC; banked; useful (ANIMAL FOOD, MEDICINES, POISONS, MATERIALS, FUELS, ENVIRONMENTAL USES, SOCIAL USES). |
| ***Exothea*** |
| *Exothea diphylla* (Standl.) Lundell; CAM, QROO, YUC; Red List: LC. |
| *Exothea paniculata* (Juss.) Radlk.; CAM, CHIS, COL, GTO, HGO, JAL, MICH, OAX, QRO, SLP, QROO, TAMS, VER. |
| ***Matayba*** |
| *Matayba* *clavelligera* Radlk.; CHIS, OAX, VER; Red List: LC. |
| *Matayba* *floribunda* Radlk.; OAX (endemic). |
| *Matayba* *glaberrima* Radlk.; CHIS, VER. |
| *Matayba mexicana* (Turcz.) Radlk.; VER. |
| *Matayba* *scrobiculata* Radlk.; CHIS, COL, GRO, JAL, OAX, VER. |
| *Matayba* *sylvatica* (Casar.) Radlk.; CHIS. |
| ***Melicoccus*** |
| *Melicoccus oliviformis* Kunth; useful (FOOD). |
| ***Sapindus*** |
| *Sapindus saponaria* L.; BCS, CAM, CHIS, CHIH, COAH, COL, DGO, GTO, GRO, HGO, JAL, MEX, MICH, MOR, NAY, NLE, OAX, PUE, QRO, QROO, SLP, SIN, SON, TAB, TAMS, VER, YUC; banked; useful (MEDICINES, POISONS, MATERIALS, ENVIRONMENTAL USES, SOCIAL USES). |
| ***Thouinia*** |
| *Thouinia paucidentata* Radlk. ex Millsp.; CAM, CHIS, COL, GRO, JAL, MEX, MICH, NAY, OAX, QROO, YUC; Red List: LC; banked. |
| *Thouinia serrata* Radlk.; COL, JAL, NAY, OAX (endemic). |
| *Thouinia villosa* DC.; CAM, CHIH, COL, DGO, GRO, HGO, JAL, MEX, MICH, MOR, NLE, OAX, PUE, QRO, QROO, SLP, SIN, SON, TAMS, ZAC (endemic); banked; useful. |
| ***Thouinidium*** |
| *Thouinidium decandrum* (Bonpl.) Radlk.; CAM, CHIS, COL, DGO, GRO, JAL, MEX, MICH, MOR, NAY, OAX, SIN, TAB, VER, ZAC; banked. |
| *Thouinidium insigne* (Brandegee) Radlk.; OAX, PUE (endemic); banked; useful. |
| *Thouinidium oblongum* Radlk.; HGO, PUE, VER (endemic). |
|  |
| **Sapotaceae** |
| ***Chrysophyllum*** |
| *Chrysophyllum* *cainito* L.; useful (FOOD). |
| *Chrysophyllum* *mexicanum* Brandegee; CAM, CHIS, GRO, HGO, OAX, PUE, QRO, QROO, SLP, TAB, TAMS, VER, YUC. |
| *Chrysophyllum* *oliviforme* L.; CHIS, SLP; useful (FOOD, ENVIRONMENTAL USES). |
| *Chrysophyllum* *venezuelanense* (Pierre) T.D.Penn.; CAM, CHIS, OAX, PUE, QROO, TAB, VER; Red List: LC; useful (FOOD, MATERIALS). |
| ***Manilkara*** |
| *Manilkara chicle* (Pittier) Gilly; CAM, CHIS, OAX, PUE, QROO, TAB, VER. |
| *Manilkara zapota* (L.) P.Royen; CAM, CHIS, CHIH, COL, DGO, GRO, HGO, JAL, MEX, MICH, MOR, NAY, OAX, PUE, QRO, QROO, SLP, SIN, SON, TAB, TAMS, VER, YUC, ZAC; banked; useful (FOOD, MATERIALS, FUELS, ENVIRONMENTAL USES). |
| ***Micropholis*** |
| *Micropholis melinoniana* Pierre; CHIS, GRO, OAX, TAB, VER; Red List: LC. |
| ***Pouteria*** |
| *Pouteria* *amygdalina* (Standl.) Baehni; CAM, CHIS, QROO; Red List: VU. |
| *Pouteria* *belizensis* (Standl.) Cronquist; CHIS, TAB, VER; Red List: VU. |
| *Pouteria* *briocheoides* Lundell; CHIS; Red List: VU. |
| *Pouteria* *campechiana* (Kunth) Baehni; CAM, CHIS, COL, GRO, HGO, JAL, MEX, MICH, MOR, NAY, OAX, PUE, QRO, QROO, SLP, SIN, TAB, VER, YUC; Red List: LC; useful (FOOD, MATERIALS). |
| *Pouteria* *durlandii* (Standl.) Baehni; CAM, CHIS, OAX, PUE, QROO, SLP, TAB, VER; Red List: LC; useful (FOOD, ANIMAL FOOD, MATERIALS). |
| *Pouteria* *izabalensis* (Standl.) Baehni; CHIS; Red List: NT; useful (FOOD, ANIMAL FOOD, MEDICINES, MATERIALS, ENVIRONMENTAL USES). |
| *Pouteria* *reticulata* (Engl.) Eyma; CAM, CHIS, OAX, QROO, TAB, VER, YUC; useful (MATERIALS). |
| *Pouteria* *rhynchocarpa* T.D.Penn.; OAX, VER (endemic); Red List: EN. |
| *Pouteria* *sapota* (Jacq.) H.E.Moore & Stearn; CAM, CHIS, COL, GTO, GRO, JAL, MEX, MICH, MOR, OAX, PUE, QRO, QROO, SLP, SIN, TAB, VER, YUC; useful (FOOD, MATERIALS, ENVIRONMENTAL USES). |
| *Pouteria* *squamosa* Cronquist; OAX, VER; Red List: VU. |
| *Pouteria* *torta* (Mart.) Radlk.; CHIS, OAX, TAB, VER; useful (FOOD, MATERIALS). |
| *Pouteria* *viridis* (Pittier) Cronquist; CHIS, VER; Red List: LC. |
| ***Sideroxylon*** |
| *Sideroxylon* *americanum* (Mill.) T.D.Penn.; CAM, GRO, MOR, OAX, QROO, VER, YUC. |
| *Sideroxylon* *capiri* (A.DC.) Pittier; CHIS, CHIH, COL, DGO, GRO, JAL, MEX, MICH, MOR, NAY, OAX, PUE, QRO, QROO, SLP, SIN, SON, TAB, VER, ZAC; NOM-59: A; banked; useful (FOOD, MATERIALS, FUELS, ENVIRONMENTAL USES, SOCIAL USES). |
| *Sideroxylon* *cartilagineum* (Cronquist) T.D.Penn.; BCS, COL, GRO, JAL, MEX, MICH, MOR, NAY, OAX, SIN, ZAC (endemic); Red List: LR/nt; NOM-59: P. |
| *Sideroxylon* *celastrinum* (Kunth) T.D.Penn.; CAM, CHIS, CHIH, COAH, COL, DGO, GRO, HGO, JAL, MICH, NLE, OAX, QRO, QROO, SLP, SIN, TAB, TAMS, VER, YUC. |
| *Sideroxylon* *contrerasii* (Lundell) T.D.Penn.; CHIS, HGO, OAX, PUE, QRO, VER; Red List: LR/nt. |
| *Sideroxylon* *durifolium* (Standl.) T.D.Penn.; CHIS; Red List: VU. |
| *Sideroxylon* *eriocarpum* (Greenm. & Conz.) T.D.Penn.; GRO, MOR, OAX (endemic). |
| *Sideroxylon* *eucoriaceum* (Lundell) T.D.Penn.; CHIS, VER; Red List: VU. |
| *Sideroxylon* *excavatum* T.D.Penn.; GRO, OAX (endemic); Red List: EN. |
| *Sideroxylon* *floribundum* Griseb. |
| *Sideroxylon* *lanuginosum* Michx.; AGS, CHIH, COAH, JAL, NLE, SLP, SIN, SON, TAMS, VER. |
| *Sideroxylon* *leucophyllum* S.Watson; BCN, BCS, SON. |
| *Sideroxylon* *obtusifolium* (Roem. & Schult.) T.D.Penn.; CAM, CHIS, COL, GRO, JAL, MICH, OAX, PUE, QROO, TAB, VER, YUC; banked. |
| *Sideroxylon* *occidentale* (Hemsl.) T.D.Penn.; BCN, BCS, CHIH, JAL, MICH, SIN, SON, VER (endemic). |
| *Sideroxylon* *palmeri* (Rose) T.D.Penn.; CHIS, DGO, GTO, HGO, JAL, MEX, MICH, NAY, OAX, PUE, QRO, QROO, SLP, SIN, TAB, TAMS, VER (endemic); banked; useful (FOOD). |
| *Sideroxylon* *peninsulare* (Brandegee) T.D.Penn.; BCS, JAL, MICH, SIN (endemic); Red List: VU. |
| *Sideroxylon* *persimile* (Hemsl.) T.D.Penn.; CAM, CHIS, CHIH, COL, DGO, GRO, HGO, JAL, MICH, MOR, NAY, OAX, PUE, QROO, SIN, SON, TAB, VER, ZAC. |
| *Sideroxylon* *portoricense* Urb.; CHIS, COL, GRO, HGO, JAL, MEX, MOR, OAX, SLP, TAB, VER; useful (MATERIALS). |
| *Sideroxylon* *salicifolium* (L.) Lam.; CAM, CHIS, COL, GRO, JAL, OAX, PUE, QROO, TAMS, VER, YUC. |
| *Sideroxylon* *socorrense* (Brandegee) T.D.Penn.; COL, NAY, SIN (endemic); Red List: VU. |
| *Sideroxylon* *stenospermum* (Standl.) T.D.Penn.; CHIS, COL, GRO, JAL, MICH, NAY, OAX, SIN, VER. |
| *Sideroxylon* *stevensonii* (Standl.) Standl. & Steyerm.; CHIS. |
| *Sideroxylon* *tepicense* (Standl.) T.D.Penn.; CHIS, CHIH, COL, GRO, MEX, NAY, OAX, SLP, SIN, SON. |
|  |
| ***Schoepfia*ceae** |
| ***Schoepfia*** |
| *Schoepfia* *californica* Brandegee; BCN, BCS, OAX (endemic). |
| *Schoepfia* *flexuosa* Schult.; CAM, CHIS, CHIH, COL, GTO, GRO, HGO, JAL, MEX, MICH, NAY, NLE, OAX, PUE, QRO, QROO, SLP, SIN, SON, TAB, TAMS, VER, YUC, ZAC. |
| *Schoepfia* *pringlei* B.L.Rob.; GRO, MEX, MICH (endemic). |
| *Schoepfia* *schreberi* J.F.Gmel.; useful (MEDICINES). |
| *Schoepfia* *shreveana* Wiggins; SON (endemic). |
|  |
| **Scrophulariaceae** |
| ***Buddleja*** |
| *Buddleja* *americana* L.; CAM, CHIS, CDMX, GTO, GRO, HGO, JAL, MEX, MICH, MOR, NAY, OAX, PUE, QRO, QROO, SLP, TAMS, VER; useful (MEDICINES). |
| *Buddleja* *cordata* Kunth; AGS, CHIS, CHIH, COAH, COL, CDMX, DGO, GTO, GRO, HGO, JAL, MEX, MICH, MOR, NAY, NLE, OAX, PUE, QRO, SLP, SIN, SON, TAMS, TLAX, VER, ZAC; banked; useful (MEDICINES). |
| *Buddleja* *crotonoides* A.Gray; BCS, CHIS, CHIH, GRO, JAL, MICH, MOR, OAX, PUE, VER. |
| *Buddleja* *parviflora* Kunth; AGS, CHIS, CHIH, COAH, COL, CDMX, DGO, GTO, GRO, HGO, JAL, MEX, MICH, MOR, NAY, NLE, OAX, PUE, QRO, SLP, SIN, SON, TAMS, TLAX, VER, ZAC (endemic); Red List: LC; banked; useful (ANIMAL FOOD, SOCIAL USES). |
| *Buddleja* *sessiliflora* Kunth; AGS, BCN, CHIH, COAH, COL, CDMX, DGO, GTO, GRO, HGO, JAL, MEX, MICH, MOR, NAY, NLE, OAX, PUE, QRO, SLP, SIN, SON, TAMS, TLAX, VER, ZAC; banked; useful (ANIMAL FOOD, MEDICINES). |
|  |
| **Simaroubaceae** |
| ***Picrasma*** |
| *Picrasma mexicana* Brandegee; CHIS, COL, JAL, NAY, VER, ZAC (endemic). |
| ***Recchia*** |
| *Recchia* *connaroides* (Loes. & Soler.) Standl.; OAX (endemic). |
| *Recchia mexicana* Moc. & Sessé ex DC.; CHIS, COL, GRO, JAL, MICH, NAY, OAX, TAB (endemic); banked. |
| *Recchia simplicifolia* T.Wendt & E.J.Lott; CHIS, OAX, TAB, VER (endemic); Red List: EN. |
| ***Simarouba*** |
| *Simarouba amara* Aubl.; CAM, CHIS, COL, GRO, JAL, MICH, MOR, OAX, PUE, QROO, TAB, VER, YUC; Red List: LC. |
|  |
| **Siparunaceae** |
| ***Siparuna*** |
| *Siparuna grandiflora* (Kunth) Perkins; CHIS, OAX, VER. |
|  |
| **Solanaceae** |
| ***Cestrum*** |
| *Cestrum dumetorum* Schltdl.; CHIS, COL, DGO, GRO, HGO, JAL, MOR, NLE, OAX, PUE, QRO, SLP, SIN, TAMS, VER, ZAC; banked; useful (MEDICINES, ENVIRONMENTAL USES, SOCIAL USES). |
| *Cestrum nocturnum* L.; CAM, CHIS, COAH, COL, GRO, HGO, JAL, MEX, MICH, MOR, NAY, OAX, PUE, QRO, QROO, SLP, SIN, TAB, TAMS, VER, YUC, ZAC; Red List: LC; banked; useful (MEDICINES, POISONS, ENVIRONMENTAL USES). |
| *Cestrum tomentosum* L.f.; AGS, CHIS, CHIH, COL, DGO, GTO, GRO, HGO, JAL, MEX, MICH, MOR, NAY, OAX, PUE, SIN, SON, VER, ZAC; Red List: LC; banked; useful (ANIMAL FOOD, MEDICINES, MATERIALS). |
| ***Solanum*** |
| *Solanum umbellatum* Mill.; BCN, CAM, CHIS, CHIH, COL, DGO, GTO, GRO, HGO, JAL, MEX, MICH, MOR, NAY, NLE, OAX, PUE, QRO, QROO, SLP, SIN, SON, TAB, TAMS, VER, YUC, ZAC. |
|  |
| **Staphyleaceae** |
| ***Staphylea*** |
| *Staphylea pringlei* S.Watson; COAH, HGO, NLE, SLP, TAMS, VER (endemic). |
| ***Turpinia*** |
| *Turpinia insignis* (Kunth) Tul.; CHIS, GRO, HGO, OAX, PUE, VER. |
| *Turpinia occidentalis* (Sw.) G.Don; CHIS, COL, DGO, GRO, HGO, JAL, NAY, OAX, PUE, QRO, SLP, TAB, TAMS, VER; banked. |
| *Turpinia parvifoliola* L.O.Williams; CHIS. |
| *Turpinia tricornuta* Lundell; CHIS, GRO. |
|  |
| **Styracaceae** |
| ***Styrax*** |
| *Styrax* *argenteus* C.Presl; BCS, CHIS, COL, DGO, GRO, JAL, MEX, MICH, NAY, OAX, QRO, SLP, SIN, TAMS, VER; Red List: LC; useful (MATERIALS). |
| *Styrax* *austromexicanus* P.W.Fritsch; GRO, OAX (endemic). |
| *Styrax* *conterminus* Donn.Sm.; CHIS, GRO, OAX. |
| *Styrax* *gentryi* P.W.Fritsch; BCS, SIN (endemic). |
| *Styrax* *glabrescens* Benth.; CHIS, GTO, GRO, HGO, MEX, MICH, OAX, PUE, QRO, SLP, TAMS, VER; banked. |
| *Styrax* *lanceolatus* P.W.Fritsch; HGO, QRO, SLP, TAMS (endemic). |
| *Styrax* *platanifolius* Engelm. ex Torr.; COAH, NLE, TAMS. |
| *Styrax* *radians* P.W.Fritsch; CHIS, COL, DGO, GRO, JAL, MEX, MICH, NAY, OAX, SIN, VER (endemic). |
| *Styrax* *tuxtlensis* P.W.Fritsch; VER (endemic). |
| *Styrax* *uxpanapensis* P.W.Fritsch; OAX, VER (endemic). |
|  |
| **Surianaceae** |
| ***Recchia*** |
| *Recchia sessiliflora* Gonz.-Murillo & Cruz Durán; GRO (endemic). |
|  |
| **Symplocaceae** |
| ***Symplocos*** |
| *Symplocos* *austromexicana* Almeda; OAX (endemic). |
| *Symplocos* *breedlovei* Lundell; CHIS, OAX; Red List: EN. |
| *Symplocos* *citrea* Lex.; AGS, COL, CDMX, GTO, GRO, HGO, JAL, MEX, MICH, MOR, NAY, OAX, QRO, SLP, VER, ZAC (endemic). |
| *Symplocos* *coccinea* Bonpl.; CHIS, HGO, OAX, PUE, VER (endemic); Red List: VU. |
| *Symplocos* *excelsa* L.O.Williams; CHIS, OAX, VER (endemic); NOM-59: Pr. |
| *Symplocos* *hartwegii* A.DC.; CHIS, PUE, VER. |
| *Symplocos* *hintonii* Lundell; GRO (endemic). |
| *Symplocos* *jurgensenii* Hemsl.; OAX (endemic). |
| *Symplocos* *pachycarpa* L.M.Kelly & Almeda; GRO, OAX (endemic). |
| *Symplocos* *pycnantha* Hemsl.; CHIS, GRO, HGO, OAX, VER. |
| *Symplocos* *schiedeana* Schltdl.; CHIS, OAX, VER. |
| *Symplocos* *sousae* Almeda; COL, GRO, JAL, OAX (endemic); Red List: DD; NOM-59: Pr. |
| *Symplocos* *tacanensis* Lundell; CHIS; Red List: VU. |
|  |
| **Tapisciaceae** |
| ***Huertea*** |
| *Huertea cubensis* Griseb.; CHIS, PUE, VER; Red List: VU; useful (ENVIRONMENTAL USES, MATERIALS). |
|  |
| **Taxaceae** |
| ***Taxus*** |
| *Taxus globosa* Schltdl.; CHIS, COAH, HGO, NLE, OAX, PUE, QRO, SLP, TAMS, VER; Red List: EN; NOM-59: Pr; useful (MEDICINES, MATERIALS). |
|  |
| **Thymelaeaceae** |
| ***Daphnopsis*** |
| *Daphnopsis* *americana* (Mill.) J.R.Johnst.; CHIS, COL, GRO, HGO, JAL, MEX, MICH, MOR, OAX, PUE, QROO, VER, YUC; banked; useful. |
| *Daphnopsis* *ficina* Standl. & Steyerm.; CHIS, GRO, NAY, OAX. |
| *Daphnopsis* *flavida* Lundell; CHIS (endemic). |
| *Daphnopsis* *liebmannii* Nevling; CHIS, OAX (endemic). |
| *Daphnopsis* *megacarpa* Nevling & Barringer; VER (endemic). |
| *Daphnopsis* *mexiae* Nevling; JAL, NAY (endemic). |
| *Daphnopsis* *mollis* (Meisn.) Standl.; CHIS, HGO, JAL, OAX, QRO, QROO, SLP, TAMS, VER (endemic); banked; useful (MATERIALS). |
| *Daphnopsis* *monocephala* Donn.Sm.; CHIS. |
| *Daphnopsis* *nevlingii* J.Jiménez Ram. & J.L.Contr.; GRO, OAX (endemic). |
| *Daphnopsis* *radiata* Donn.Sm.; CHIS, OAX, VER. |
| *Daphnopsis* *tuerckheimiana* Donn.Sm.; CHIS, VER. |
| ***Dirca*** |
| *Dirca mexicana* G.L.Nesom & Mayfield; NLE, TAMS (endemic). |
|  |
| **Ticodendraceae** |
| ***Ticodendron*** |
| *Ticodendron incognitum* Gómez-Laur. & L.D.Gómez; CHIS, OAX; Red List: VU. |
|  |
| **Ulmaceae** |
| ***Ampelocera*** |
| *Ampelocera hottlei* (Standl.) Standl.; CAM, CHIS, OAX, PUE, QROO, TAB, VER; Red List: LC; useful (MEDICINES, MATERIALS). |
| ***Phyllostylon*** |
| *Phyllostylon rhamnoides* (J.Poiss.) Taub.; CAM, COL, GTO, HGO, JAL, NLE, OAX, PUE, QRO, SLP, TAMS, VER, YUC; useful (MATERIALS). |
| ***Ulmus*** |
| *Ulmus* *crassifolia* Nutt.; COAH, NLE, TAMS; Red List: LC; useful (MATERIALS). |
| *Ulmus* *ismaelis* Todzia & Panero; OAX. |
| *Ulmus mexicana* (Liebm.) Planch.; CHIS, GRO, HGO, JAL, OAX, PUE, QRO, SLP, TAB, VER, ZAC; banked. |
| *Ulmus* *serotina* Sarg.; COAH, NLE; useful (ENVIRONMENTAL USES). |
|  |
| **Urticaceae** |
| ***Boehmeria*** |
| *Boehmeria caudata* Sw.; CHIS, GRO, JAL, OAX, VER; Red List: LC; useful (MEDICINES). |
| *Boehmeria pavonii* Wedd.; CHIS. |
| ***Cecropia*** |
| *Cecropia angustifolia* Trécul; OAX, PUE. |
| *Cecropia obtusifolia* Bertol.; CAM, CHIS, CHIH, COL, DGO, GRO, HGO, JAL, MEX, MICH, MOR, NAY, OAX, PUE, QRO, QROO, SLP, SIN, TAB, TAMS, VER, YUC; Red List: LR/lc; banked; useful (FOOD, MEDICINES, MATERIALS). |
| *Cecropia peltata* L.; CAM, CHIS, HGO, GRO, MICH, NAY, OAX, PUE, QRO, QROO, SLP, TAB, VER, YUC; useful (MEDICINES). |
| ***Coussapoa*** |
| *Coussapoa oligocephala* Donn.Sm.; CAM, CHIS, PUE, QRO, QROO, SLP, TAB; Red List: VU. |
| *Coussapoa purpusii* Standl.; CAM, CHIS, COL, GRO, JAL, NAY, OAX, TAB, VER; Red List: VU. |
| ***Discocnide*** |
| *Discocnide mexicana* (Liebm.) Chew; CHIS, COL, GRO, HGO, JAL, MEX, MICH, MOR, OAX, PUE, QRO, SLP, VER, YUC; useful (MEDICINES). |
| ***Myriocarpa*** |
| *Myriocarpa* *bifurca* Liebm.; CHIS, OAX, VER. |
| *Myriocarpa* *cordifolia* Liebm.; CHIS, HGO, OAX, PUE, TAMS, VER (endemic). |
| *Myriocarpa* *cubilgueitzensis* A.K.Monro; CHIS, OAX, TAB, VER. |
| *Myriocarpa* *heterostachya* Donn.Sm.; CHIS, GRO, OAX, TAB, VER; Red List: LC. |
| *Myriocarpa* *longipes* Liebm.; CHIS, COL, GRO, HGO, JAL, MEX, MICH, MOR, NAY, OAX, PUE, QRO, SLP, TAB, TAMS, VER; useful (ENVIRONMENTAL USES). |
| *Myriocarpa* *obovata* Donn.Sm.; CHIS, OAX, VER; Red List: LC. |
| *Myriocarpa* *trifurca* A.K.Monro; CHIS, VER (endemic). |
| ***Pourouma*** |
| *Pourouma bicolor* Mart.; Red List: LC; useful (FOOD). |
| ***Pouzolzia*** |
| *Pouzolzia obliqua* (Wedd.) Wedd.; CHIS, TAB; Red List: LC. |
| *Pouzolzia pringlei* Greenm.; OAX, PUE (endemic). |
| *Pouzolzia purpusii* Brandegee; CHIS, OAX, PUE (endemic). |
| ***Urera*** |
| *Urera* *baccifera* (L.) Gaudich. ex Wedd.; CAM, CHIS, CHIH, COL, DGO, GTO, GRO, HGO, JAL, MEX, MICH, MOR, NAY, OAX, PUE, QROO, SIN, SON, TAB, TAMS, VER, YUC; Red List: LC; useful (MEDICINES). |
| *Urera* *caracasana* (Jacq.) Gaudich. ex Griseb.; CHIS, GRO, HGO, OAX, QRO, SLP, TAB, VER; Red List: LC; useful (MEDICINES, MATERIALS). |
| *Urera* *glabriuscula* V.W.Steinm.; CHIS, OAX, TAB, VER. |
| *Urera* *pacifica* V.W.Steinm.; COL, GRO, JAL, MEX, MICH, NAY, OAX, SIN (endemic). |
| *Urera* *simplex* Wedd.; CHIS, HGO, OAX, PUE, QRO, SLP, TAB, VER; Red List: LC; banked. |
| *Urera* *verrucosa* (Liebm.) V.W.Steinm.; CHIS, COL, GRO, HGO, JAL, MEX, MICH, MOR, OAX, PUE, QRO, VER; Red List: LC. |
|  |
| **Verbenaceae** |
| ***Citharexylum*** |
| *Citharexylum* *affine* D.Don; AGS, CHIS, COL, CDMX, GTO, GRO, HGO, JAL, MEX, MICH, NAY, OAX, PUE, QRO, SIN, TAB, VER, ZAC; Red List: LC; banked. |
| *Citharexylum* *altamiranum* Greenm.; DGO, GTO, HGO, QRO, SLP, ZAC (endemic); banked. |
| *Citharexylum* *berlandieri* B.L.Rob.; COL, DGO, GTO, GRO, HGO, JAL, MEX, NLE, OAX, QRO, SLP, SIN, TAMS, VER; banked. |
| *Citharexylum* *glabrum* (S.Watson) Greenm.; COL, GRO, JAL, MEX, NAY, OAX, QRO, SLP (endemic). |
| *Citharexylum* *hidalgense* Moldenke; HGO, JAL, MEX, MICH, NLE, PUE, QRO, SLP, TAMS, VER, ZAC (endemic). |
| *Citharexylum* *mocinoi* D.Don; CHIS, COL, DGO, GRO, JAL, MEX, NAY, OAX, VER. |
| *Citharexylum* *steyermarkii* Moldenke; CHIS, VER. |
| *Citharexylum* *tetramerum* Brandegee; HGO, OAX, PUE, QRO, SLP (endemic); banked. |
| *Citharexylum* *ligustrinum* Van Houtte; CDMX, HGO, PUE, QRO, SIN, VER (endemic). |
| ***Duranta*** |
| *Duranta* *erecta* L.; BCS, CAM, CHIS, COL, CDMX, GRO, HGO, JAL, MEX, MICH, MOR, OAX, PUE, QRO, QROO, SLP, SIN, TAB, TAMS, VER, YUC; Red List: LC; banked. |
| ***Lippia*** |
| *Lippia* *mcvaughii* Moldenke; CHIS, COL, JAL, MICH, OAX (endemic). |
| *Lippia mexicana* G.L.Nesom; CHIS, COL, CDMX, GTO, HGO, JAL, MEX, MICH, MOR, PUE, QRO, SON (endemic); useful (MEDICINES). |
| *Lippia* *myriocephala* Schltdl. & Cham.; AGS, CAM, CHIS, DGO, GTO, GRO, HGO, JAL, MEX, MICH, MOR, NAY, OAX, PUE, QRO, QROO, SLP, SIN, TAB, TAMS, VER, YUC; Red List: LC; banked; useful (MATERIALS, FUELS, ENVIRONMENTAL USES). |
| *Lippia* *umbellata* Cav.; AGS, CAM, CHIS, CHIH, COAH, COL, CDMX, DGO, GTO, GRO, HGO, JAL, MEX, MICH, MOR, NAY, OAX, PUE, QRO, QROO, SLP, SIN, SON, TAB, TAMS, VER, YUC, ZAC; banked. |
| ***Petrea*** |
| *Petrea xolocotzia* Christenh. & Byng; CHIS; Red List: EN. |
|  |
| **Viburnaceae** |
| ***Sambucus*** |
| *Sambucus canadensis* L.; banked. |
| *Sambucus nigra* L.; AGS, BCN, CAM, CHIS, CHIH, COAH, COL, CDMX, DGO, GTO, GRO, HGO, JAL, MEX, MICH, MOR, NAY, NLE, OAX, PUE, QRO, QROO, SLP, SIN, SON, TAB, TAMS, TLAX, VER, YUC, ZAC; Red List: LC; banked. |
| ***Viburnum*** |
| *Viburnum* *acutifolium* Benth.; CHIS, COL, GRO, JAL, MEX, MICH, MOR.OAX (endemic). |
| *Viburnum* *caudatum* Greenm.; GRO, HGO, PUE, SLP, VER (endemic); banked. |
| *Viburnum* *ciliatum* Greenm.; GRO, HGO, MEX, PUE, SLP, VER (endemic). |
| *Viburnum* *discolor* Benth.; CHIS, OAX. |
| *Viburnum* *disjunctum* C.V.Morton; CHIS, OAX. |
| *Viburnum* *elatum* Benth.; CHIS, COAH, CDMX, GTO, GRO, HGO, JAL, MEX, MICH, NAY, NLE, OAX, QRO, SLP, SIN, TAMS, VER, ZAC (endemic); Red List: LR/cd. |
| *Viburnum* *hartwegii* Benth.; CHIS, COL, HGO, JAL, MICH, OAX, VER; Red List: LC. |
| *Viburnum* *jucundum* C.V.Morton; CHIS, OAX. |
| *Viburnum* *loeseneri* Graebn.; CHIS, GTO, GRO, JAL, MEX, MICH, OAX, SLP, VER (endemic). |
| *Viburnum* *macdougallii* Matuda; COAH, GRO, HGO, MICH, NLE, OAX, PUE, SLP, VER (endemic). |
| *Viburnum* *microcarpum* Schltdl. & Cham.; HGO, MEX, MICH, OAX, PUE, SLP, VER (endemic). |
| *Viburnum* *rufidulum* Raf.; COAH; Red List: LC; useful (ENVIRONMENTAL USES). |
| *Viburnum* *stenocalyx* (Oerst.) Hemsl.; CDMX, GRO, HGO, MEX, MICH, MOR, OAX, PUE, QRO, VER (endemic). |
| *Viburnum* *sulcatum* (Oerst.) Hemsl.; OAX (endemic). |
| *Viburnum* *tiliifolium* (Oerst.) Hemsl.; GRO, HGO, MEX, OAX, PUE, SLP, VER (endemic); banked. |
|  |
| **Violaceae** |
| ***Ixchelia*** |
| *Ixchelia mexicana* (Ging. ex DC.) H.E.Ballard & Wahlert; BCS, CHIH, COL, GTO, GRO, HGO, JAL, MEX, MICH, OAX, QRO, SLP, SIN, SON, TAMS, VER, YUC. |
| *Ixchelia uxpanapana* (T.Wendt) Wahlert & H.E.Ballard; OAX, VER. |
| ***Orthion*** |
| *Orthion* *malpighiifolium* (Standl.) Standl. & Steyerm.; CHIS, OAX, VER. |
| *Orthion* *montanum* Lundell; CHIS (endemic). |
| *Orthion* *oblanceolatum* Lundell; CHIS, OAX, VER. |
| *Orthion* *veracruzense* Lundell; OAX, VER (endemic). |
| ***Rinorea*** |
| *Rinorea deflexiflora* Bartlett; OAX, VER; useful (ENVIRONMENTAL USES). |
| *Rinorea guatemalensis* (S.Watson) Bartlett; CAM, CHIS, OAX, PUE, QROO, TAB, VER; Red List: LC. |
| *Rinorea sylvatica* (Seem.) Kuntze; OAX. |
|  |
| **Vochysiaceae** |
| ***Vochysia*** |
| *Vochysia guatemalensis* Donn.Sm.; CHIS, OAX, TAB, VER; Red List: LC; banked; useful (MATERIALS). |
| *Vochysia tabascana* Sprague; TAB, VER (endemic). |
|  |
| **Winteraceae** |
| ***Drimys*** |
| *Drimys granadensis* L.f.; CHIS, GRO, HGO, OAX, PUE, QRO, SLP, VER. |
|  |
| **Zamiaceae** |
| ***Dioon*** |
| *Dioon holmgrenii* De Luca, Sabato & Vázq.Torres; OAX (endemic); Red List: EN; Cites: II. |
| *Dioon purpusii* Rose; OAX, PUE (endemic); Red List: VU; Cites: II. |
| *Dioon rzedowskii* De Luca, A.Moretti, Sabato & Vázq.Torres; OAX (endemic); Red List: EN; Cites: II. |
| *Dioon spinulosum* Dyer ex Eichl.; CAM, CHIS, OAX, QROO, TAB, VER, YUC (endemic); Red List: EN; Cites: II. |
|  |
| **Zygophyllaceae** |
| ***Guaiacum*** |
| *Guaiacum coulteri* A.Gray; BCN, CHIS, CHIH, COAH, COL, GRO, JAL, MICH, NAY, OAX, PUE, SIN, SON, VER; Red List: LR/cd; NOM-59: A; Cites: II; banked; useful (MEDICINES, MATERIALS, ENVIRONMENTAL USES). |
| *Guaiacum sanctum* L.; CAM, CHIS, MICH, NAY, OAX, QROO, TAB, VER, YUC; Red List: NT; NOM-59: A; Cites: II; useful (MEDICINES, MATERIALS, SOCIAL USES). |
